# Supplementary material for: BRCA2 BRC missense variants disrupt RAD51-dependent DNA repair
Source: eLife. 2022 Sep 13;11:e79183. doi: 10.7554/eLife.79183 (PMC9545528; doi:10.7554/eLife.79183)
Supplement: Figure 1—source data 1. [file elife-79183-fig1-data1.zip › Figure 1-source data 1/Figure1B-source data1/Figure1B-source data1-aligment.docx]

**10 20 30 40 50 60 70 80 90 100 110 120 130**

**....|....|....|....|....|....|....|....|....|....|....|....|....|....|....|....|....|....|....|....|....|....|....|....|....|....|**

**P51587 HUMAN**  1 **------------------------------------MPIGSKERPTFFEIFKTRCNKADLGPISLNWFEELSSEAPPYNSEPAEESEHKNNNYEPNLFKTPQRKPS-YNQLASTPIIFKEQGLTLPLY-Q**

**Q9W157 DROME**  1 **----------------------------------------------------------------------------------------------------------------------------------**

**P97929_MOUSE**  1 **------------------------------------MPVEYKRRPTFWEIFKARCSTADLGPISLNWFEELSSEAPPYNSEPPEESEYKPHGYEPQLFKTPQRNPP-YHQFASTPIMFKERSQTLPLD-Q**

**Q864S8 FELCA**  1 **------------------------------------MPIGCKERPTFFEIFRTRCNKADLGPISLNWFEELCLEAPPYNSEPTEESGYKIS-YEPNLFKTPQRKP--CHQLASTPIIFKEQGLIPPIYQQ**

**O35923 RAT**  1 **------------------------------------MTVEYKRRPTFWEIFKARCSTADLGPISLNWFEELFSEAPPYNTEHPEESEYKPQGHEPQLFKTPQRNPS-YHQFASTPIMFKEQSQTLPLD-Q**

**A5A3F7_STRPU**  1 **METTPTSTQARYVSQGCQKHTKLRHRPLQFVYDFKSGQHSVHSNTSLTSLFEESSLCSSLGEVDTSWFDSERWTSNDETKWKKEDILKTPQASQRENKACS--TPGLFTPDGLSPVARRGHKDYPDCHPL**

**A4ZZ89_MONDO**  1 **------------------------------------MSAGYQGKTTFFEVFKTRCSESDLGPISLNWFEELTSEAPPYNYEISEDPGSKTEITEVNYFKTPQRKPFTYNQLASTPLIFKEQNVNCSLN-S**

**Q8MKI9_CANFA**  1 **------------------------------------MPVGCKERPTFFEIFKTRCNQADLGPISLNWFEELSLEAPPYNSEPTEESGYKIS-YEPNLFKTPQRKP--YNQLASTPIVFR-----EPIYQQ**

**A4ZZ90 XENTR**  1 **------------------------------------MAAPQLGKSVFYDLFSTHCSHSDLGPISLNWFEELTAEALPYKSRTCEDHECSLDDLHENNIKTPKLKSSIYSQLDSTPVIFKERLLSPLFASS**

**140 150 160 170 180 190 200 210 220 230 240 250 260**

**....|....|....|....|....|....|....|....|....|....|....|....|....|....|....|....|....|....|....|....|....|....|....|....|....|....|**

**P51587 HUMAN**  93 **SPVKELDKFKLDLGRNVPNSR--HKSLRTVKTKMDQADDVSCPLLNSCLSESPVVLQCTHVTPQR-----------DKSVVCGSLFHTPKFVKGR-------------QTPKHISESLGAEVDP-DMSWS**

**Q9W157 DROME**  1 **----------------------------------------------------------------------------------------------------------------------------------**

**P97929_MOUSE**  93 **SPFRELG-------KVVASSK--HKTHSKKKTKVDPVVDVASPPLKSCLSESPLTLRCTQAVLQR-----------EKPVVSGSLFYTPKLKEG--------------QTPKPISESLGVEVDP-DMSWT**

**Q864S8 FELCA**  92 **SPLKELG-------KDITNSK--HRSCCTMKSKMDQTNDVTSPPLNSCLSESP-LLRSTHVTPQR-----------EKSVVCGSLFHTPKLTKG--------------QTPKRISESLGAEVDP-DMSWS**

**O35923 RAT**  93 **SPFKELG-------NVVANSK--RKHHSKKKARKDPVVDVASLPLKACPSESPCTPRCTQVAPQR-----------RKPVVSGSLFYTPKLEE----------------TPKHISESLGVEVDP-DMSWT**

**A5A3F7_STRPU**  129 **PDDESASSFLRPKRFRKVPSSIHTTPNSQIITSHQPTSGPTAETIEEEEEEDKGEVAQSADIQFSPYPTWNPTFHKDTPVFMKQLFKTPSTLFGAGDKRACSTPLQRLSTPQRLSRSLGAEPEESDLSWT**

**A4ZZ89_MONDO**  94 **SPLSGPDHCKLDLGKDTAMDKELRKSHCVLKTKLGQANDVTSPPLSTCLSESPAVLRGAYRTPQR-----------EKPVAYGNLLYTPKLMKV--------------NTPKHISESLGAEVDP-DMSWS**

**Q8MKI9_CANFA**  87 **SPLKELDKYRLDSGKDITDSK--HKSCCTMKSKMDRANDVTSPPLNSYLSESP-VLRCTHVTPQR-----------EKSVVCGSLFHTPKLMKG--------------QTPKRISESLGAEVDS-DMSWS**

**A4ZZ90 XENTR**  95 **STELDKR-------QNATDNGNNVRLEKSNCTQMQHQASEVFSPSSRCLNESPAVIKEIFKTPLRNKYLHKTPQCDWKPDICSSLFCTPKLMKN---------------QTGCIKESLGAEVDP-EMSWS**

**270 280 290 300 310 320 330 340 350 360 370 380 390**

**....|....|....|....|....|....|....|....|....|....|....|....|....|....|....|....|....|....|....|....|....|....|....|....|....|....|**

**P51587 HUMAN**  196 **SSLATPPTLSSTVLIVRNEEASETVFPHDTTANVKSYFSNHDESLKKNDRFIASVTDSENTNQR----EAASHGFGKTSGNSFK-VNSCKDHIGKSM--PNVLEDEVYETVVDTSEE---DSFSLCFSKC**

**Q9W157 DROME**  1 **----------------------------------------------------------------------------------------------------------------------------------**

**P97929_MOUSE**  188 **SSLATPPTLSSTVLIARDEEARSSVTPADSPATLKSCFSNHNESPQKNDRSVPSVIDSENKNQQ----EAFSQGLGKMLGDSSGKRNSFKDCLRKPI--PNILEDG--ETAVDTSEE---DSFSLCFPKR**

**Q864S8 FELCA**  186 **SSLATPPTLSSTVLIVRDEEASAAVFPNDTTAIFKSYFCNHDESLKKNDRFIPSGPDSENKSQR----EAKSQGLGKMVGNSCDKVNSCKDPFGNST--LNVLEDGVRERVADVSEE---DSFPLCVPKC**

**O35923 RAT**  186 **SSLATPPTLSSTVLIARDEEAHRNAFPADSPASLKSYFSNHNESLKKNDRFIPSVSDSENKSQQ----EAFSQGLEKMLGDSSSKINRFRDCLRKPI--PNVLEDG--ETAVDTSGE---DSFSLCFPKR**

**A5A3F7_STRPU**  259 **SSLATPPMSSKGCKTVTSSTKKVIEDDSWQTHDVAKVLFQDGGSVAEMDSCDEGDEGLTQPLQVIKLPSITDESQEEEFTIETEDGGESDKSIADGSKAIAALGNEQCSRFDVVGGATSPDNLKNDQDGT**

**A4ZZ89_MONDO**  198 **SSLATPPTLSSTVLIVRDGQTPGARTPDDTTIMLQKYLSQHGERPKRLDRSTSSVPNIENIHAEG---YGTSQELEKMIDDSFDDGNNFKDHGGKMINMPNIMEEEICDPIINTPVE---DDASFNFTGY**

**Q8MKI9_CANFA**  188 **SSLATPPTLSSTVLIVRDEEVSAAVFPNDTTAIFKSCFSNHDESLKKNDRFIPCGPGKENKNQR----EAKSQSLG----NSFGKVNSTKDHFVKST--PNVLEDEVHEKVLDVSEEE--DSFSLCVPKY**

**A4ZZ90 XENTR**  202 **SSLATPPSPTVIIAHANDQPS------GNKHAAIVQSLFPNCENMEANNLPPPETGTQNEDRQIC---AGLEKPGSCSASKAVPEASAISSRAAWKKTVANTVKDEEVSRTVENALEGMEDVLSIFFASE**

**400 410 420 430 440 450 460 470 480 490 500 510 520**

**....|....|....|....|....|....|....|....|....|....|....|....|....|....|....|....|....|....|....|....|....|....|....|....|....|....|**

**P51587 HUMAN**  316 **RTKNLQKVRTSKTRKKIFHEANADECEKSKNQVKE-KYSFVSEVEPNDTDPLDSNVAH---QKPFESGSDKISKEVVPSLACEWSQLTLSGLNGAQMEKIPLLHISSCDQNISEKDLLDTENKRKKDFLT**

**Q9W157 DROME**  1 **----------------------------------------------------------------------------------------------------------------------------------**

**P97929_MOUSE**  307 **RTRNLQKMRMGKTRKKIFSETRTDELSEEARRQTDDKNSFVFEMELRESDPLDPGVTS---QKPFYSQNEEICNEAVQCSDSRWSQSNLSGLNETQTGKITLPHISSHSQNISE-DFIDMKKEGTG-SIT**

**Q864S8 FELCA**  307 **KTRNLQKIKTSKTRKNIFNET-TDECKEAKKQMKENKHSFVSEMEANASDPLDSNVTN---QKPFGNGSDKISKEVVLSSASESCHLTLSGLNGTHMEKLPLLCISSCDQNNSEKDLITTEKECTN-FII**

**O35923 RAT**  305 **RTRNLQKTRMGKMKKKIFSETRTDGLSEEARGQADDKNSFALEIEPRDSEPLDPSVTN---QKPLYSQSGDISSEAGQCSDSIWSQPDPSGLNGTQTRKIPLLHISFHKQSILE-DFIDMKKEGTG-SIT**

**A5A3F7_STRPU**  389 **RTVMNNSLVPPTTSVQSSMTYHGKPHLAKDEEHESVGEYAVPDQYVDEKASVREKVEDMDNAAELKSRTIANGECTDTNVTSKLAHGSHSMKNHLHVPIQPTMSSQQSSIIGSSQTLFNSQESIKNEKSA**

**A4ZZ89_MONDO**  322 **IVGNLRKVKCDKTRK-KYSKTKTNEYEDTKNLSKKENYSSASETELEYCYPLISKAENNEIQKSIKNVNEEMLEDDVPSSATQWSQLNLSGLDVTQIEKTPLPHISFCVQNSEERPANES-NECSS----**

**Q8MKI9_CANFA**  306 **KTRNLQKIKTSKTRKNIFNETKTSECEEAKKQMKENKHSLVSEMEPNDSHPLDWNVTH---EKPFGNGTDKISKEIVLSSASGCSDLTLSSLNGAQMEKTPLLHTS-YDQNNSEKDLIITDKECTN-FIT**

**A4ZZ90 XENTR**  323 **KTPGLRKLKNSTQARRKVESIKSQKCQVSLFKAEEGESILVPLDEHHKCDHEMLMKPE-----QDYKMNAVNQKIAKETTPYEWSQLNICELDITQSQDSTLHAANLCCEDITVNKSDTCNITEAE-KQE**

**530 540 550 560 570 580 590 600 610 620 630 640 650**

**....|....|....|....|....|....|....|....|....|....|....|....|....|....|....|....|....|....|....|....|....|....|....|....|....|....|**

**P51587 HUMAN**  442 **SENSLPRISSLPK-SEKPLNEETVVNKRDEEQHLESHTDCILAVKQAISGTSPVASSFQGIKKSIFRIRESPKETFNASFSGH----MTDPNFKKETEASESGLEIHT------VCSQKEDSLCPNLIDN**

**Q9W157 DROME**  1 **----------------------------------------------------------------------------------------------------------------------------------**

**P97929_MOUSE**  432 **SEKSLPHISSLPE-PEKMFSEETVVDKEHEGQHFESLEDSIAG-KQMVSRTSQAACLSPSIRKSIFKMREPLDETLGTVFSDS----MTNSTFTEEHEASACGLGILT------ACSQREDSICPSSVDT**

**Q864S8 FELCA**  432 **LEDSLPQISGVPKCTEKILNEEIVVNKIDEGQCLESHEDSILAVKQAVFETSLIASPLQGIRKSIFRIRESPEETFSAVFSNN----ITDPNFKEEHEASESVLEKHS------ICSQKEDSLSTSSLDN**

**O35923 RAT**  429 **----FPHISSLPE-PEKMFSEETLVDKEHEGQHLESLEDSISG-KQMVSGTSQTACLSPSIRKSIVKMREPLEETLDTVFSDS----MTSSAFTEELDASAGGLEIHT------ACSQREDSLCPSSVDT**

**A5A3F7_STRPU**  519 **IEETLAFLLATPPSRREKLLNRLGTSSSGIGETTSFEKAACDAAGSTLDGRSDSPVTSNSVPKKSTSDDTLMSPPVGILWKRSIPGHLSGGPERNSLESECSVDAKTNGQVGETPNRPAPDVDMLSPISP**

**A4ZZ89_MONDO**  445 **-------LSTLKT-------VEIAAYKEDEEKITTPNKGSVMLLKQISPEISPPNAISQDRKNSKFKMRQTSEETVSMDFLECSSLCTVNASITTEAKVFENKLENPKSSLDDIICPEKHGLLSPNTDGK**

**Q8MKI9_CANFA**  431 **LENSWPQISNVPKYSEKTLNEEIVVNKINEGQCLESHEDSVVSVKQAIYETTLIASPLQGIRKSIFRIRESPEGMSNAMFSNN----MTNPNFKEP-EASESGLEKHT------ICSQKEDSLCTSSIDD**

**A4ZZ90 XENTR**  447 **EVNEPSTDNVLSNKVGKESTLNVNCINADLNNMASSSNNSVHSLNLSQCEKMDSSEISNSEVGCITKLTTHPAKMATTGELSSVDDCAKKPQERVTISTSFSTLKKQS------KFMYSVNTVLTGHIAS**

**660 670 680 690 700 710 720 730 740 750 760 770 780**

**....|....|....|....|....|....|....|....|....|....|....|....|....|....|....|....|....|....|....|....|....|....|....|....|....|....|**

**P51587 HUMAN**  561 **GSWPATT-----------TQNSVALKNAGLISTLKKKTNKFIYAIHDETSYKGKKIPKDQKSELINCSAQ--FEANAFEAPLTFANADSGLLHSS---VKRSCSQNDSEEPTLSLTSSFGTILRKCSRNE**

**Q9W157 DROME**  1 **----------------------------------------------------------------------------------------------------------------------------------**

**P97929_MOUSE**  550 **GSWPTTL-----------TDTSATVKNAGLISTLKNKKRKFIYSVSDDASLQGKKLQTHRQLELTNLSAQ--LEASAFEVPLTFTNVNSGIPDSS---DKKRCLPNDPEEP--SLTNSFGTATSKEISY-**

**Q864S8 FELCA**  552 **GSWPATI-----------KHTSVALKNSGLISTLKKKTKKFIYVVNDETSYQGLKTQKDQQSGLMNYSAQ--FEANVLEGPLTFANADSGLLHSS---VKKTCLQNDSKEPILSLTNSFGTLLRKVSNKG**

**O35923 RAT**  544 **GSWPTTL-----------TDTSATVKNAGLITTLKNKRRKFIYSVSDDASHQGKKLQTQRQSELTNLSAP--FEASAFEVPFPFTNVDSGIPDSS---IKRSNLPNDPEEPSLSLTNSFVTAASKEISY-**

**A5A3F7_STRPU**  649 **NFAEALCRVTDIASREQPSSNKSTLTVQKCLEVERNLVGDFDSPDPDTAVSHIKVVDQSTSEDHTDIVVKQSLHPTEITGENAIAGHLSAPITSSLPSSAKNKLPKHLSKQEPSPVVPPSRSLAVSNLNT**

**A4ZZ89_MONDO**  562 **GSCATVS-----------KRNSAPLNSTGIISHFKKRTKKFIYVVNNDSSYQGEKIQKTQEFGSVNCSASSHLELNSFEEHHEFTNAISDFLDSS---IRRKCSQFDPKEETAPLTECDKTMMEEYFHES**

**Q8MKI9_CANFA**  550 **GSWPATI-----------KHTSVALKNLGLISSLKKKTKKFIYVINDETSNQGLKTQKDQESRLINLSTQ--FEANAFEGPLTFTNADSGLLHSSS--IKKNCLQNDSEKPALSLTSSFGTILRKVSSNG**

**A4ZZ90 XENTR**  571 **NTSIATR-----------RSLNSHLSSDEPKPHIKENQNEPESNAKYYSNGLQKPVFLEKENDNKGISLCKTISDGQNTSEGARAPDYFEERVPET--KDRCKATLSIREKVVATANCFARKQLETDYPE**

**790 800 810 820 830 840 850 860 870 880 890 900 910**

**....|....|....|....|....|....|....|....|....|....|....|....|....|....|....|....|....|....|....|....|....|....|....|....|....|....|**

**P51587 HUMAN**  675 **TCS-NNTVISQDLDYKEAKCNKEKLQLFITPEADSLSCLQEGQCENDPKSKKVSDIKEEVLAAACHP--VQHSKVEYS----DTDFQSQKSLLYDHENASTLILTPTSKDVLSNLVMISRGKE-SYKMSD**

**Q9W157 DROME**  1 **----------------------------------------------------------------------------------------------------------------------------------**

**P97929_MOUSE**  660 **----IHALISQDLNDKEAIVIEEKPQPYTAREADFLLCLPERTCENDQKSPKVSNGKEKVLVSACLPS-----AVQLS----SISFESQENPLGDHNGTSTLKLTPSSKLPLSKADMVSREK--MCKMPE**

**Q864S8 FELCA**  666 **SSSPNNKIISQDLDYKEAKIKKEKLQSFISTETNCLSSLQEKHCEDDTKSQRVADRKEEILPAVSQPS-VPYSEVEDS----GIHFQTLKSFSSDPDKSS--QLTPHPRDPPSNPVGLSRGRE-SYEVSE**

**O35923 RAT**  656 **----IHALISQDLNDKEAILSEEKPQPYTALEADFLSCLPERSCENDQKSPKVSDRKEKVLVSACRPSGRLAAAVQLS----SISFDSQENPLGSHNVTSTLKLTPSPKTPLSKPVVVSRGK--MCKMPE**

**A5A3F7_STRPU**  779 **QTTAKGRFKYTAAVPRKDGASTSKTPSWLSRAIYVEEDEEEDKTADAVQADTIKDESSKKPWIMKRPCRTATTFKTFS-----TFCKTAPNPPSSTQSQTGPRTSTTPTSSRSIVQSAPPHNKTLVRIRE**

**A4ZZ89_MONDO**  678 **RFP-HNEVTSQDLGFREVEMNKGELESDIT-ETDNQSQSQERSYNDFSKRQRDSHVKECILTATCLSP-RKHTEIELGNIAENSHFTTKNSTLHDSVDTSINSLTQLPQDPQELSVVIYGKKEPLCKVKQ**

**Q8MKI9_CANFA**  665 **ASSPNNKIISQDPDYKEAKINKKKLESFITTETDCLSSLQEKHWEDDAKKQRVSDIKEKVLPTVSHPP-VPHSEVEGS----DIHFQSPESFSFDCDNTS--LLTPSSRDSPSSLVVMSRGKE-SYKISE**

**A4ZZ90 XENTR**  688 **DASAQHEALQFH---AKQYVSCSMLNSNVSNDMQIKPQVLTQACDFRSSAENLVEKTKNQLRRNEDQS---FSSTGIG-----KKPASGEKLVGDCNEGSFLHVKQEISAAQALVGNGYETRDTPDVTSD**

**920 930 940 950 960 970 980 990 1000 1010 1020 1030 1040**

**....|....|....|....|....|....|....|....|....|....|....|....|....|....|....|....|....|....|....|....|....|....|....|....|....|....|**

**P51587 HUMAN**  797 **KLKGN--------NYESDVELTKNIPMEKNQDVCALNENYKNVELLPPEKYMRVASPSRKVQFNQNTNLRVIQKNQEETTSISKIT----VNPDSEELFSDNENNFVFQVANERNNLALGNTKELHETDL**

**Q9W157 DROME**  1 **----------------------------------------------------------------------------------------------------------------------------------**

**P97929_MOUSE**  776 **KLQCE--------SCKVNIELSKNI-LEVN-EICILSENSKTPGLLPPGENIIEVASSMKSQFNQNAKIVIQ-KDQKGSPFISEVA----VNMNSEELFPDSGNNFAFQVTNKCNKPDLGSSVELQEEDL**

**Q864S8 FELCA**  788 **TLKCK--------NHEAGFELTK--TMENSQEIHVLNEHAKKAKLLSTEKYVTEASPSMKVPFNQNAHLTIIQKDQKETTLISKIT----MNPNSEELFPDGDN-FVFKITKERNVPVLGSIKELQDSDL**

**O35923 RAT**  777 **KLQCK--------SCKDNIELSKNIPLGVN-EMCVLSENSETPELLPPLEYITEVSSSVKSQFNQNTKIAVVQKDQKDSTFISEVT----VHMNSEELFPEKENNFAFQVTNESNKPNIGSTVEFQEEDL**

**A5A3F7_STRPU**  904 **IPHSWPS------PKRLRLDEEREECPIGNDLKSLSPAVDEHDGEITKSTPLESDVKSHHVQDVVSLHVEEGVEDQKDLGDFHDKVNSEDLVPSSEGDLHQMAPNVGFSSASGKAIFVSPEALKKAERLL**

**A4ZZ89_MONDO**  805 **KLRFRRNSDSTFITNKVRSELKESFGNKGNQETEVLNEILGDDRELSSEKCKEMTSVSLGAQVNKNAGLFVSLRDQENTNLITEKTA---IELYSEENLHFNEENFIFQTIDKKNIRVTDSSEEFFATSF**

**Q8MKI9_CANFA**  787 **KLKCK--------NHETGFELTKNIPMEKNQDIHVLNADSKNAKLLSTEKHITVASSSVKVQFNQNANLTTIQKDQKETTLISKIT----VNPNSEELFPDDENNFVLKITNESNTPVLGNTKELHDSNL**

**A4ZZ90 XENTR**  807 **HTRSVIS--------NHGKSLWEEETALTELPVPEILSKTFKGFKTASNKKIHISDKNIVKGCDLFKEIEFGIGDAKPCNEENKEP----LKKTPFDVPGYETNLKGFKTASNKEINVSENKFAKGRLLF**

**1050 1060 1070 1080 1090 1100 1110 1120 1130 1140 1150 1160 1170**

**....|....|....|....|....|....|....|....|....|....|....|....|....|....|....|....|....|....|....|....|....|....|....|....|....|....|**

**P51587 HUMAN**  915 **TCVNEPIFKNSTMVLYGDTGDKQATQVSIKKDL-----VYVLAE----ENKNSVKQHIKMTLGQD--------------------LKSDISLNIDKIPEKNND-YMNKWAGLLGPISNHSFGGSFRTASN**

**Q9W157 DROME**  1 **----------------------------------------------------------------------------------------------------------------------------------**

**P97929_MOUSE**  891 **SHTQGPSLKNSPMAVDEDVDDAHAAQVLITKDSDSLAVVHDYTE----KSRNNIEQHQKGTEDKD--------------------FKSNSSLNMK--SDGNSD-CSDKWSEFLDPVLNHNFGGSFRTASN**

**Q864S8 FELCA**  903 **CCVKEPVLENSTMVVYTDMDDKQAAKVSITKGFD-SSNIDDLTE----KDRNSIKQQLRMTLDQD--------------------SKSDITLDSDMKSNGNND-YMDNWARLSDPILNHNFGNGFRTASN**

**O35923 RAT**  894 **SHAKGHSLKNSPMTVDRDLDDEQAGQVLITEDSDSLAVVHDCTK----KSRNTIEQHQKGTADKD--------------------FKSNSSLYLK--SDGNND-YLDKWSEFLDPLMNHKLGGSFRTASN**

**A5A3F7_STRPU**  1028 **EDVDREKPPEEFQLNLSAGDHQCGFQSASGKSIAVSDAALSKAKKIIAEVDSHVEEIDDSSEKQPRSLGFTSARGSVINISAAAMAKARKIMNDINQDEESNPTLHEKREGQTYAKHPVDGGGDGRRIHN**

**A4ZZ89_MONDO**  932 **CYLEESIPKNS-----TEIDYKQTVQELFMEDSFSSEMVPVFTRDSQDKNQNSKLACECISQSKS--------------------SYSKSTIDTNMKPKRNEENYLGKWAQNFDPDSNKSFGCGFKTASN**

**Q8MKI9_CANFA**  905 **CCVRDSVPKNSTMVVCTDLDDKQTAKVSIMKDCY-SSSIDDLTE----RNRSTIKQQLKMTLDQD--------------------SKSDITSDIVRKSNGNSD-YMDNWARLSDPISNHSFENGFKTASN**

**A4ZZ90 XENTR**  925 **KDIEEESGQTTAIMAKDNELFIKPASSNKPDIFKSTCIKGMNPS----ENLDSMPPKKGDVQQSA--------------------GLKHSDQNDVFGFDDPPAKGNVQTLGYFNMSASETDFKGFKTASN**

**1180 1190 1200 1210 1220 1230 1240 1250 1260 1270 1280 1290 1300**

**....|....|....|....|....|....|....|....|....|....|....|....|....|....|....|....|....|....|....|....|....|....|....|....|....|....|**

**P51587 HUMAN**  1015 **KEIKLSEHNIKKSKMFFKDIEEQYPTS---------LACVEIVNTLALDNQKKLSK-----PQSINTVSAHLQSSVVVSDCKNSHITPQMLFSKQDFNSNHNLTPSQKAEITELSTILEESGSQFEFTQF**

**Q9W157 DROME**  1 **----------------------------------------------------------------------------------------------------------------------------------**

**P97929_MOUSE**  994 **KEIKLSEHNVKKSKMFFKDIEEQYPTR---------LACIDIVNTLPLANQKKLSEPHIFDLKSVTTVSTQSHNQSSVS-HEDTDTAPQMLSSKQDFHS-NNLTTSQKAEITELSTILEESGSQFEFTQF**

**Q864S8 FELCA**  1007 **KEIKLSEHNIKKSKMLFKDIEERYPTN---------LACIEIVNTP-LESQEKLSKPHILDPQSINTVSGCVQSSAYVSDSENRHTTPPTLSLKRDFDSNHNLTPSQKAEITELSTILEESGSQFEFTQF**

**O35923 RAT**  997 **KEIKLSEDNVKKSKMFFKDIEEQYPTS---------LDCIDTVSTLQLANKKRLSEPHTFDLKSGTTVSTQCHSQSSVS-HEDTHTAPQMLSSKQDFHSSHNLTPSQKAEITELSTILEESGSQFEFTQF**

**A5A3F7_STRPU**  1158 **EEVVDKVSLPGHASLKGKADGRQRHKGFRPFKAPCLVANIQRPCNLPSPTSISSMNQNTAIDVITESERGESLEMYKDSVPDDLQTPFKQGNAVVPEIQSTTLQVEQKIETSESSIILEQTQDKQENTDS**

**A4ZZ89_MONDO**  1037 **KEIILSEHNIKKGKLFFKDIEQYYNSSSVEVVNSSISVAAENIKTDSDSKKQEKNSFPAWKSEPSHIISKDVQSNIFVLNEKEIDPALEDLCSEKDLDLNHSLTASQKAEITELSTILEETGSQFEFTQF**

**Q8MKI9_CANFA**  1009 **KEIKLSENNIRKSKMLFKDIEEHYPTN---------LACLEIVNTSSLESQKKPSKSHALDPQSINIISGFVQNSTYVSDSESGHTAPPTLSLKQDFDSNRNLTPSQKAEITELSTILEESGSQFEFTQF**

**A4ZZ90 XENTR**  1031 **KDIIISESTLAKGKLIFEDIEDTRYSETG-----------RTIDCKAKGGALSTSNLVMHNTMCKNEPSTSENTNDKAPKRAKVQTPMAENSHLKEPSSDKNAMDAANHQMTLKASAYSPKAASSALPRY**

**1310 1320 1330 1340 1350 1360 1370 1380 1390 1400 1410 1420 1430**

**....|....|....|....|....|....|....|....|....|....|....|....|....|....|....|....|....|....|....|....|....|....|....|....|....|....|**

**P51587 HUMAN**  1131 **RKPSYILQKSTFEVPENQMTILKTTSEECRDADL-------HVIMNAPSIGQVDSSKQFEGTVEIKRKFAGLLKNDCNKSASGYLTDENEVGFRGFYSAHGTKLNVSTEALQKAVKLFSDIENISEETSA**

**Q9W157 DROME**  1 **----------------------------------------------------------------------------------------------------------------------------------**

**P97929_MOUSE**  1113 **RKPSHIAQN-TSEVPGNQMVVLSTASKEWKDTDL-------HLPVDP-SVGQTDHSKQFEGSAGVKQSFPHLLEDTCNKNTSCFLPNINEMEFGGFCSALGTKLSVSNEALRKAMKLFSDIENS-EEPSA**

**Q864S8 FELCA**  1127 **RKPSHLKQKNPCEMPEKHLTISNTTPEEQKDGHL-------RLTINALSISQGDSSKKFEGIIGGKQKLACLSKTSCNKSASGHLTGKNEVEFRGFYSARGTKLNVCSEALQKAKKLFSDLENISEETSV**

**O35923 RAT**  1117 **KNPSHIAQNNTSAVLGNQMAVVRTASEEWKDVDL-------HLPLNPSSVGQIDHNKKFECLVGVKQSSSHLLEDTCNQNTSCFLP-IKEMEFGGFCSALGTKLSVSNEALRKAMKLFSDIENISEEPST**

**A5A3F7_STRPU**  1288 **MN-GQLVDNGELEISEDECLSASQAAREIKAAEEMELVYN-FMQEEDTGFSQLDVSSIVAAGRTEKGAAQILKCADKHKNPIQKEQGHASNCYEHLEMSHHAIEPAPTQDFPNEHIGHFIVPVDQQQRDA**

**A4ZZ89_MONDO**  1167 **RKQSSTVQTLDISTNKADELGNLNTFELQMGVELGDHFGAEYETDNNRSPVQVDLNVNSEVVINVKQRTVYLPKSNSEQGAS--TDIFTESEFKGFCSALGRKMNVSNEALQKAMKLFSDIEEIGEGTPL**

**Q8MKI9_CANFA**  1130 **RKPSHIIQKNPFEMPENQLTILNSTSKEWKDDDL-------HLTTNAPSISQVDS-KKSEGIIGGKQKFACLSRTSCNRSASGYSTDKNEVEFRGFYSARGTKLNVGSEALQKAKKLFSDLENINEETSV**

**A4ZZ90 XENTR**  1150 **KKKPISTTKTSFQLNEHLTESQQAEISELS-----------SILENADSQFDFTQFRKVPSVTEKQNS----TEGGSESQNLNNSDVWKDVDFNDSFAAG----RDHSEGMEAIPSSPGIKELPSAGECC**

**1440 1450 1460 1470 1480 1490 1500 1510 1520 1530 1540 1550 1560**

**....|....|....|....|....|....|....|....|....|....|....|....|....|....|....|....|....|....|....|....|....|....|....|....|....|....|**

**P51587 HUMAN**  1254 **EVHPISLSSSKCHDSVVSMFKIE-NHN-DKTVSEKNNKCQLILQNNIEMTTGTFVEEITENYKRNTENEDNKYTAASRNSHNL-EFDGSDSSKNDTVCIHKDETDLL--------------FTDQHNICL**

**Q9W157 DROME**  1 **----------------------------------------------------------------------------------------------------------------------------------**

**P97929_MOUSE**  1233 **KVGPRGFSSSAHHDSVASVFKIK-KQNTEKSFDEKSSKCQVTLQNNIEMTTCIFVGRNPEKYIKNTKHEDS-YTSSQRN--NLENSDGSMSSTSGPVYIHKGDSDLP---------------ADQGSKCP**

**Q864S8 FELCA**  1250 **EVD-RSFSSSKCNGS-VSMFKKE-NCNNEKKLNEKNNKYRLILQNNIEMTTGIFVAEDTEGYKRNIENKANKYTDASRNVYNFREADGSDSSKNDTVYIHKEENGLP--------------YIDQHDIDL**

**O35923 RAT**  1239 **KVGPRGFSSCAHHDSVASVFKIK-KQNTDKSFDEKSSKCQVTVQNNKEMTTCILVDENPENYVKNIKQDNN-YTGSQRNAYKLENSDVSKSSTSGTVYINKGDSDLPF-------------AAEKGNKYP**

**A5A3F7_STRPU**  1416 **PFGTIGKPTNRNEESPASNLHKE-SQHGVAFVQSHSLSRSCESVQNLHSPKCARKEIQDIYYLKPTEEDDAHETDATNRDATNREAPVADLSMNTVRQLMDDNSILMEEPDRDGTLSRKHEVQKEKISQV**

**A4ZZ89_MONDO**  1295 **QNLRNGPLDRSQKSNIVPAFKIANYANNSKDFKGKDAKPQSNAENN----TGIFVEENMLNCSRDPENKQS--SSPLFISSHKDSSKLKGSVEPDSDNSQREKNDLS--------------SIDQQN-YP**

**Q8MKI9_CANFA**  1252 **EVD-RSFSSSKYNDS-VSMIQIE-DCN-DKNLNEKNNKCRLILQNNIEMTTDIFVEEYTESYRRNTENEGNQCTDAGRNTCNS-ESDGSDSSKNDTVYIHEEENGLP--------------CIDQHNIDL**

**A4ZZ90 XENTR**  1261 **SKETHDSMPDFVTLAPKQGFLVKQNERLFAGFNLASGKQVNIDNDVLKKAAELFNDIDNDKELLSHAKEESRKSNIKHSSKLINNENCGKTEHTSEHLVCQSNVSLP------------------FIKTL**

**1570 1580 1590 1600 1610 1620 1630 1640 1650 1660 1670 1680 1690**

**....|....|....|....|....|....|....|....|....|....|....|....|....|....|....|....|....|....|....|....|....|....|....|....|....|....|**

**P51587 HUMAN**  1367 **KLSGQFMKEGNTQIKEDLSDLTFL-EVAKAQEACHGNTSNKEQLTATKTEQNIKDFETS--DTFFQTASGKNISVAKESFNKIVNFFDQK--PEELHNFS--LNSELHSDIRKNKMDILSYEETDIVKHK**

**Q9W157 DROME**  1 **----------------------------------------------------------------------------------------------------------------------------------**

**P97929_MOUSE**  1344 **ESCTQYAREENTQIKENISDLTCL-EIMKAEETC-MKSSDKKQLPSDKMEQNIKEFN-----ISFQTASGKNTRVSKESLNKSVNIFNRET--DELTVISDSLNSKILHGINKDKMHTSCHKKAISIKKV**

**Q864S8 FELCA**  1363 **KLSSQFIKEGNTQIKEGLSDLTCL-EVVKAEETLHVNTSNKEHLTANTMGRITKDFDIF--DVSFQTASGKNIRVSRASLNKVTNLLDQKCTEEELNNFADSLNSELLSGIDINKADISHHGEMEILKKR**

**O35923 RAT**  1354 **ESCTQYVREENAQIKESVSDLTCL-EVMKAEETCHMKSSDKEQLPSDKMEQNMKEFN-----ISFQTASGKNIRVSKESLNKSVNILDQET--EDLTVTSDSLNSKILCGINKDKMHISCHKKSINIKKV**

**A5A3F7_STRPU**  1545 **DSHTAYQGNADSTITFKTAQGQKDEPGARMDDNNIEEHDPVGFKTAGGKKIQVSESALQKATLLLSDCNSDVETMKESSLEISDELLNQLIPTSSAHDICISDATTLAENVELCKSEDQNAKASECIPPG**

**A4ZZ89_MONDO**  1404 **KMSIQLIRQENYPVKGGLLDLNCLGKAVKDEKIFASNVSGIEELVSNQEEQKMRENENSSHLQSFQTASGRNIMVSKDSFSKVAHLFAEECSVKELNDFSFPFTLKMLN-TALNKSVESSGKKSENLAEN**

**Q8MKI9_CANFA**  1363 **KLFSQFMKEGNTQIKEGLSDLTCL-EVMKAEETSHVTMSNKQQLTAN-TGQNIKDFDTF--YLSFQTASRKNIRVSKESLNKARSLLNQKWTEEELNNFSDSLNSELLPGIDIKKTDISNHEVIENTERK**

**A4ZZ90 XENTR**  1373 **GKNATILVSGNEKSQNQELQLDCL----KCESTDVKHTPQKETINDNSKCNESSLSELS--MKGFQTASGRNIMLSESSIQKARNIFAEEHEDSFTLRCNIQNTIQIPQPVNEPTQFPYVN---------**

**1700 1710 1720 1730 1740 1750 1760 1770 1780 1790 1800 1810 1820**

**....|....|....|....|....|....|....|....|....|....|....|....|....|....|....|....|....|....|....|....|....|....|....|....|....|....|**

**P51587 HUMAN**  1490 **I----------LKESVPVGTGNQLVTFQGQPE-RDEKIKEPTLLGFHTASGKKVKIAKESLDKVKNLFDEKEQGTSEITSFSHQWAKTLKYREACKD-LELACETIEITAAPKCKEMQNSLNNDKNLVSI**

**Q9W157 DROME**  1 **----------------------------------------------------------------------------------------------------------------------------------**

**P97929_MOUSE**  1465 **F-----------EDHFPIVTVSQLPA-QQHPEYEIESTKEPTLLSFHTASGKKVKIMQESLDKVKNLFDET-QYVRKTASFS-QGSKPLKDS---KKELTLAYEKIEVTAS-KCEEMQN-------FVSK**

**Q864S8 FELCA**  1490 **Q----------MKESDLTGTENKSLTLQQRPEYEIKKIKEPTILGFHTASGKRIEIAKESLDKVKNLFDEQEQDKSEMTNFSHRGTKMSKGREECEGGLRLACKTIEITPASKEEEMQKPLEKN-LVSNE**

**O35923 RAT**  1476 **F-----------EEHFPIGTVSQLPALQQYPEYEIESIKEPTLLSFHTASGKKVKIMQESLDKVKNLFDET-QYVRKTTNFGHQESKPLKDREDYKERLTLAYEKIEVTAS-KCEEMQN-------FVSK**

**A5A3F7_STRPU**  1675 **DVLQREGMITHINSGCPVIRNDSTSVDEHPPSTLSSSLPALCQVGFQTAGGKDIKISELALQKAKLVIGENDENPVDHSFDSGSDAHFSASRQDSVSVGFQTARGNKVHVSRSALRRAK------QILES**

**A4ZZ89_MONDO**  1533 **E-----------IEEMAVATKKELMPIQKGPKIENKKWKEYNMVGFHTASGKEVTISKESLTKVKHFFVEENLEND-VTNIKNLETEFLNEREKINEKLVQAYEMVKLNNSQVCEEMQS-------FQDN**

**Q8MKI9_CANFA**  1489 **DKI--------TKESDLIGTENILLILQQRPESKIKKIKESAVLGFHTASGKKIEITKESLDKVKNLFEEKEQDNSEITNFSHRGAKMSKDREECKDGRELACGTTEITTTPEYEETHSSLEKKKLVSNE**

**A4ZZ90 XENTR**  1487 **-----------------LGPKPTTTSGWQEKNILSRSTEKGFMPGFCTAGGKKVSVSDDSLAKAHKLFQEECTFSKEGKLDEVKQNKFMNSEP-------------------------------------**

**1830 1840 1850 1860 1870 1880 1890 1900 1910 1920 1930 1940 1950**

**....|....|....|....|....|....|....|....|....|....|....|....|....|....|....|....|....|....|....|....|....|....|....|....|....|....|**

**P51587 HUMAN**  1608 **ETVVPPKLLSDNLCRQTENLKTSKSIFLKVKVHENVEKETAKSPATCYTNQSPYSVIENSALAFYTSCSRKTSVSQTSLLEAKKWLREGIFDGQPERIN----------TADYVGNYLYENNSNSTIAEN**

**Q9W157 DROME**  1 **----------------------------------------------------------------------------------------------------------------------------------**

**P97929_MOUSE**  1570 **ETEMLPQQN-YHMYRQTENLKTSNGTSS--KVQENIENNVEKNPRICCICQSSYPVTEDSALAYYTEDSRKTCVRESSLSKGRKWLREQ--GDKLGTRNTIKIECVKEHTEDFAGNASYEHSLVIIRTEI**

**Q864S8 FELCA**  1609 **IVVV-PRLLSDNLYKQTENLKIPNRASLKVKVHENTGKETAKKP-TTCTNQSTYSATENSALSFYTGHGRKISVSQSSILEVKKWLRGGELDDQPEKT--------------------------------**

**O35923 RAT**  1586 **QTEMLPQQN-DHMYRQTENLTS-NGSSP--KVHGNIENKIEKNPRICCICQSSYFVTEDSALACYTGDSRKTCVGESSLSKGKKWLREQ--SDKLGTRNTIEIQCVKEHTEDFAGNALYEHSLVIIRTEI**

**A5A3F7_STRPU**  1799 **DIEDIRECSPRVSPMLHPNTSDSDTSQNATCKMDNNHKTVQSEQVPQKCSSVTSTGLFPKTVGFQTASGNSVTVSEDSLSRARQLFAECDAVSGTSSNTESG----------------------------**

**A4ZZ89_MONDO**  1644 **DEKVLPVRT-TDLPKITDSILSNEPSNN--VLFEEKLSEIKENCILSDAKQSTGLPVEMSGLGFYTGHGKSVPVSETSLLEARKWLKEIDLGGKTKASG-------------------------------**

**Q8MKI9_CANFA**  1611 **IAALRPRLLSDNLYKQTENLKISDHASQKVDVHENTEKETAKKP-TMYTNQSTYSAIENSPLTFYTGHGRKISVSEASLFEAKKWLREGEWDDQSERINAAKVNCLKEYPDDYVENPSCGNSSNSAITEN**

**A4ZZ90 XENTR**  1563 **----LSLLTCESVLKQSDGFIEDISTSR-----NALEIRPELYPEGMCSNRASNGSGNNS--EFTAGEGISININQSSLLTTGNVLKNLP----------------------------------------**

**1960 1970 1980 1990 2000 2010 2020 2030 2040 2050 2060 2070 2080**

**....|....|....|....|....|....|....|....|....|....|....|....|....|....|....|....|....|....|....|....|....|....|....|....|....|....|**

**P51587 HUMAN**  1728 **DKNHLSEKQ-DTYLSNSSMSNSYSYHS-----DEVYNDSGYLSKNKLD-SGIEPVLKNVEDQKNTSFSKVISNVKDANAYPQTVNEDICVEELVTSSSPCKNKNAAIKLSISNSNNFEVGP---------**

**Q9W157 DROME**  1 **----------------------------------------------------------------------------------------------------------------------------------**

**P97929_MOUSE**  1695 **DTNHVSENQVSTLLSDPNVCHSYLSQSSFCHCDDMHNDSGYFLKNKID-SDVPPDMKNAEGN---TISPRVSATKERNLHPQTIN-EYCVQKLETNTSPHANKDVAIDPSLLDSRNCKVGS---------**

**Q864S8 FELCA**  1704 **----------------------------------VYNISEYLPKSKVDNSGIEPVVRNVGERENTSVSEIMFTVREADTDPQSVNEDICVQRLVTNFS-CKKENTAIKVTVSDSNNFDSTQKLNSDSNDA**

**O35923 RAT**  1710 **DTSHVSENQASTLFSDPNVCHSYLSHSSFCHHDDMHNDSGYFLKDKID-SDVQPDMKNTEGN---AIFPKISATKEIKLHPQTVN-EECVQKLETNASPYANKNIAIDSAMLDLRNCKVGS---------**

**A5A3F7_STRPU**  1900 **-----------LKAARSPKALGFQTASGSKINISDDALQKARRLISHEMKSFEEEEAAIGNATKGSISNPIPTIVGFKTASENCVEISEESLLKARQFMASDTDLGDRRDTAHPDNLVSGATDMNSEIPF**

**A4ZZ89_MONDO**  1739 **---------------------MFVSSVDVKEDSDIHVKKSVPLYTSNNTSSID--DKNISDKQDAVFLSNSMSSIKHNLKHDTEGSDFCHSNDADFFSEHISDKVMSCLKAQP-----------------**

**Q8MKI9_CANFA**  1740 **DKNHLSEKQGSTYLSNSTMSNSYSYHPGFCHSSEVYNKSEYLSRSKIDNSGIEPVIKNIRERKNIGFSEIMSPGREADTDPQSVNEDICVEKLATNSS-CKNKNTAIKVAISDSNNFNTIQKLNSDSNNS**

**A4ZZ90 XENTR**  1642 **--------------------------------------------SESSGHDVYSVTEHLSTVVKVKRYNDSGHFVNQNLAECNDNHVLSTQKNTANISNRNEACTSLAP---------------------**

**2090 2100 2110 2120 2130 2140 2150 2160 2170 2180 2190 2200 2210**

**....|....|....|....|....|....|....|....|....|....|....|....|....|....|....|....|....|....|....|....|....|....|....|....|....|....|**

**P51587 HUMAN**  1841 **-PAFRIASGKIVCVSHETIKKVKDIFTDSFSKVIKENNENKSKICQTKIMAGCYEALDDSE-DILHNSLDNDECSTHSHKVFADIQSEEILQHNQNMSG---LEKVSKISPCDVSLETSDICKCSIGKLH**

**Q9W157 DROME**  1 **-------------------------------------------------------------------------------------------------------------------------MDQNGASGS**

**P97929_MOUSE**  1810 **-LVFITAHS------QET-ERTKEIVTDNCYKIVEQNRQSKP----DTCQTSCHKVLDDSKDFICPSSSG--DVCINSRKDSFCPHNEQILQHNQSMSG---LKKAATPP---VGLETWDTSKSIRE-PP**

**Q864S8 FELCA**  1800 **VPVYTTASSERVLVAHET--KVAEGFTENCSMAIKQTTKSKP----GKIVAGYRKAPDDSEDTICPNSLDGAECSSPSHKDFAETQSEQTPQLNQSISG---FKKRSEIPPHQINLKTSDICKLSTGKRL**

**O35923 RAT**  1825 **-PVFITTHS------QET-VRMKEIFTDNCSKIVEQNRESKP----DTCQTSCHKALDNSEDFICPSSSG--DVCINSPMAIFYPQSEQILQHNQSVSG---LKKAATPP---VSLETWDTCKSIRG-SP**

**A5A3F7_STRPU**  2020 **PKERQGRSSHEDSSDNSSAQRGSVGTKNSAPGQIGFQTARGGAISVSDESLKKAKQLLEDDAQDGNVQKKREMKQTGVVSSPPMTGFQTGSGKCIHISNSAILKAKQLLSNDFDEYEDLPDVKTSSAPSH**

**A4ZZ89_MONDO**  1829 **-PAFTTASGKTVHISNEAVQKAREMFINNGDKFLKPNIENKSENNQMKNVKDPSKILENKD--ISPKSFDGKKRNLSVNEGSFSNKIYSKKAQLENISAGIALEMDSEVSHCQINSKTSDLYKSTIGKQP**

**Q8MKI9_CANFA**  1869 **VPAYSTVNSKRVFVAHQT--KVTEGFTDNCSMVTKQNTKSKSDTCHAEIVADYPKALDDSE-AIFPNSLGAIECS-PSHKVFADIQSEQTSQLNQSMSG---LEKVSETPPCQINSKTSDRCELPRGKLP**

**A4ZZ90 XENTR**  1707 **-LSFSTASGKSVTVSHDSLQKARLMLSEAANDVTVDTSKQEAAYITPAIRKTEAEKEQNTVDDSDRVHANTFSFSTASGKKVNISGNSLKQVRAVCLSS----DPKETSAALFNVEKSVFNEDVKDVSLL**

**2220 2230 2240 2250 2260 2270 2280 2290 2300 2310 2320 2330 2340**

**....|....|....|....|....|....|....|....|....|....|....|....|....|....|....|....|....|....|....|....|....|....|....|....|....|....|**

**P51587 HUMAN**  1967 **KSVSSANTCGIFSTASGKSVQVSDASLQNARQVFSEIEDSTKQVFSKVLFKSNE-HSDQLTREENTAIRTPEHLISQKGFSYNVVNSSAFSGFSTASGKQVSILESSLHKVKGVLEEFDLIRTEHSLHYS**

**Q9W157 DROME**  10 **HPNRLSQGRGAHARERGATVSAAAN----RSNIIDEMAKICEADRQTFAIARRTRGHERLAVDNSDFVAVEDLILS---------------YAEPTPEDQVEMIMSDFCSSPTYAEDEDEPSHESEPWFR**

**P97929_MOUSE**  1920 **QAAHPSRTYGIFSTASGKAIQVSDASLEKARQVFSEMDGDAKQLSSMVSLEGNE-KPHHSVKRENSVVHSTQGVLSLPKPLPGNVNSSVFSGFSTAGGKLVTVSESALHKVKGMLEEFDLIRTEHTLQHS**

**Q864S8 FELCA**  1921 **QSISYTNACGIFSTASGKCVQVSDAALQKARQVFSKVEDSAKQPFSKVSFKHNEDHSDKFTREENTMIHTPQNLLS-----------SAFSGFSTASGKQVPVSESALCKVKGILEEFDVMRTECGPQRS**

**O35923 RAT**  1935 **QEVHPSRTYGFFSTASGKAVQVSDASLEKARQVFSEIDGDAKQLASMVSLEGNE-KSHHSVKRESSVVHNTHGVLSLRKTLPGNVSSFVFSGFSTAGGKLVTVSESALHKVKGMLEEFDLIRTEHTLQHS**

**A5A3F7_STRPU**  2150 **KHPGPVELEIGFQTGGGRRIQVLQSSLLQARHVICDEKDANQNVLKSESSGQRKKDNSEPVDMDSDESHETDALPDQ----------NIVQGFQTGHGKKVHVSEASIQRAKQFLADENDGSPDQSVVLG**

**A4ZZ89_MONDO**  1957 **KGSS---SVGIFCTANGKPVQVSDNSLKKARQVFSEIANDSEQLLS---IKGSESHPETLIGEENAMLYAKINLPAQKDYLSNMQNLYNNSGFNTASGKQVLISESALQKVKGMLVEFDLMRNECLESIQ**

**Q8MKI9_CANFA**  1992 **KSVSYTNACGIFSTASGKSVQVSDAAIQKAREVFSKLEDSAKQLFPEVSLKDNEEHSEKFTNEENTVIYTSQNLLS-----------SAFSGFRTASGKQVPVSESALCKVKGMLEEFNLIRTESCLQHS**

**A4ZZ90 XENTR**  1833 **QPNVTMPKAVSFSTASGKTVQLSDESLKKARVIFSEIDTCPLMQQQTNESTVEEIVIGGGMTKSKQMPLTTEKVETTRK-------NNGTFGFNTASGKQVSVSESALQKVKDIFQEFDDPDNYEQN---**

**2350 2360 2370 2380 2390 2400 2410 2420 2430 2440 2450 2460 2470**

**....|....|....|....|....|....|....|....|....|....|....|....|....|....|....|....|....|....|....|....|....|....|....|....|....|....|**

**P51587 HUMAN**  2096 **PTSRQNVSKILPR--VDKRNPEHCVNSEMEKTCSKEFKLSNNLNVEGGSSENNHSIKVSPYLSQFQ-QDKQQLVLGTKVSLVENIHVLGKEQASPKNVKMEIGKTETFSDVPVKTNIEVCSTYSKDSENY**

**Q9W157 DROME**  121 **FRN-KRIRTYSRKRDPKSHKAVQNEKRRGSSGLSVQRDLNTSFTSMACDFDASSQKIHEVLLNLSQYFSATATASGPTPVPSQIDLPTEARQD--------------------------------SGDEC**

**P97929_MOUSE**  2049 **PIP-EDVSKILPQPCAEIRTPEYPVNSKLQKTYNDKSSLPSNYK-ESGSSGNTQSIEVSLQLSQMERNQDTQLVLGTKVSHSK-ANLLGKEQTLPQNIKVKTDEMKTFSDVPVKTNVGEY--YSKESENY**

**Q864S8 FELCA**  2040 **PTSRQDVSKMPPPSCVENKTPKHSVNSKLEKAYNKEFKLSSNSKIENGSSEN-HSVQVSPYPSQFK-QDK-QLIQGNKASLVENIHLLEKEQALPKNIKWKL-ETEAFPNLPLKTDTAIHSTDSKDPENY**

**O35923 RAT**  2064 **PTP-EDVSKIPPQPCLESRTPEYSVSSKLQKTYNDKSRSPSNYK-ESGSSGNTQSLEVSPQLSQMERKQETQSVLGTKVSQRK-TNILEKKQNLPQNIKIESNKMETFSDVSMKTNVGEY--YSKEPENY**

**A5A3F7_STRPU**  2270 **FQTGRGKKVQVSAASLQEARHFLTDETAPLQDQNIAVGFQTGHGKKVQISEASLLKAKQFLAHENDPSPDQSVAIGFQTGHGKKVQISEASLQKAKQFLADDTDALPVKNIAVGFQTGHGKKVQISEASL**

**A4ZZ89_MONDO**  2081 **DVTPKGLSPVLCIE--GSETPKHIVNSKSKEICNKELHFSNNCNVEISSSENSRIIEVS--CAQFK-QDMIHPLLETKLTPAKEISFLKKGQVGLKDVDMES--SEAIDGLPVKRNLQVSSPNSKAQETS**

**Q8MKI9_CANFA**  2111 **STSRQDVSKMPPPSCIGKRTPEHSRNSKLDKACNKEFRLSSNCNNQSGSSENHHSIKVSPCPSQLK-RDKPQLLVGSKGSLVENIHPLGKEQALPKNIKTEIGKAETFPNLPVKTNIEFCSTYSKDPENY**

**A4ZZ90 XENTR**  1952 **----KSLVRLPVSSKIKESTPGTKRLVQTAGSSYKNDNLQCKAG-NLRTFQDKQAGKKSLTYSEAAISPIITTVPGNQLTPIQLVKVSTNTSALANKTKPDLYVATAQN----------------TPQND**

**2480 2490 2500 2510 2520 2530 2540 2550 2560 2570 2580 2590 2600**

**....|....|....|....|....|....|....|....|....|....|....|....|....|....|....|....|....|....|....|....|....|....|....|....|....|....|**

**P51587 HUMAN**  2223 **FETEAVEIAKAFMEDDELTDSKLPSHATHSLFTCPENEEMVLSNSRIGK-------RRGEPLILVGEPSIKRNLLNEFDRIIENQEKS--LKASKSTPDGTIKDRRLFMHHVSLEPITCVPFRTTKERQE**

**Q9W157 DROME**  218 **FNAELENLRDDLLQSGFTFEASFYENEHE----------------------------------QEGPGTMADTIYGDSMQSVPTTRLR-----LESGKRNVWSDADSTLKKADVEIAE------------**

**P97929_MOUSE**  2174 **FETEAVESAKAFMEDDELTDSEQ-THAKCSLFTCPQNE--TLFNSRTRK-------RGGVTVDAVGQPPIKRSLLNEFDRIIESKGKS--LTPSKSTPDGTVKDRSLFTHHMSLEPVTCGPFCSSKERQG**

**Q864S8 FELCA**  2166 **FETETVEIAKAFMEDGELTDADLLSHARHFLPTCQHSEETLVSNSRRGK-------RRG-VLVSVGEPPIKRNLLNEFDRIIKNQEKS--LKASKSTPDGIIKDRSLFMHHISLEPVTCGPFSTTKKRQE**

**O35923 RAT**  2189 **FETEAVEIAKAFMEDDELTDSEQ-THAKCSLFACPQNE--ALLNSRTRK-------RGGMAGVAVGQPPIKRSLLNEFDRIIESKGKS--LTPSKSTPDGTIKDRRLFTHHMSLEPVTCGPFCSSKERQE**

**A5A3F7_STRPU**  2400 **QIAKQFLAHEKDAGTDALSKNSAVPGEANENPLSGEPSKMSGFMTGEGRKVQVSEASLQQARNSLGSQAIRNDIGGELSNGTLPHGVQQPTLTSRADGDHLTHQRKDSKEEFHQQERIRESSRGDGSLAT**

**A4ZZ89_MONDO**  2204 **MEREAVEIAKAFMQDDEFTDSELPHNAKKSFFTYKNNEERILTNSRFGK-------RRMEERASYGEPPIKRKLLYEFDRAVENQAKS--LKPSKSSPDGTMKDRRKLMHHISLKPVTCDPFSTAKEWRQ**

**Q8MKI9_CANFA**  2240 **FETETVEIAKAFMEDGELTDSELLSHAKHFVFTCQNTKEMVLLNSRIGK-------RRGDALVSVGEPPIKRNLLNEFDRIIKNQETS--LKASKSTPDGILKDRSLFMHHISLEPISCGPFRTTEERQE**

**A4ZZ90 XENTR**  2062 **FEIEAAESARAFLEDDDLTDMGSAAHETYLYLN-------QTSNTRTGK-------RSRTEGPTPGEPPIKRRLLPEFDRTTDTRQQTGILKPLISTPD-MMRDRRRFLYTLPLKPSTCNPESALRQVT-**

**2610 2620 2630 2640 2650 2660 2670 2680 2690 2700 2710 2720 2730**

**....|....|....|....|....|....|....|....|....|....|....|....|....|....|....|....|....|....|....|....|....|....|....|....|....|....|**

**P51587 HUMAN**  2344 **IQNPNFTAPGQEFLSKSHLYEHLTLEKSSSNLAVSGHPFYQVSATRNEKMRHLITTGRPTKVFVPPFKTKS--HFHRVEQCVR-NINLEENRQK--QNIDGHGSDDSKNKINDNEIHQFNKNNSNQAAAV**

**Q9W157 DROME**  296 **---------NKKLLMGQTQAKNVDIEENTN----------------------------------------------------------------------------------------------------**

**P97929_MOUSE**  2292 **AQRPHLTSPAQELLSKGHPWRHSALEKSPSSPIVSILPAHDVSATRTERTRHSG---KSTKVFVPPFKMKS--QFHGDEHFNSKNVNLEGKNQK--------STDGDREDGNDSHVRQFNK---------**

**Q864S8 FELCA**  2286 **IQNPNFTAPGQKFLSKSHFYEHLALEKSSSNVSISGQPFCTVPATRSEKRGHSITPSKPVKVFVPPFKTKS--RFLQDEQHISKNTHVEENKQKP-NNIDEHSSGDSKNNINNSEIHQLNKNNSSQAATM**

**O35923 RAT**  2307 **TQSPHVTSPAQGLQSKEHPSRHSAVGKSSSNPTVSAL--------RSERTRHSVS-DKSTKVFVPPFKVKS--RFHRDEHFDSKNVNLEGKNQK--------SADGVSEDGNDSDFPQFNK---------**

**A5A3F7_STRPU**  2530 **PSNLPAPGGLAKKSMVDTRNRQSNQQRNKGTGSRGTGQKPYKPPRRVDDKGSTSAAGLIPRCSNPTVVLNDRTKQCKARQSSPPTSLSTSSHCVSDGSLHRAGPSVKSCPDQDATNQSQSQLEATPTIAE**

**A4ZZ89_MONDO**  2325 **MRYPNFTAPGQELASKYHFLKHQTLENSSHNLLLQSPSYKD-SSTDNGKVKSISVRGKPAKVFVPPFKTNSNSSTATDDEFVRKRLKVKVKEYTPENEKQKYGDAHNSGDSENNENYLSDKDNSNQATTD**

**Q8MKI9_CANFA**  2361 **IQNPNFTAPGQEFLPKSHFYEHLASEKSSSNLSVSRQPFCMVPATGNEKRRHLIAPGKPVKVFVPPFKTKS--HFHRDEQCISKNTKLEKNKQNS-KDIDELGSGDSEKNINDSGIHQLKKNNSNQAATI**

**A4ZZ90 XENTR**  2175 **------CIPTQAHLHSKVKIFHQSLPIKSPDVASDSTSKSYSPTAAKETINCSSASKIPAKKFVPPFKKTVATLADNQSNSVQNGSSDGLIESIVYPKEDKVETICSSKDQFDDS---------------**

**2740 2750 2760 2770 2780 2790 2800 2810 2820 2830 2840 2850 2860**

**....|....|....|....|....|....|....|....|....|....|....|....|....|....|....|....|....|....|....|....|....|....|....|....|....|....|**

**P51587 HUMAN**  2469 **TFTKCEEEPLDLITSLQNARDIQDMRIKKKQRQRVFPQPGSLYLAKTSTLPRISLKAAVGGQVPSACSHKQLYTYGVSKHCIKINSKNAESFQFHTEDYFGKESLWTGKGIQLADGGWLIPSNDGKAGKE**

**Q9W157 DROME**  317 **----------FILEGIPLSEWLTPMELPEISKDVIKHIPD----------KKLKLEPSSQKEQKSSKDSNASKVRGASKQSCDINTKNEGTTILDQPNAAEQENLLN----------------DGELLEE**

**P97929_MOUSE**  2399 **----------DLMSSLQSARDLQDMRIKNKERRHLRLQPGSLYLTKSSTLPRISLQAAVGDRAPSACSPKQLYIYGVSKECINVNSKNAEYFQFDIQDHFGKEDLCAGKGFQLADGGWLIPSNDGKAGKE**

**Q864S8 FELCA**  2413 **VFTKCEKEPLDLIASLQNARDIQDMRIREKRKQHIFPQPGSLFLAKTSTVPRISLRVAVEGRVPSACSHKQLYMYGVSKHCVKINSKNAESFQFHTQDYFGKEVQWAKEGIQLADGGWLIPSNDGKAGKE**

**O35923 RAT**  2408 **----------DLMSSLQNARDLQDIRIKNKERHHLCPQPGSLYLTKSSTLPRISLQAAVGDSVPSACSPKQLYMYGVSKACISVNSKNAEYFQFAIEDHFGKEALCAGKGFRLADGGWLIPSDDGKAGKE**

**A5A3F7_STRPU**  2660 **G-------GCSFVARWAEARRAQQQRIDAKRKKVIKPELGSLWRSRK-EQDRISLGEFVNHQYPCSMSDSELFGHGILSSTLSVRPSNAEGYEFAGEDHFSPSCLESDRGIPLDDGGRLVLSPNGLAGKK**

**A4ZZ89_MONDO**  2454 **IFKECGNLHLEMIENLQHAREMQDMRIKKKQTQKIHPQAGSLYLRKTSTLPRSSLRVVVEGKVPSTYSSKQLYMYGITKQCLKINSKNSESFKFHSEDYFSKEHLLDGNGIQLADGGWLIPTDEGKLGKE**

**Q8MKI9_CANFA**  2488 **IFTKNEKEPLDLITNLQNARDIQDMRIKKKQRQHIFPQPGSLYLAKTSTLPRISLREAVEGRVPSACSHKQLYMYGVSKHCVKINSKNAESFQFHAQDYFGKEGLWSGEGIQLADGGWLIPSNDGKIGKE**

**A4ZZ90 XENTR**  2284 **-------DILQMTSNLRCSKDLQEMRIRKKLRQKIKPHPGSLYRLKMSHVKRISLQSAVAERCPTLYSREQLYRYGIVKNHIGVSSENALSFQFHCSNYFTKELLLSGNGVQLADGGWLIPTEQGNAGKE**

**2870 2880 2890 2900 2910 2920 2930 2940 2950 2960 2970 2980 2990**

**....|....|....|....|....|....|....|....|....|....|....|....|....|....|....|....|....|....|....|....|....|....|....|....|....|....|**

**P51587 HUMAN**  2599 **EFYRALCDTPGVDPKLISRIWVYNHYRWIIWKLAAMECAFPKEFANRCLSPERVLLQLKYRYDTEIDRSRRSAIKKIMERDDTAAKTLVLCVSDIISLSANISETSSNKTSSADTQK--VAIIELTDGWY**

**Q9W157 DROME**  412 **FLFNDWQPMQCSNGPSTSKNAIQ----------GPKENINSINLDDKEQPEKQTPNKSQTISSHQLNGIRKKSFKFIEVSEEMKIK-GEEFVDKVVSGLYHFSHKCNLRTEEYSDNHS-----QVMESTQ**

**P97929_MOUSE**  2520 **EFYRALCDTPGVDPKLISSIWVANHYRWIVWKLAAMEFAFPKEFANRCLNPERVLLQLKYRYDVEIDNSRRSALKKILERDDTAAKTLVLCISDIISPSTKVSETSGGKTSGEDANK--VDTIELTDGWY**

**Q864S8 FELCA**  2543 **EFYRALCDTPGVDPNLISRIWVYNHYRWIIWKLAAMEFAFPKEFANRCLSPERVLLQLKYRYDMEIDRSKRSAIKKIMERDDTAAKTLVLCISETISSSTDLSETSGSKTSGVGTKN--VGIVELTDGWY**

**O35923 RAT**  2529 **EFYRALCDTPGVDPKLISSVWVSNHYRWIVWKLAAMEFAFPKEFANRCLNPERVLLQLKYRYDVEIDNSSRSALKKILERDDTAAKTLVLCVSDIISLSTNVSETSGSKASSEDSNK--VDTIELTDGWY**

**A5A3F7_STRPU**  2782 **EFYSALLDTPGVDPKLLKEEWVFNHYKWIIWKAAAMEVAYPLQLGGRFLTPNWVLLQLKYRYDREIDHSQRPALRKILERDDAASRRMVLCVAAIGNTGSSAGHDGKGAEQRKSLQAVSHPTLELTDGWY**

**A4ZZ89_MONDO**  2584 **EFYRALCDTPGVDPKLITKTWVYNHYRWIIWKLAAMEFTFPKEFASRCLTPEWVLLQLKYRYDIEIDESRRSAIKKIMERDETPAKTLVLCVSEIISSGTNISITANSKNNSVETKKE-SAVIEVTDGWY**

**Q8MKI9_CANFA**  2618 **EFYRALCDTPGVDPNCISRVWVYNHYRWIIWKLAAMEFAFPKEFANRCLSPERVLLQLKYRYDVEIDKSRRSAIKKIMERDDTAAKTLVLCISEIISSSADISETSSSKTSSVGTKK--VGIIELTDGWY**

**A4ZZ90 XENTR**  2408 **EIYRAFCDTPGVDPKLISAEWVHNHYRWIVWKLAAMEVRFPKTFACRCLTPERVLLQLKYRYDVEIDKSQRSAIKKIMERDDSPAKTLVLCIAKIISQGTRLPNACSNKTEPADSKES-SAVIEVTDSWY**

**3000 3010 3020 3030 3040 3050 3060 3070 3080 3090 3100 3110 3120**

**....|....|....|....|....|....|....|....|....|....|....|....|....|....|....|....|....|....|....|....|....|....|....|....|....|....|**

**P51587 HUMAN**  2727 **AVKAQLDPPLLAVLKNGRLTVGQKIILHGAELVGSPDACTPLEAPESLMLKISANSTRPARWYTKLGFFPDPRPFPLPLSSLFSDGGNVGCVDVIIQRAYPIQWMEKTSSGLYIFRNEREEEKEAAKYVE**

**Q9W157 DROME**  526 **CAEFKSAPSKPIEILDGKETYNAIAKVDVGEINGKFSPLNNDTIAEPEFCGFRTASNKAIPISEKMKIKT-------------------------------AEFMAEFQS------KETIQQNDYLVHQP**

**P97929_MOUSE**  2648 **AVRAQLDPPLMALVKSGKLTVGQKIITQGAELVGSPDACAPLEAPDSLRLKISANSTRPARWHSRLGFFRDPRPFPLPLSSLFSDGGNVGCVDIIVQRVYPLQWVEKTVSGLYIFRSEREEEKEALRFAE**

**Q864S8 FELCA**  2671 **AIKAQLDPPLLALVKKGRLTVGHKIIIHGAELAGSPDACTPLEAPESLILKISANSTRPACWYAKLGFFPDPRPFPLPLSSLFSDGGNVGCVDVVIQRTYPIQWMEKTPSGLCIFRNEREEEREATKYAE**

**O35923 RAT**  2657 **AVKAQLDPPLLALVKSGRLTVGQKIITQGAELVGSPDACAPLEAPDSLRLKISANSTRPARWHSKLGFFHDPRPFPLPLSSLFSDGGNVGCVDVIVQRVYPLQWVEKTVSGSYIFRNEREEEKEALRFAE**

**A5A3F7_STRPU**  2912 **SIPAAIDPPLANHVRSGRIVCGTKLCISGAELVGAQDACSPLEIPEGLKLKITANSTRRARYDARLGLQADPRPFPLPMTSLHPEGGTIGCLDVLILRTYPMQFMEKLPEGGSVFRNAKEEAKAAALHAS**

**A4ZZ89_MONDO**  2713 **AIKALLDSSLLAILQKGKLTTGQKIITHGAELIGSQEACTPLEAPESLMLKLSANSTRPARWYAKLGFFSDPRPFPLPLSSLFSEGGNVGCVDIVVQRVYPTQWMEKTLSGSYIFRNERAEEKEALKYAE**

**Q8MKI9_CANFA**  2746 **AIKAQLDPPLLALVKNGRLTVGQKITIHGAELVGSPDACTPLEAPESLMLKISANSTRPACWYTKLGFSPDPRPFPLPLSSLFSDGGNVGCVDVVVQRAYPIQWMERTPSGLCIFRNEREEEKEATKYAE**

**A4ZZ90 XENTR**  2537 **GIKVLLDPCLTALLHKGRLFIGQKLIVHGAELIGSDDACSPLEAPESLMLKIAANSTRPVRWHTKLGYFKDPRPFCLHLSSLLSEGGVVGCVDVVIQRIYPMQWMEKMANGLYVFRNDRAEEREAEKHSA**

**3130 3140 3150 3160 3170 3180 3190 3200 3210 3220 3230 3240 3250**

**....|....|....|....|....|....|....|....|....|....|....|....|....|....|....|....|....|....|....|....|....|....|....|....|....|....|**

**P51587 HUMAN**  2857 **AQQKRLEALFTKIQEEFEEHEENT------TKPYLPSRALTRQQVRALQDGAELYEAVKNAADPAYLEGYFSEEQLRALNNHRQMLNDKKQAQIQLEIRKAMESAEQKEQGLS-RDVTTVWKLRIVSYSK**

**Q9W157 DROME**  619 **NDKPTSVGLDTALKRSIESSEEMR----------------------------------SKASKLVVVDTTMREPHQPTLDPVCRDLNESQ----FFGFRTASNKAIEITEAMEKRGAMFLAQSKATDQLN**

**P97929_MOUSE**  2778 **AQQKKLEALFTKVHTEFKDHEEDT------TQRCVLSRTLTRQQVHALQDGAELYAAVQYASDPDHLEACFSEEQLRALNNYRQMLNDKKQARIQSEFRKALESAEK-EEGLS-RDVTTVWKLRVTSYKK**

**Q864S8 FELCA**  2801 **AQQKKLEVLFNKIQAEFEKHDENI------TKRCVPLRALTRQQVCALQDGAELYEAVKNAPDPASLEAYFSEEQIRALNNHRQMLNDKKQAQIQLEFRKAMESAEQGEQMLP-RDVTTVWKMRIISYGK**

**O35923 RAT**  2787 **AQQKKLEALFTKVHTELKEHEEDI------AQRRVLSRALTRQQVHALQDGAELYAAVQDASDPEHLETCFSEEQLRALNNYRQMLSDKKQARIQSEFRKALEAAEK-EEGLS-RDVSTVWKLRVTSYKK**

**A5A3F7_STRPU**  3042 **RKQNKMEQLFTQIQKQFEAKQATKGQGGKRRRSLPSSRSRNPVEVEKLQSGEELFEAMEAAIDPAAFESVLSERQCSTLHAYRRLQNEVKQADLQAAFNRSLAQQNK--EGKFERTVVPLMKVRVGDYNA**

**A4ZZ89_MONDO**  2843 **CQQKKLEALLTKIQAEFEKHEENS------ATQHRQSHILTRQQVRALQDGADLYEAVKNAPDPAYVEGCFSKEQLRTLNHHRQILNDKKQEQLQAEFRKAIAAAELEEHGLAKRDVTTVWKLRVVNYKK**

**Q8MKI9_CANFA**  2876 **IQQKKLEVLFNKIQAEFEKNDENI------TKQCIPSCALTRQQICALQDGAELYEAVTNAPDPSDLEGYFSEEQLRALNNHRQMLNDKKQAQIQLEFKKAMESAEQGEQILP-RDVTTVWKLRIISYRK**

**A4ZZ90 XENTR**  2667 **NQQKKLEMLFSKIQAEFEQREVTCN-----RRKGLRRRSLNAQQMQTLQDGAEIYEAIQNESDPGYLESYLSAEQLKALNHHRQLLNDKKQALIQAEFRKAIECSEQDANGCTRRDVTPVWKLRIADYRN**

**3260 3270 3280 3290 3300 3310 3320 3330 3340 3350 3360 3370 3380**

**....|....|....|....|....|....|....|....|....|....|....|....|....|....|....|....|....|....|....|....|....|....|....|....|....|....|**

**P51587 HUMAN**  2979 **--KEKDSVILS-IWRPSSDLYSLLTEGKRYRIYHLATSKSKSKSERANIQLAATKKTQYQQLPVSDEILFQIYQPREPLHFSKFLDPDFQPSCSEVDLIGFVVSVVKKTGLAP------FVYLSDECYNL**

**Q9W157 DROME**  710 **------------GWQP-SDFPDVLPTSPNNEIHSINVENNKAVN---TKTVSETEFFGFRTASNKGIVISENTKIKVAQFMSEFQAADASTDSNQPTVISEES---------------------------**

**P97929_MOUSE**  2899 **--KEK-SALLS-IWRPSSDLSSLLTEGKRYRIYHLAVSKSKSKFERPSIQLTATKRTQYQQLPVSSETLLQVYQPRESLHFSRLSDPAFQPPCSEVDVVGVVVSVVKPIGLAP------LVYLSDECLNL**

**Q864S8 FELCA**  2923 **--KEKDSVTLS-IWRPSSDLYSLLTEGKRYRIYHLATSQSKSKSERAHIQLTATKKTQYQQLPASDELLFQVYQPREPLYFNKLLDPDFQPPCSEVDLIGFVVSVVKKIGFAP------LVYLSDECHNL**

**O35923 RAT**  2908 **--REK-SALLS-IWRPSSDLPSLLTEGQRYRIYHLSVSKSKNKFEWPSIQLTATKRTQYQQLPVSSETLLQLYQPRELLPFSKLSDPAFQPPCSEVDVVGVVVSVVKPIGLAP------LVYLSDECLHL**

**A5A3F7_STRPU**  3170 **PINKGQQTTFLTLWRPPDDLVTELVEGKRFRISSVATSAGRNMPGMCPVQLASTRATRYEELPAASLKLQRSYIPRAAASMQWLGRGYTQAAYGELDAVGIVVSLDEPSRQHPGSSQYHAVYLADQDAAV**

**A4ZZ89_MONDO**  2966 **--HEKDSVILS-IWRPLSDIYALLKEGNRYRIYNLATSPSKSKSDRAHIRLTATKKTQYQQLPVSHEALSQVYQPREAVPLDKLMEPSFRPLCAEVDVVGVVISVTKKPGVAP------LVYLSDEYYNL**

**Q8MKI9_CANFA**  2998 **--KEKDSVTLS-IWRPSPDLYSLLIEGKRYRIYHLAASQSKSKSGKANTQLTATKKTQYQQLPASDEILSQVYQPREPLYFNKLLDPDFQPPCSEVDLIGFVVSVVKKIGLAP------VVYLSDECHNL**

**A4ZZ90 XENTR**  2791 **--YETDAAYILNIWRPLPDVLSLLKEGCRYKMYHLAASTSKGKSLAADLQLTATKKTRFQQLQPSESILEQIYSPREVTDFSRFQEPLFSAPYAEVDLVGLIISIYKKTGAAP------VVYISDESHNI**

**3390 3400 3410 3420 3430 3440 3450 3460 3470 3480 3490 3500 3510**

**....|....|....|....|....|....|....|....|....|....|....|....|....|....|....|....|....|....|....|....|....|....|....|....|....|....|**

**P51587 HUMAN**  3101 **LAIKFWIDL----NEDIIKPHMLIAASNLQWRPES----KSGLLTLFAGDFSVFSASPKEGHFQETFNKMKNTVENIDILCNEAENKLMHILHANDP------------------KWSTPTKDCTSGPYT**

**Q9W157 DROME**  797 **----------------------------------------RNIDAKFVDEAAAEDSANKPTFCNVQSLKNTSDIEHFKHDFFVEHSAKEEHPLCSQP------------------LVRTPRRSQEIHSSL**

**P97929_MOUSE**  3020 **LVVKFGIDL----NED-IKPRVLIAASNLQCQPES----TSGVPTLFAGHFSIFSASPKEAYFQEKVNNLKHAIENIDTFYKEAEKKLIHVLEGDSP------------------KWSTPNKDPTREPHA**

**Q864S8 FELCA**  3045 **LAIKVWTDL----NEDIVKPHTLIAASNLQWRPES----KSGIPTLFAGDFSRFSASPKEGHFQETFHKMKNTIENVETFCNDAENKLVHILNANSP------------------KVSTPMKDYASEPHT**

**O35923 RAT**  3029 **LVVKFGIDL----NED-IKPRVLIAASNLQWRPES----TSRVPTLFAGNFSVFSASPKEAHFQERVTNMKHAIENIDTFYKEAEKKLIQVLKGDSP------------------KWSTPNKDPTREPYP**

**A5A3F7_STRPU**  3300 **MVIKFWTSPSALNLDDMLKPGSFISVSNLQYRSISSTLSAVALPTATASDYTVFTPNPKAGYLQVALSQLRGAIKDVPKCLELMQRKVQQLKDAPSQPAYPGSTPRNHPYTAGRERVTTPLAGLSRARSG**

**A4ZZ89_MONDO**  3088 **LAVKFWIDL----NEDVIKLHTLIAASNLQWKTES----RAGIPMLFAGEFSVFSASPKQSHFQERFNKLKNSVENIDLFCDGAEKKLSHLLDTHNS------------------KQPNAVREYNLDSPS**

**Q8MKI9_CANFA**  3120 **LAIKFWTDF----NEDIIKPYTLIAASNLQWRPEA----KSGIPTLFAGDFSRFSASPKEEHFQETFHKMKNTVENIGMFYNDAENKLVHILNANDP------------------KLSTPTKDYASEPHT**

**A4ZZ90 XENTR**  2914 **VALKFWTDLGQLGLEEITKPRTYISASNLRWRSDC----IEGIPTLYVGDLANISSNPKESHLQRAIQKLKLSVQNVQDFWNSSQTALMKTLQINST------------------DTTECSKNPTTPTWK**

**3520 3530 3540 3550 3560 3570 3580 3590 3600 3610 3620 3630 3640**

**....|....|....|....|....|....|....|....|....|....|....|....|....|....|....|....|....|....|....|....|....|....|....|....|....|....|**

**P51587 HUMAN**  3205 **AQIIPG-----TGNKLLMSSPNCEIYYQSPLSLCMAKRKSVSTPVSAQMTSKS-CKGEKEIDD-------------------------QKNCKKRRALDFLSRLPLPPPVSPICT-FVSPAAQKAFQPP-**

**Q9W157 DROME**  870 **SQLAGK----------------------SPLDQATKKSVIARRNLLSLKRKRKIILSTETSTS--------------------------------------CALPTMERFAPKPSSTSTPLADRDLNRS-**

**P97929_MOUSE**  3123 **ASTCCASDLLGSGGQFLRISPTGQQSYQSPLSHCTLKGKSMPLAHSAQMAAKS-WSGENEIDD-------------------------PKTCRKRRALDFLSRLPLPSPVSPICT-FVSPAAQKAFQPP-**

**Q864S8 FELCA**  3149 **IQTVLG-----LGNKLSMSSPNSEMNYQSPLSLCKPKAKSVPTPGSAQMTSKSCYKGERELDD-------------------------PKTCKKRKALDFLSRLPLPPPVSPICT-FVSPAAQKAFQPP-**

**O35923 RAT**  3132 **ASTCSASDLAS-GGQLPRSSPTDQQSYRSPLSCCTPTGKSTPLAHSAWMAAKS-CSGENEIED-------------------------PKTCRKKRALDLLSRLPLPPPLSPVCT-FVSPAAQKAFQPP-**

**A5A3F7_STRPU**  3430 **IQSSPPTSVTSQGQPYQLMTVRSCPKNFPKTSQPTRDENLNPRIQVAGSTPSHSHPGTSRVSTNPTPRTPCQSQVSAMSGVDVSPRVLAQHVELERRSQLLSRIPSPPPLSPLPYPAASPALKRGFKIPS**

**A4ZZ89_MONDO**  3192 **PQAKLG------------LGPMISPFHREPNHHCP-EGNSGATISSVKISPTSDCRGDRKMDD-------------------------PKICKKRRSLDFLSRLPLPPPISPIRT-FVSPAAQKAFQPP-**

**Q8MKI9_CANFA**  3224 **AQIVLG-----IGNKFLMSSPNNEMNYQSPLSLCKPKEKSVPIPGSTQMTSKSYCKEEKEMDD-------------------------PKTCKKRRALDFLSRVPLPPSVSPICT-FVSPAAQKAFQPP-**

**A4ZZ90 XENTR**  3022 **SDVSAR------------------SGYLTPLHHSGKRLLN----SVHTSDPQTENPGCSKEIQ-------------------------LKTCKKRKALDFLNRIPSPPPVTPVRP-FVSPSLQKAFRPP-**

**3650 3660 3670 3680 3690 3700 3710 3720 3730 3740 3750 3760 3770**

**....|....|....|....|....|....|....|....|....|....|....|....|....|....|....|....|....|....|....|....|....|....|....|....|....|....|**

**P51587 HUMAN**  3301 **------------RSCGTKYETPIKKKELNSP-QMTPFKKFNEISLLESNSIADEELALINTQALLSGSTGEKQFISVSESTRTAPTSSEDYLRLKRRCTTSLIKEQESSQASTEECEKNKQDTITTKKYI**

**Q9W157 DROME**  938 **----------------------------------------KDCTKNRQDAEDMSPICMQPKKSRRLGLSRSRY---------------------------------------------------------**

**P97929_MOUSE**  3224 **------------RSCGTKYATPIKKE-PSSPRRRTPFQKTSGVSLPDCDSVADEELALLSTQALTPDSVGGNEQAFPGDSTRNPQPAQRPDQQVGPRSRKESLRDCRGDSSEKLAVES------------**

**Q864S8 FELCA**  3246 **------------RSCGTKYETPIKKRELNSP-QMTP-LKFNDTSLVESDSIADEELALINTQALLSGLAGEDQLMSLNDSPRTAPTSSKDYVRPKSYPTAPGIRDCENPQASTEGGEPDVQDTDTVKRSS**

**O35923 RAT**  3232 **------------RSCGTKYPTPLKKEGPSSPWSRAPFQKASGVSLLDCDSVADEELALLSTQALVPHSVGGSEQVFPSDSTRTEGPSASTEARPANRSKRESLRDCRDDSDGKLAAETVPDYS-------**

**A5A3F7_STRPU**  3560 **PLVRGSQKPDPEKSLRKRLRDELEEQDDKDSEQQCKQQSDIGIDEGENTYKKQKTDSHVDDSNQKEVKSQTDNHSNLDDTIEEYEDRGEDDFGDDLGLSASGLRELEMSWSQSEPLPEPAARDRTLKMNT**

**A4ZZ89_MONDO**  3281 **------------RSCGNKYNVPVKKNTSNSP--QMTIKTLSGTTMAENDLIADEELALINTQALLPNSSE------------------------------------------------------------**

**Q8MKI9_CANFA**  3321 **------------RSCGTKYETLMKK-ELNSP-QMTP-RKFNDLSLLESDSIADEELAMINTQALLLGSPGEHQLVSVSDSTRTAPTSSKDYLGLKRHSTAPGVRGPESPQACTRKREPRVQNTSDLKRTS**

**A4ZZ90 XENTR**  3102 **------------RSCSVQKLGPETKGNTENVQGTTP-ECTKDLAKLEGEFVADEELAMINTQALLLGLEEEKKKTEQKTSRTAGKMTAHESPIENASPVPAQEQQTEEALNIPVGNSEKSYLCLRKRKRK**

**3780 3790 3800 3810 3820 3830 3840 3850 3860 3870 3880 3890 3900**

**....|....|....|....|....|....|....|....|....|....|....|....|....|....|....|....|....|....|....|....|....|....|....|....|....|....|**

**P51587 HUMAN**  3418 **----------------------------------------------------------------------------------------------------------------------------------**

**Q9W157 DROME**  971 **----------------------------------------------------------------------------------------------------------------------------------**

**P97929_MOUSE**  3329 **----------------------------------------------------------------------------------------------------------------------------------**

**Q864S8 FELCA**  3363 **MRLQRRQQQT------------------------------------------------------------------------------------------------------------------------**

**O35923 RAT**  3343 **----------------------------------------------------------------------------------------------------------------------------------**

**A5A3F7_STRPU**  3690 **CQAEEGCPSRSKDSLDEDKDALPTDMSLEKDNHRISGLQDEKHVASGIDKAVEGTVEACKDVVGVHETERDVVPCTKPVQPEQLADHTGVNCDDPTHPTTNNSNLTMANASETTSICKVEDSGALILPPH**

**A4ZZ89_MONDO**  3337 **----------------------------------------------------------------------------------------------------------------------------------**

**Q8MKI9_CANFA**  3437 **LRLQRQQTQK------------------------------------------------------------------------------------------------------------------------**

**A4ZZ90 XENTR**  3219 **----------------------------------------------------------------------------------------------------------------------------------**

**3910 3920 3930 3940 3950 3960 3970 3980 3990 4000 4010 4020 4030**

**....|....|....|....|....|....|....|....|....|....|....|....|....|....|....|....|....|....|....|....|....|....|....|....|....|....|**

**P51587 HUMAN**  3418 **----------------------------------------------------------------------------------------------------------------------------------**

**Q9W157 DROME**  971 **----------------------------------------------------------------------------------------------------------------------------------**

**P97929_MOUSE**  3329 **----------------------------------------------------------------------------------------------------------------------------------**

**Q864S8 FELCA**  3372 **----------------------------------------------------------------------------------------------------------------------------------**

**O35923 RAT**  3343 **----------------------------------------------------------------------------------------------------------------------------------**

**A5A3F7_STRPU**  3820 **CETIGENVEANGAGIMENVKQVEDIPDQVSANLNNPESMKETHAAENHRNSEGSASSNQTKDATKEDNSQGSILSQQSRGNDSSQNSTPVDGSQDSSKQDKSSGPSSPKGNKRKCLSLQRKKAVPEEAGA**

**A4ZZ89_MONDO**  3337 **----------------------------------------------------------------------------------------------------------------------------------**

**Q8MKI9_CANFA**  3446 **----------------------------------------------------------------------------------------------------------------------------------**

**A4ZZ90 XENTR**  3219 **----------------------------------------------------------------------------------------------------------------------------------**

**4040 4050**

**....|....|....|....|....|....**

**P51587 HUMAN**  3418 **-----------------------------**

**Q9W157 DROME**  971 **-----------------------------**

**P97929_MOUSE**  3329 **-----------------------------**

**Q864S8 FELCA**  3372 **-----------------------------**

**O35923 RAT**  3343 **-----------------------------**

**A5A3F7_STRPU**  3950 **VIEILEDDNPPLLKRSSRLRLQQKKSYKF**

**A4ZZ89_MONDO**  3337 **-----------------------------**

**Q8MKI9_CANFA**  3446 **-----------------------------**

**A4ZZ90 XENTR**  3219 **-----------------------------**
