# Supplementary figures and images for: BRCA2 BRC missense variants disrupt RAD51-dependent DNA repair
Source: eLife. 2022 Sep 13;11:e79183. doi: 10.7554/eLife.79183 (PMC9545528; doi:10.7554/eLife.79183)

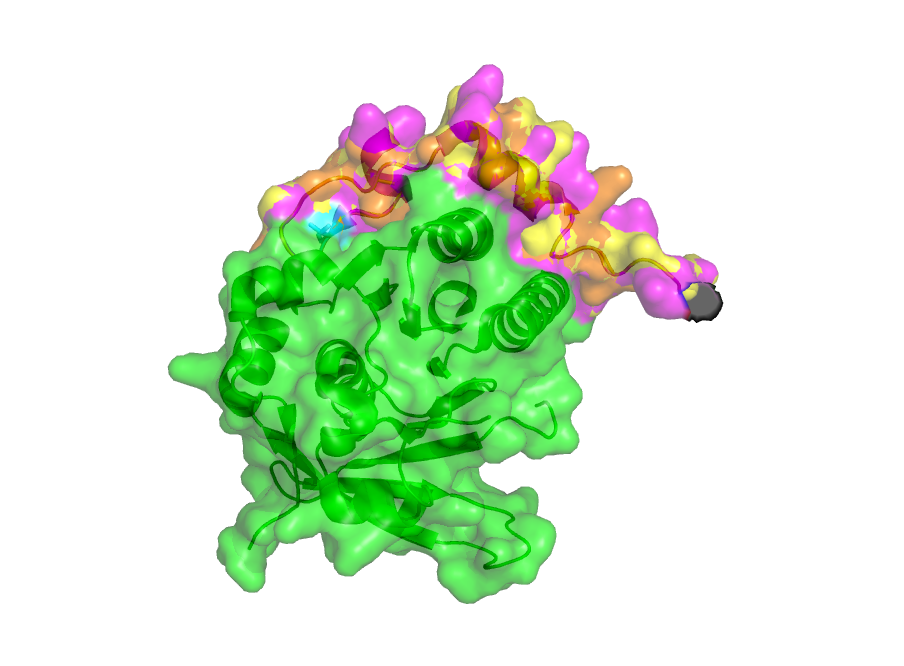

Supplement: Figure 1—source data 1. [file elife-79183-fig1-data1.zip › Figure 1-source data 1/Figure1C-source data1/Figure1C-source data4-BRC2-4-7.png]

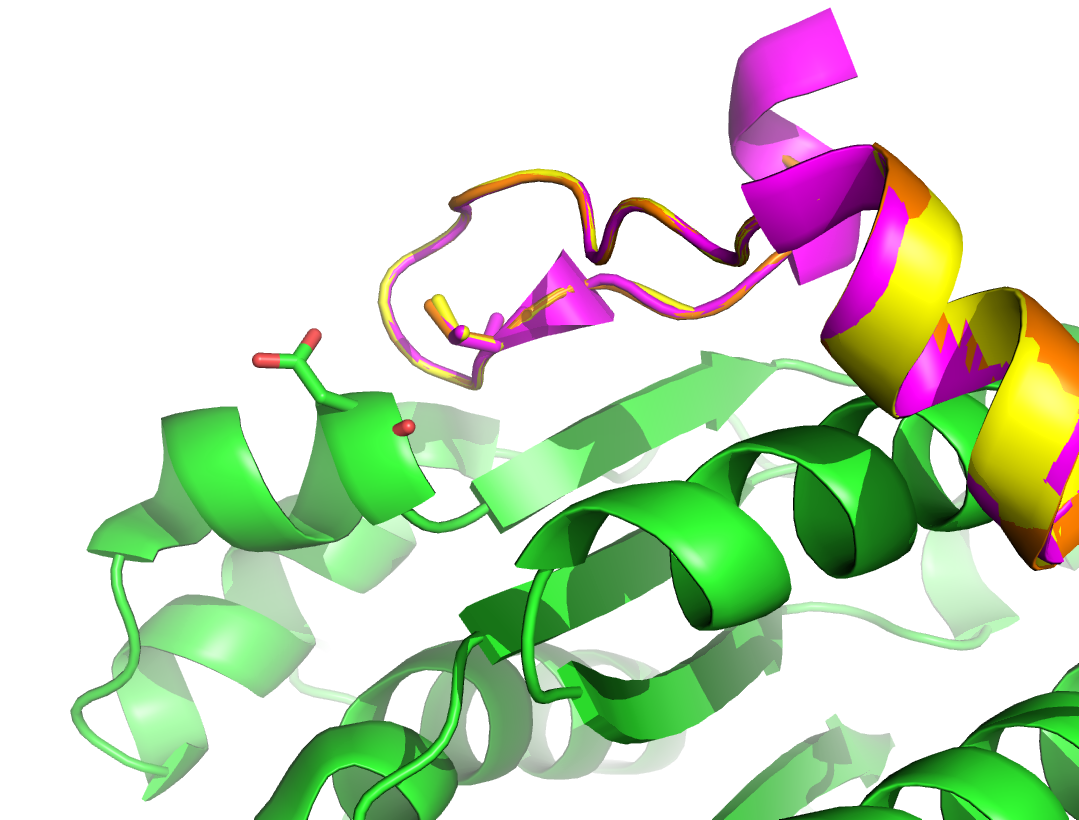

Supplement: Figure 1—source data 1. [file elife-79183-fig1-data1.zip › Figure 1-source data 1/Figure1C-source data1/Figure1C-source data5-surfaceBRC247RAD51.png]

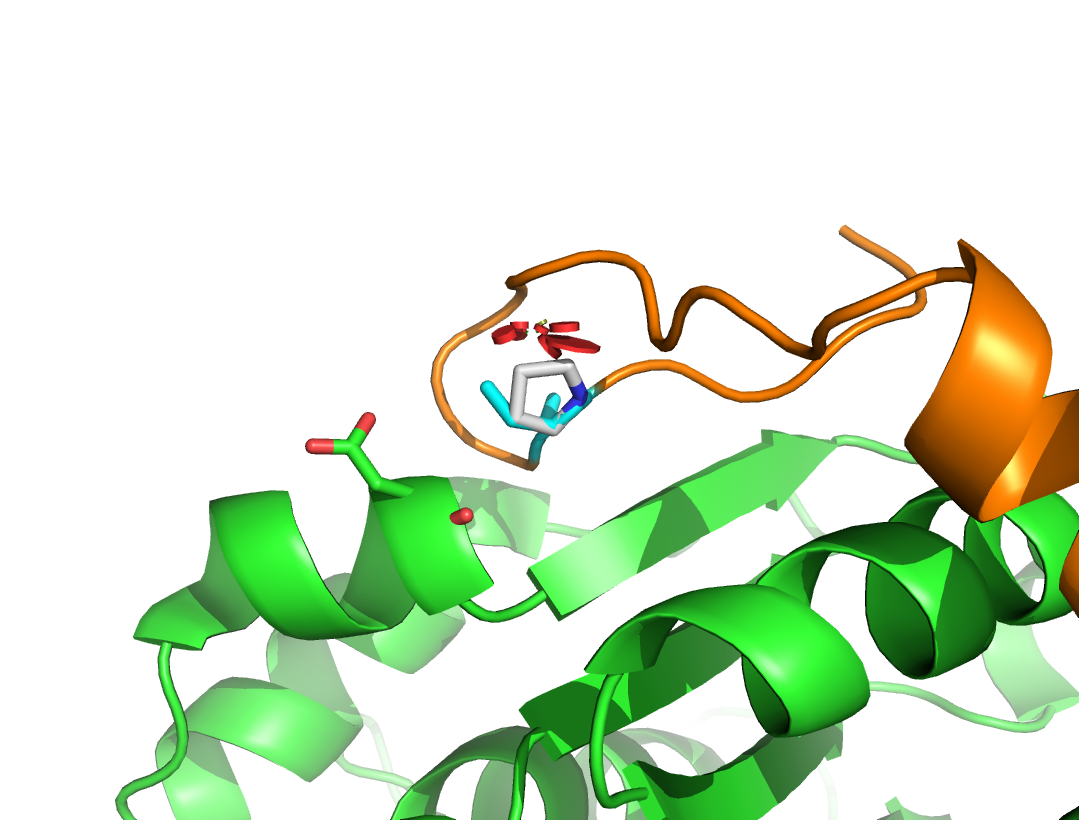

Supplement: Figure 1—source data 1. [file elife-79183-fig1-data1.zip › Figure 1-source data 1/Figure1C-source data1/Figure1C-source data6-BRC2 S1221P clashes.png]

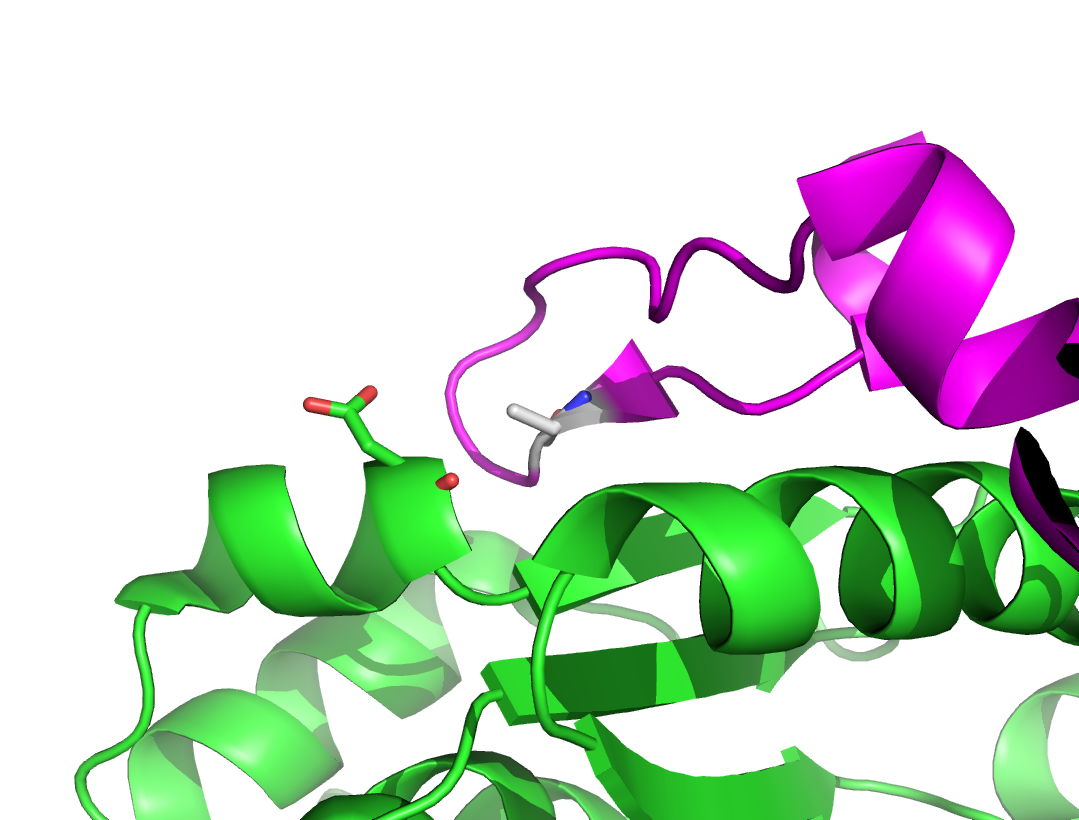

Supplement: Figure 1—source data 1. [file elife-79183-fig1-data1.zip › Figure 1-source data 1/Figure1C-source data1/Figure1C-source data7-BRC4 T1526A clashes.png]

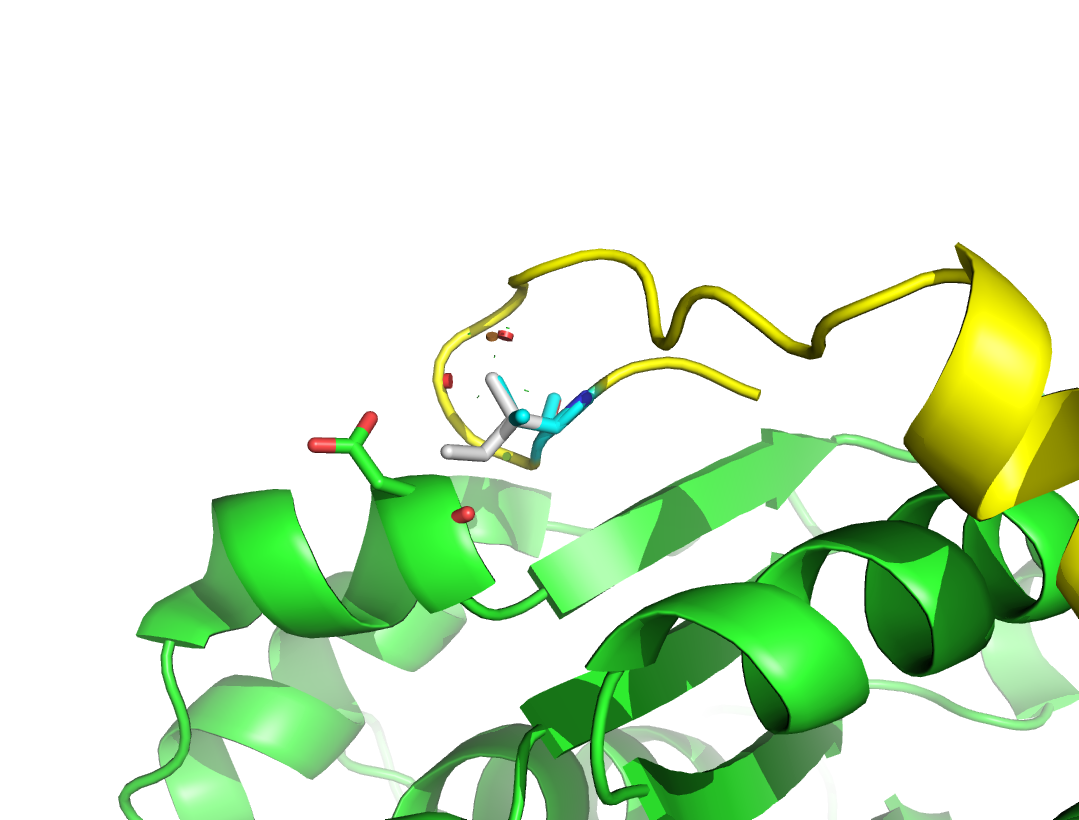

Supplement: Figure 1—source data 1. [file elife-79183-fig1-data1.zip › Figure 1-source data 1/Figure1C-source data1/Figure1C-source data8-BRC7 T1980I clashes.png]

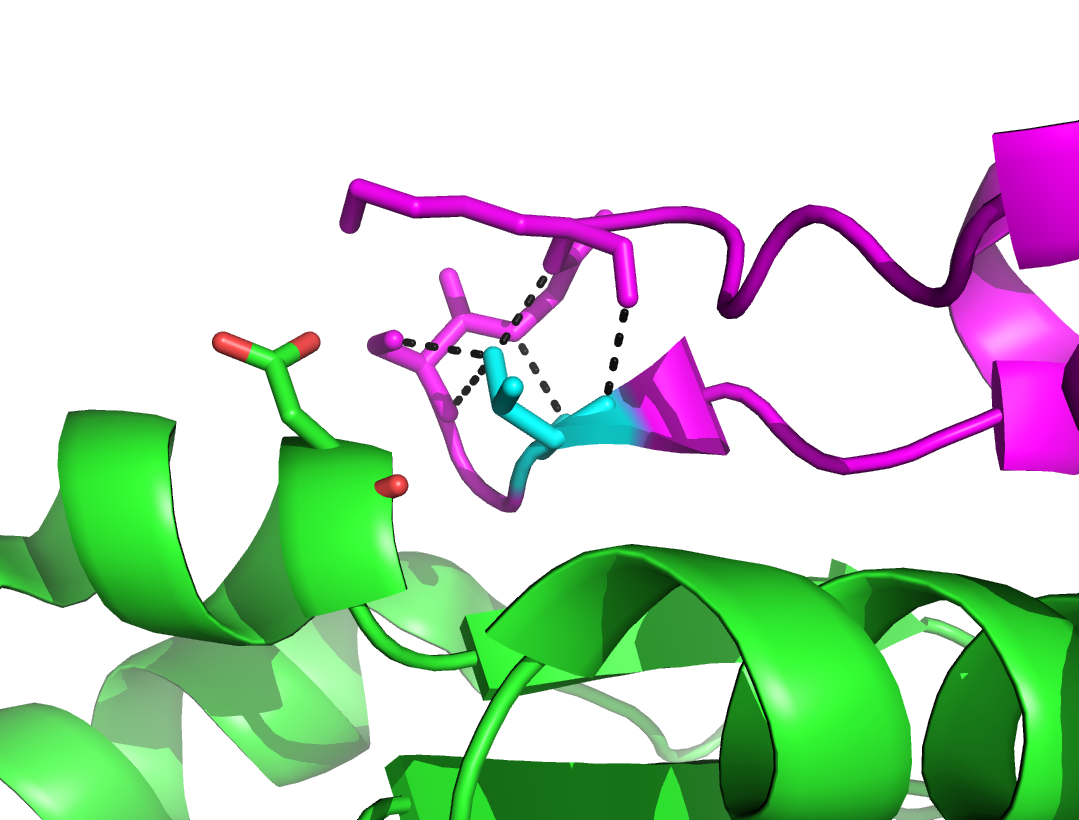

Supplement: Figure 1—figure supplement 1—source data 1. — T1526A polar contacts. T1526I polar contacts. T1526P polar contacts. [file elife-79183-fig1-figsupp1-data1.zip › Figure 1-figure supplement 1-source data1/T1526 polar contacts.png]

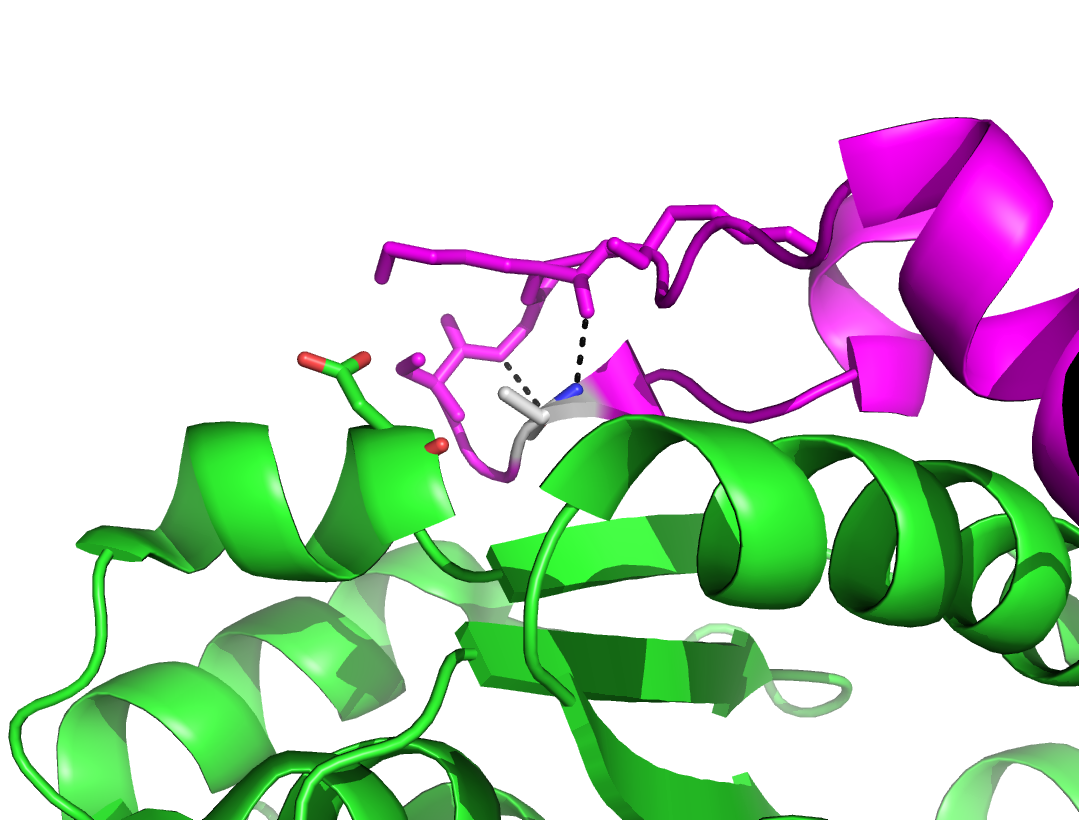

Supplement: Figure 1—figure supplement 1—source data 1. — T1526A polar contacts. T1526I polar contacts. T1526P polar contacts. [file elife-79183-fig1-figsupp1-data1.zip › Figure 1-figure supplement 1-source data1/T1526A polar contacts.png]

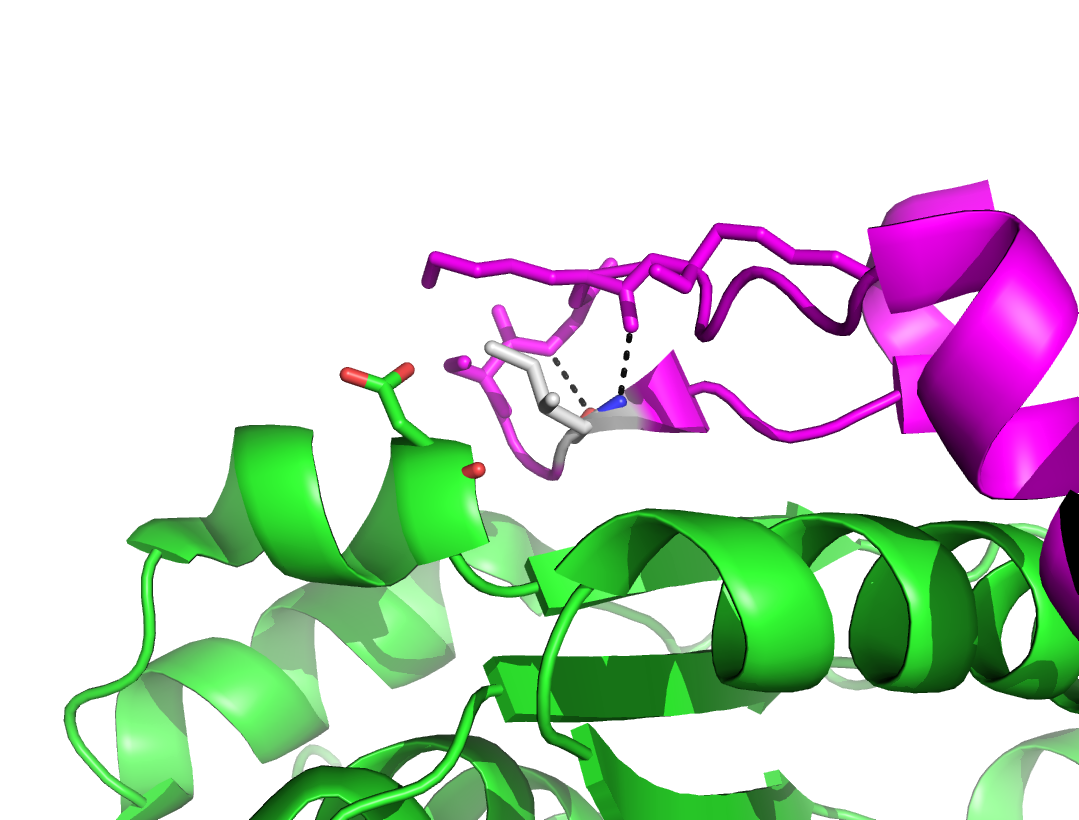

Supplement: Figure 1—figure supplement 1—source data 1. — T1526A polar contacts. T1526I polar contacts. T1526P polar contacts. [file elife-79183-fig1-figsupp1-data1.zip › Figure 1-figure supplement 1-source data1/T1526I polar contacts.png]

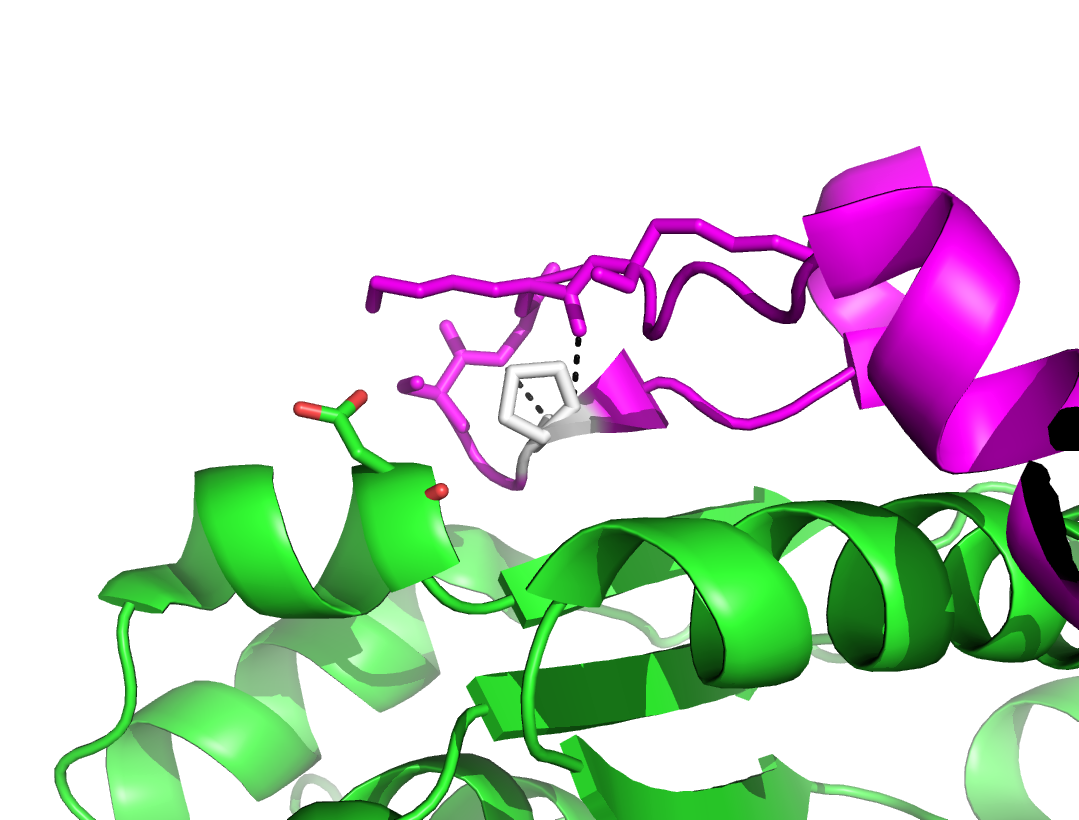

Supplement: Figure 1—figure supplement 1—source data 1. — T1526A polar contacts. T1526I polar contacts. T1526P polar contacts. [file elife-79183-fig1-figsupp1-data1.zip › Figure 1-figure supplement 1-source data1/T1526P polar contacts.png]

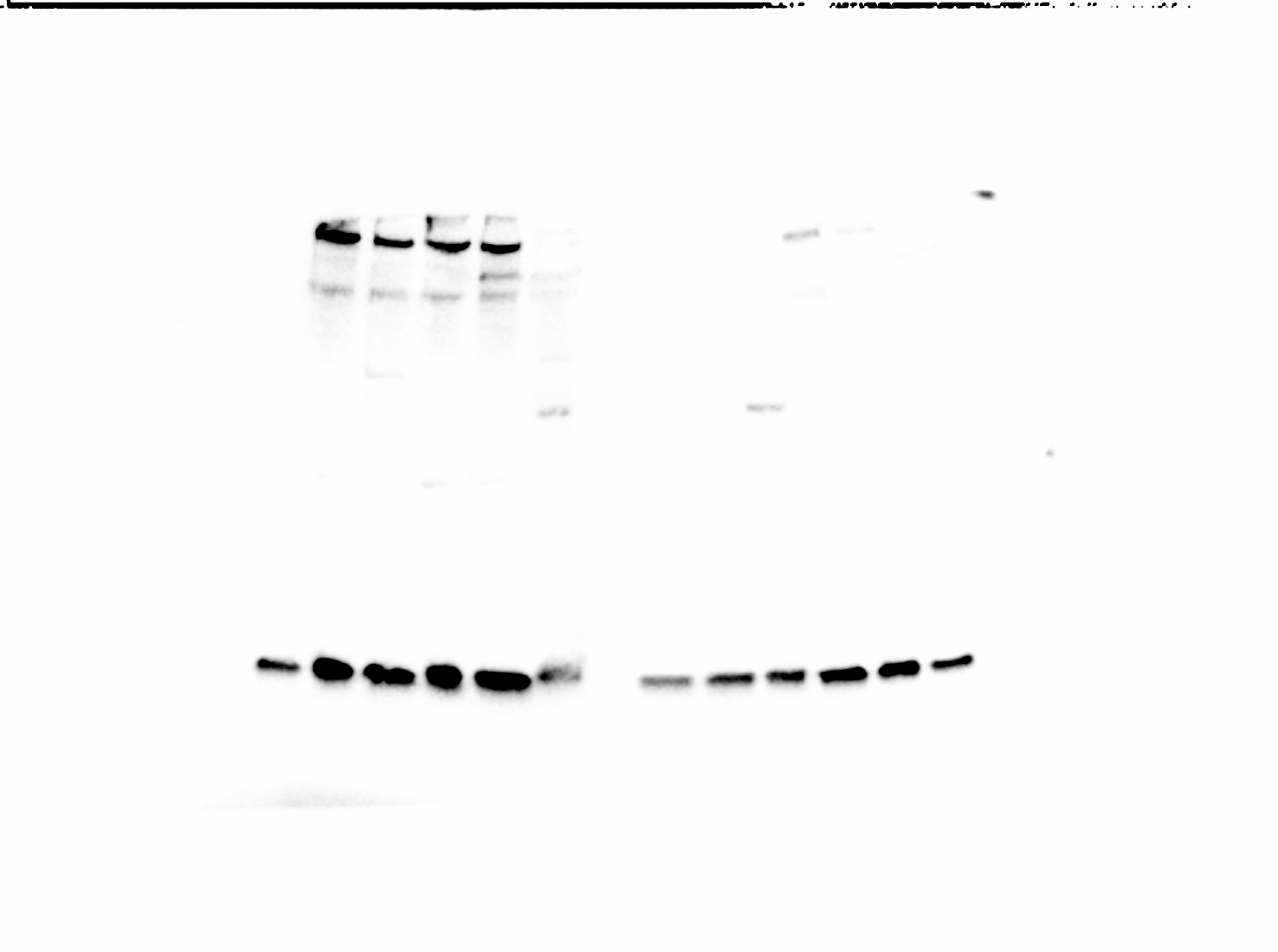

Supplement: Figure 2—source data 1. [file elife-79183-fig2-data1.zip › Figure 2-source data 1/Figure2A-source data1/Figure2A-source data1-raw BRCA2 RAD51.tif]

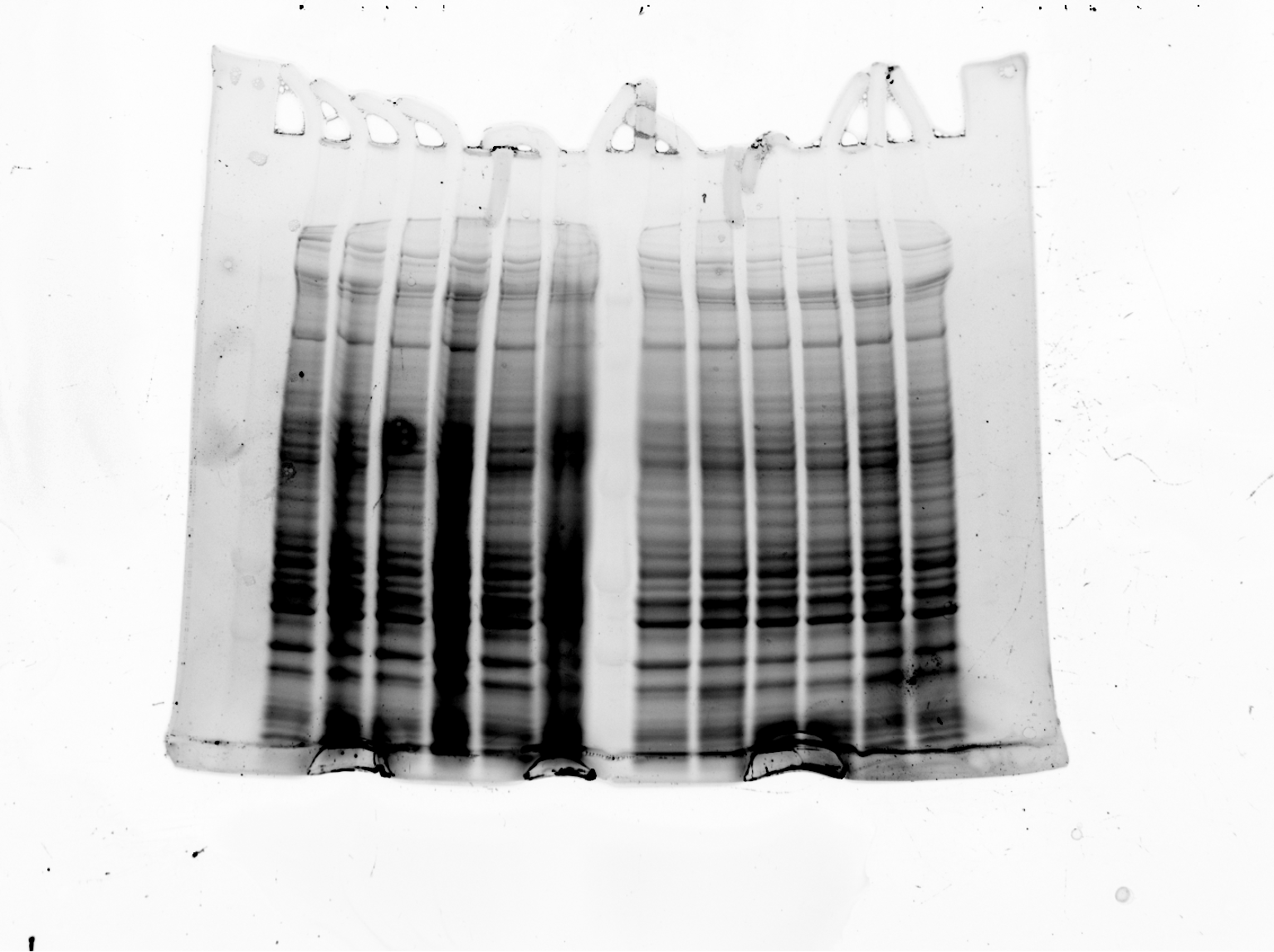

Supplement: Figure 2—source data 1. [file elife-79183-fig2-data1.zip › Figure 2-source data 1/Figure2A-source data1/Figure2A-source data2-raw StainFree.tif]

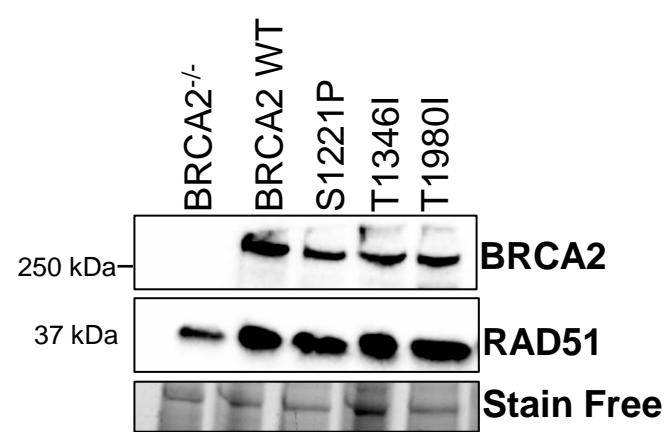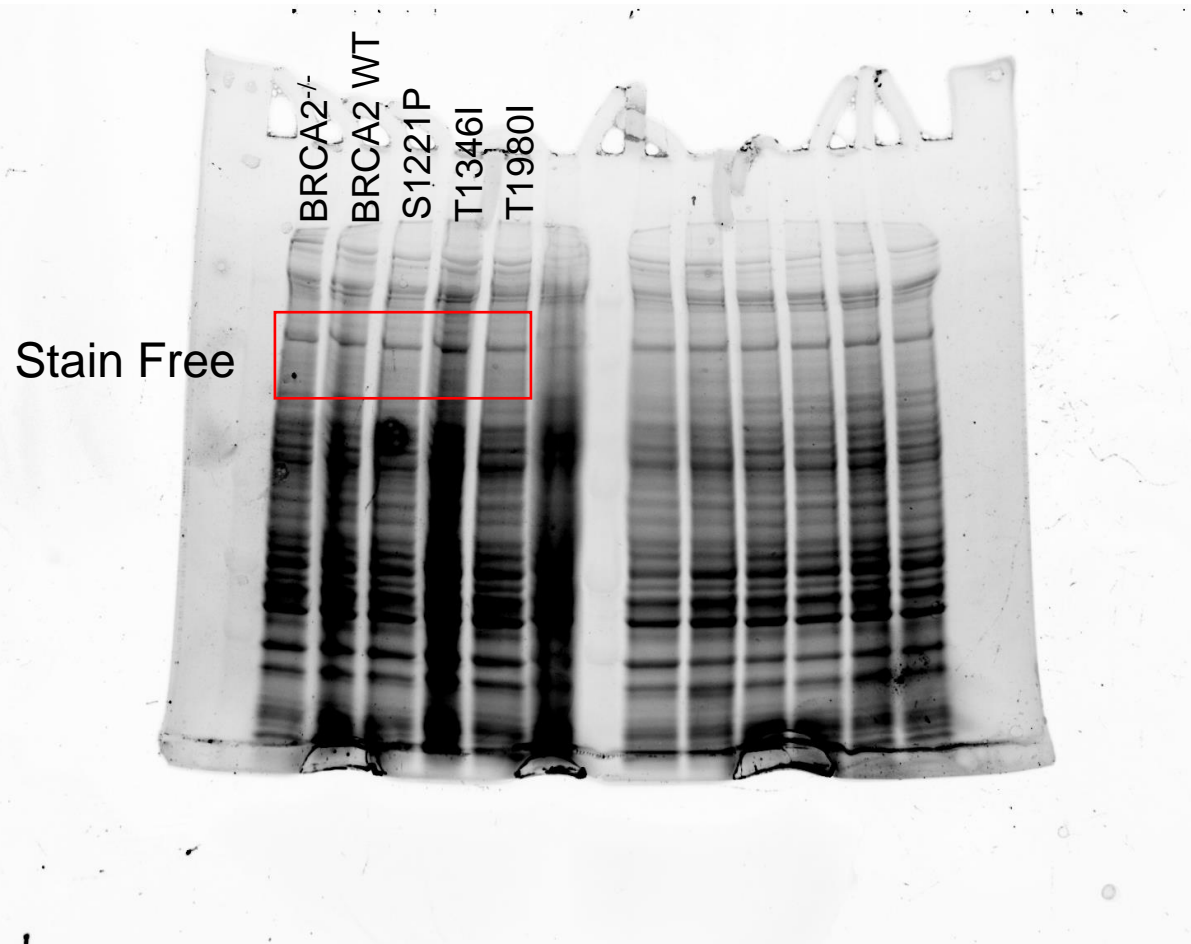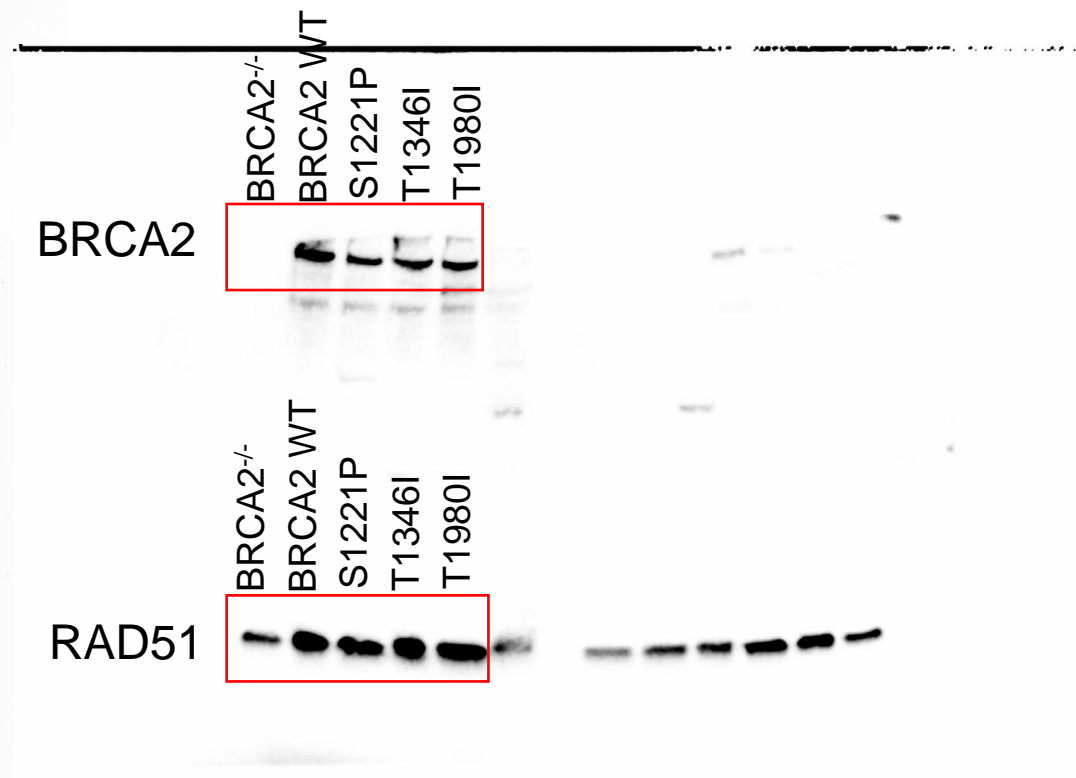

Supplement: Figure 2—source data 1. [file elife-79183-fig2-data1.zip › Figure 2-source data 1/Figure2A-source data1/Figure2A-source data3-highlightedbandsandlabels.pdf]

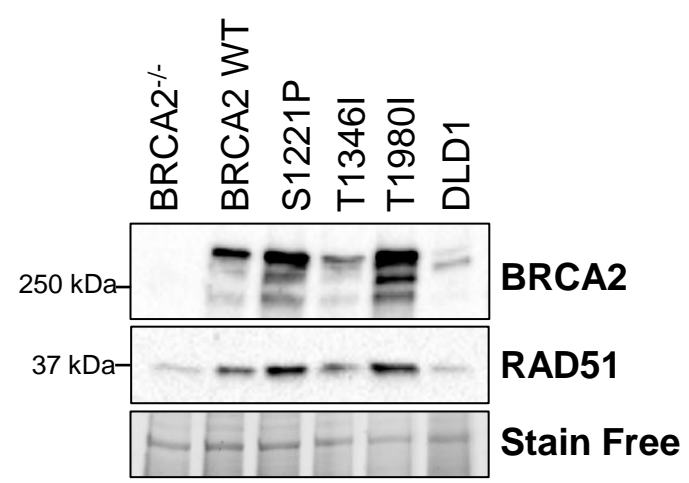

# Nt and Ct BRCA2 antibodies

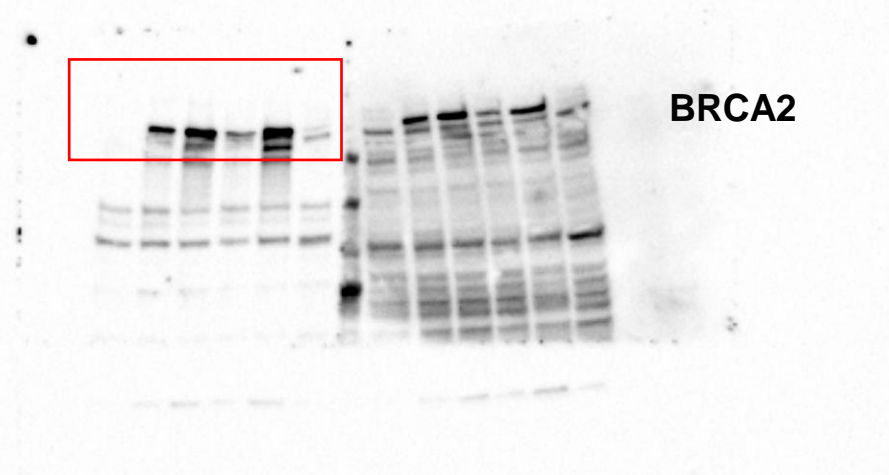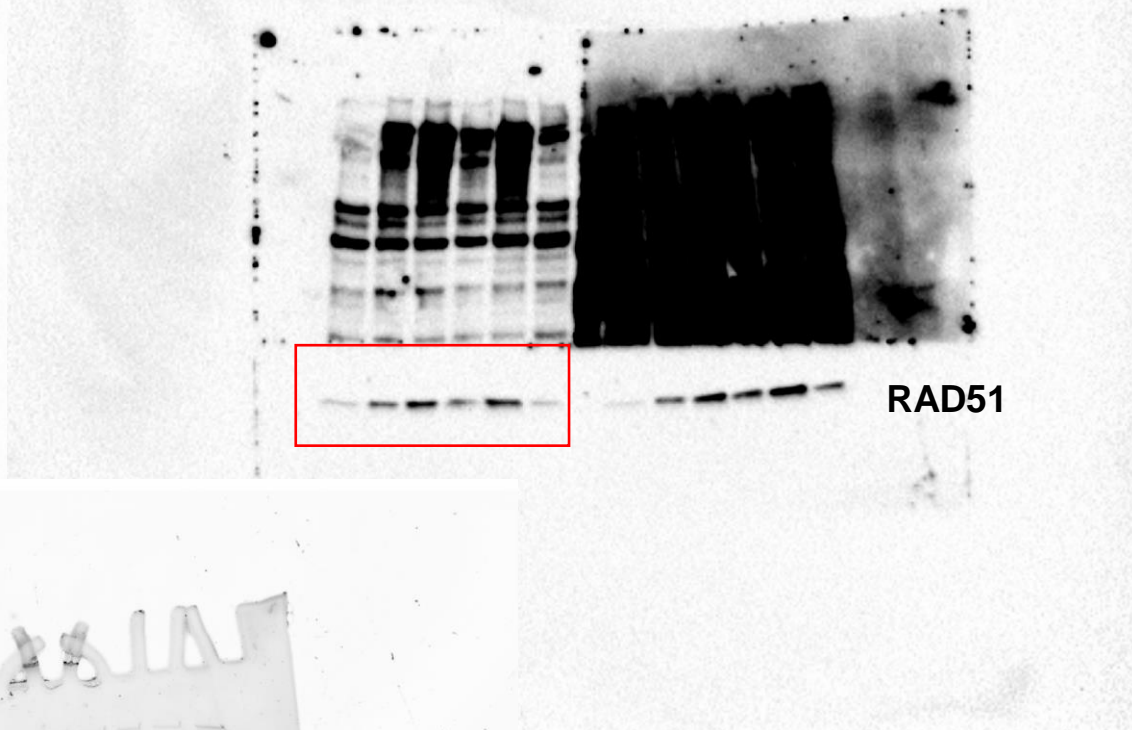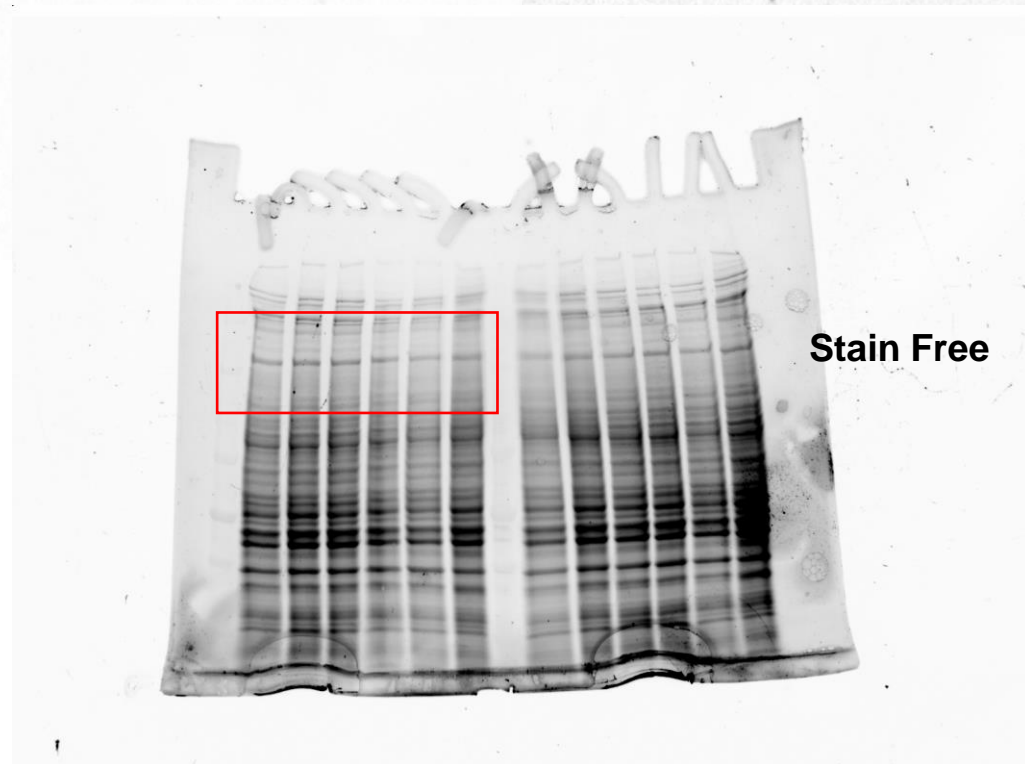

Supplement: Figure 2—figure supplement 1—source data 1. — Raw Stain Free. Nt Ct BRCA2 RAD51 highlighedbandsandlabels. [file elife-79183-fig2-figsupp1-data1.zip › Figure 2-figure supplement 1-source data1 A/Figure1-suppfig2-highlightedbandsandlabelsA.pdf]

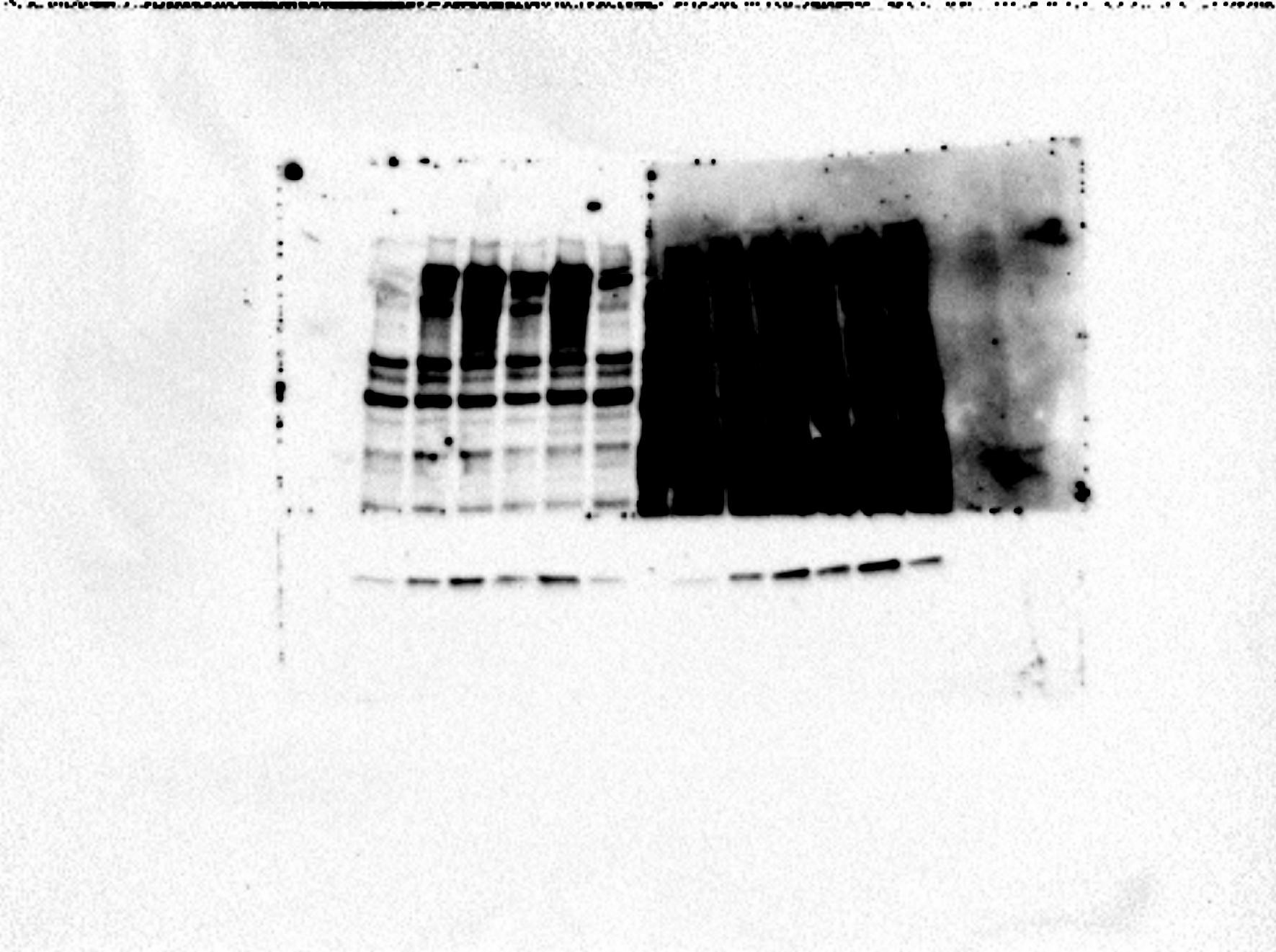

Supplement: Figure 2—figure supplement 1—source data 1. — Raw Stain Free. Nt Ct BRCA2 RAD51 highlighedbandsandlabels. [file elife-79183-fig2-figsupp1-data1.zip › Figure 2-figure supplement 1-source data1 A/Raw Nt Ct RAD51 antibodies Exposure_120.0sec.tif]

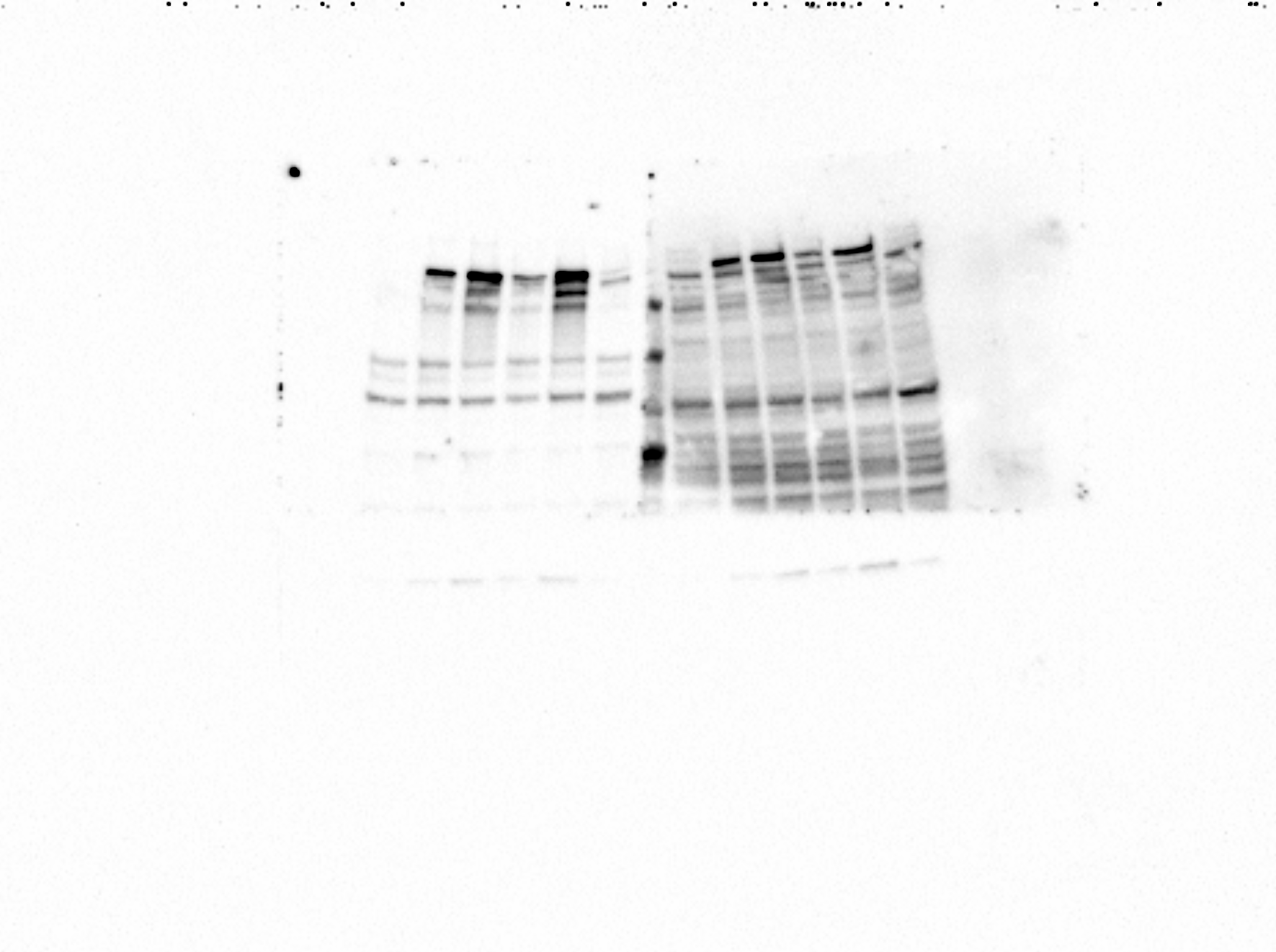

Supplement: Figure 2—figure supplement 1—source data 1. — Raw Stain Free. Nt Ct BRCA2 RAD51 highlighedbandsandlabels. [file elife-79183-fig2-figsupp1-data1.zip › Figure 2-figure supplement 1-source data1 A/Raw Nt Ct RAD51 antibodies_Exposure_15.6sec.tif]

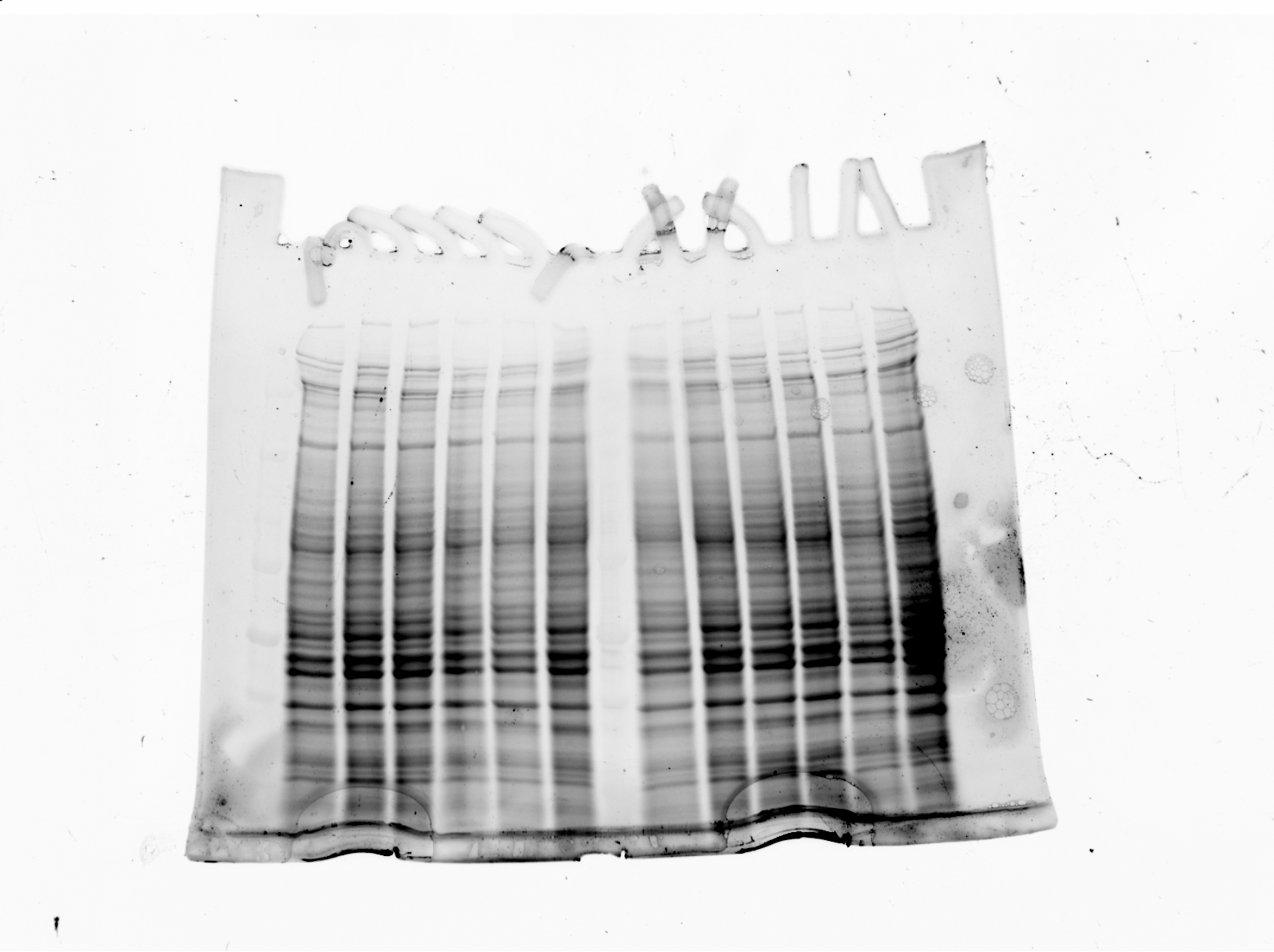

Supplement: Figure 2—figure supplement 1—source data 1. — Raw Stain Free. Nt Ct BRCA2 RAD51 highlighedbandsandlabels. [file elife-79183-fig2-figsupp1-data1.zip › Figure 2-figure supplement 1-source data1 A/StainFree Nt Ct RAD51 antibodies.tif]

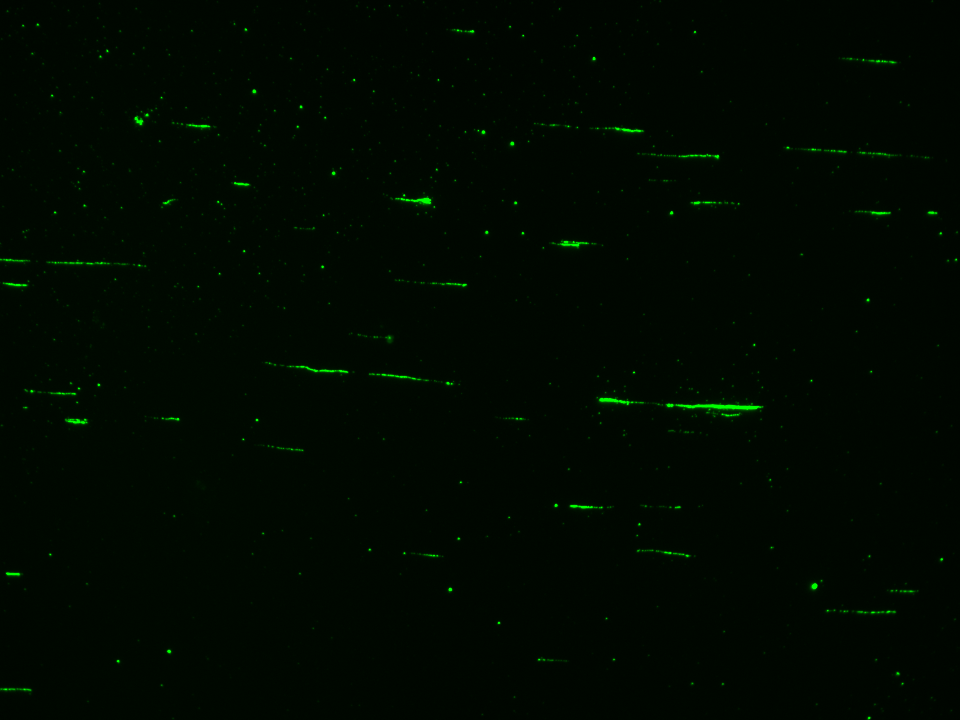

Supplement: Figure 3—source data 1. [file elife-79183-fig3-data1.zip › Figure 3-source data 1/Figure3E-source data1- original images DNA fibers/2021-03-19 BRCA2--_09/CH3.tif]

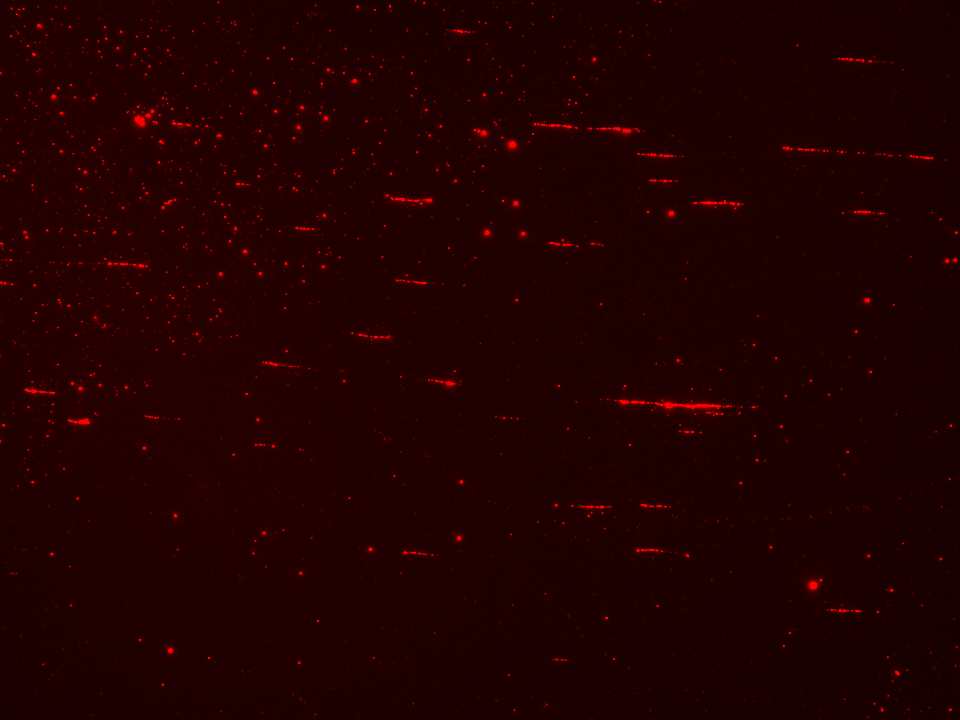

Supplement: Figure 3—source data 1. [file elife-79183-fig3-data1.zip › Figure 3-source data 1/Figure3E-source data1- original images DNA fibers/2021-03-19 BRCA2--_09/CH4.tif]

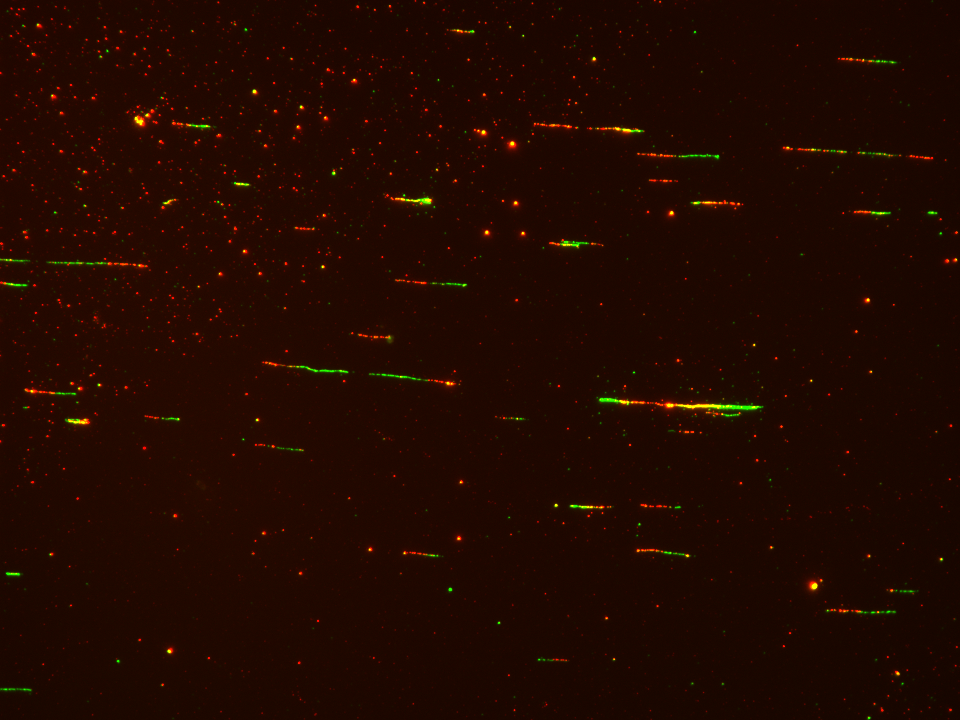

Supplement: Figure 3—source data 1. [file elife-79183-fig3-data1.zip › Figure 3-source data 1/Figure3E-source data1- original images DNA fibers/2021-03-19 BRCA2--_09/Over.tif]

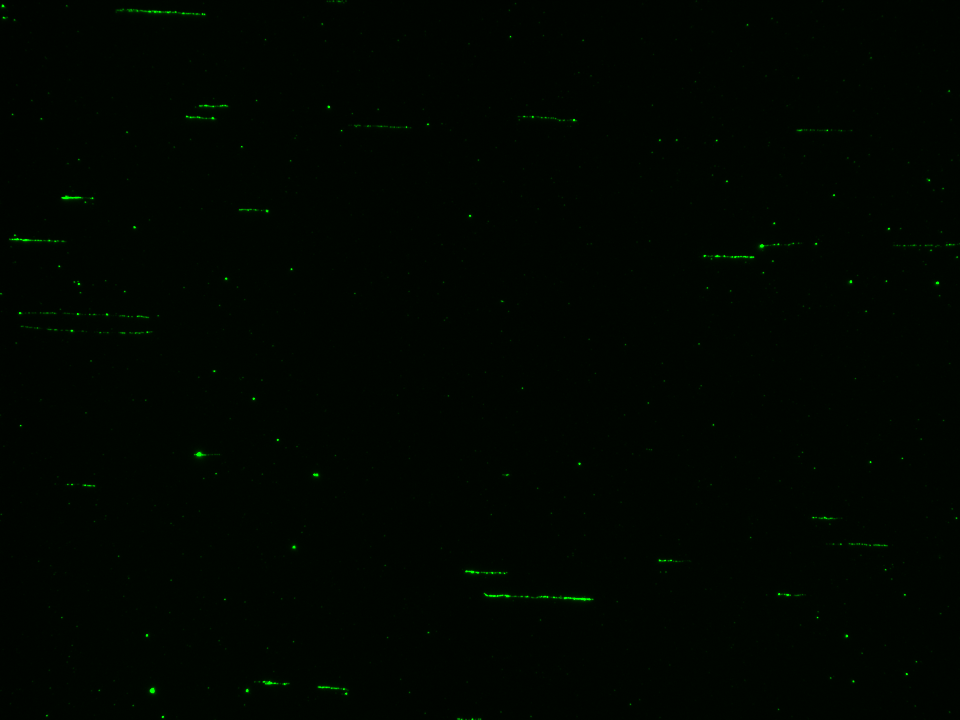

Supplement: Figure 3—source data 1. [file elife-79183-fig3-data1.zip › Figure 3-source data 1/Figure3E-source data1- original images DNA fibers/2021-03-19 S1221P_24/CH3.tif]

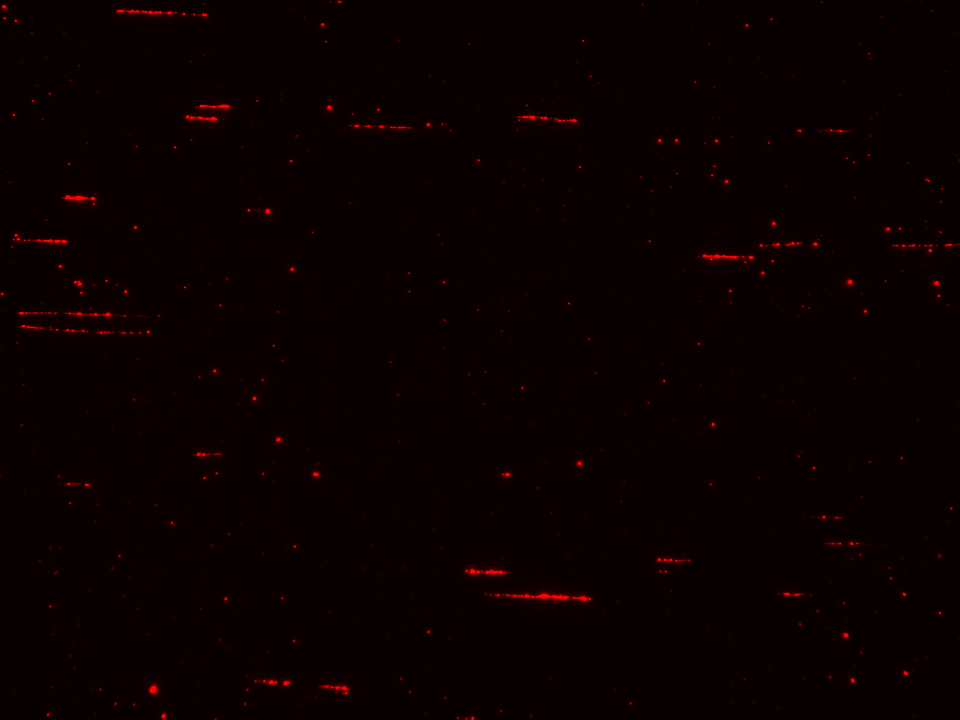

Supplement: Figure 3—source data 1. [file elife-79183-fig3-data1.zip › Figure 3-source data 1/Figure3E-source data1- original images DNA fibers/2021-03-19 S1221P_24/CH4.tif]

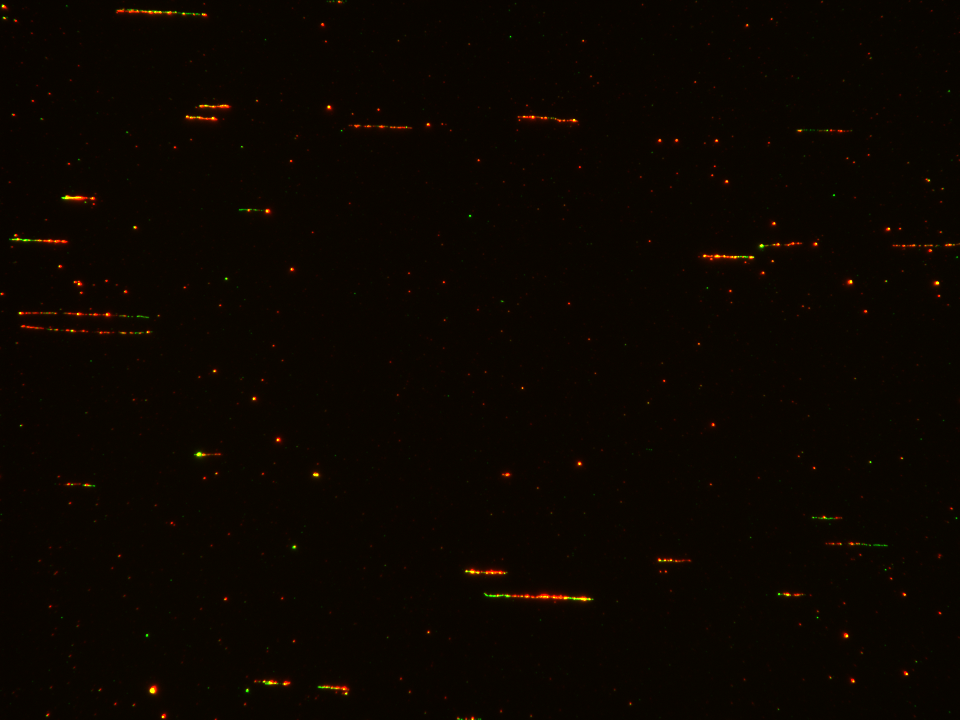

Supplement: Figure 3—source data 1. [file elife-79183-fig3-data1.zip › Figure 3-source data 1/Figure3E-source data1- original images DNA fibers/2021-03-19 S1221P_24/Overlay.tif]

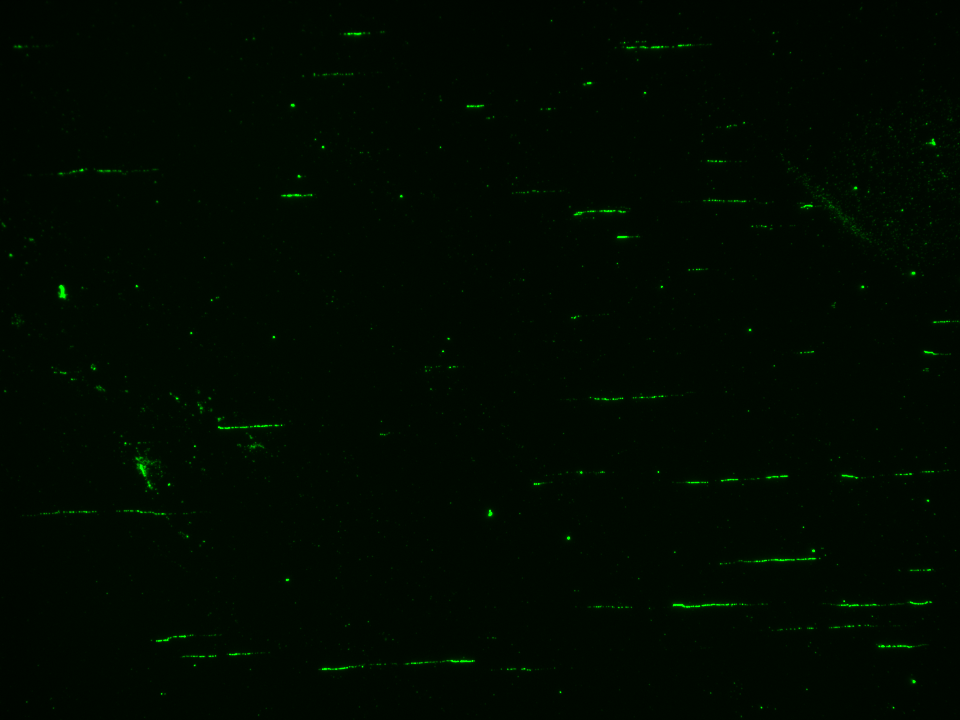

Supplement: Figure 3—source data 1. [file elife-79183-fig3-data1.zip › Figure 3-source data 1/Figure3E-source data1- original images DNA fibers/2021-05-28 T1346I_08/CH3.tif]

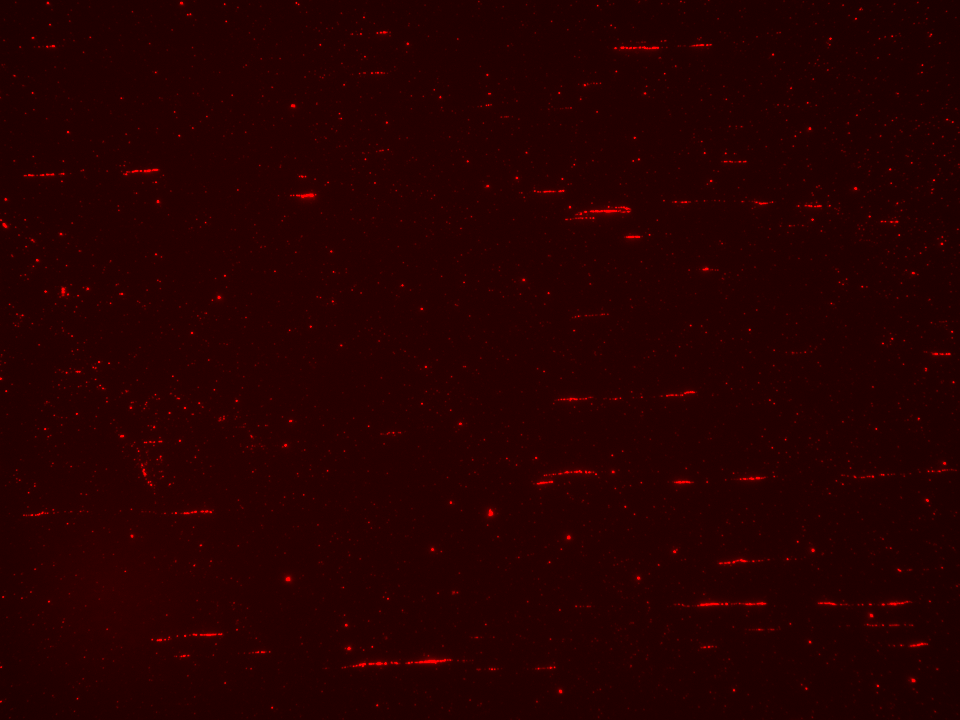

Supplement: Figure 3—source data 1. [file elife-79183-fig3-data1.zip › Figure 3-source data 1/Figure3E-source data1- original images DNA fibers/2021-05-28 T1346I_08/CH4.tif]

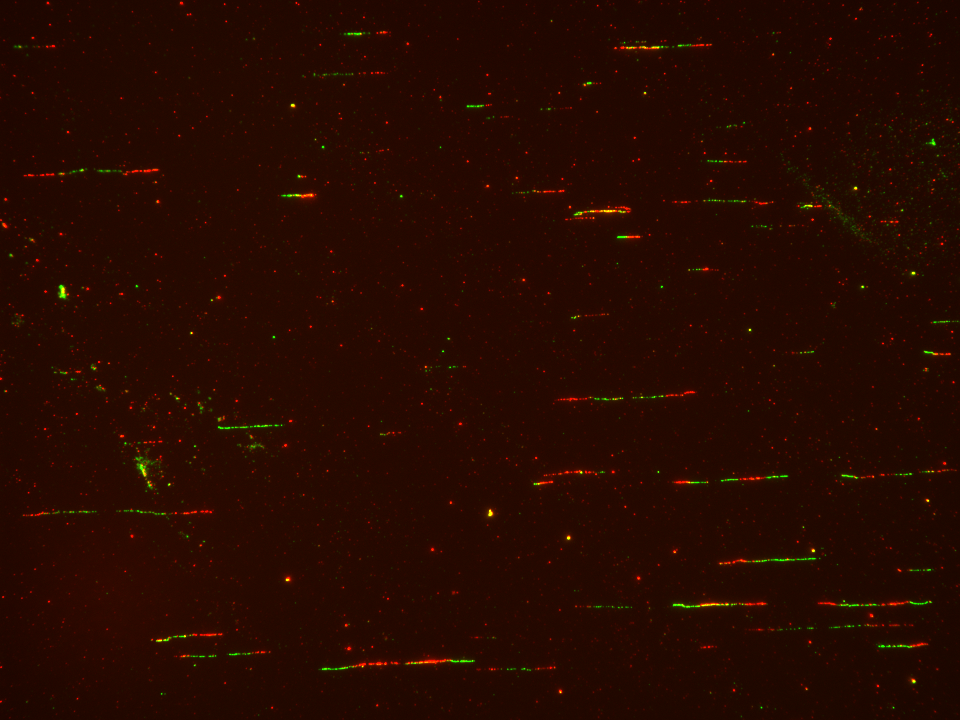

Supplement: Figure 3—source data 1. [file elife-79183-fig3-data1.zip › Figure 3-source data 1/Figure3E-source data1- original images DNA fibers/2021-05-28 T1346I_08/Over.tif]

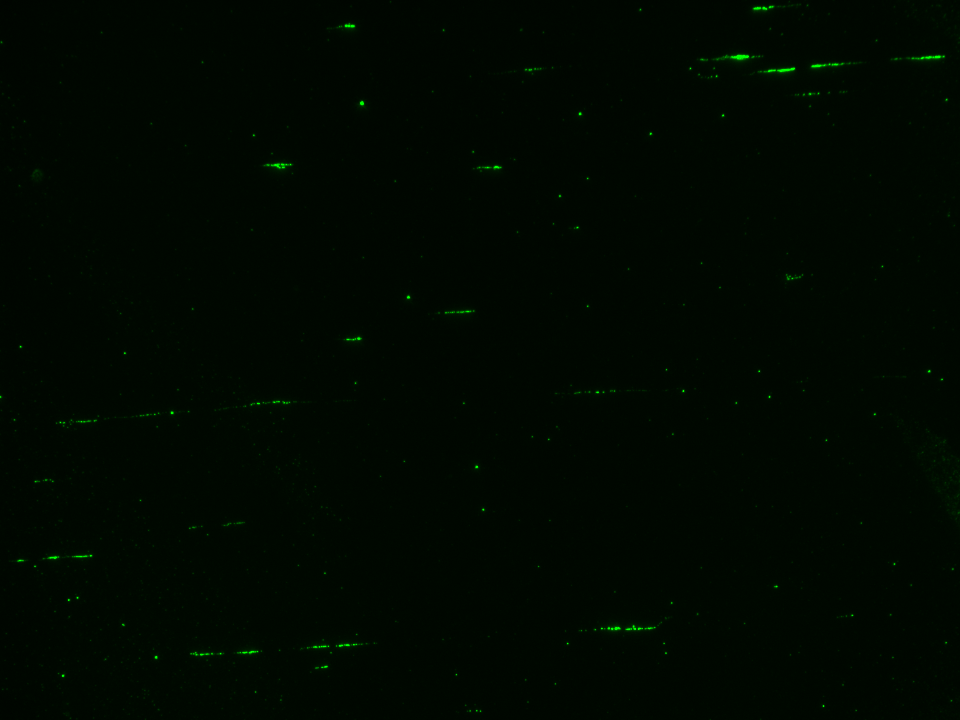

Supplement: Figure 3—source data 1. [file elife-79183-fig3-data1.zip › Figure 3-source data 1/Figure3E-source data1- original images DNA fibers/2021-06-07 BRCA2WT_31/CH3.tif]

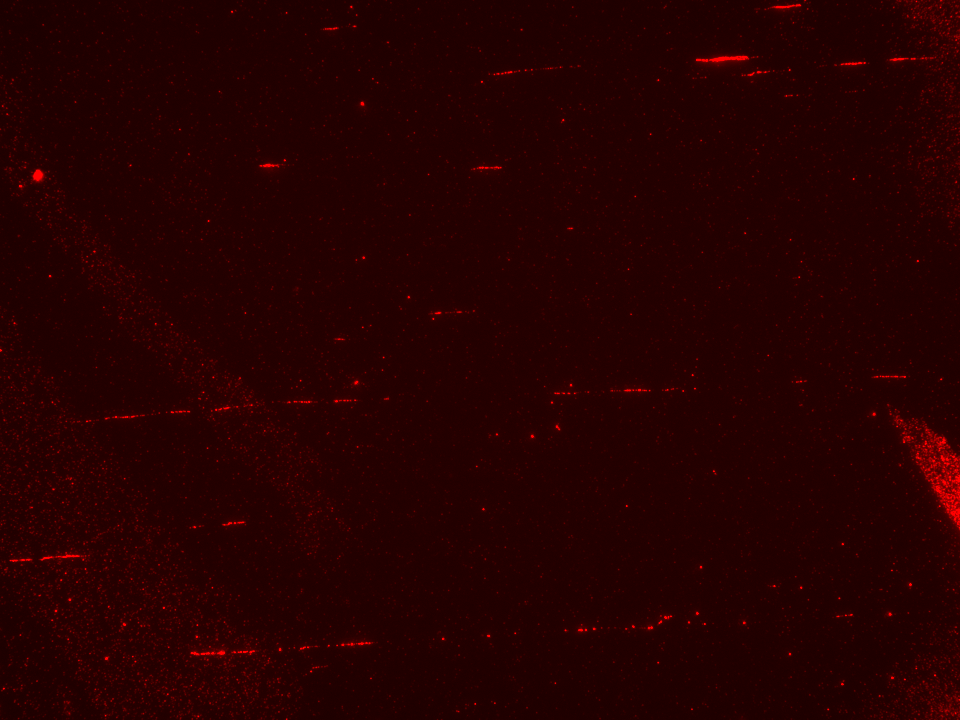

Supplement: Figure 3—source data 1. [file elife-79183-fig3-data1.zip › Figure 3-source data 1/Figure3E-source data1- original images DNA fibers/2021-06-07 BRCA2WT_31/CH4.tif]

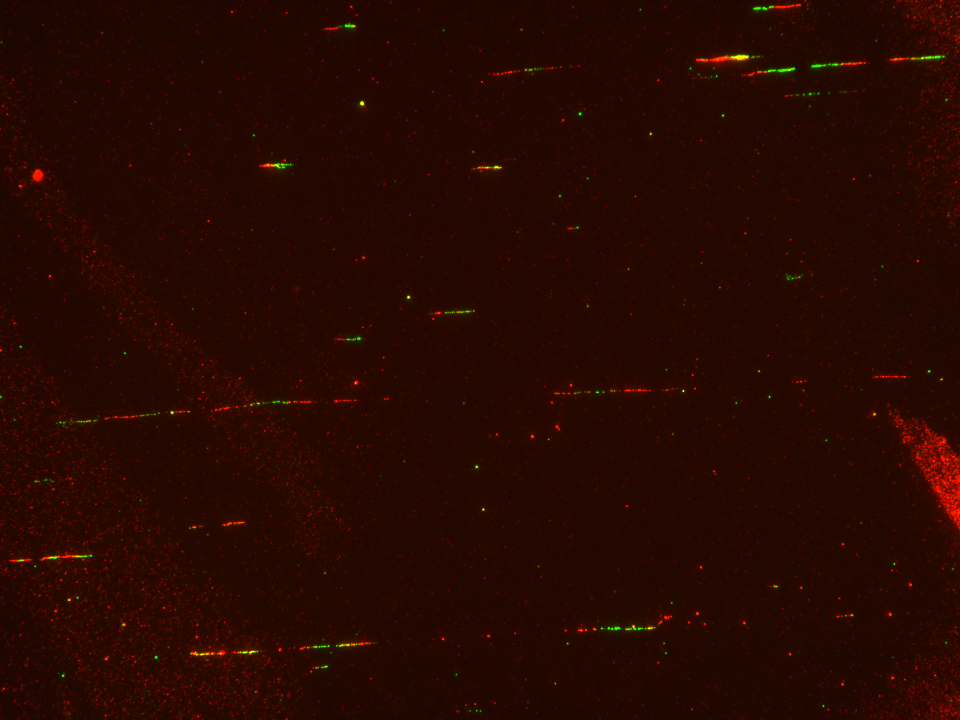

Supplement: Figure 3—source data 1. [file elife-79183-fig3-data1.zip › Figure 3-source data 1/Figure3E-source data1- original images DNA fibers/2021-06-07 BRCA2WT_31/Ov.tif]

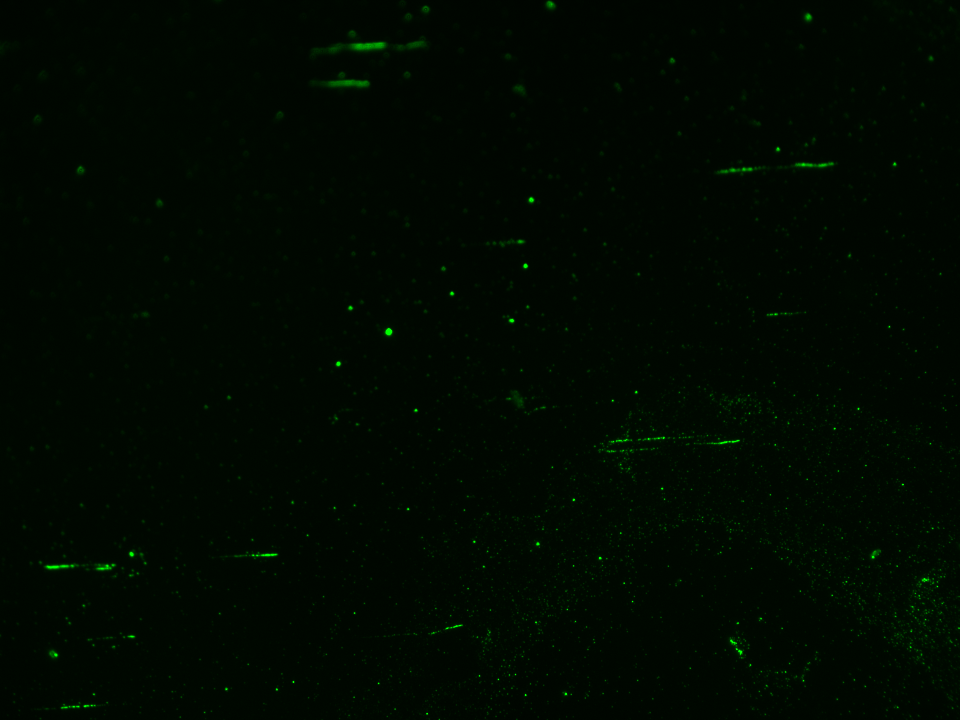

Supplement: Figure 3—source data 1. [file elife-79183-fig3-data1.zip › Figure 3-source data 1/Figure3E-source data1- original images DNA fibers/2021-06-07 T1980I_04/CH3.tif]

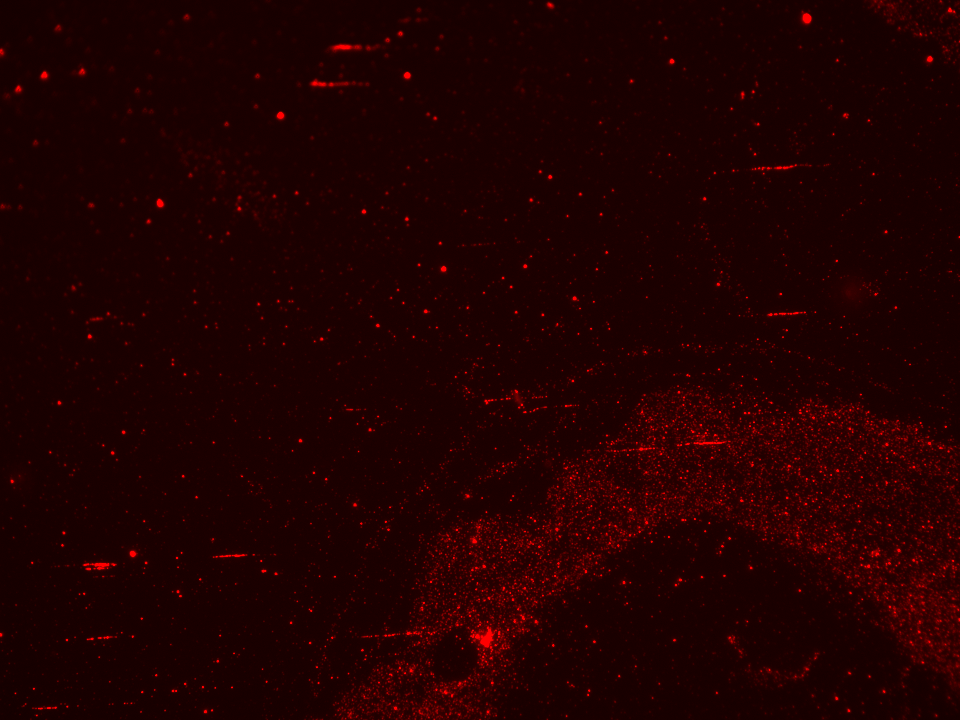

Supplement: Figure 3—source data 1. [file elife-79183-fig3-data1.zip › Figure 3-source data 1/Figure3E-source data1- original images DNA fibers/2021-06-07 T1980I_04/CH4.tif]

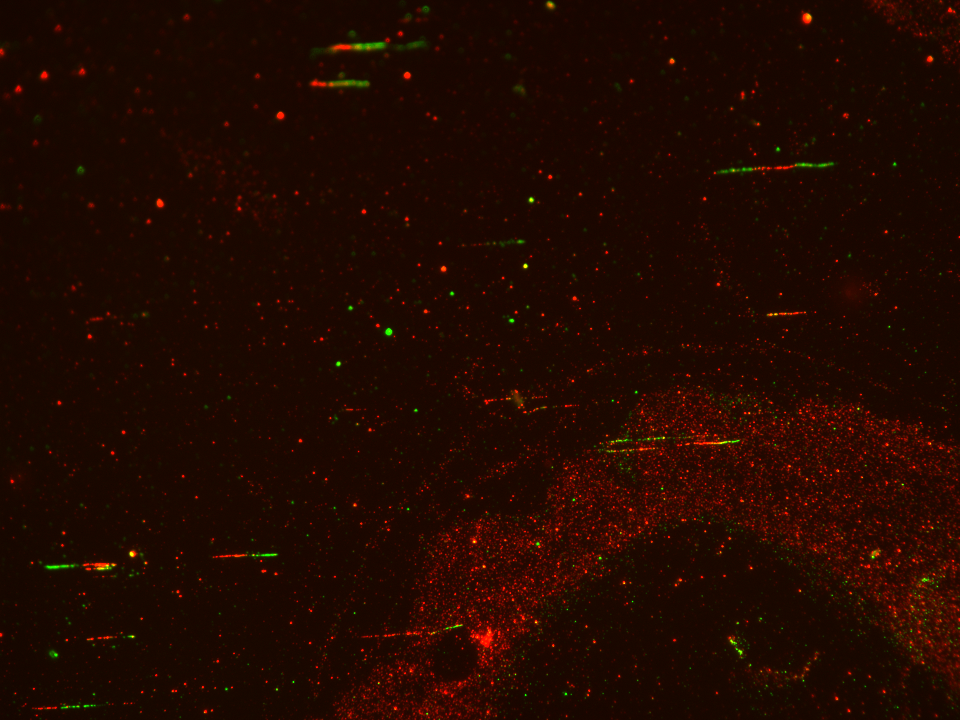

Supplement: Figure 3—source data 1. [file elife-79183-fig3-data1.zip › Figure 3-source data 1/Figure3E-source data1- original images DNA fibers/2021-06-07 T1980I_04/Over.tif]

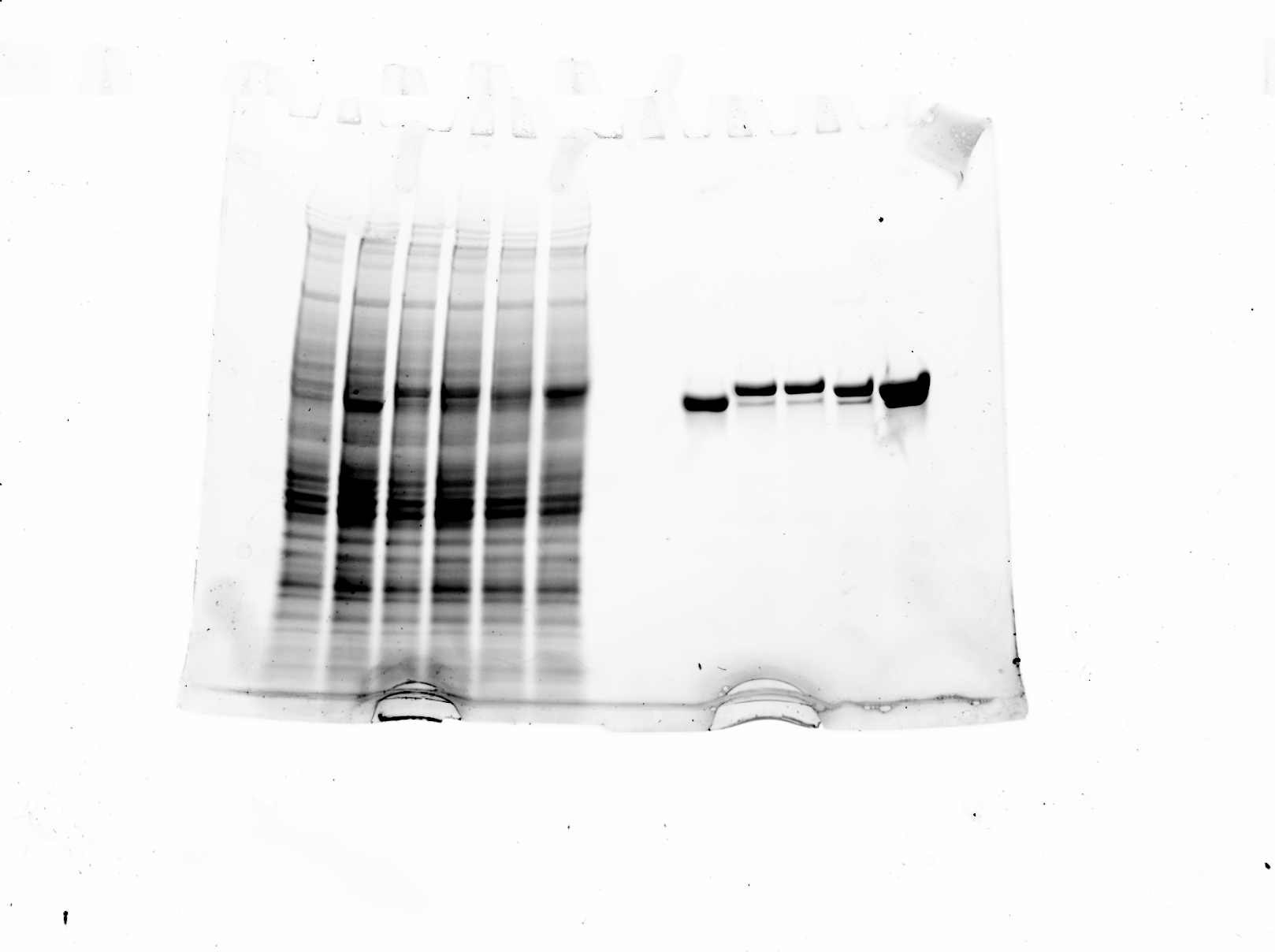

Supplement: Figure 4—source data 1. [file elife-79183-fig4-data1.zip › Figure 4-source data 1/Figure4B-source data1/Figure4B-source data 1-panel StainFree-panel MBP.tif]

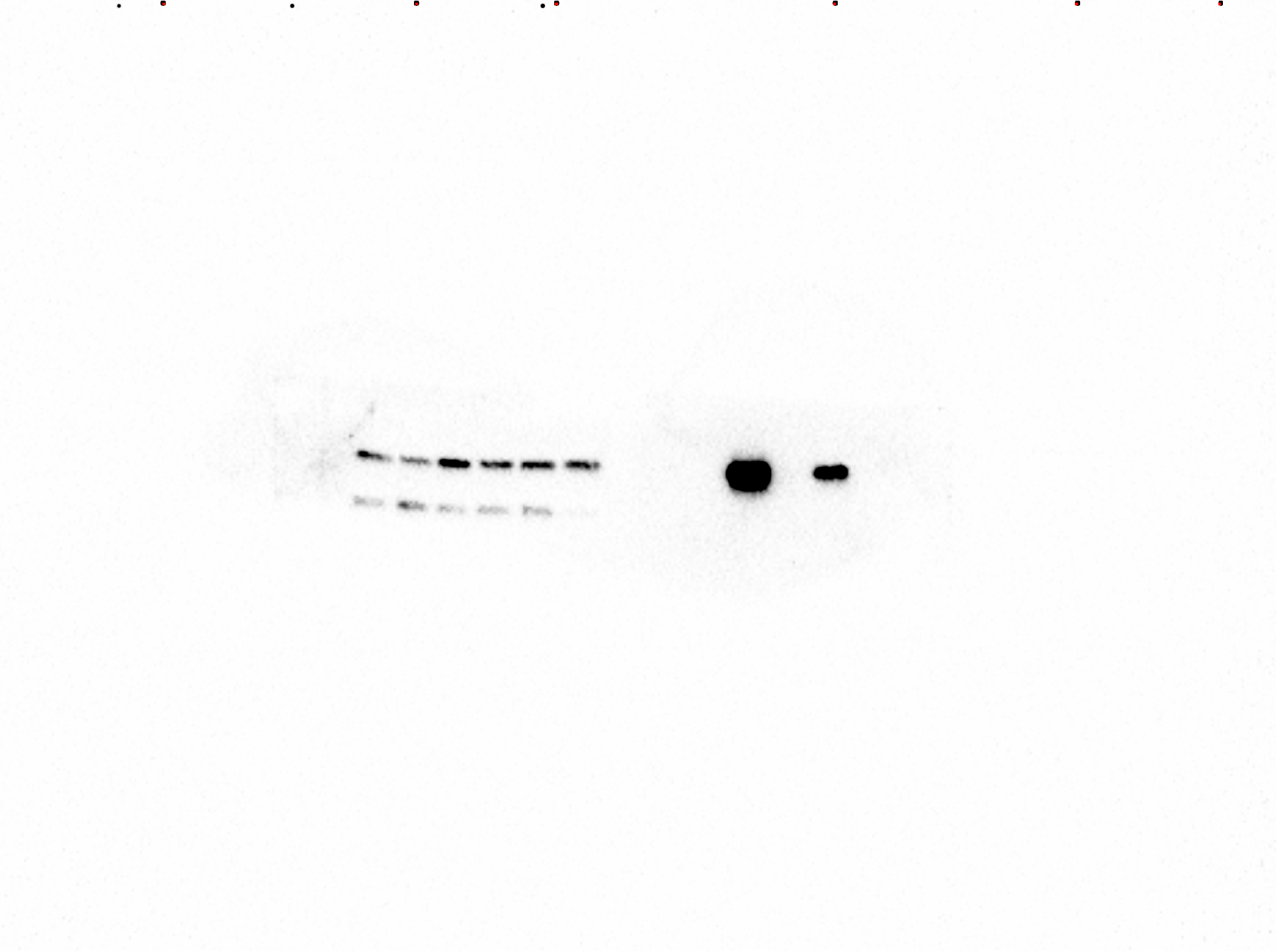

Supplement: Figure 4—source data 1. [file elife-79183-fig4-data1.zip › Figure 4-source data 1/Figure4B-source data1/Figure4B-source data 2-panel RAD51_Exposure_2.0sec.tif]

**B**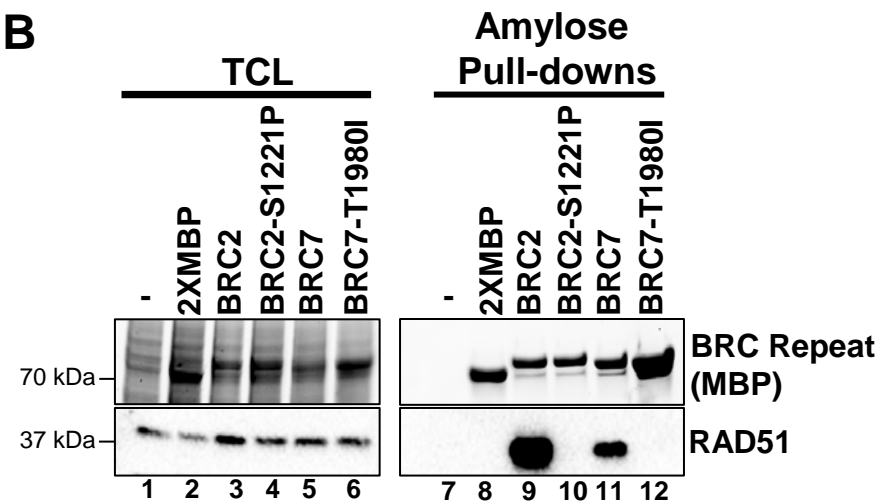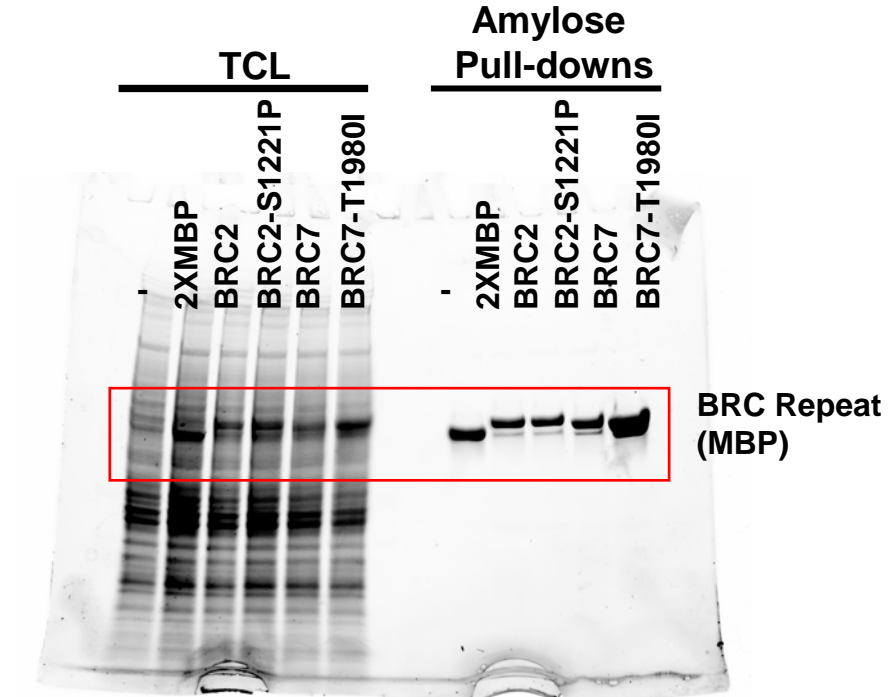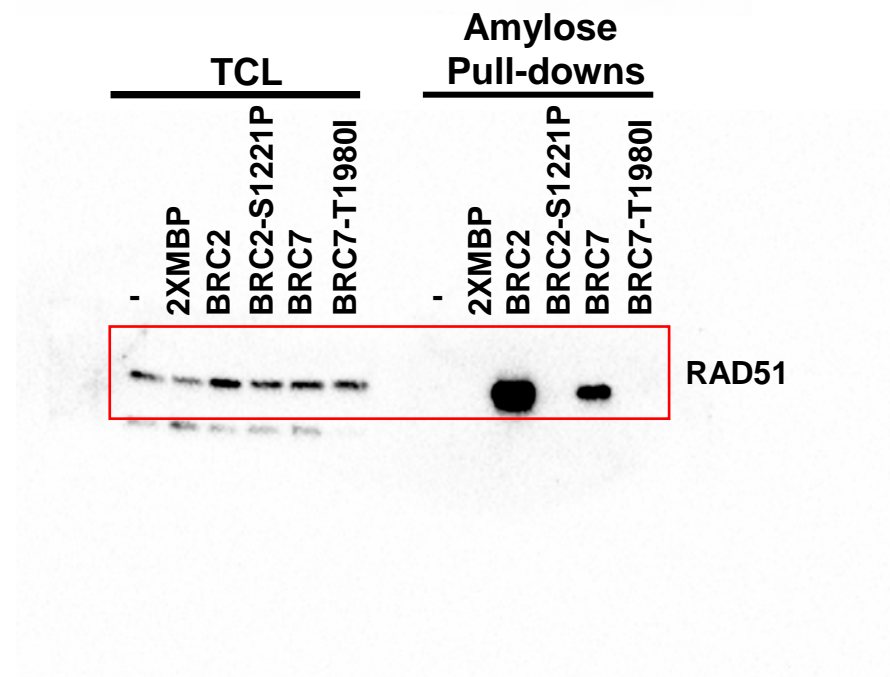

Supplement: Figure 4—source data 1. [file elife-79183-fig4-data1.zip › Figure 4-source data 1/Figure4B-source data1/Figure4B-source data 3-highlightedbandsandlabeled.pdf]

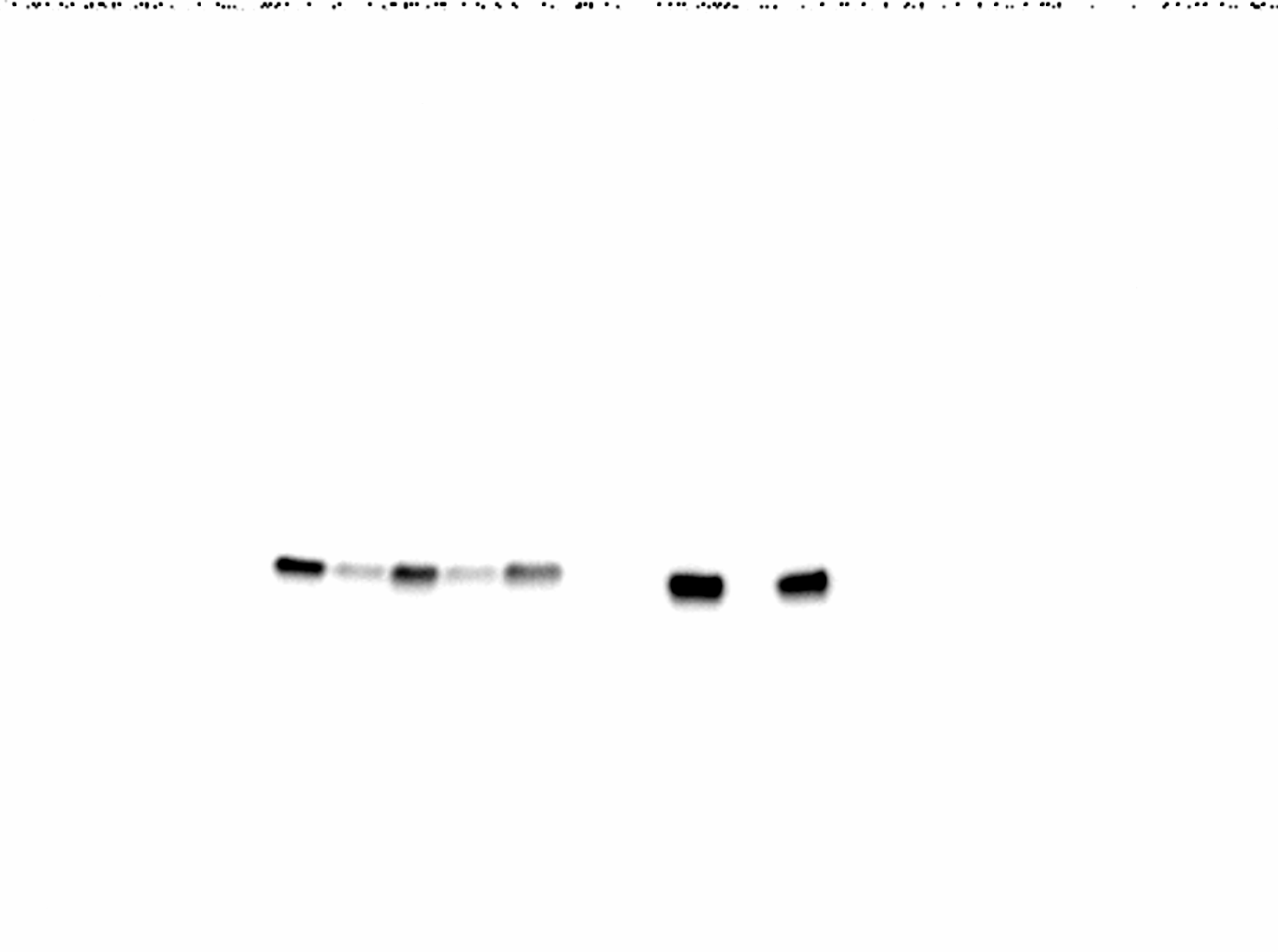

Supplement: Figure 4—source data 1. [file elife-79183-fig4-data1.zip › Figure 4-source data 1/Figure4D-source data1/Figure4D-source data1-RAD51 panel raw_7.0sec.tif]

**D****Amino-linked synthetic peptides**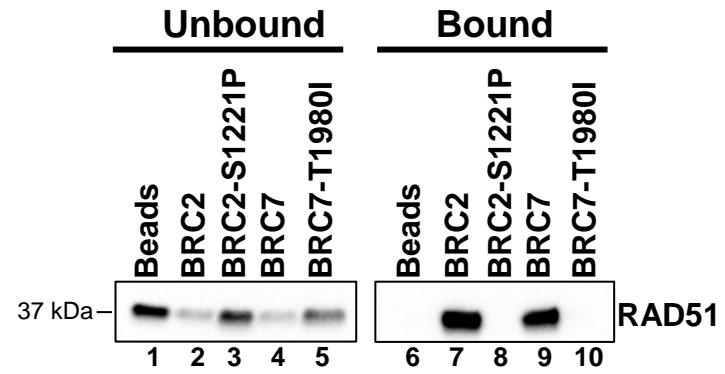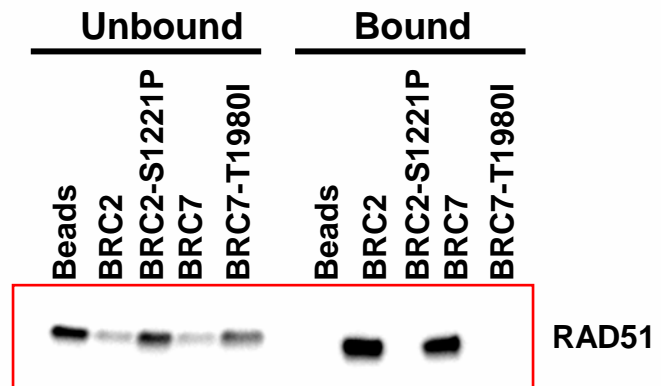

Supplement: Figure 4—source data 1. [file elife-79183-fig4-data1.zip › Figure 4-source data 1/Figure4D-source data1/Figure4D-source data2-highlightedbandsandlabeled.pdf]

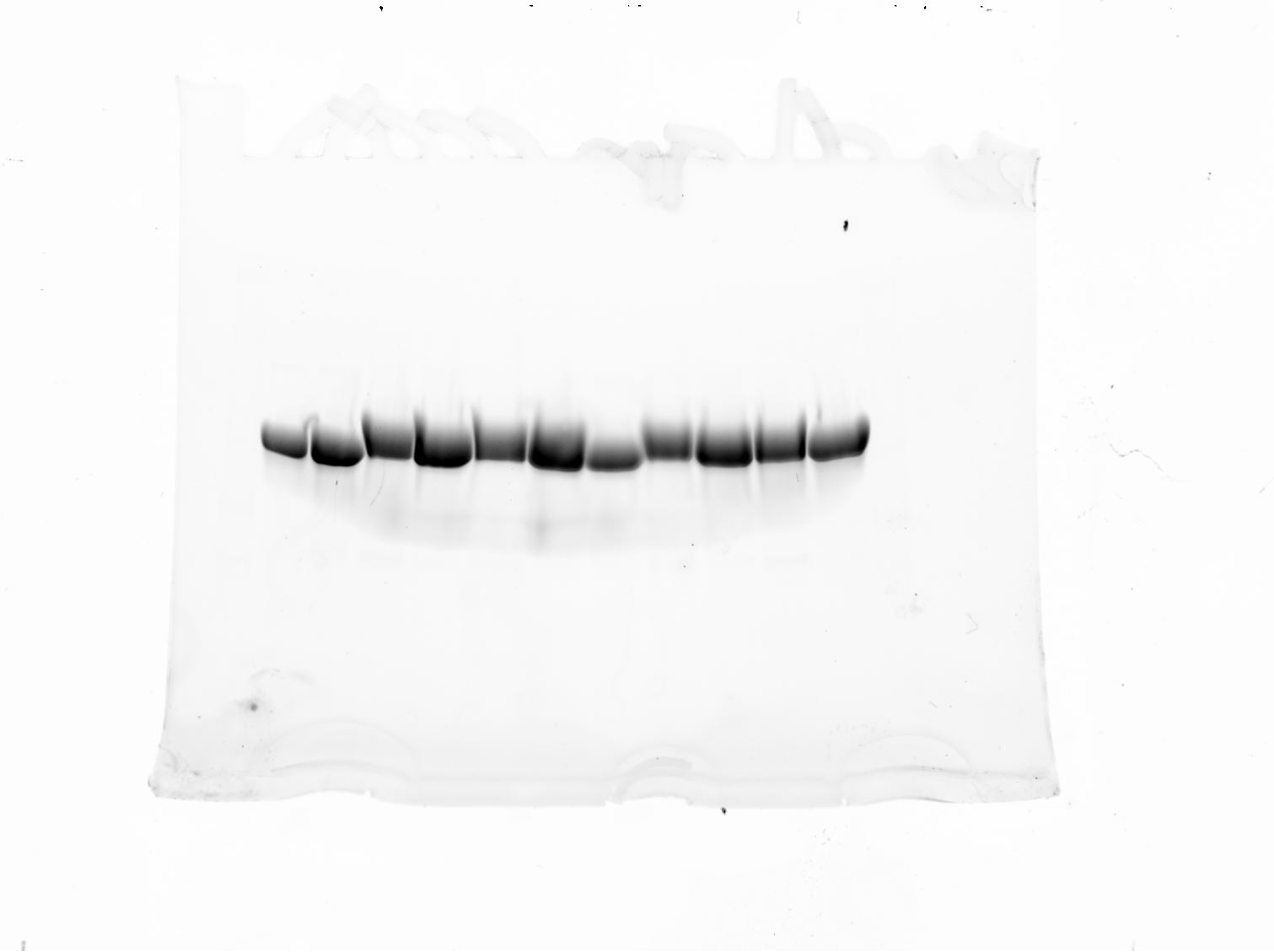

Supplement: Figure 4—source data 1. [file elife-79183-fig4-data1.zip › Figure 4-source data 1/Figure4E-source data1/Figure4E-source data1-raw BRC panel.tif]

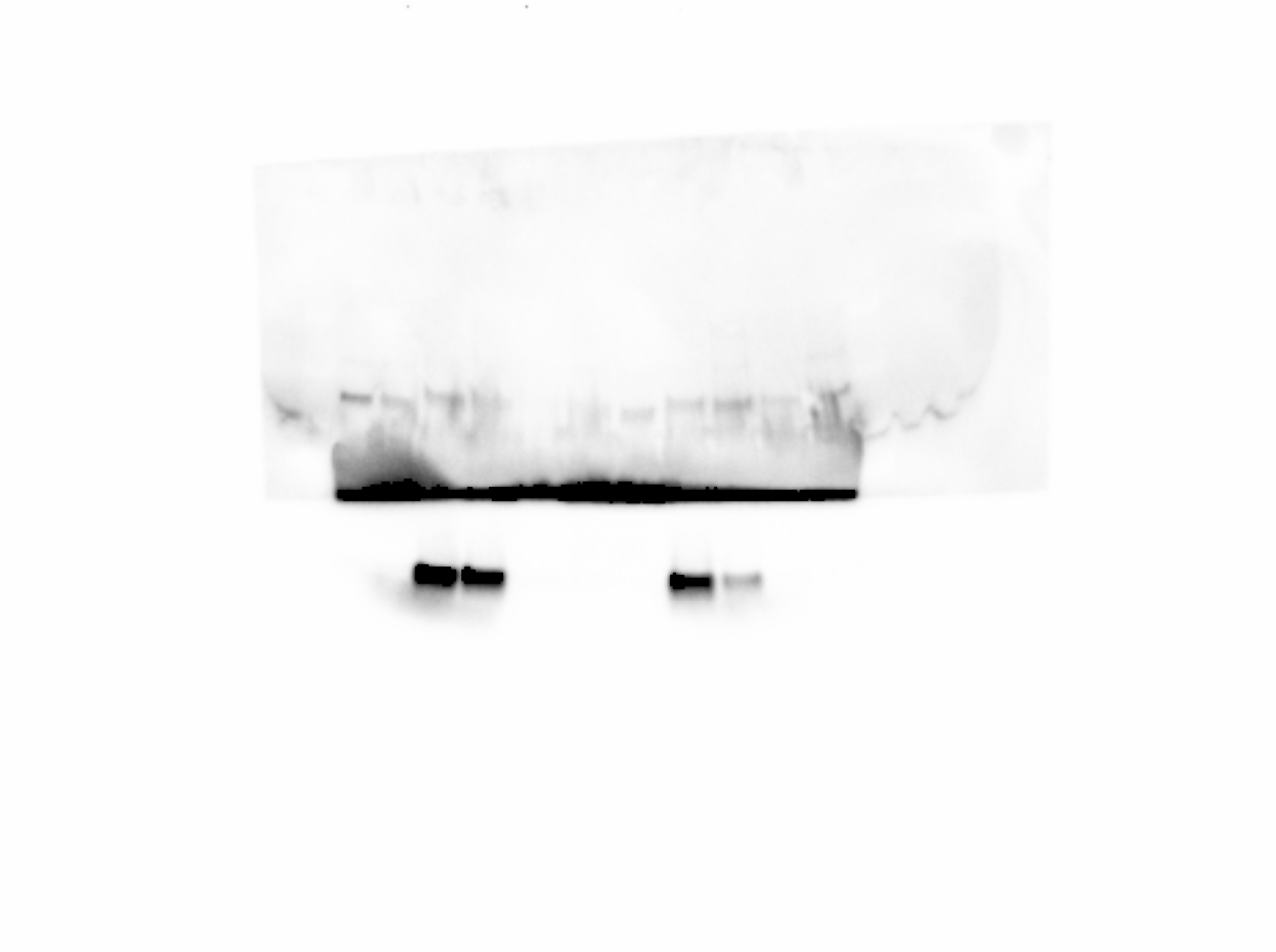

Supplement: Figure 4—source data 1. [file elife-79183-fig4-data1.zip › Figure 4-source data 1/Figure4E-source data1/Figure4E-source data2-raw RAD51 panel.tif]

**E**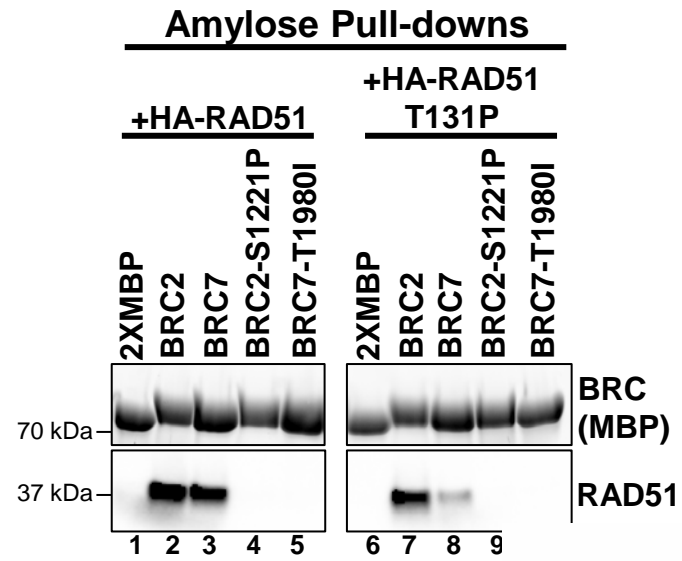

**Amylose Pull-downs**

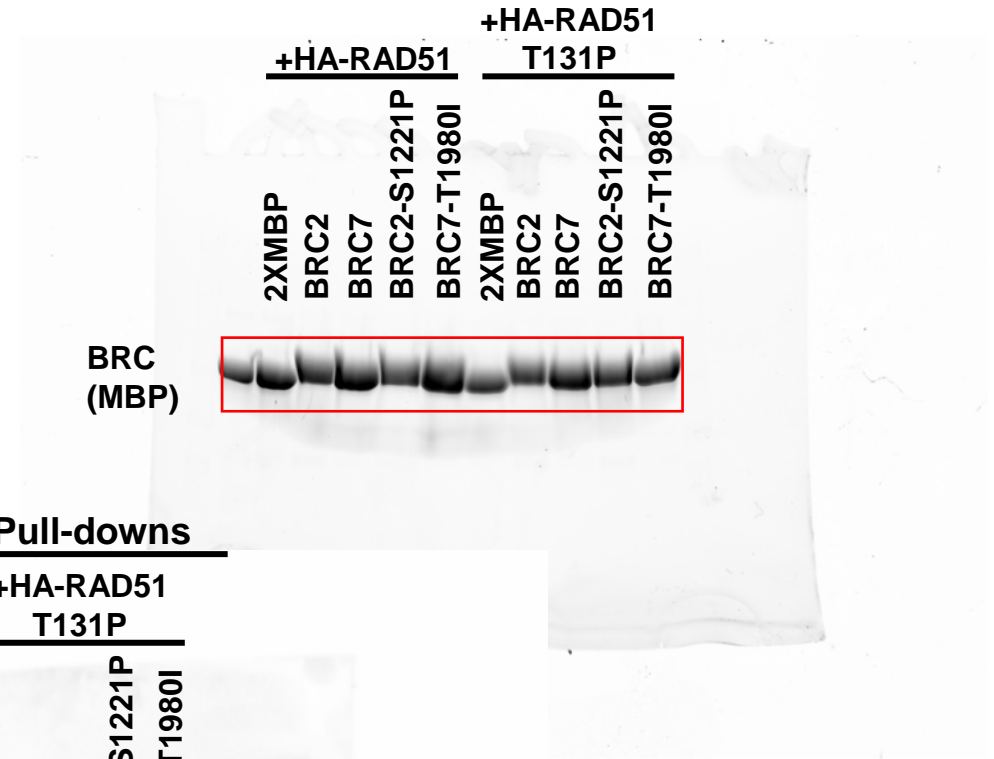

**Amylose Pull-downs**

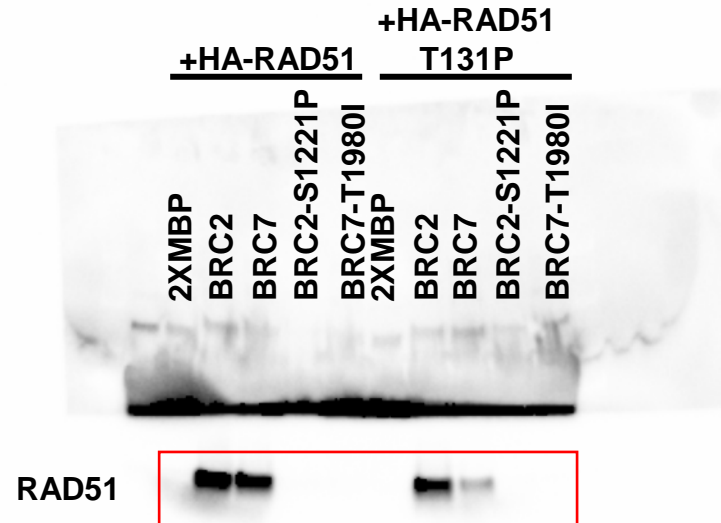

Supplement: Figure 4—source data 1. [file elife-79183-fig4-data1.zip › Figure 4-source data 1/Figure4E-source data1/Supplementary Figure4E-source data4-highlightedbandsandlabeled.pdf]

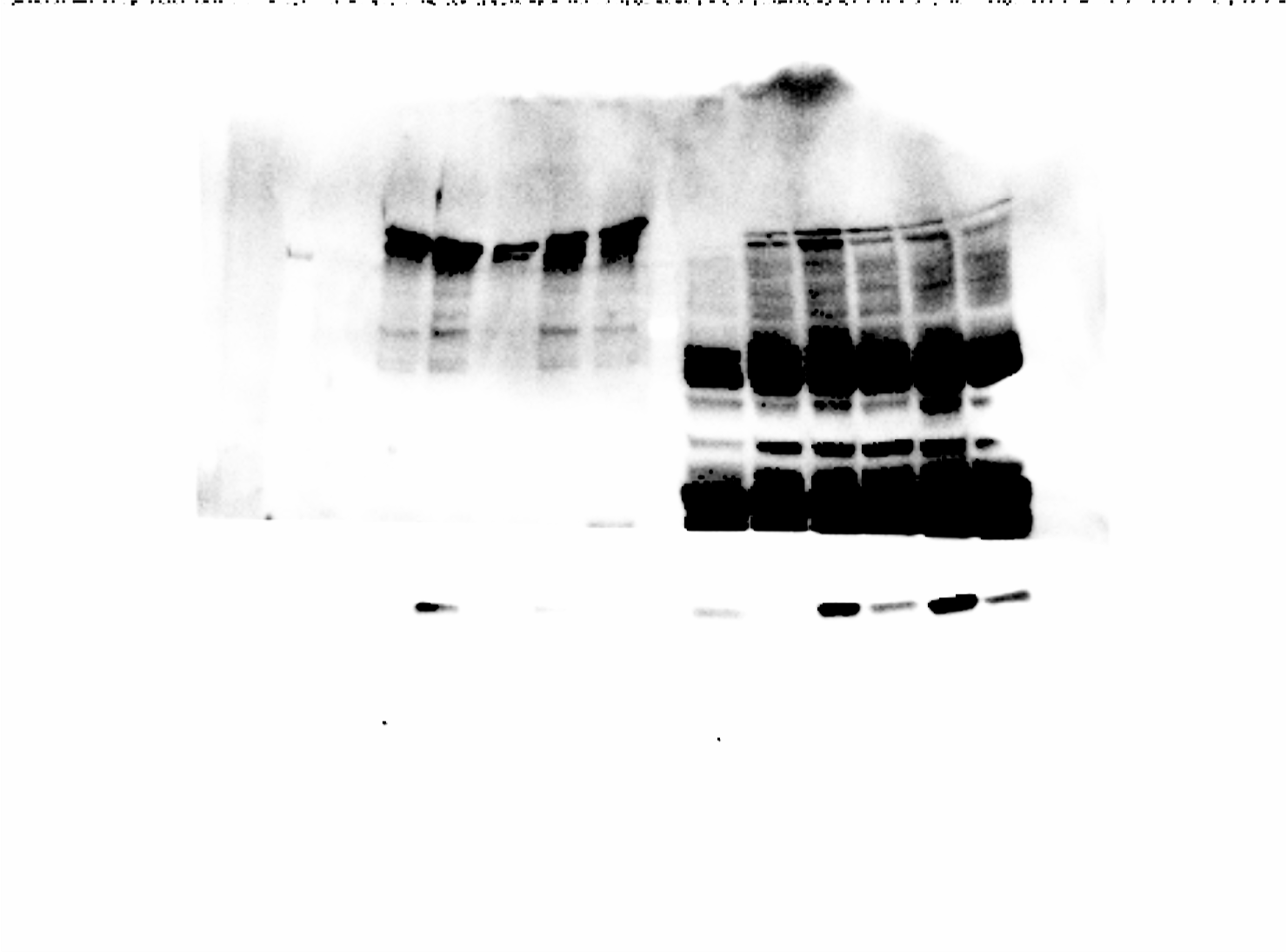

Supplement: Figure 4—figure supplement 1—source data 1. [file elife-79183-fig4-figsupp1-data1.zip › Figure 4-figure supplement 1-source data1/Figure 4-figure supplement 1A-source data 1/Figure 4-figure supplement 1A-source data1-raw BRCA panel TCL.tif]

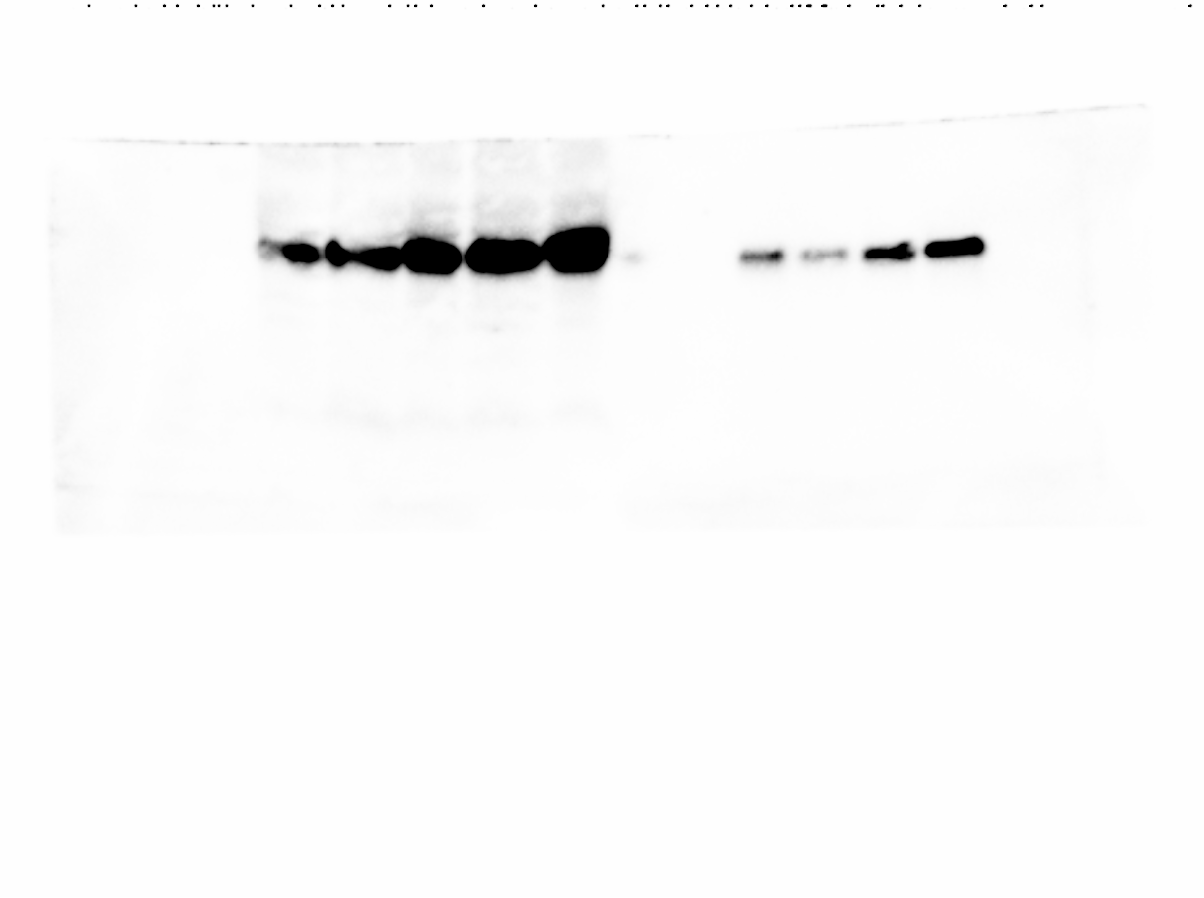

Supplement: Figure 4—figure supplement 1—source data 1. [file elife-79183-fig4-figsupp1-data1.zip › Figure 4-figure supplement 1-source data1/Figure 4-figure supplement 1A-source data 1/Figure 4-figure supplement 1A-source data2-raw RAD51 panel TCL.tif]

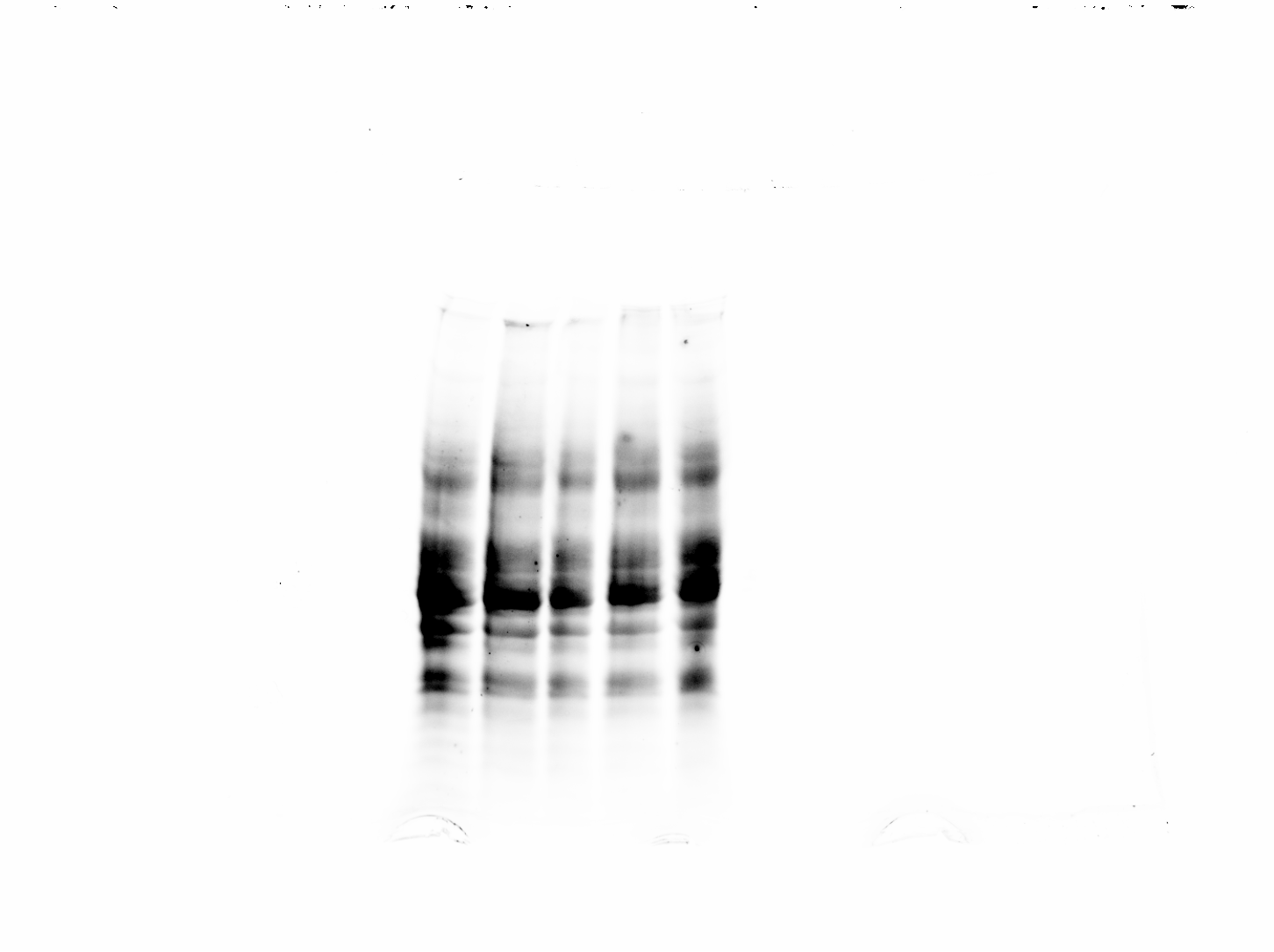

Supplement: Figure 4—figure supplement 1—source data 1. [file elife-79183-fig4-figsupp1-data1.zip › Figure 4-figure supplement 1-source data1/Figure 4-figure supplement 1A-source data 1/Figure 4-figure supplement 1A-source data3-rawStainFreepanel TCL.tif]

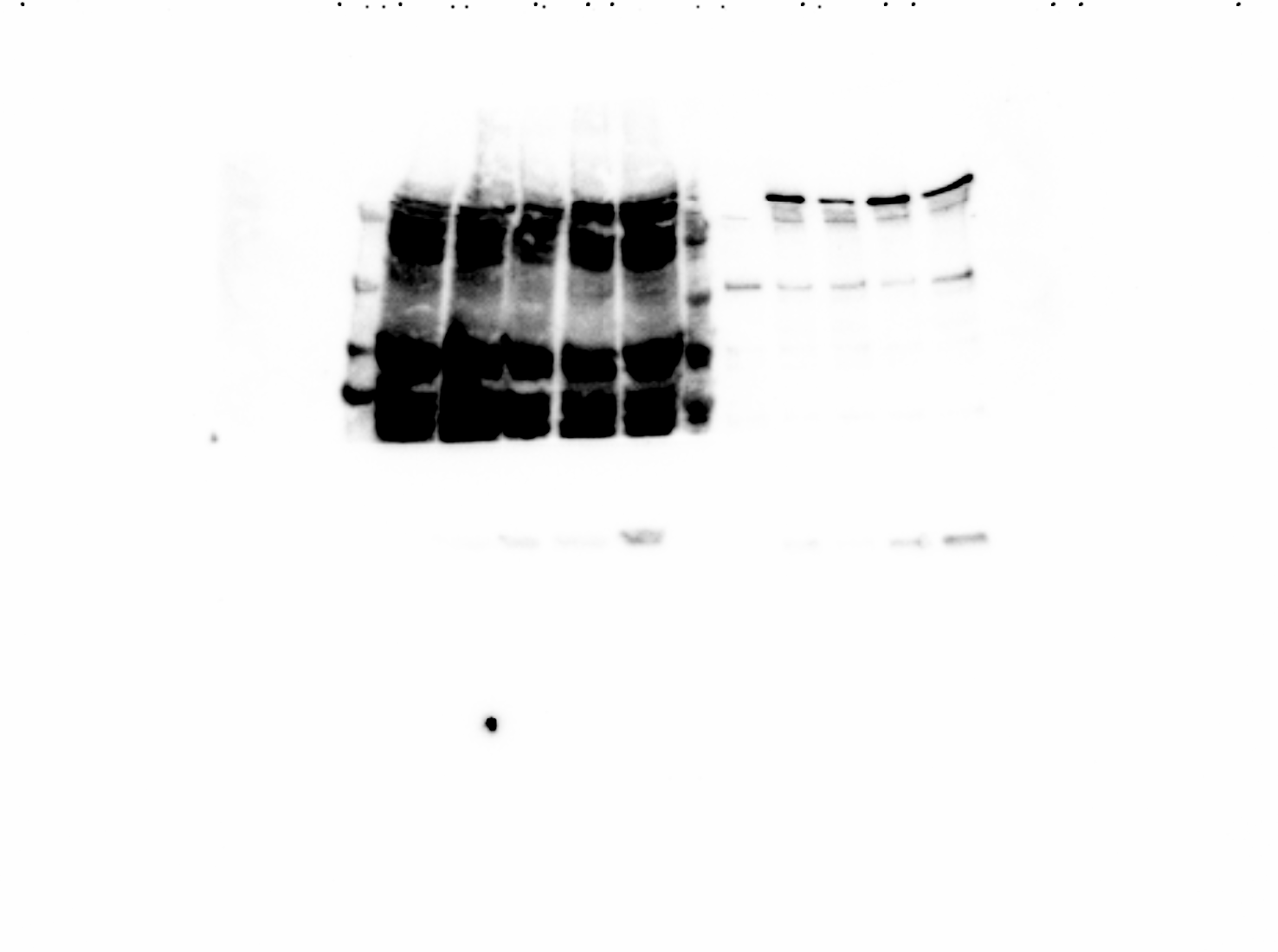

Supplement: Figure 4—figure supplement 1—source data 1. [file elife-79183-fig4-figsupp1-data1.zip › Figure 4-figure supplement 1-source data1/Figure 4-figure supplement 1A-source data 1/Figure 4-figure supplement 1A-source data4-raw BRCA panel PD.tif]

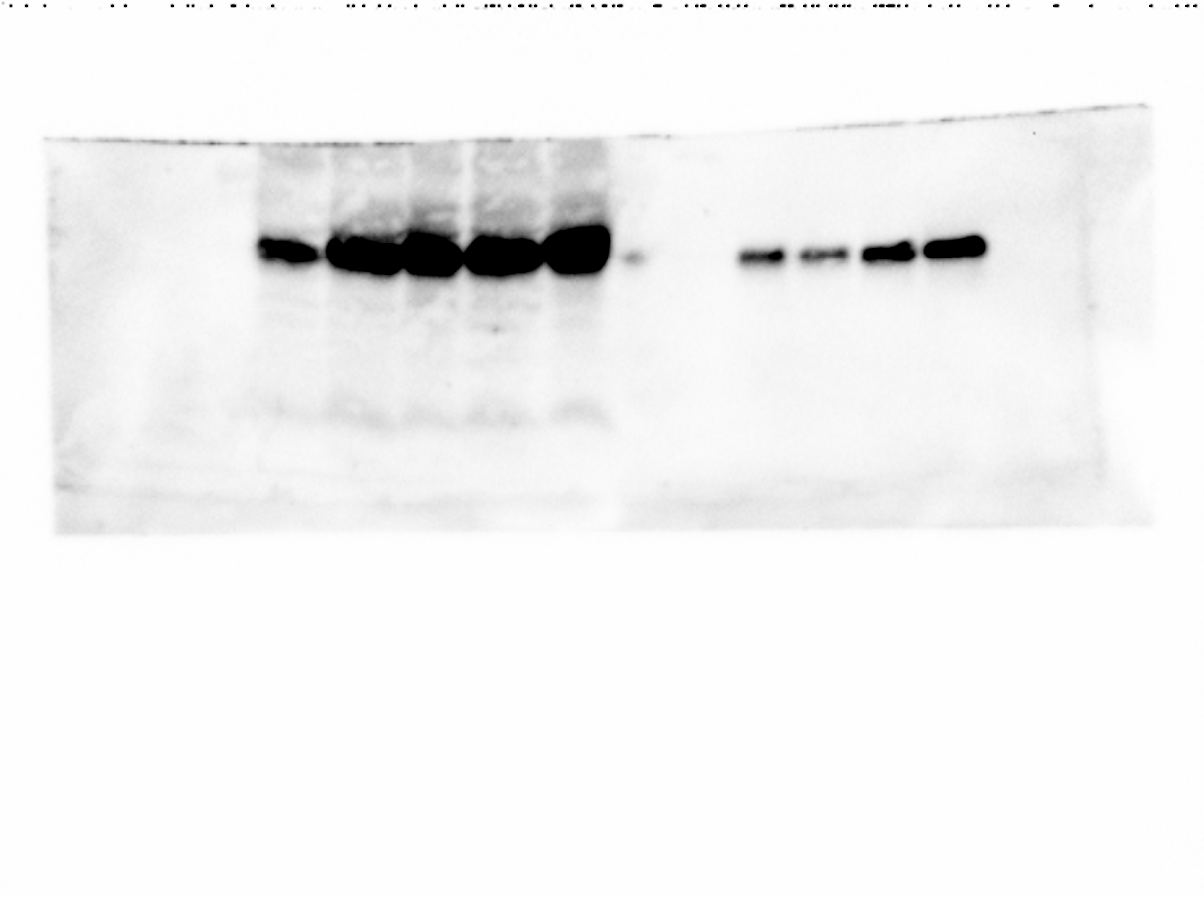

Supplement: Figure 4—figure supplement 1—source data 1. [file elife-79183-fig4-figsupp1-data1.zip › Figure 4-figure supplement 1-source data1/Figure 4-figure supplement 1A-source data 1/Figure 4-figure supplement 1A-source data5-raw RAD51 panel PD.tif]

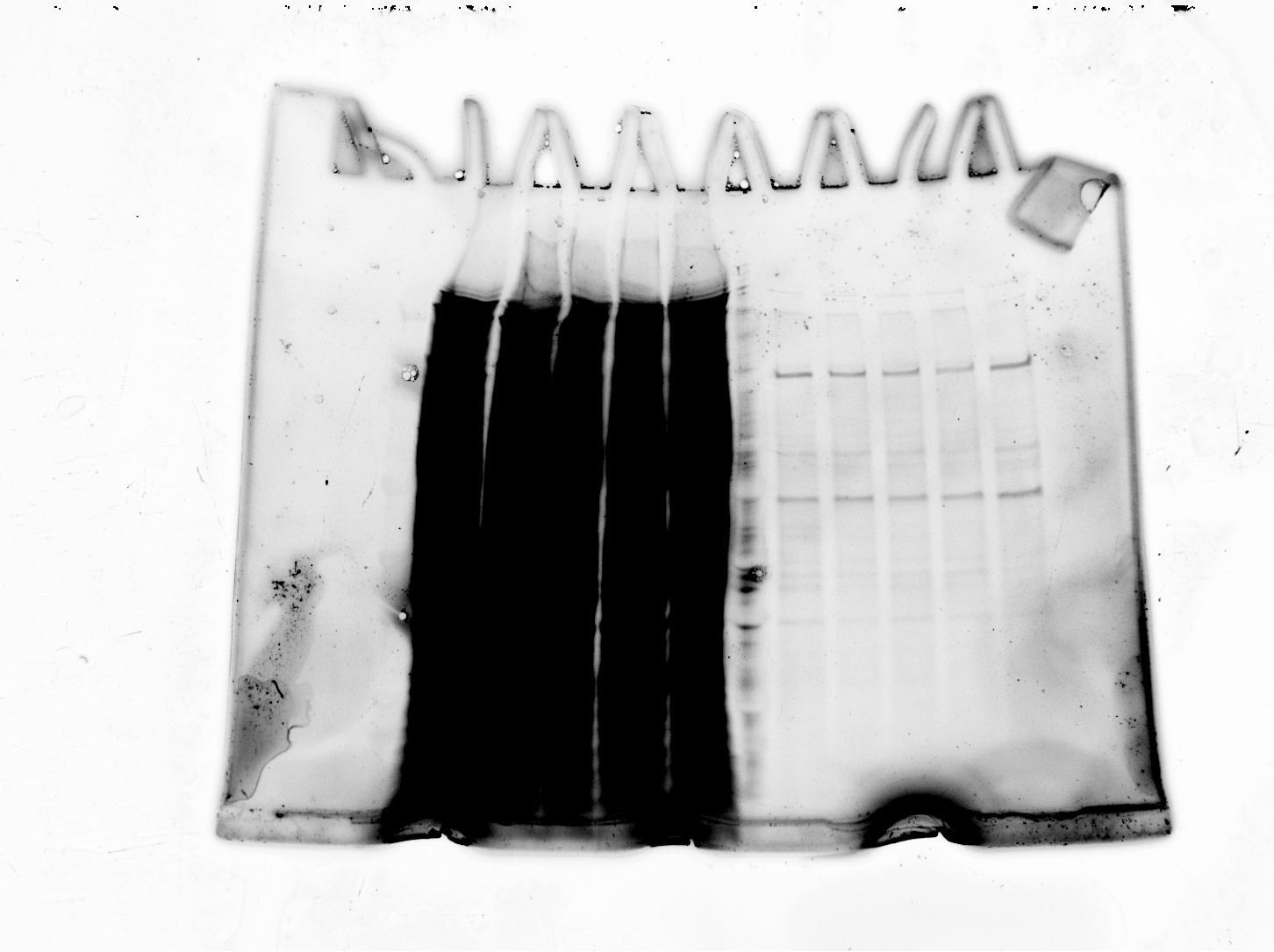

Supplement: Figure 4—figure supplement 1—source data 1. [file elife-79183-fig4-figsupp1-data1.zip › Figure 4-figure supplement 1-source data1/Figure 4-figure supplement 1A-source data 1/Figure 4-figure supplement 1A-source data6-rawStainFreepanel PD.tif]

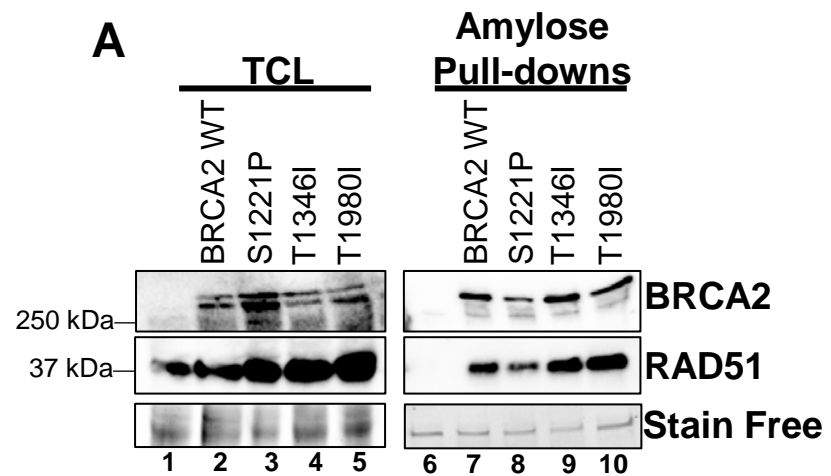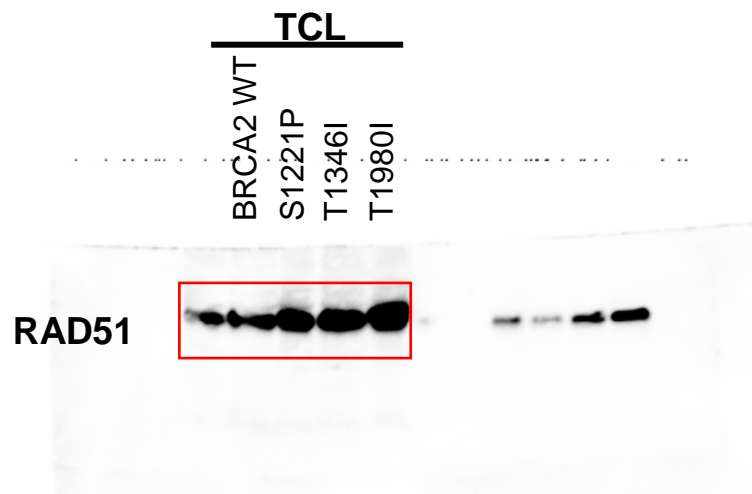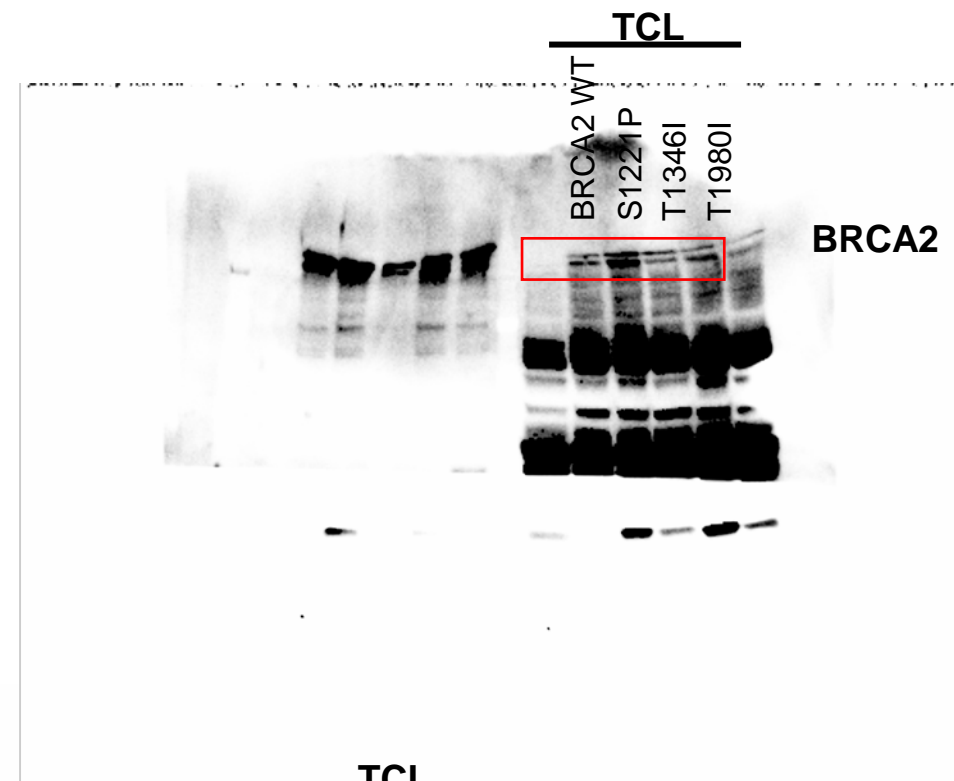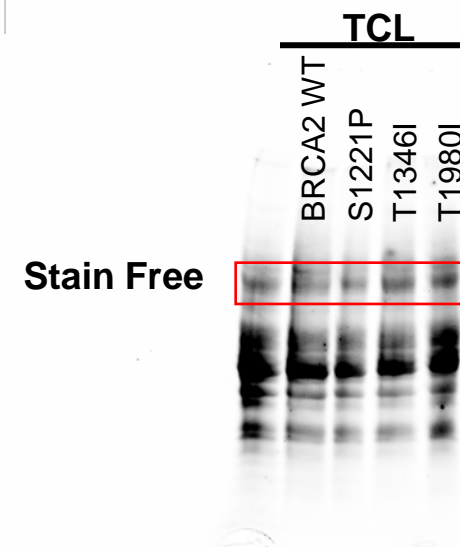

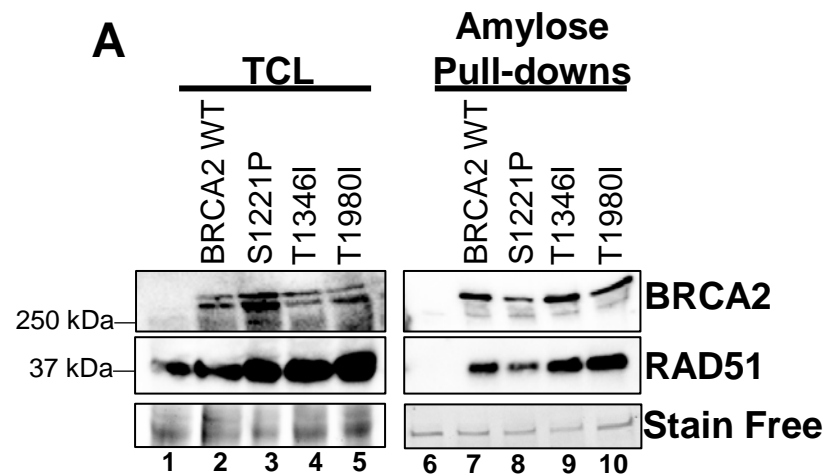

**Amylose Pull-downs**

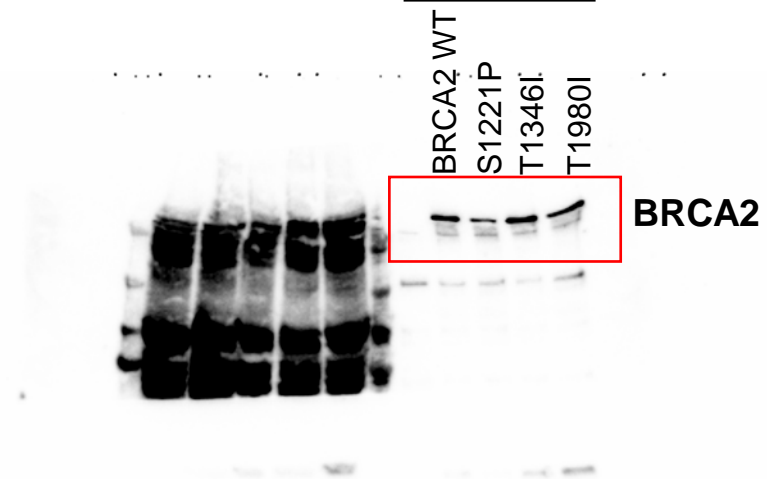

**Amylose Pull-downs**

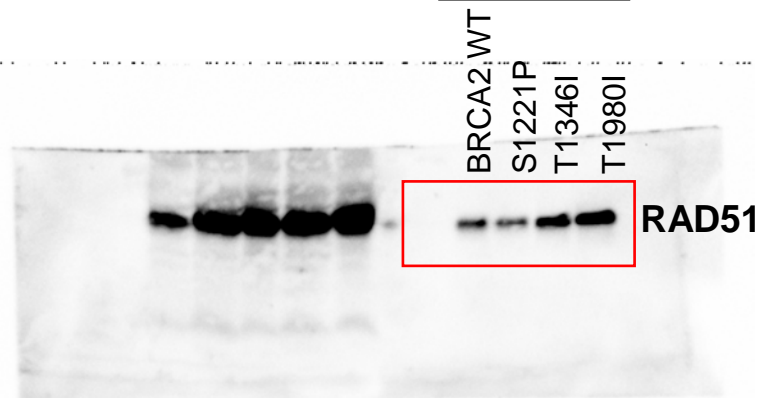

**Amylose Pull-downs**

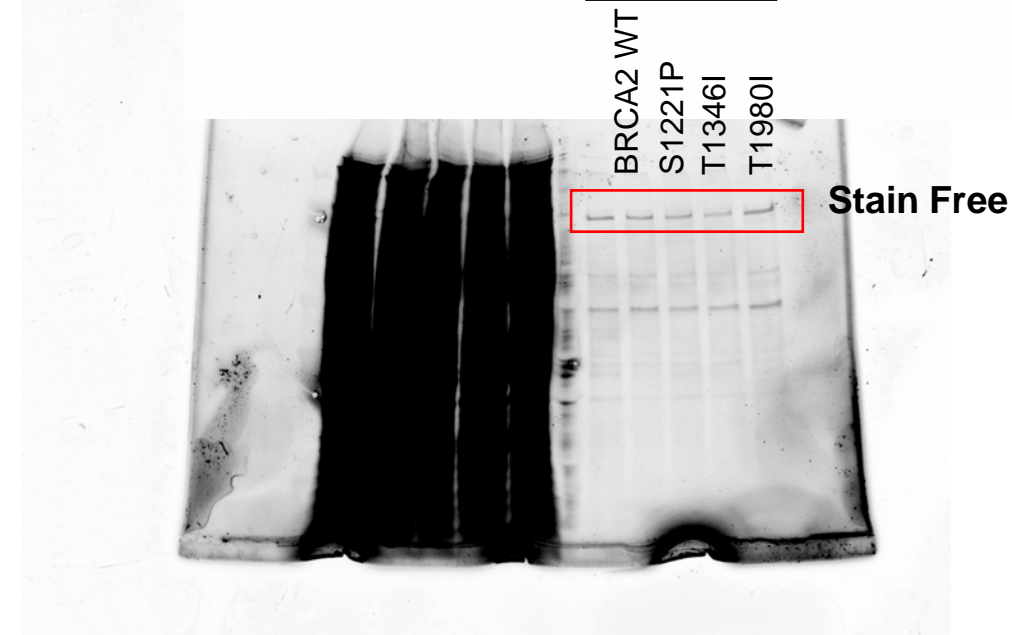

Supplement: Figure 4—figure supplement 1—source data 1. [file elife-79183-fig4-figsupp1-data1.zip › Figure 4-figure supplement 1-source data1/Figure 4-figure supplement 1A-source data 1/Figure 4-figure supplement 1A-source data7-highlightedbandsandlabeled.pdf]

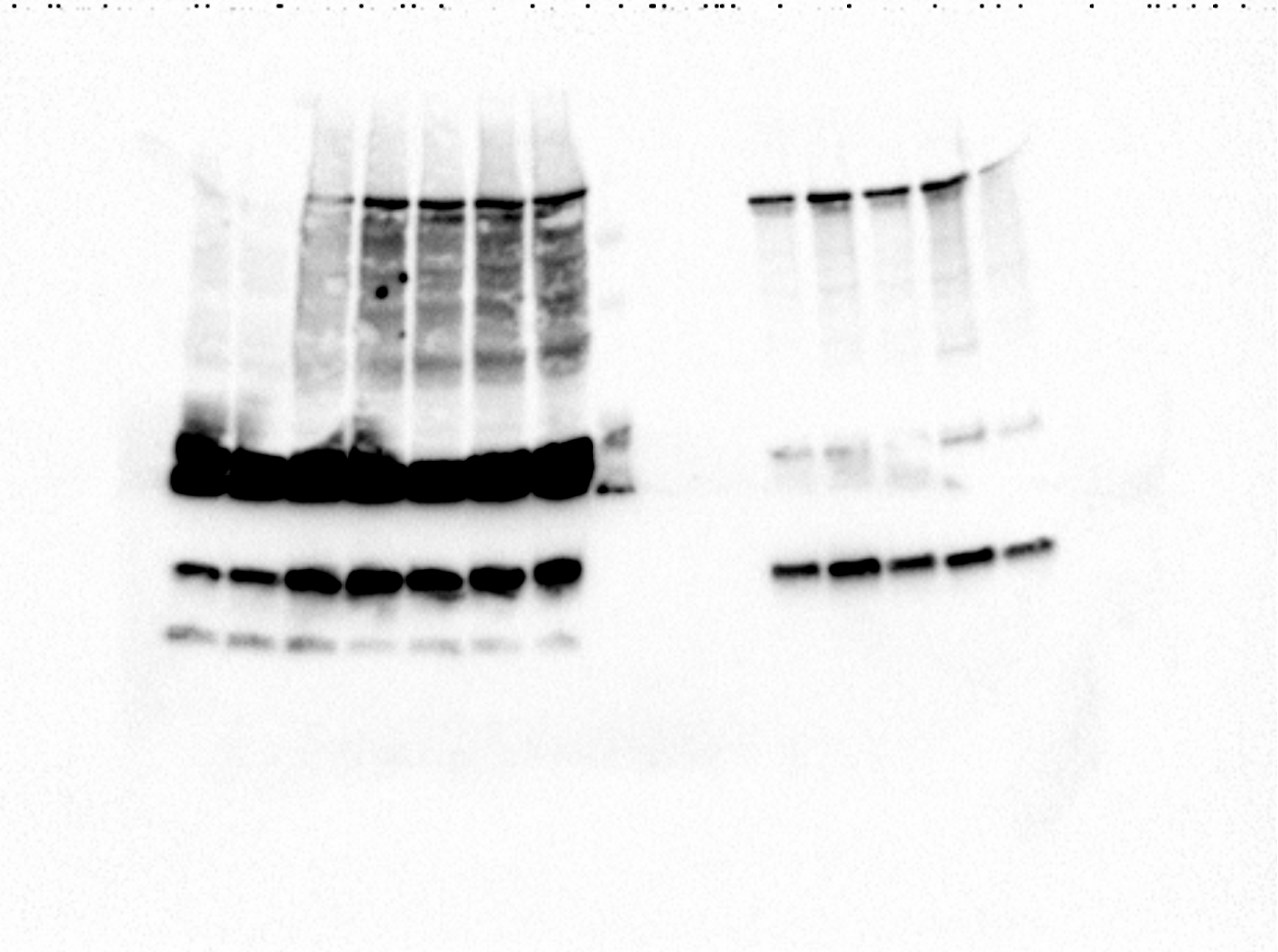

Supplement: Figure 4—figure supplement 1—source data 1. [file elife-79183-fig4-figsupp1-data1.zip › Figure 4-figure supplement 1-source data1/Figure 4-figure supplement 1B-source data 1/Figure 4-figure supplement 1B-source data1-BRCA2-RAD51panels.tif]

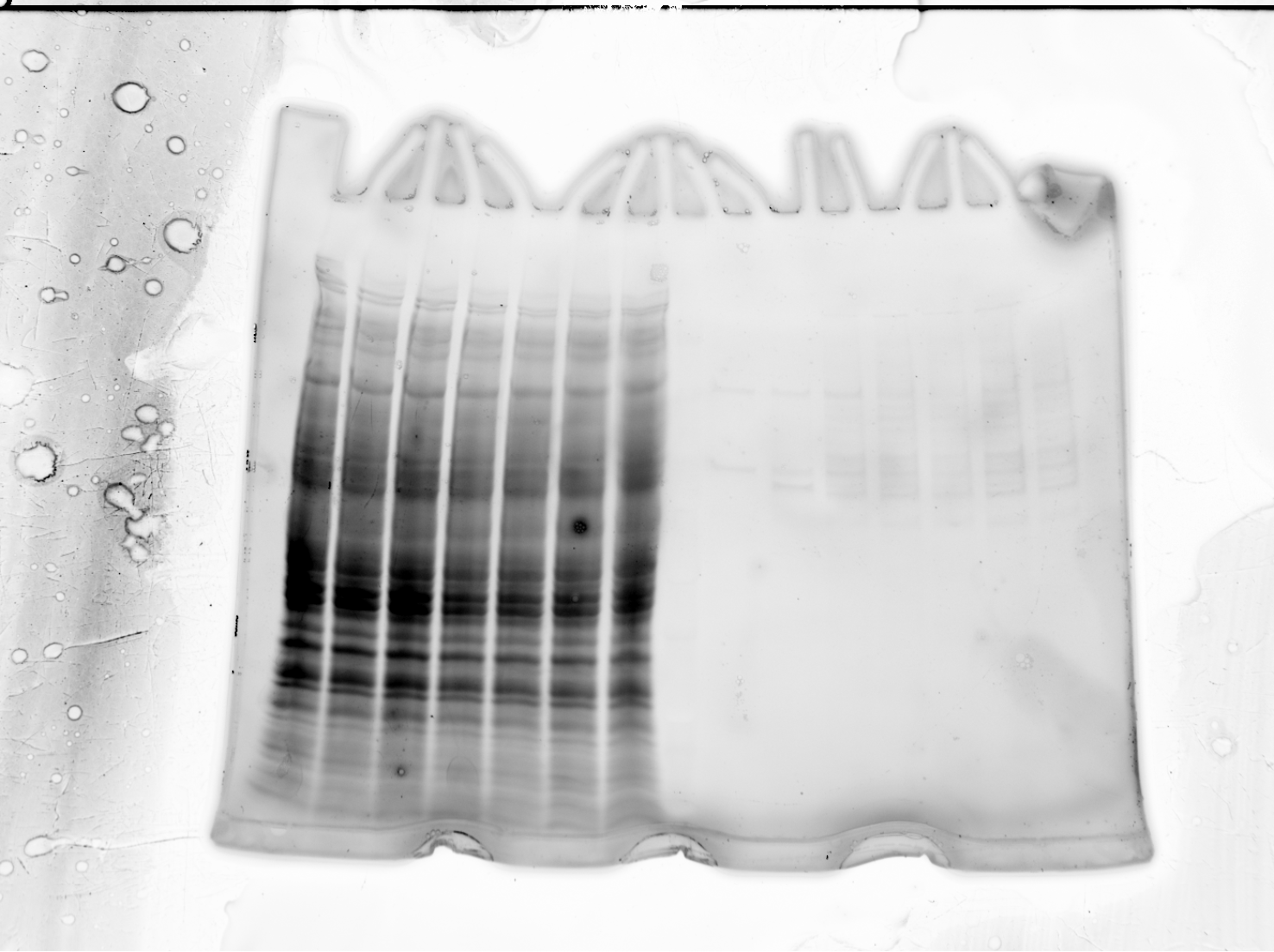

Supplement: Figure 4—figure supplement 1—source data 1. [file elife-79183-fig4-figsupp1-data1.zip › Figure 4-figure supplement 1-source data1/Figure 4-figure supplement 1B-source data 1/Figure 4-figure supplement 1B-source data2-StainFree panel TCL.tif]

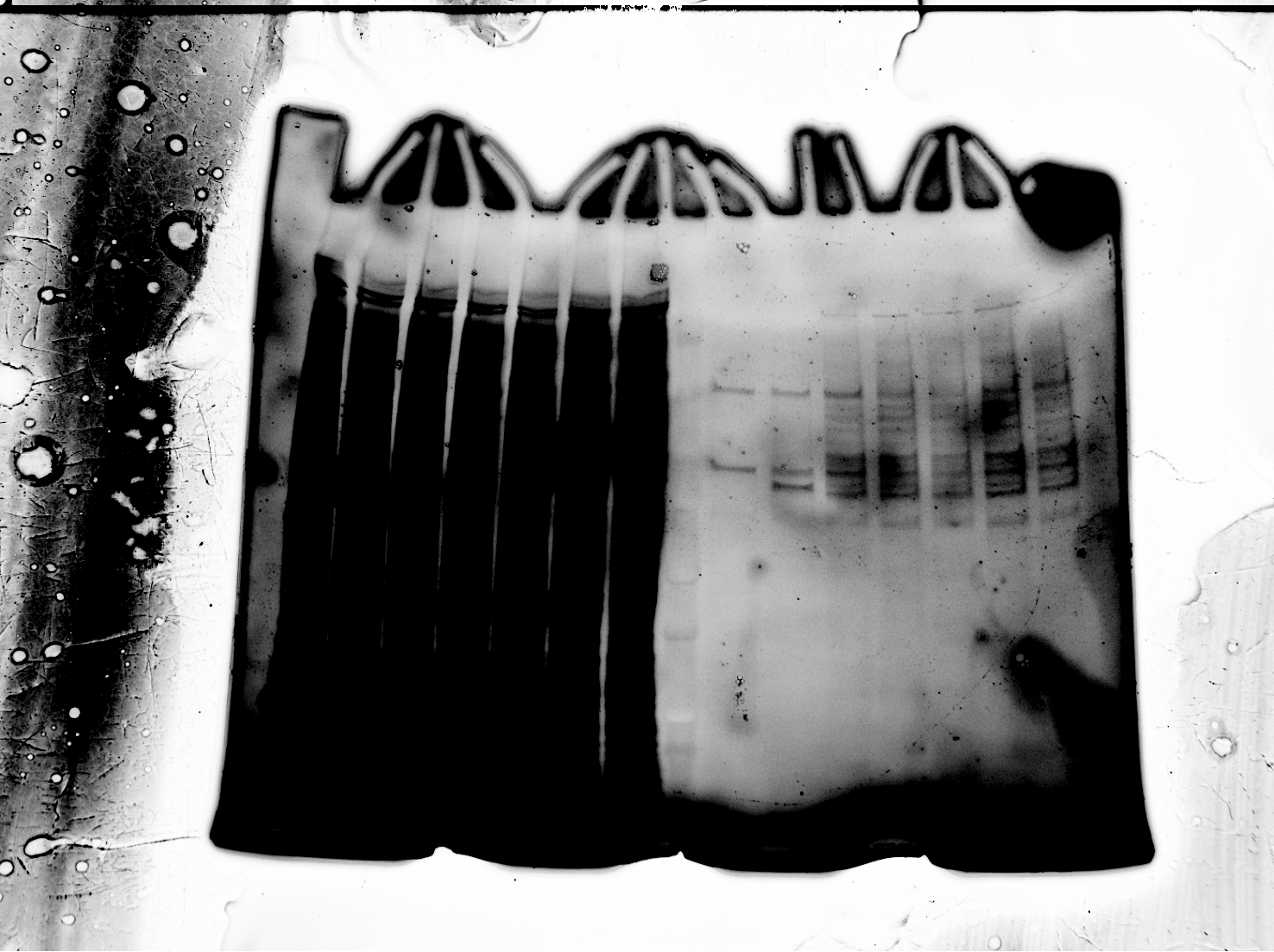

Supplement: Figure 4—figure supplement 1—source data 1. [file elife-79183-fig4-figsupp1-data1.zip › Figure 4-figure supplement 1-source data1/Figure 4-figure supplement 1B-source data 1/Figure 4-figure supplement 1B-source data3-StainFree panel PD.tif]

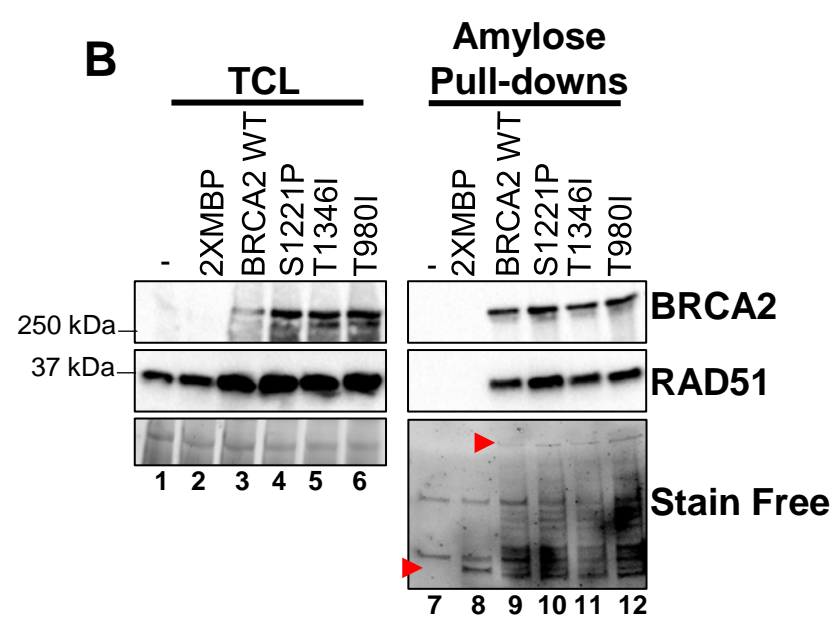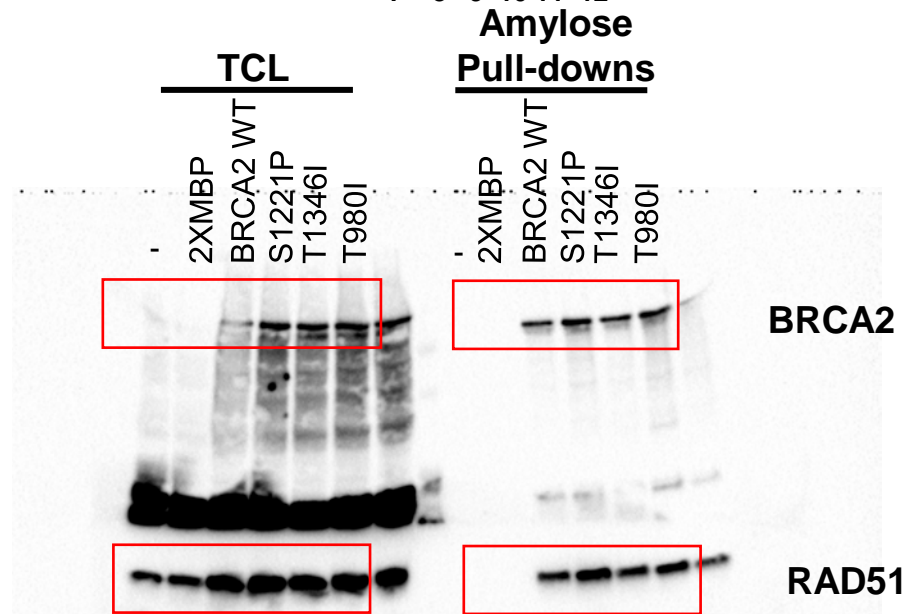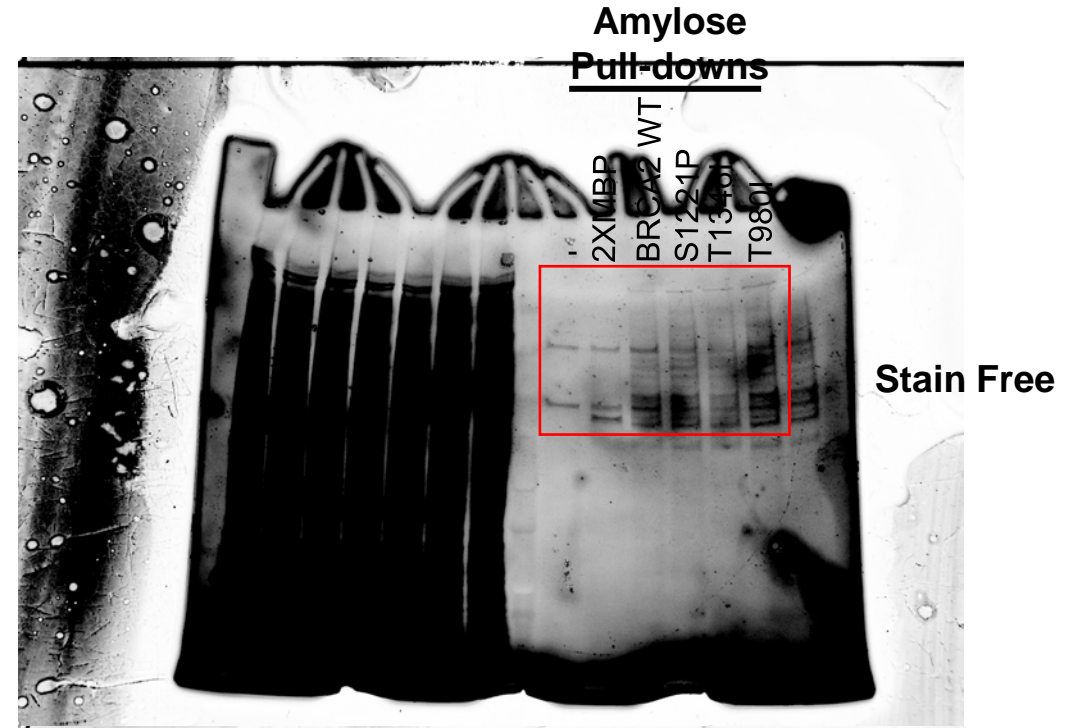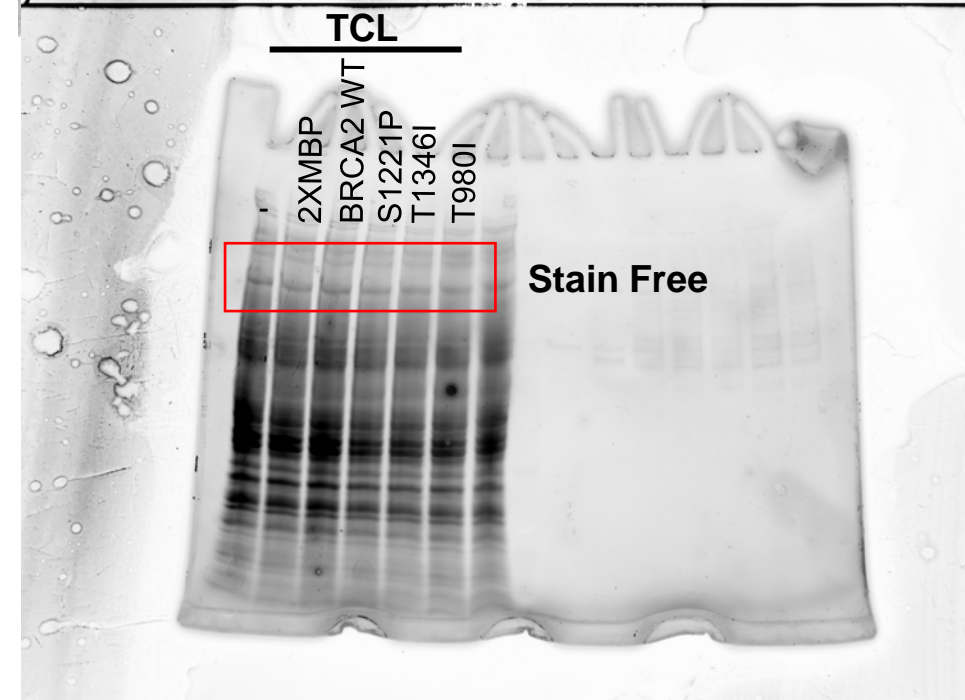

Supplement: Figure 4—figure supplement 1—source data 1. [file elife-79183-fig4-figsupp1-data1.zip › Figure 4-figure supplement 1-source data1/Figure 4-figure supplement 1B-source data 1/Figure 4-figure supplement 1B-source data4-highlightedbandsandlabeled.pdf]

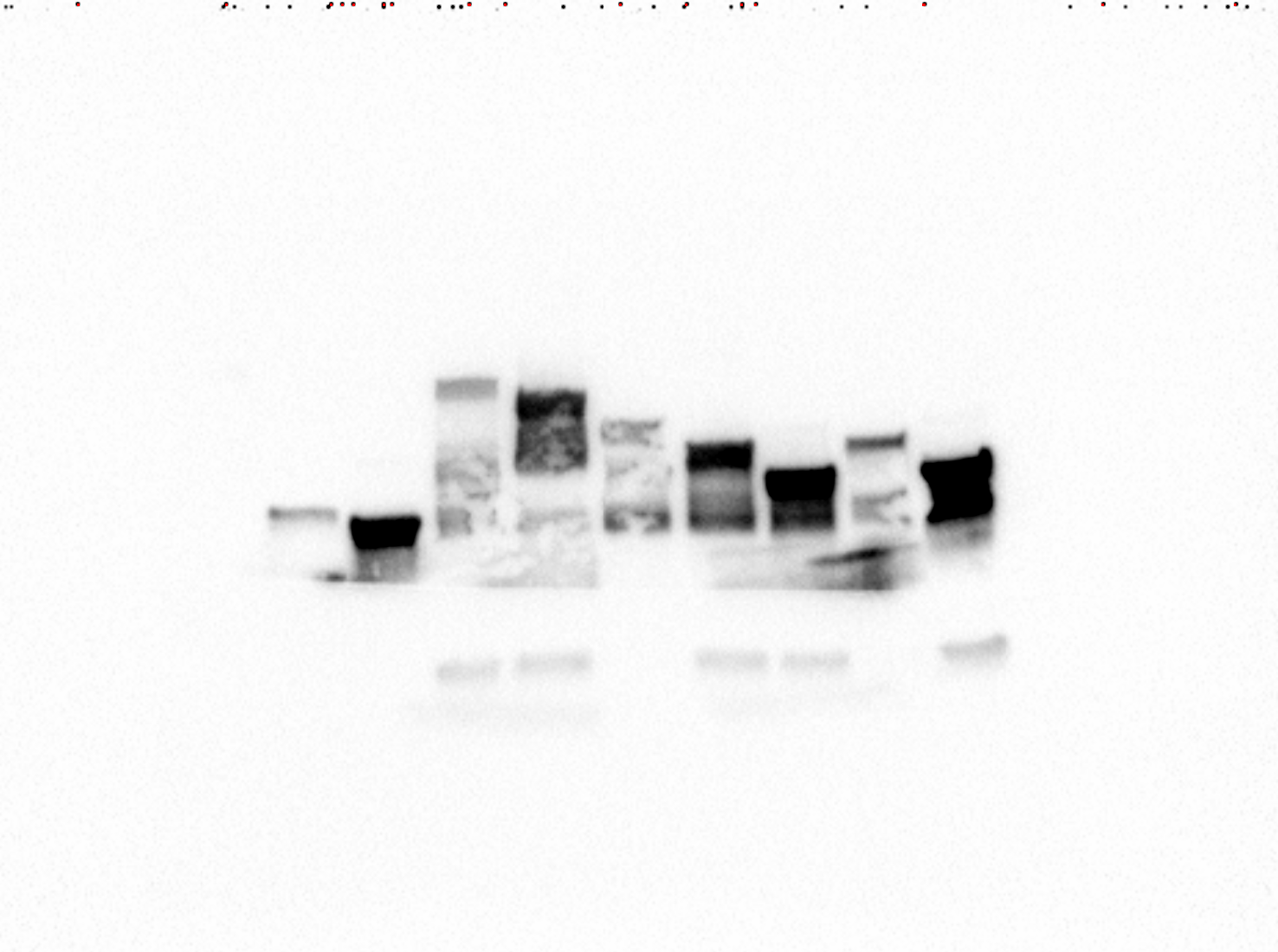

Supplement: Figure 4—figure supplement 2—source data 1. [file elife-79183-fig4-figsupp2-data1.zip › Figure 4-figure supplement 2-souce data1/Figure 4-figure supplement 2A-souce data1/Figure 4-figure supplement 2A-souce data1-BRCrepeats panel PD_3.0sec.tif]

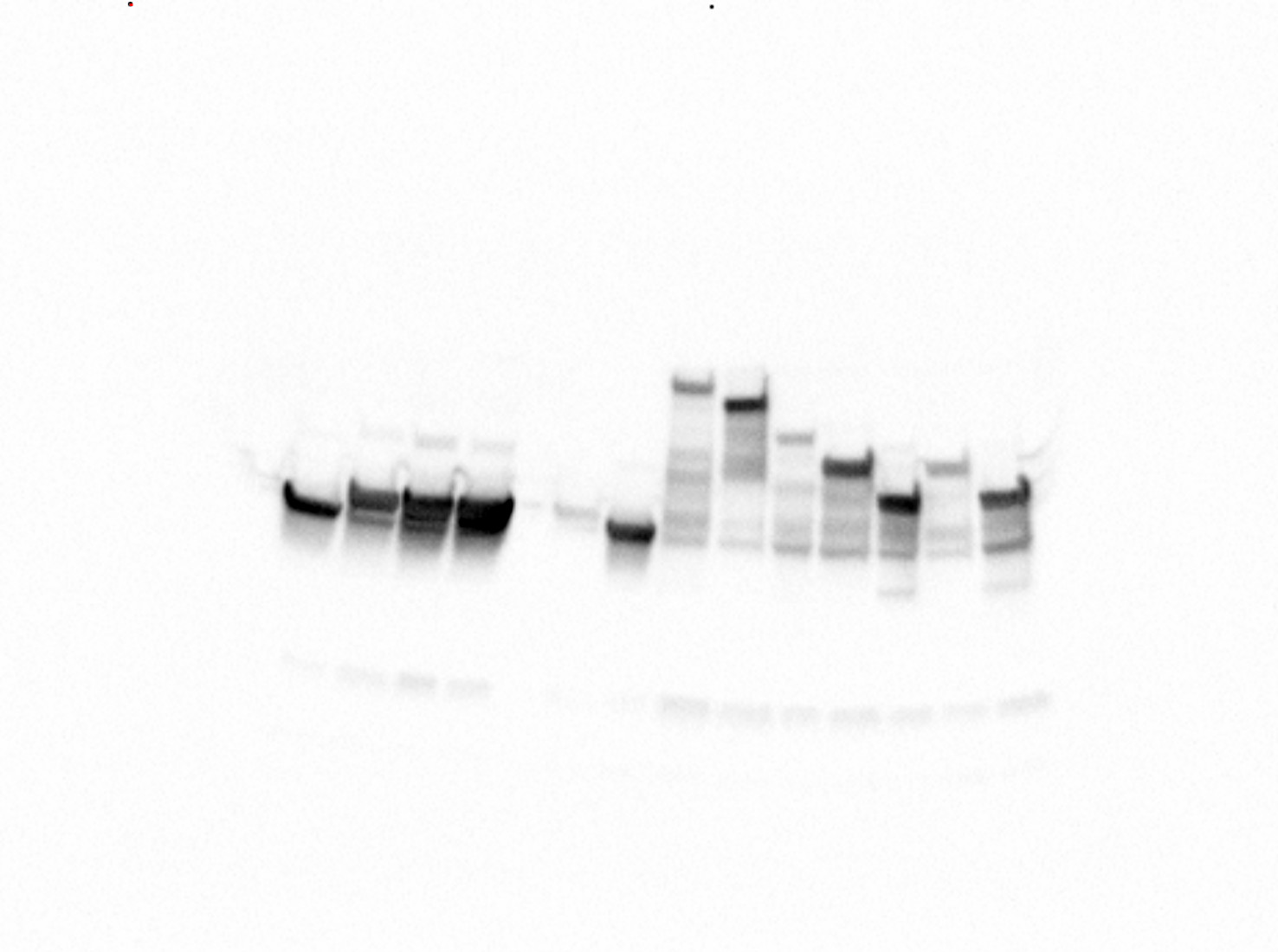

Supplement: Figure 4—figure supplement 2—source data 1. [file elife-79183-fig4-figsupp2-data1.zip › Figure 4-figure supplement 2-souce data1/Figure 4-figure supplement 2A-souce data1/Figure 4-figure supplement 2A-souce data2-BRCrepeats panelTCL_1sec.tif]

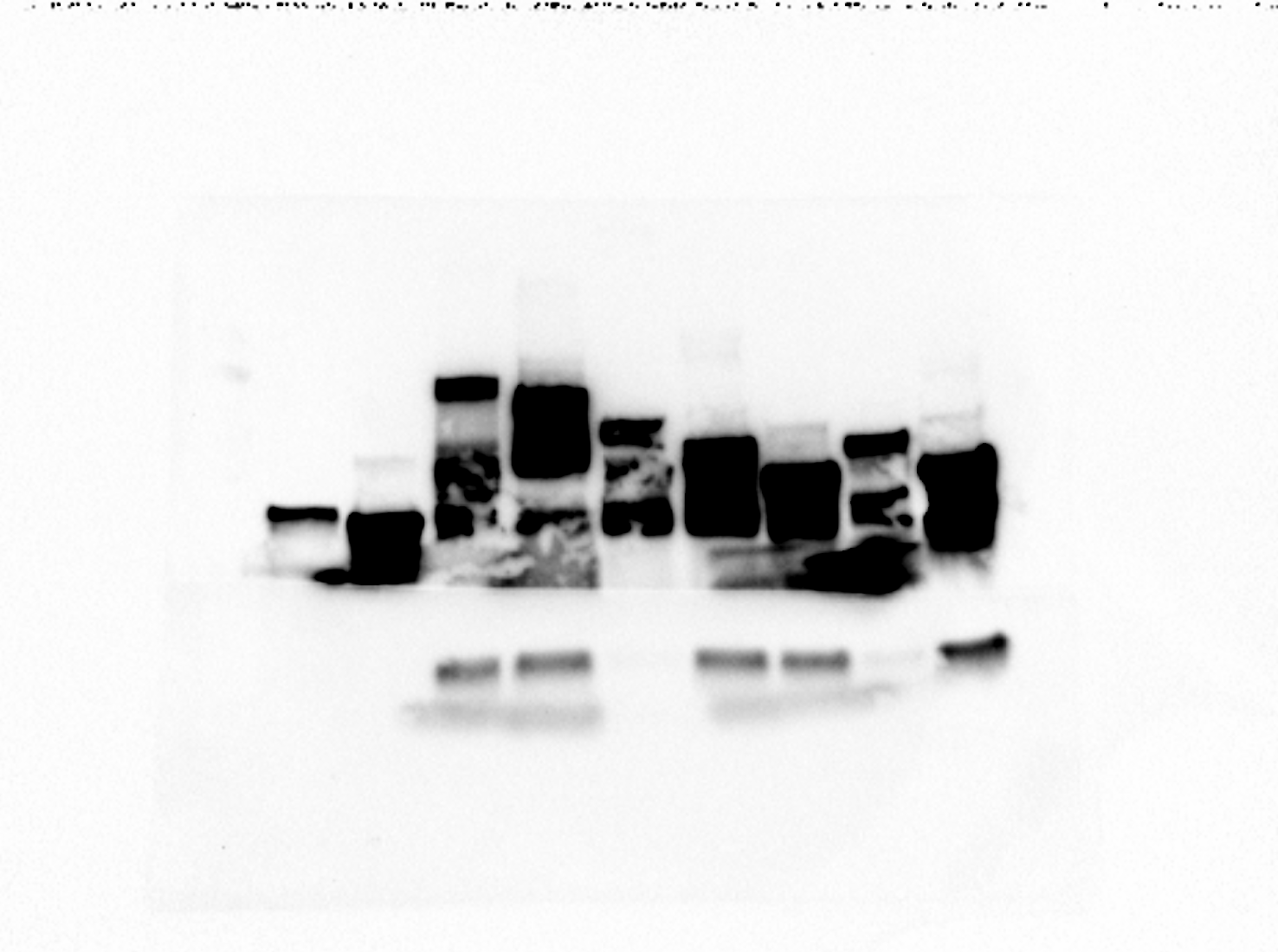

Supplement: Figure 4—figure supplement 2—source data 1. [file elife-79183-fig4-figsupp2-data1.zip › Figure 4-figure supplement 2-souce data1/Figure 4-figure supplement 2A-souce data1/Figure 4-figure supplement 2A-souce data3-RAD51 panelPD_21.1sec.tif]

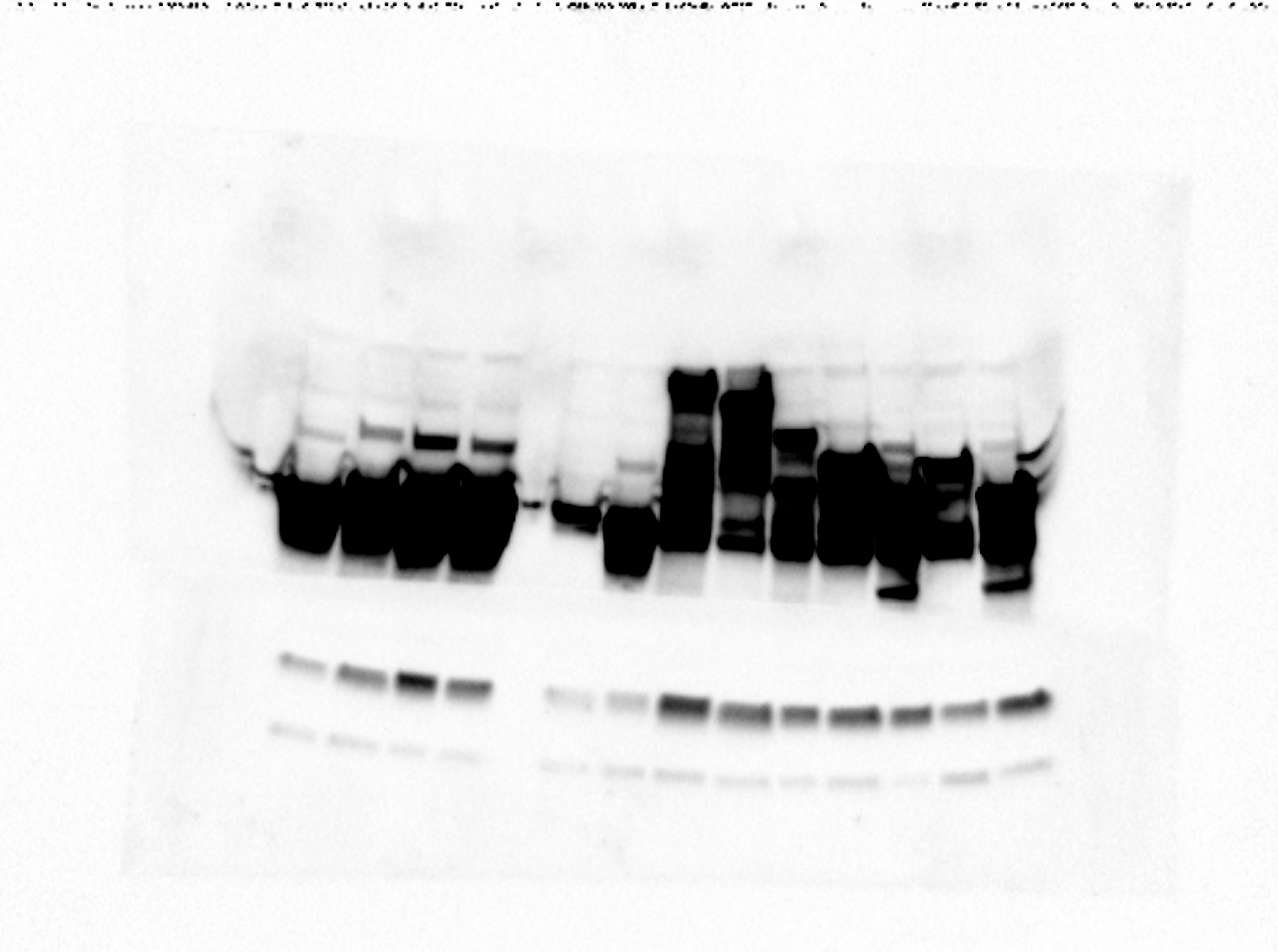

Supplement: Figure 4—figure supplement 2—source data 1. [file elife-79183-fig4-figsupp2-data1.zip › Figure 4-figure supplement 2-souce data1/Figure 4-figure supplement 2A-souce data1/Figure 4-figure supplement 2A-souce data4-RAD51 panelTCL__26.7sec.tif]

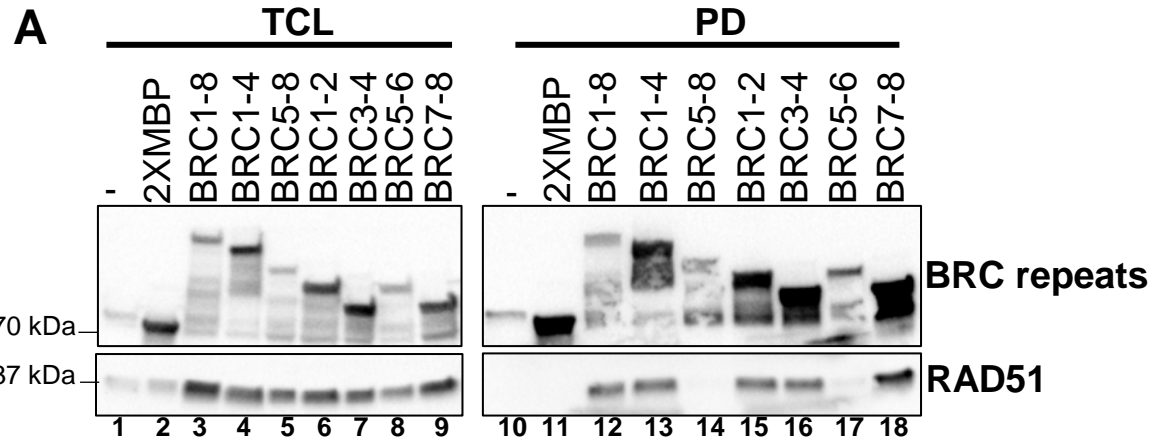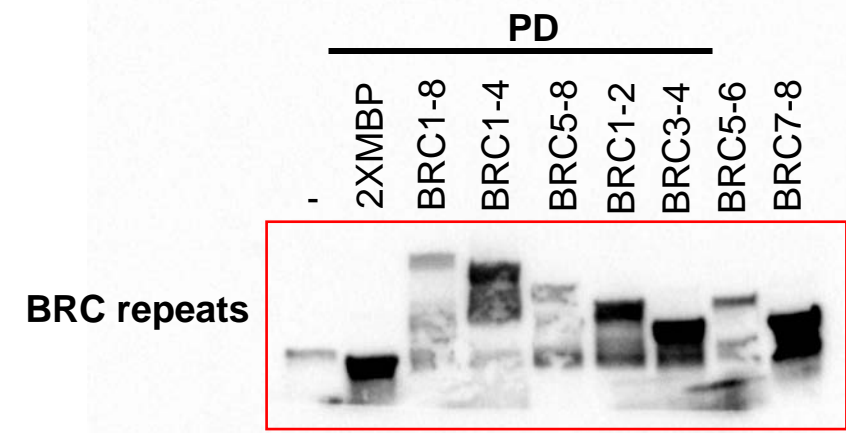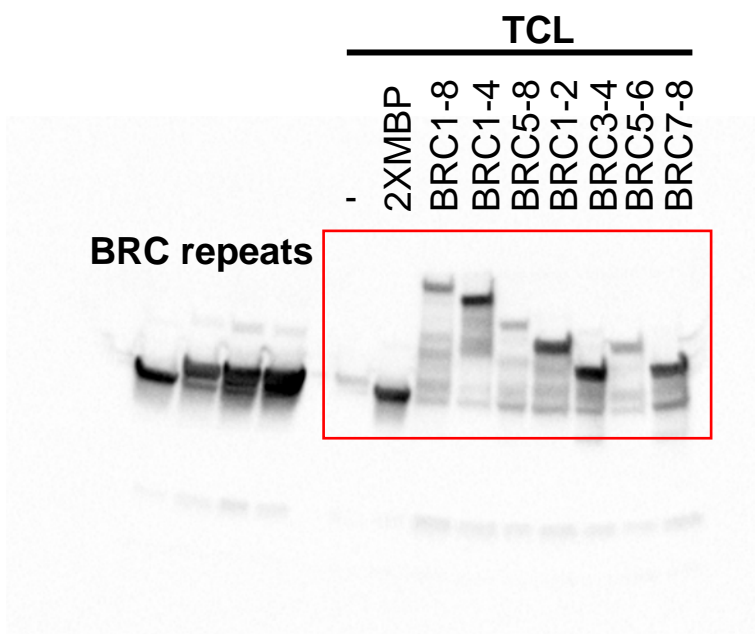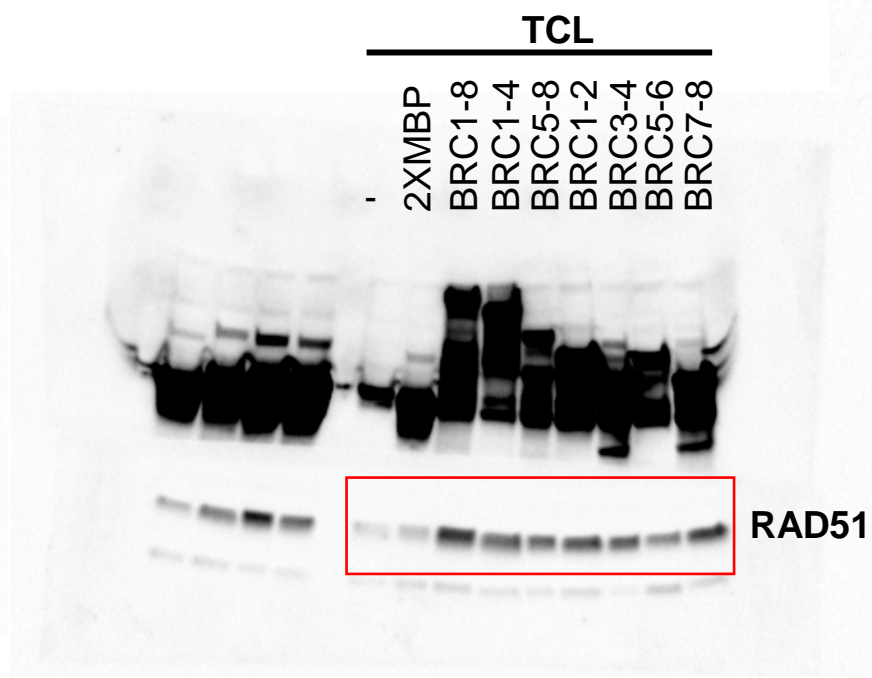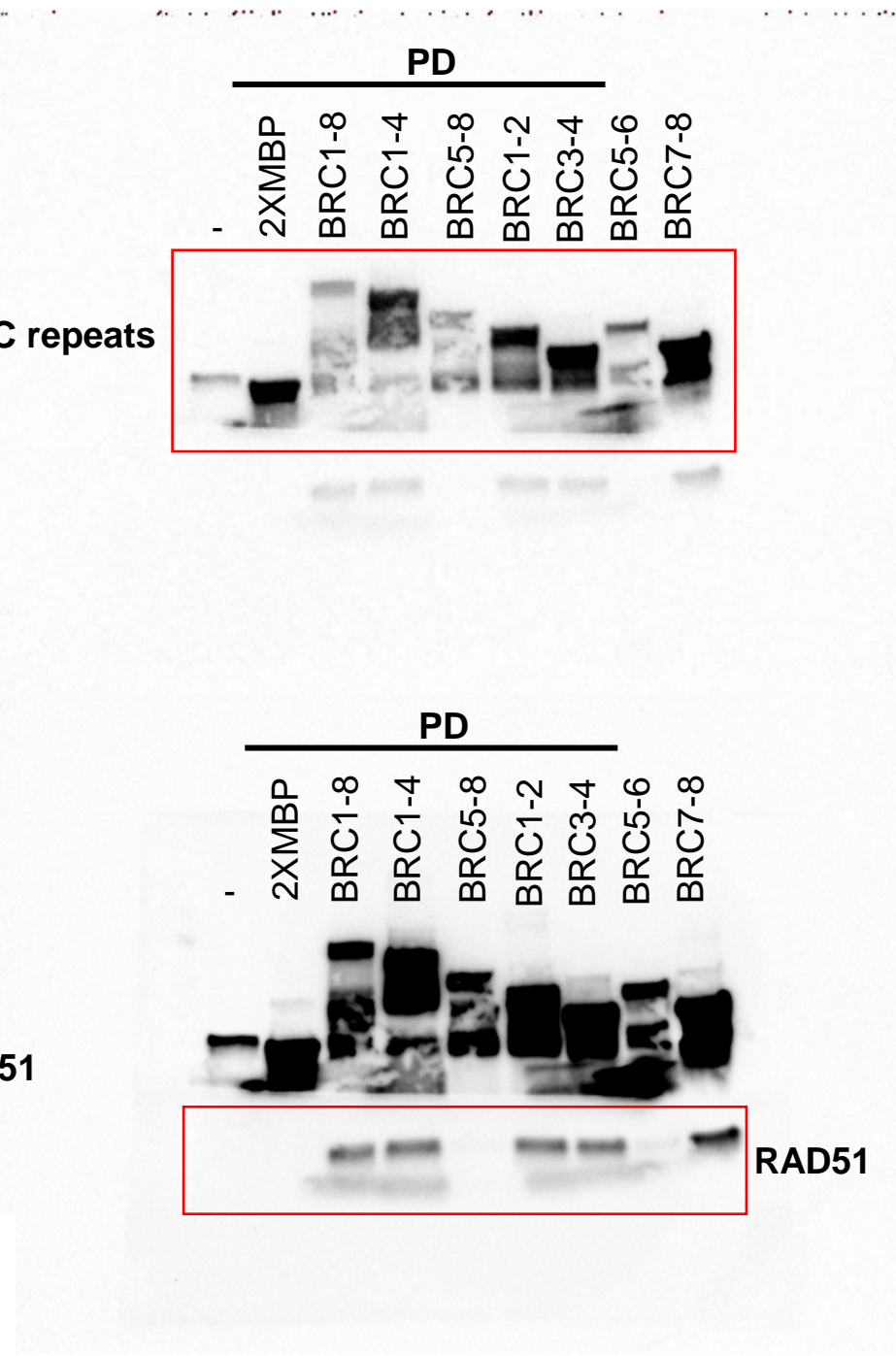

Supplement: Figure 4—figure supplement 2—source data 1. [file elife-79183-fig4-figsupp2-data1.zip › Figure 4-figure supplement 2-souce data1/Figure 4-figure supplement 2A-souce data1/Figure 4-figure supplement 2A-souce data5-highlightedbandsandlabeled.pdf]

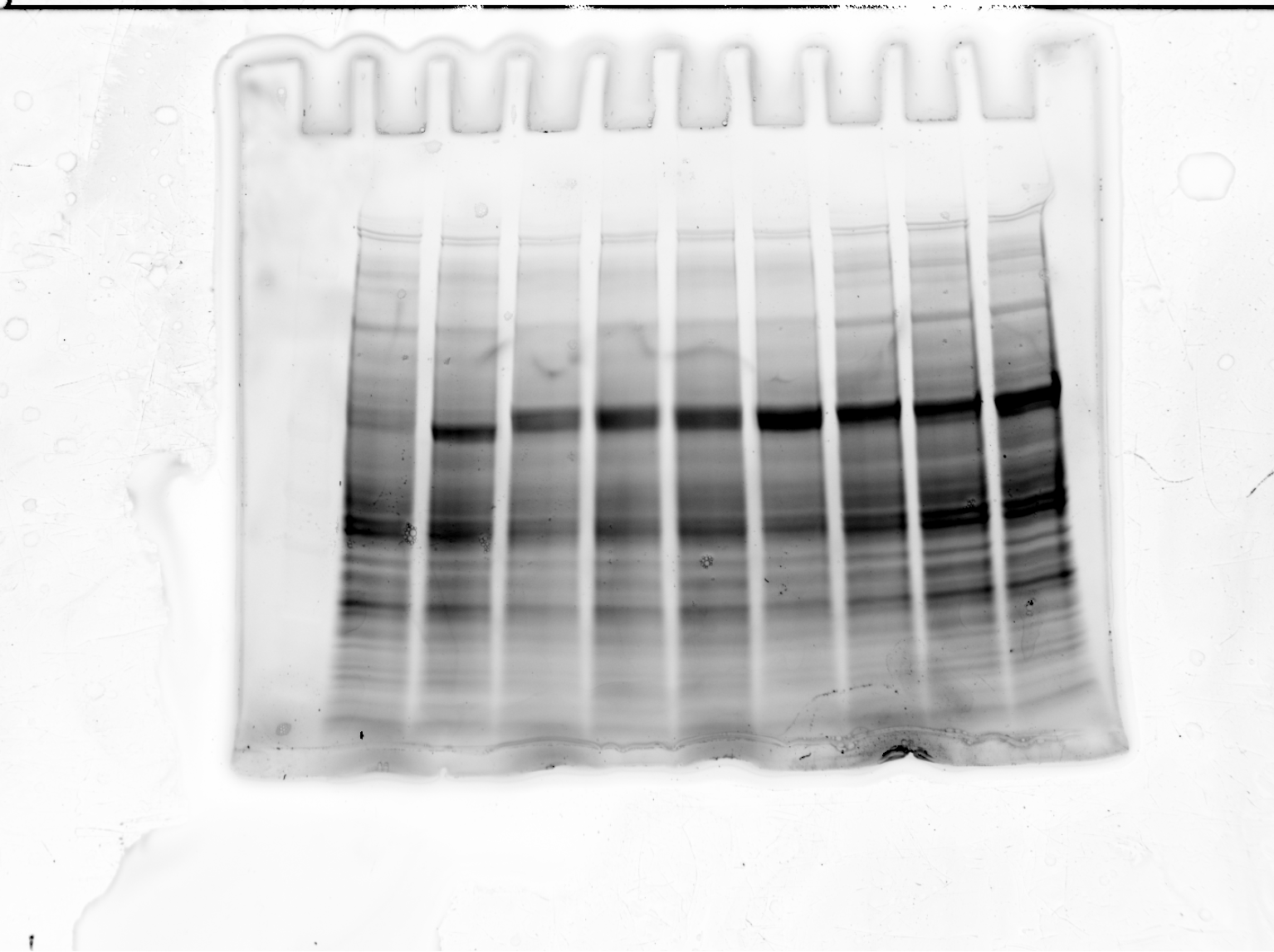

Supplement: Figure 4—figure supplement 2—source data 1. [file elife-79183-fig4-figsupp2-data1.zip › Figure 4-figure supplement 2-souce data1/Figure 4-figure supplement 2B-souce data1/Figure 4-figure supplement 2B-souce data1-rawBRC panel TCL.tif]

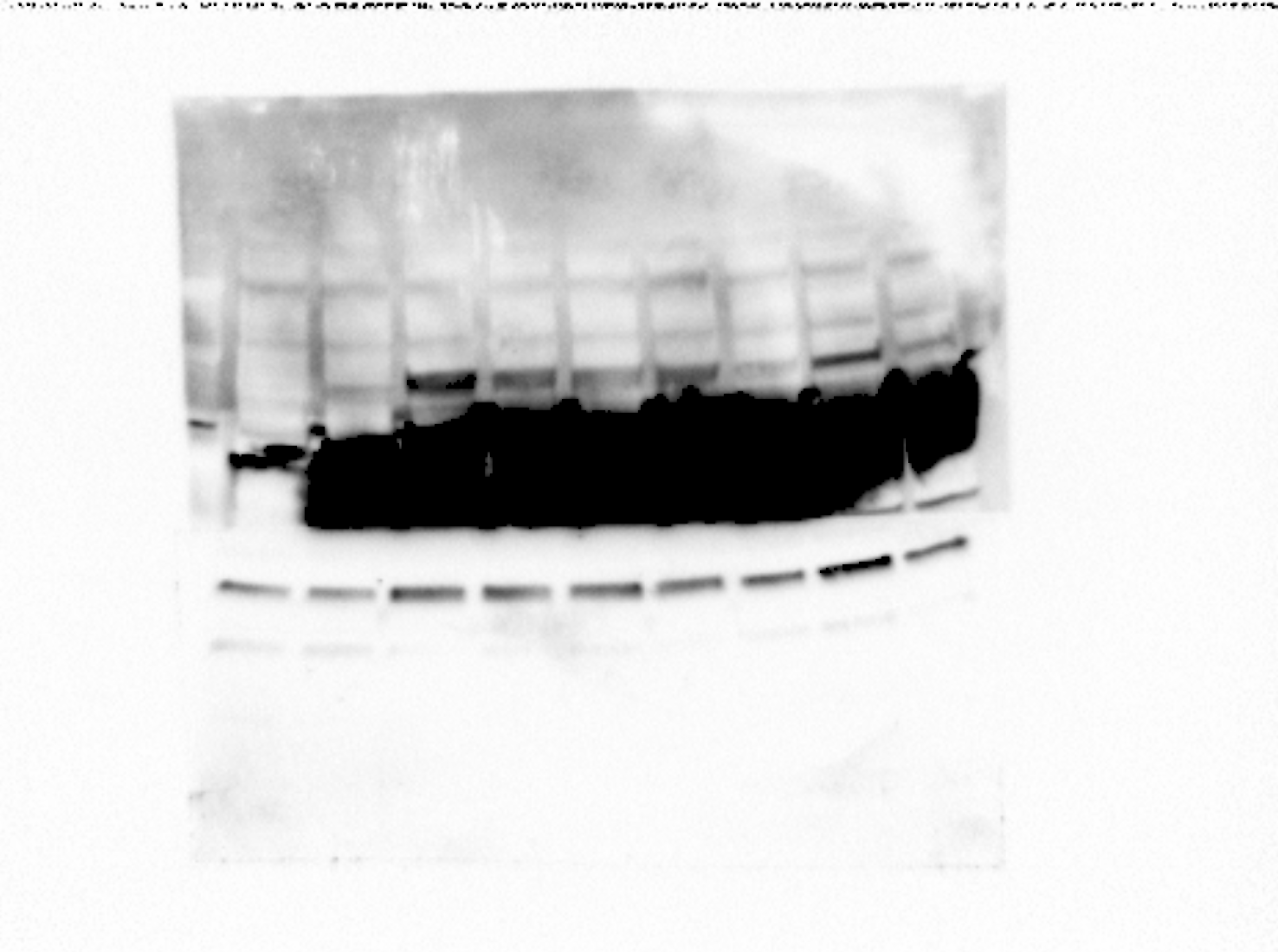

Supplement: Figure 4—figure supplement 2—source data 1. [file elife-79183-fig4-figsupp2-data1.zip › Figure 4-figure supplement 2-souce data1/Figure 4-figure supplement 2B-souce data1/Figure 4-figure supplement 2B-souce data2-rawRAD51panel TCL_Exposure_7.0sec.tif]

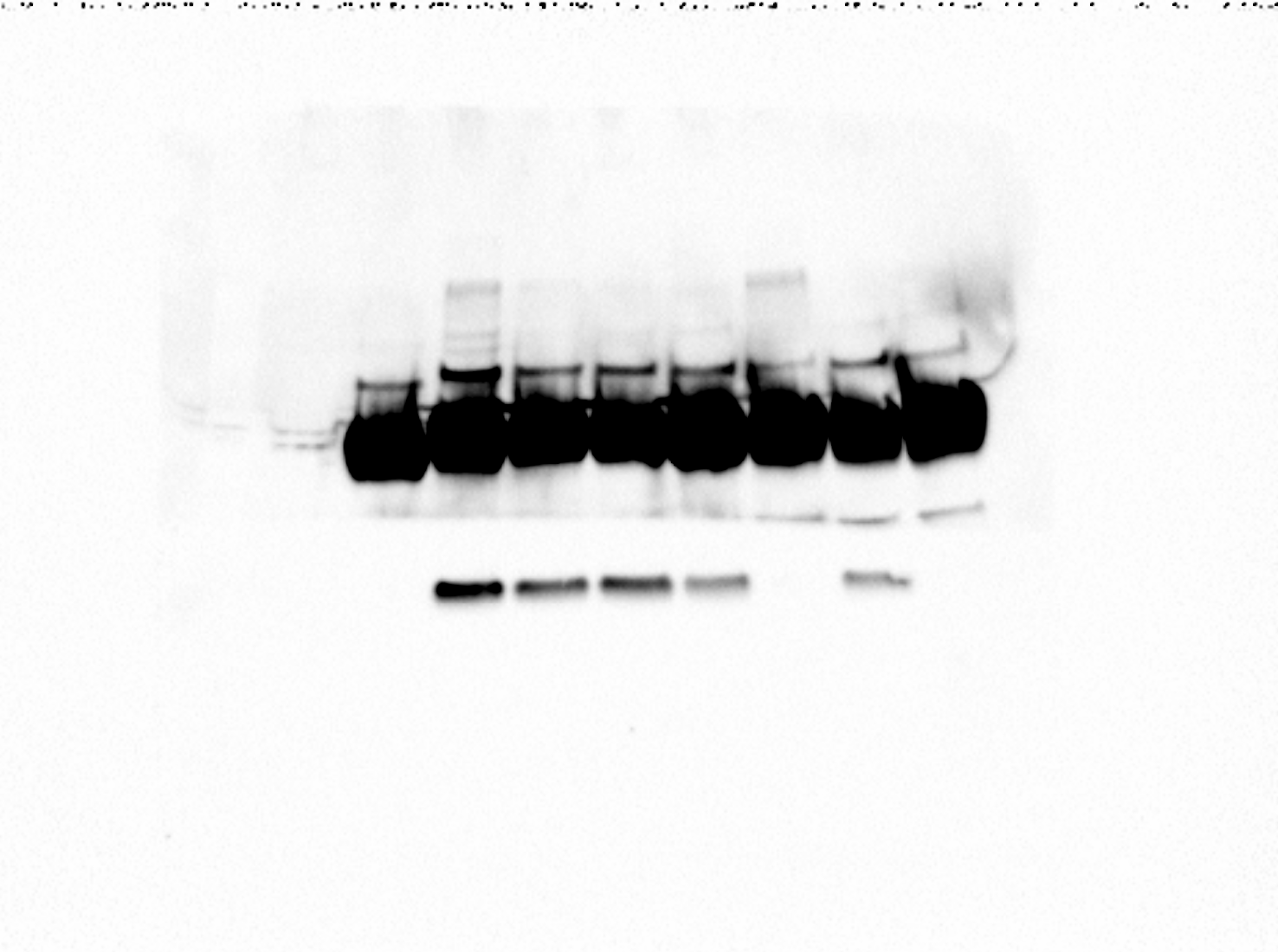

Supplement: Figure 4—figure supplement 2—source data 1. [file elife-79183-fig4-figsupp2-data1.zip › Figure 4-figure supplement 2-souce data1/Figure 4-figure supplement 2B-souce data1/Figure 4-figure supplement 2B-souce data3-rawRAD51 panel PD_Exposure_5.0sec.tif]

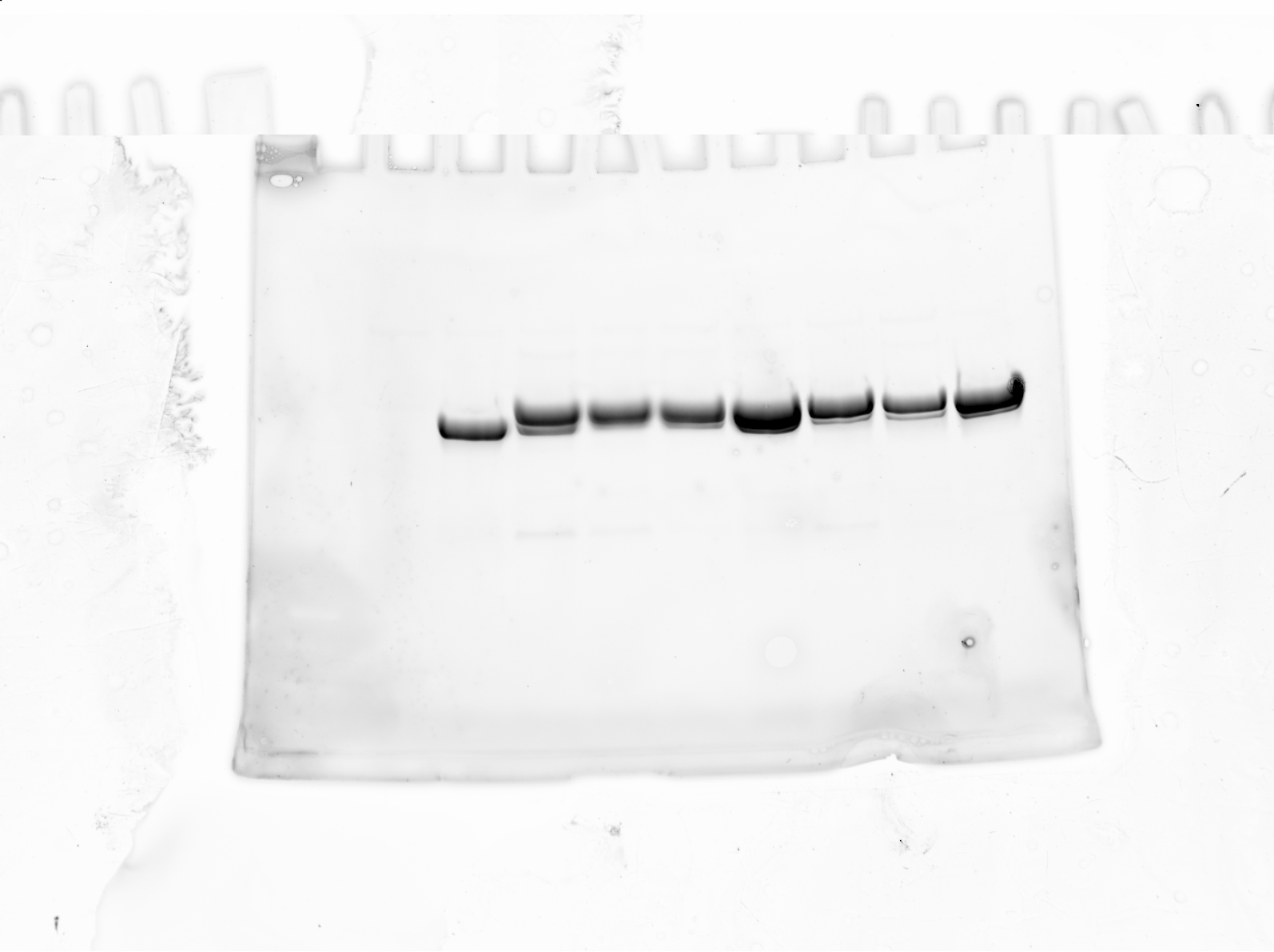

Supplement: Figure 4—figure supplement 2—source data 1. [file elife-79183-fig4-figsupp2-data1.zip › Figure 4-figure supplement 2-souce data1/Figure 4-figure supplement 2B-souce data1/Figure 4-figure supplement 2B-souce data4-rawBRC panel PD.tif]

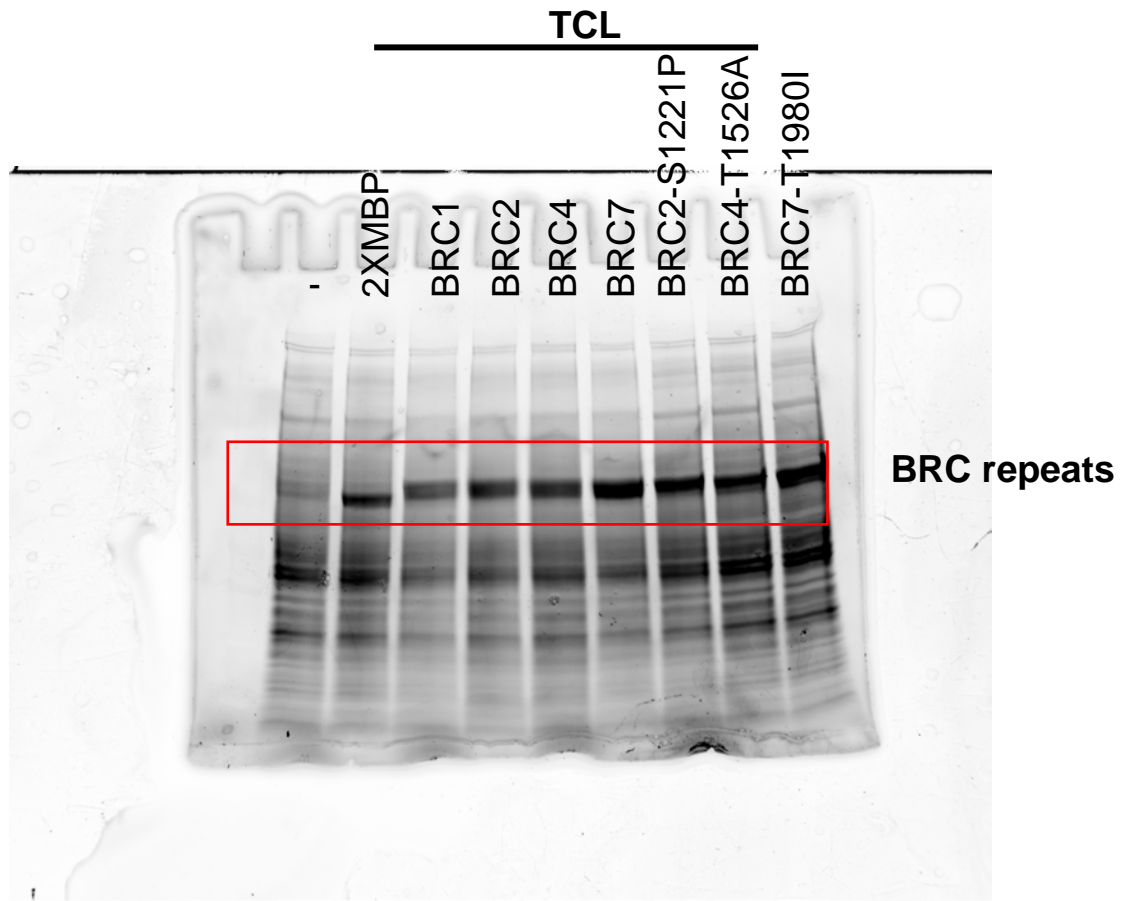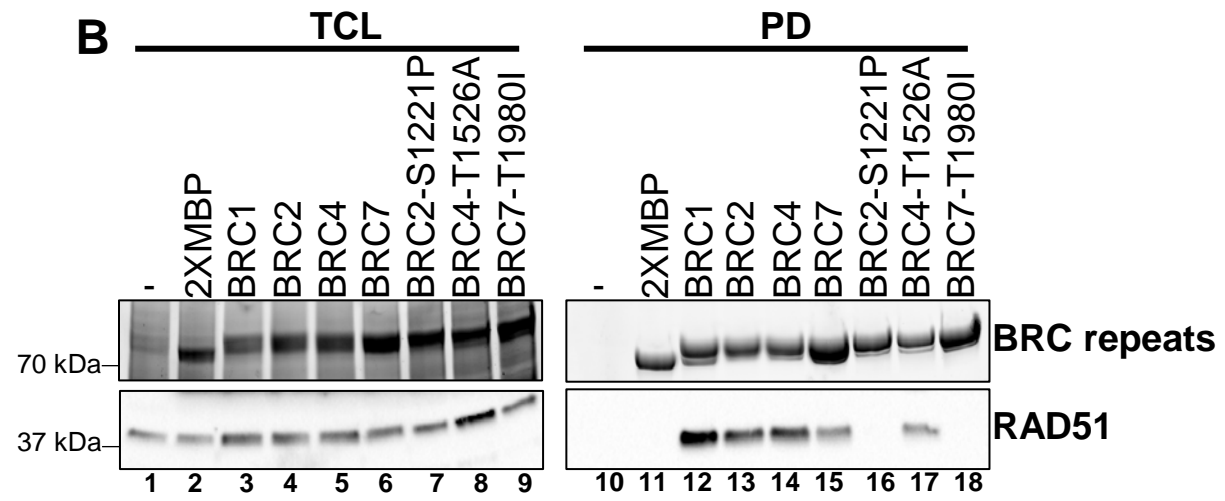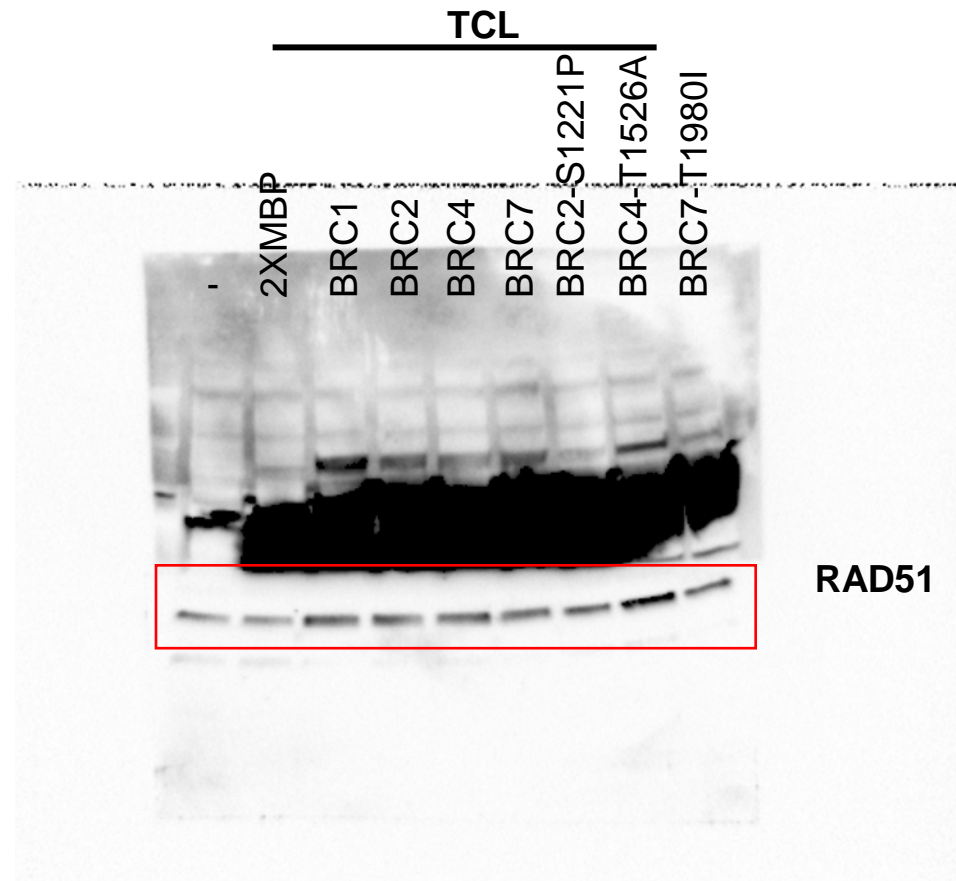

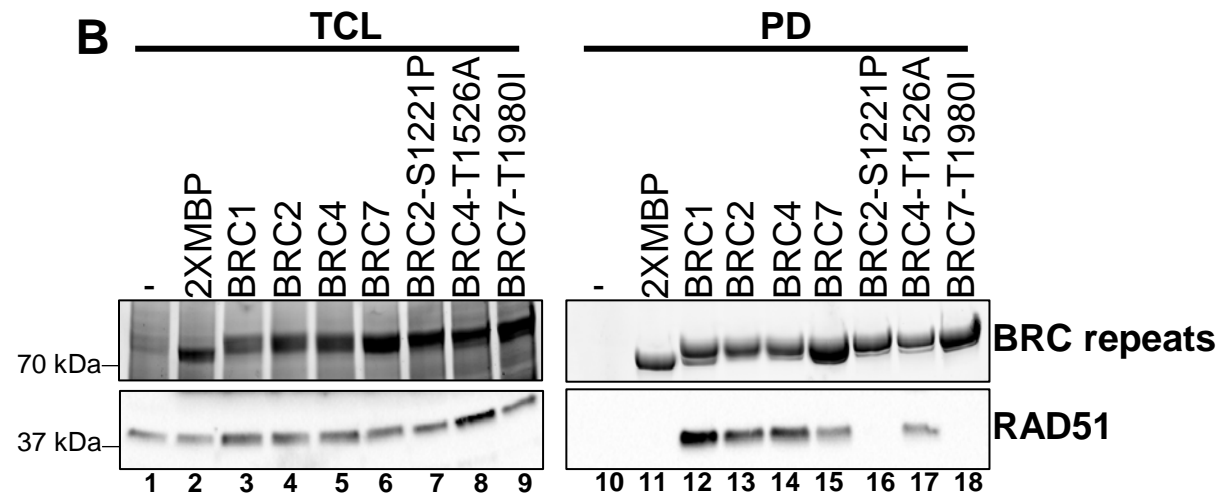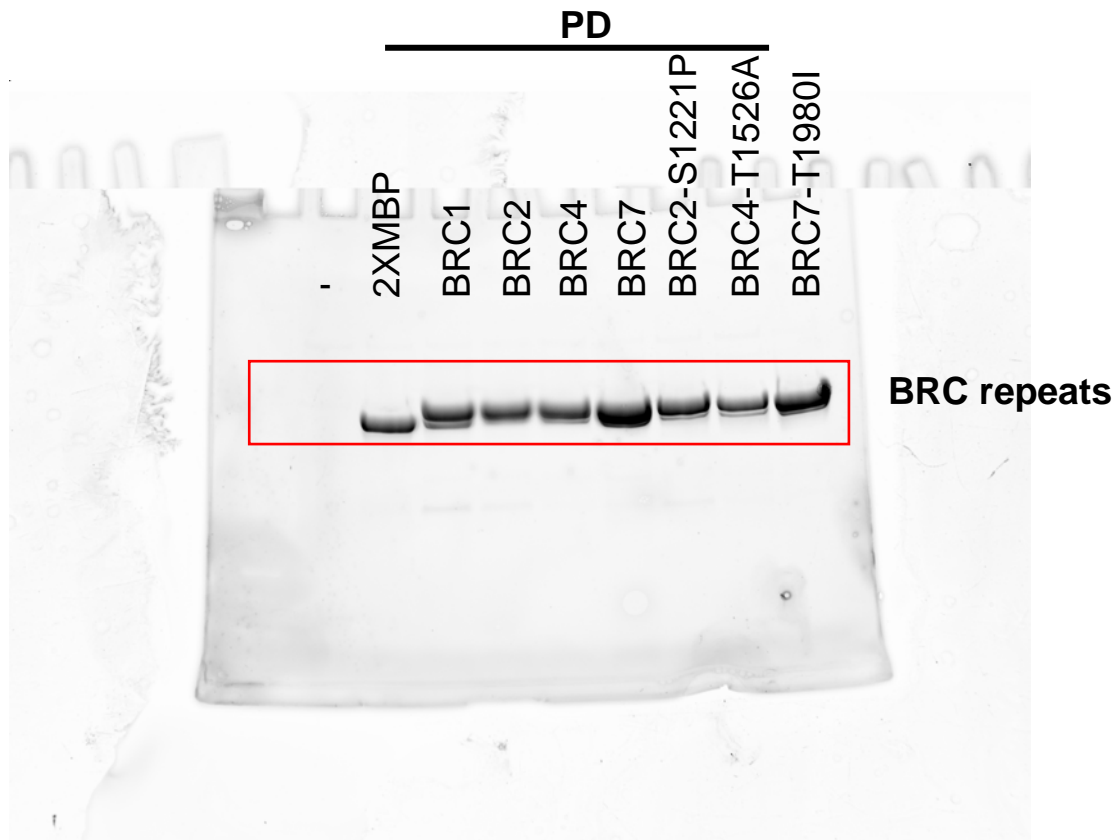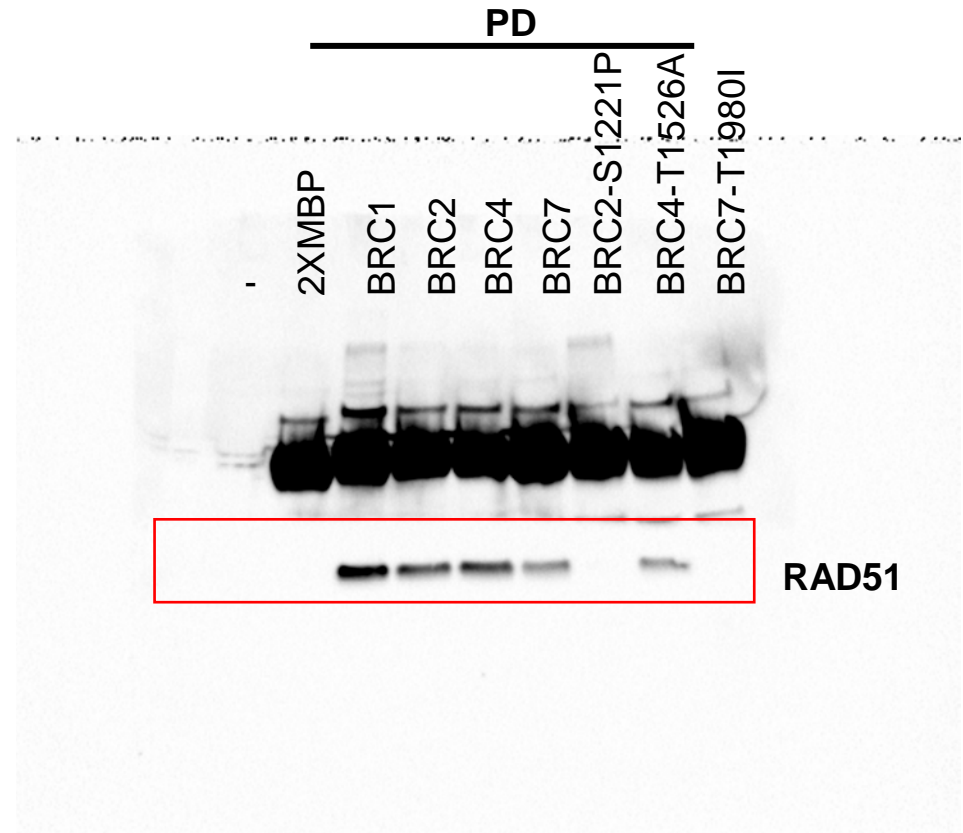

Supplement: Figure 4—figure supplement 2—source data 1. [file elife-79183-fig4-figsupp2-data1.zip › Figure 4-figure supplement 2-souce data1/Figure 4-figure supplement 2B-souce data1/Figure 4-figure supplement 2B-souce data5-highlightedbandsandlabeled.pdf]

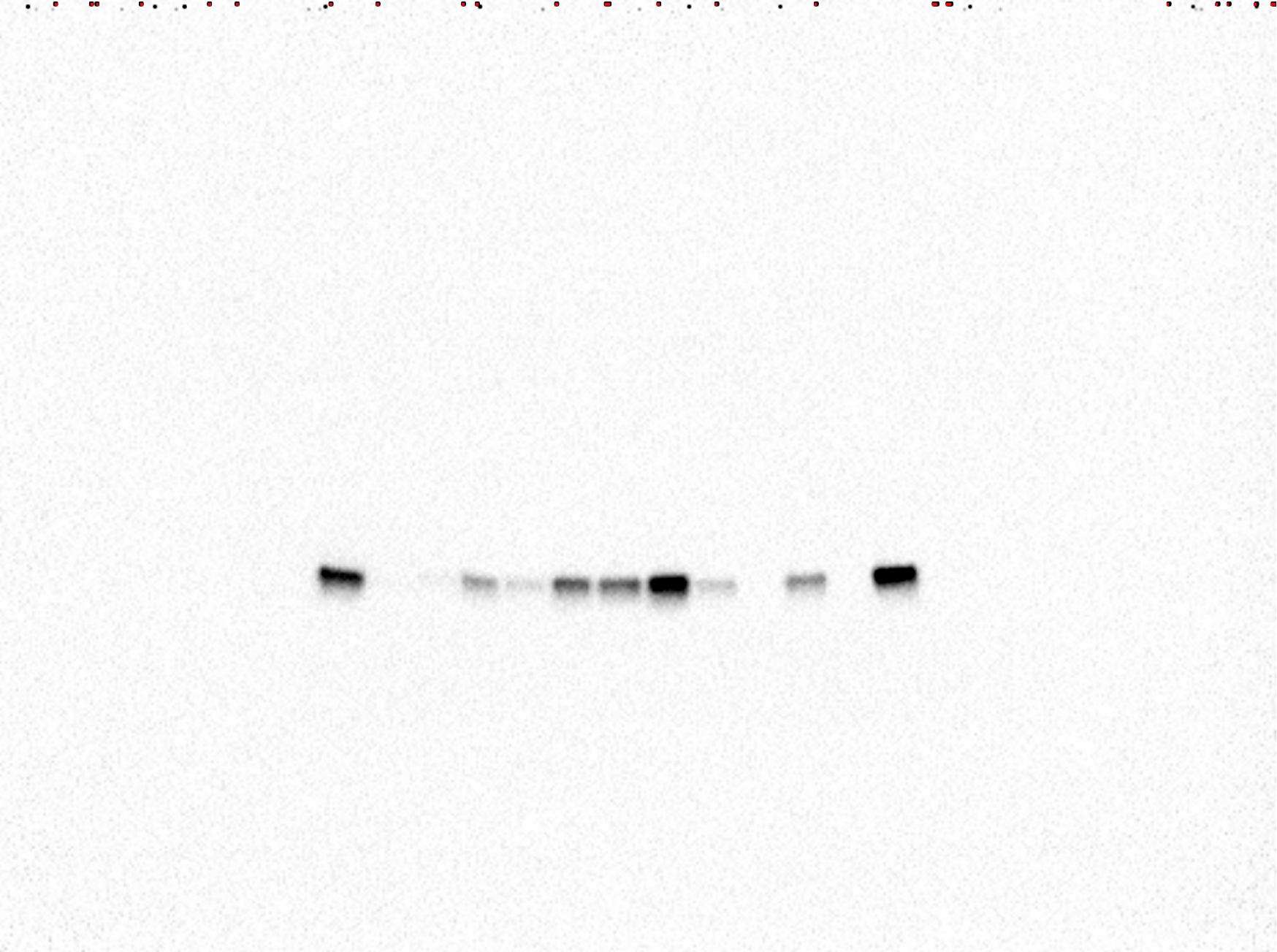

Supplement: Figure 4—figure supplement 2—source data 1. [file elife-79183-fig4-figsupp2-data1.zip › Figure 4-figure supplement 2-souce data1/Figure 4-figure supplement 2C-souce data1/Figure 4-figure supplement 2C-souce data1-RAD51 BRC2 panel.tif]

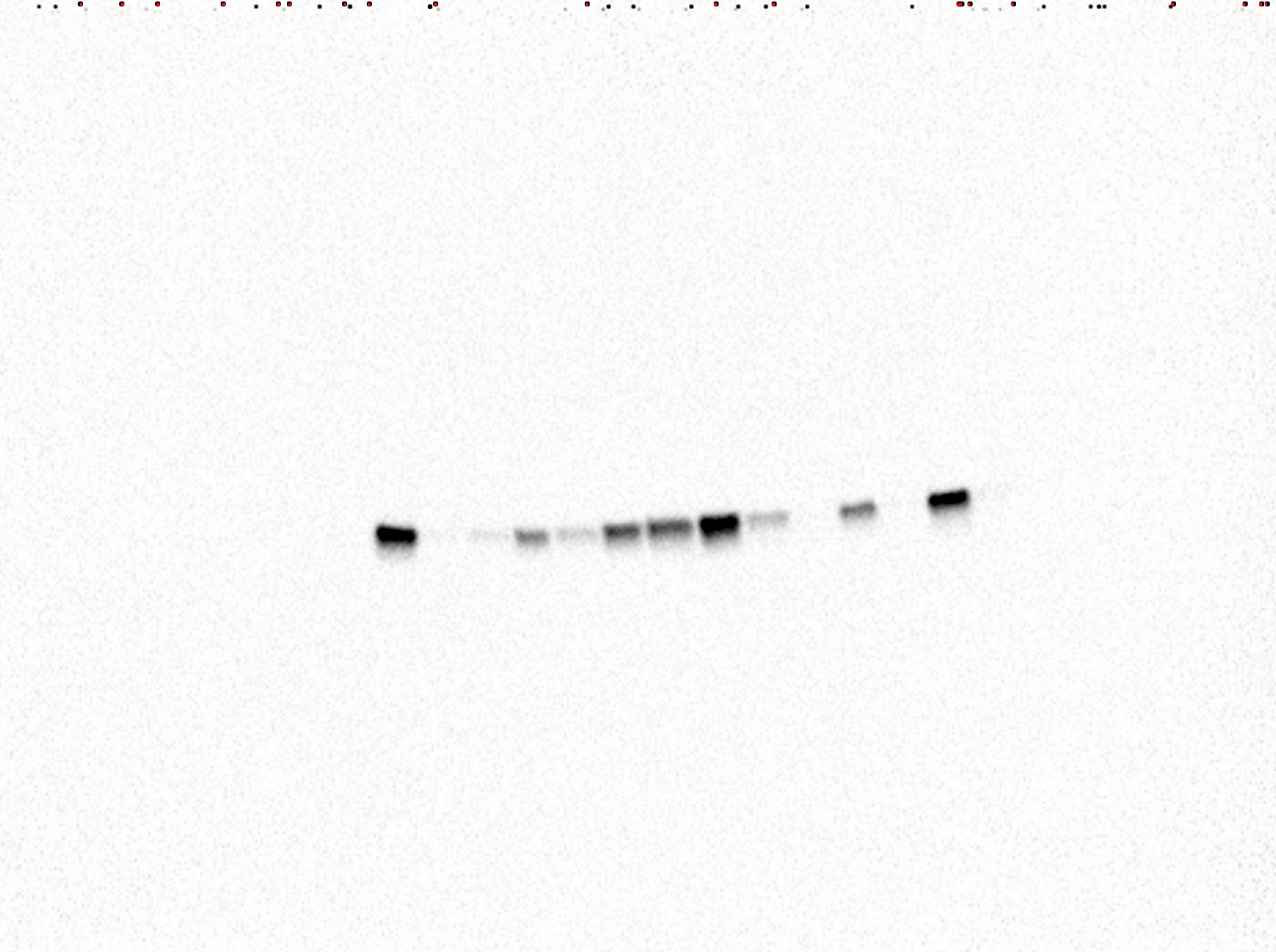

Supplement: Figure 4—figure supplement 2—source data 1. [file elife-79183-fig4-figsupp2-data1.zip › Figure 4-figure supplement 2-souce data1/Figure 4-figure supplement 2C-souce data1/Figure 4-figure supplement 2C-souce data2-RAD51 BRC7 panel.tif]

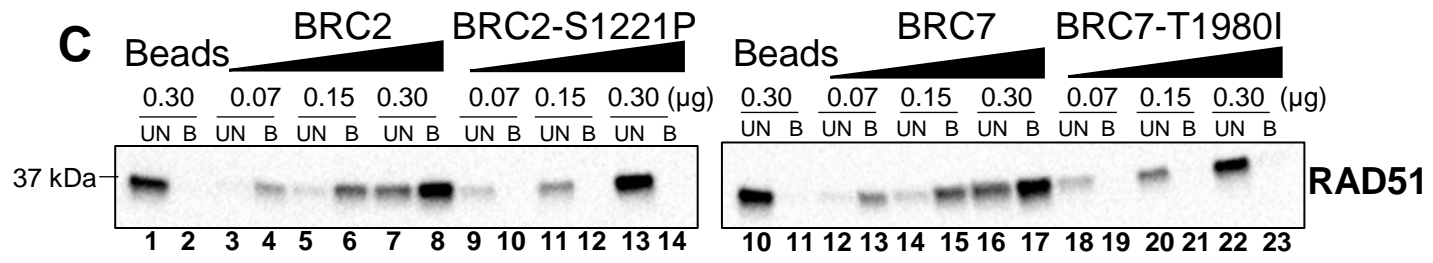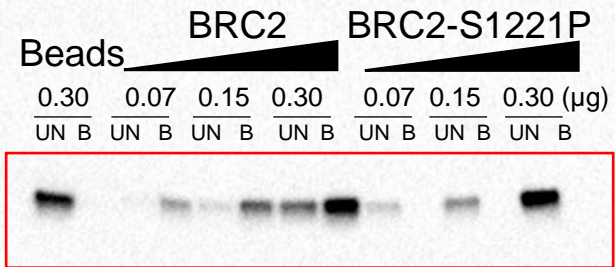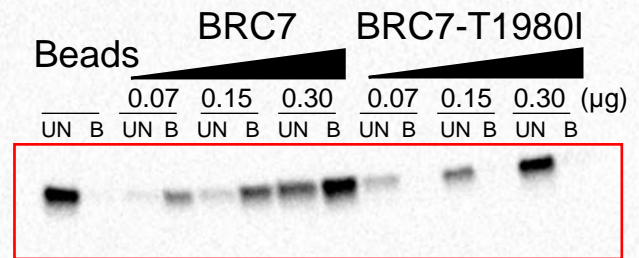

Supplement: Figure 4—figure supplement 2—source data 1. [file elife-79183-fig4-figsupp2-data1.zip › Figure 4-figure supplement 2-souce data1/Figure 4-figure supplement 2C-souce data1/Figure 4-figure supplement 2C-souce data3-highlightedbandsandlabeled.pdf]

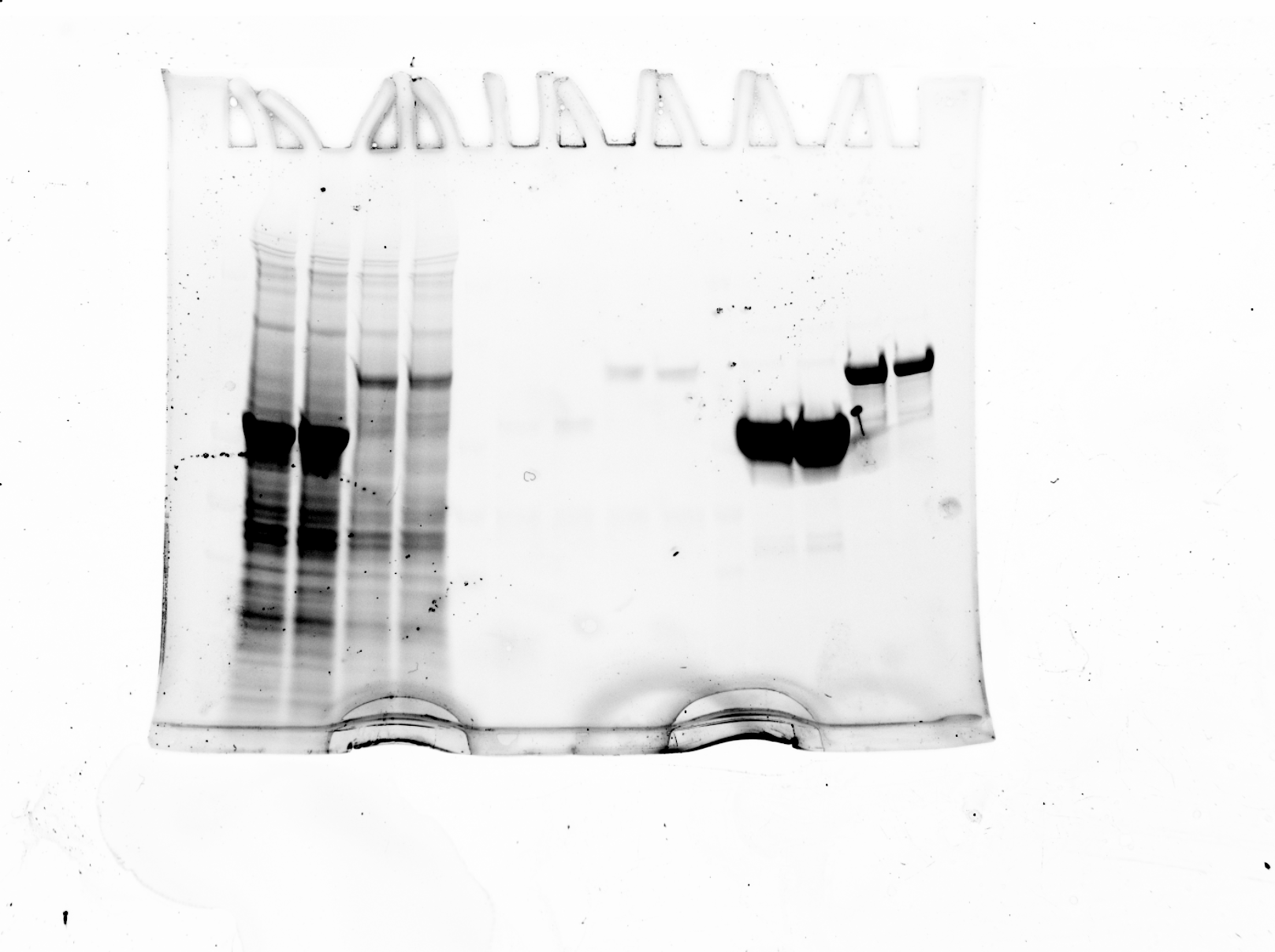

Supplement: Figure 4—figure supplement 2—source data 1. [file elife-79183-fig4-figsupp2-data1.zip › Figure 4-figure supplement 2-souce data1/Figure 4-figure supplement 2D-souce data1/Figure 4-figure supplement 2D-souce data1- TCL StainFree Panel.tif]

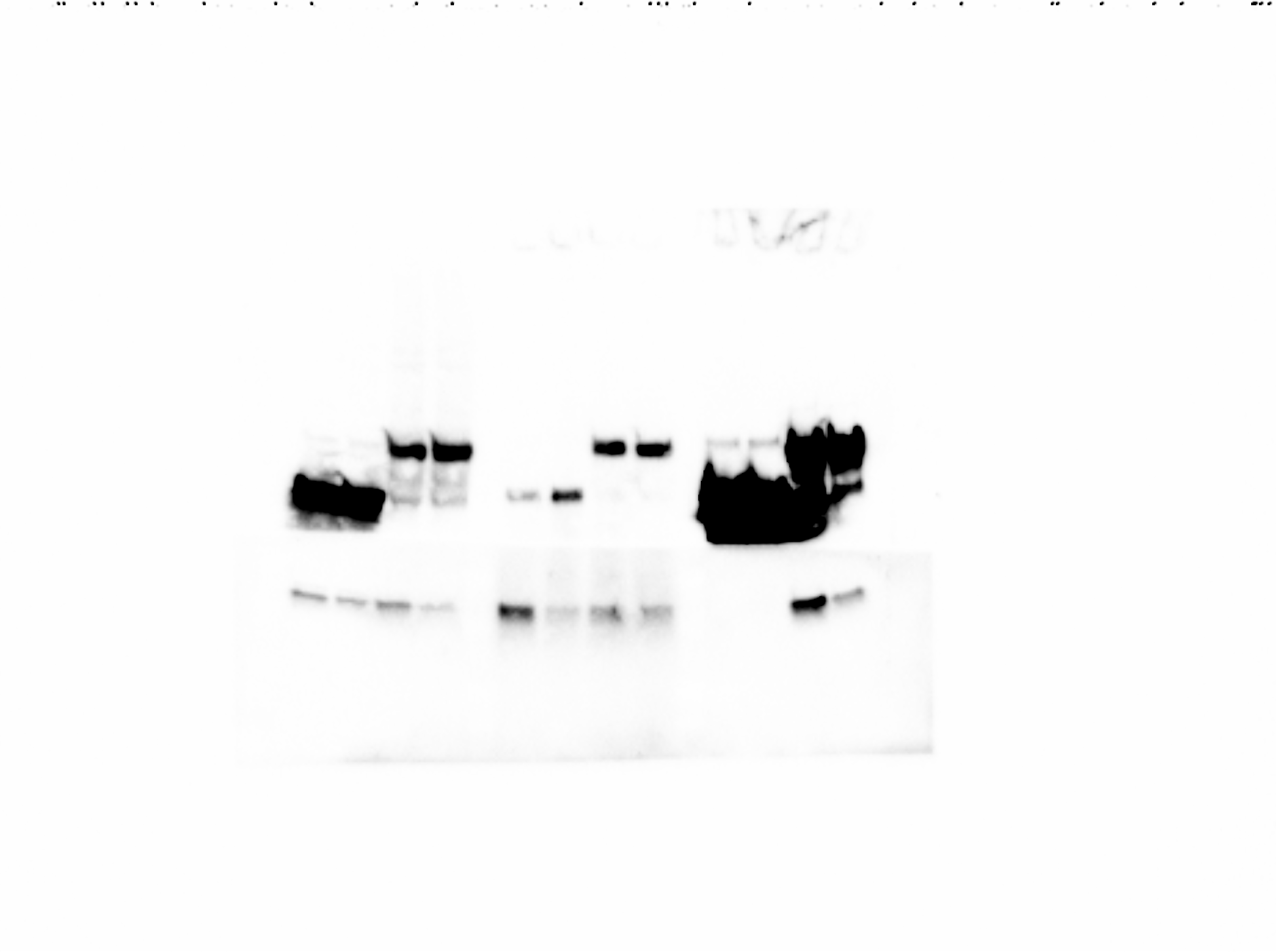

Supplement: Figure 4—figure supplement 2—source data 1. [file elife-79183-fig4-figsupp2-data1.zip › Figure 4-figure supplement 2-souce data1/Figure 4-figure supplement 2D-souce data1/Figure 4-figure supplement 2D-souce data2- TCL RAD51 Panel_Exposure_4.0sec.tif]

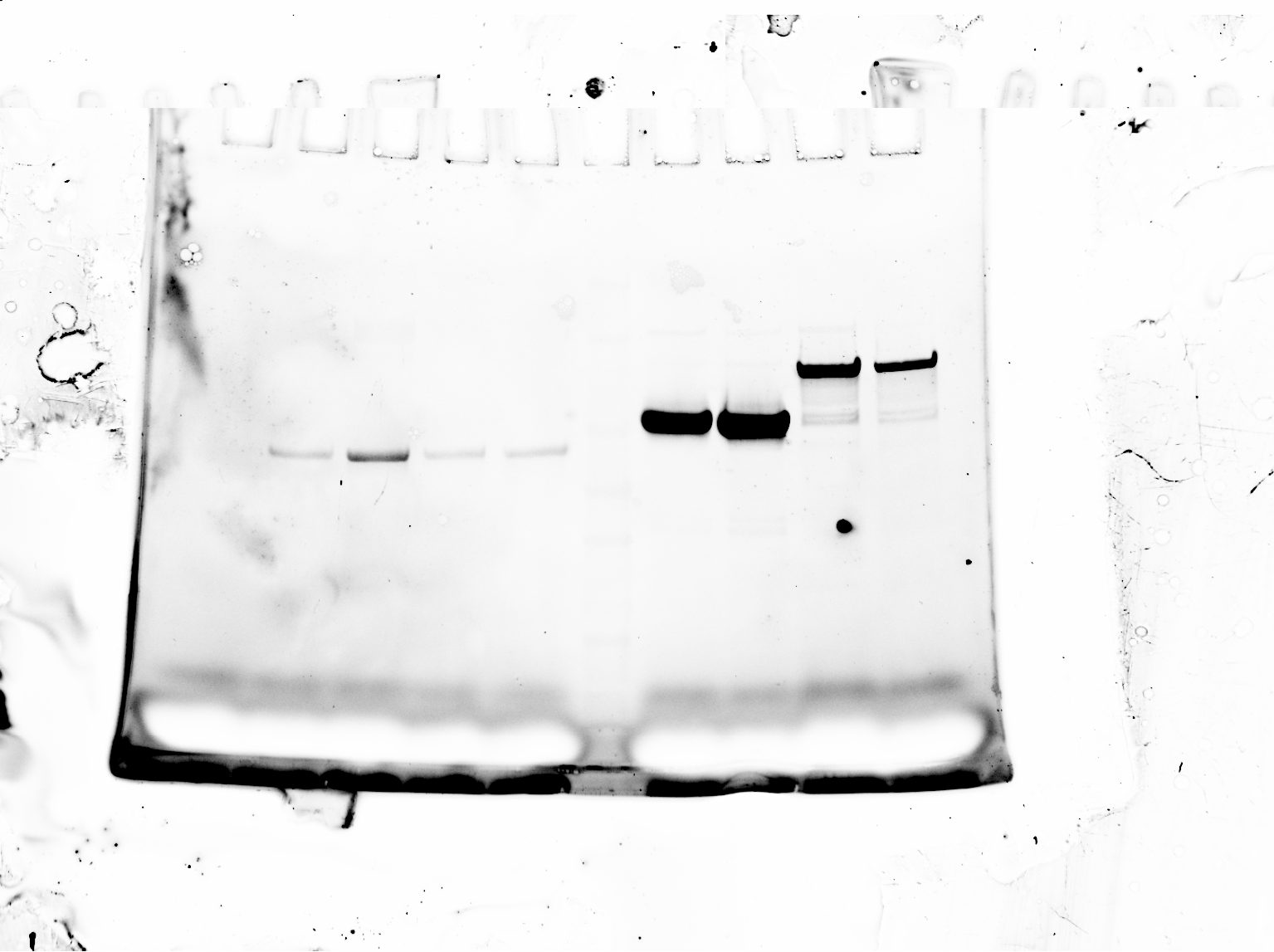

Supplement: Figure 4—figure supplement 2—source data 1. [file elife-79183-fig4-figsupp2-data1.zip › Figure 4-figure supplement 2-souce data1/Figure 4-figure supplement 2D-souce data1/Figure 4-figure supplement 2D-souce data3- PD StainFree Panel.tif]

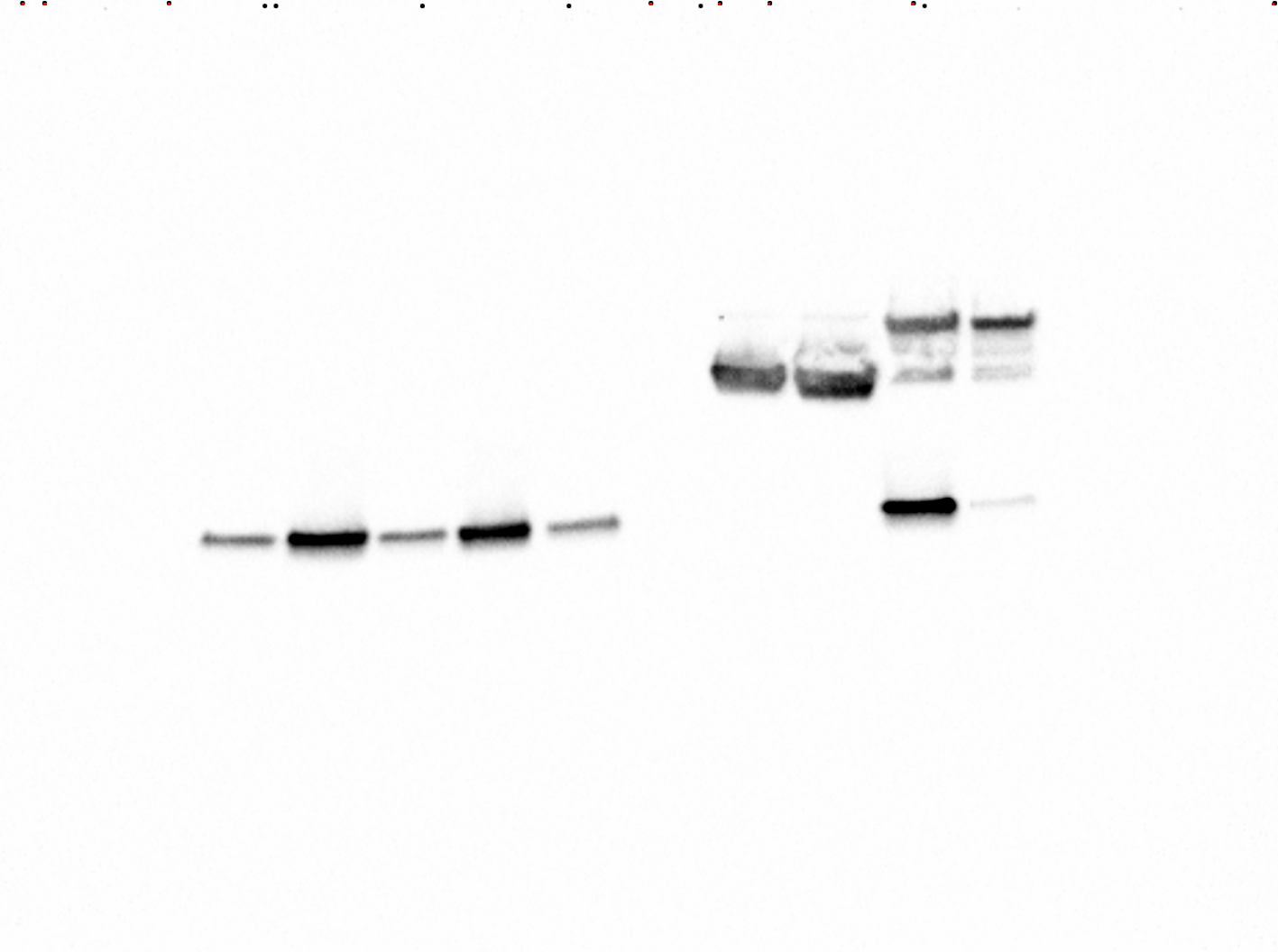

Supplement: Figure 4—figure supplement 2—source data 1. [file elife-79183-fig4-figsupp2-data1.zip › Figure 4-figure supplement 2-souce data1/Figure 4-figure supplement 2D-souce data1/Figure 4-figure supplement 2D-souce data4- PD RAD51 Panel_Exposure_2.0sec.tif]

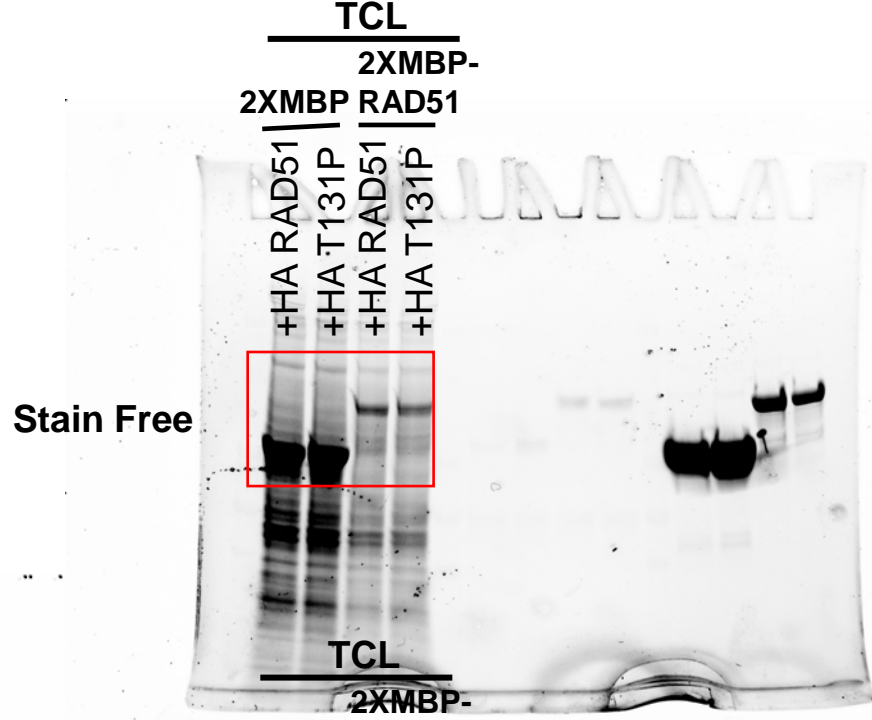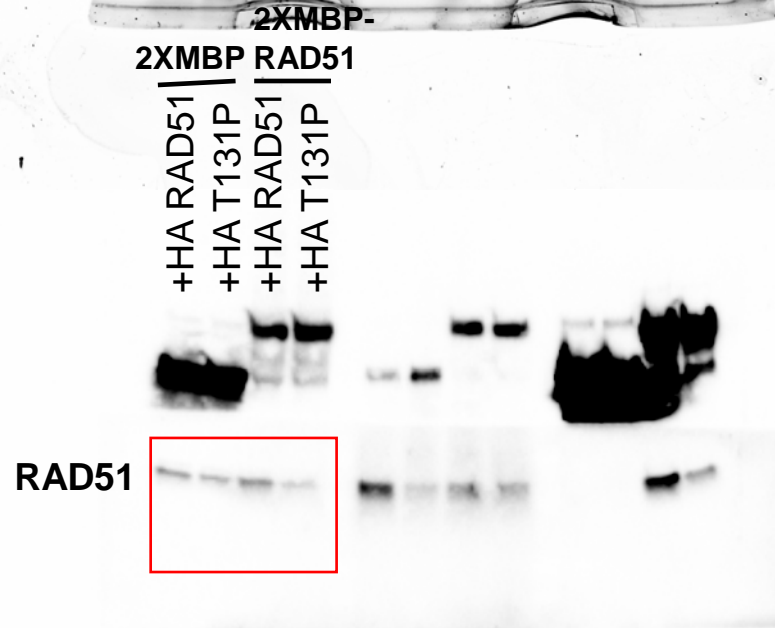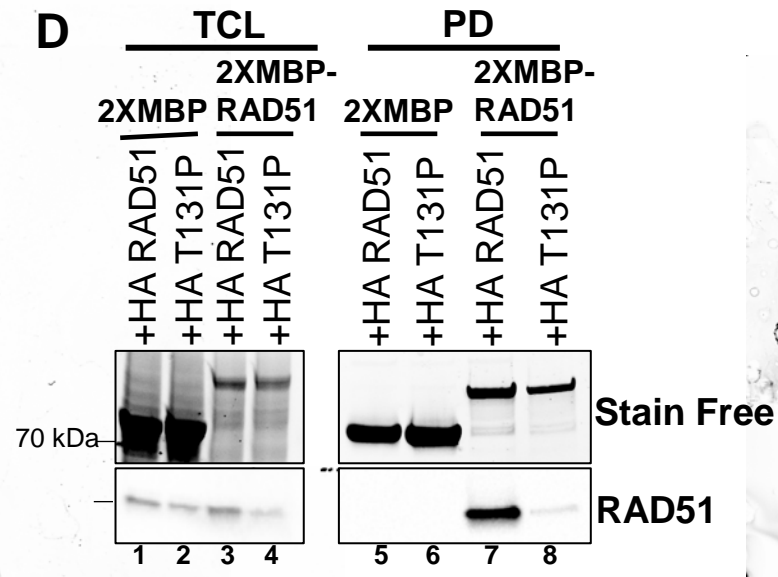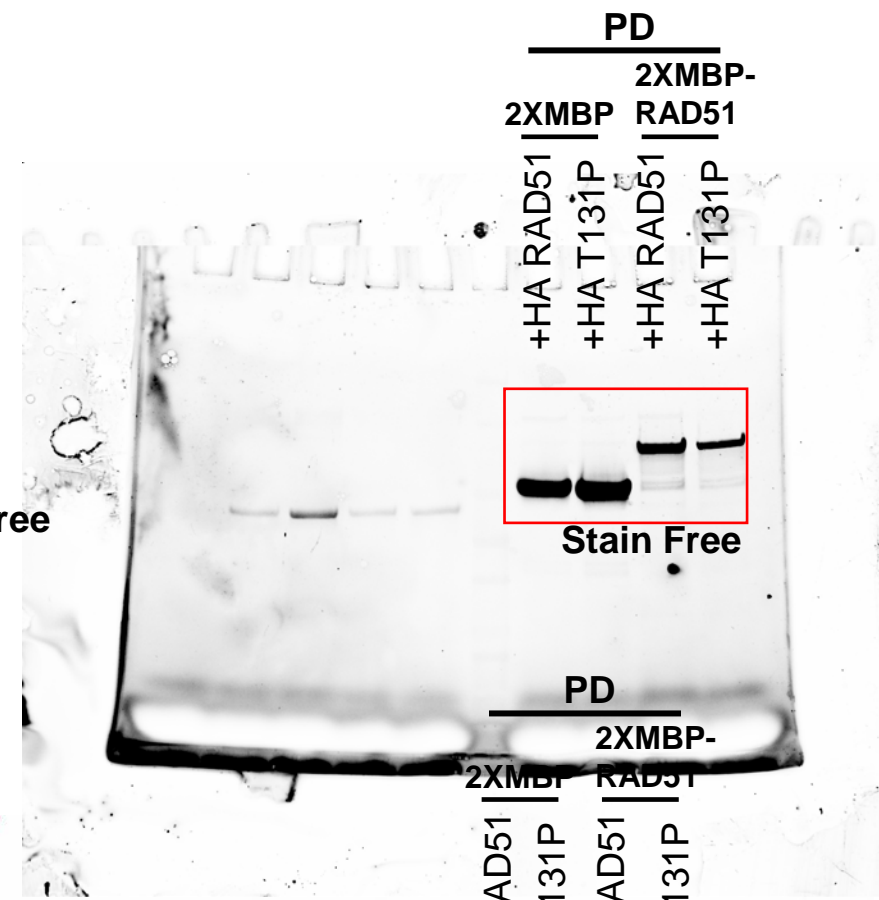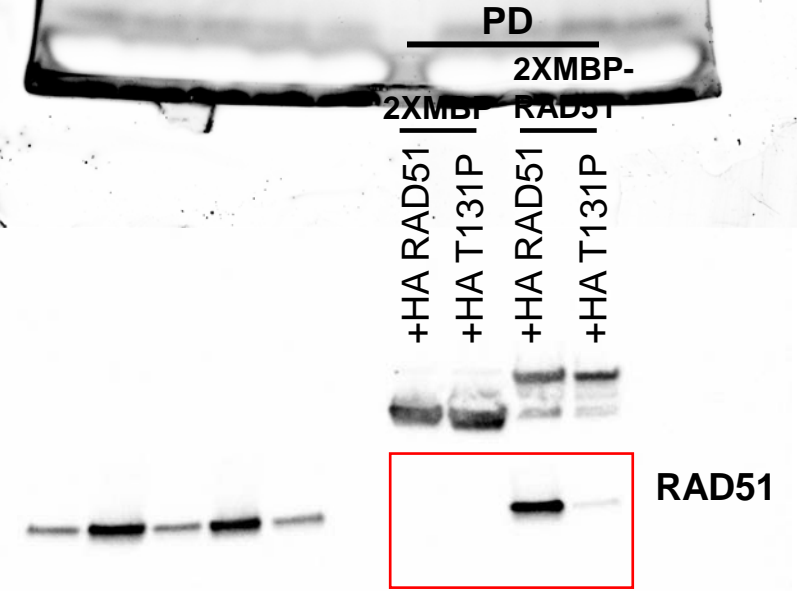

Supplement: Figure 4—figure supplement 2—source data 1. [file elife-79183-fig4-figsupp2-data1.zip › Figure 4-figure supplement 2-souce data1/Figure 4-figure supplement 2D-souce data1/Figure 4-figure supplement 2D-souce data5-highlightedbandsandlabeled.pdf]

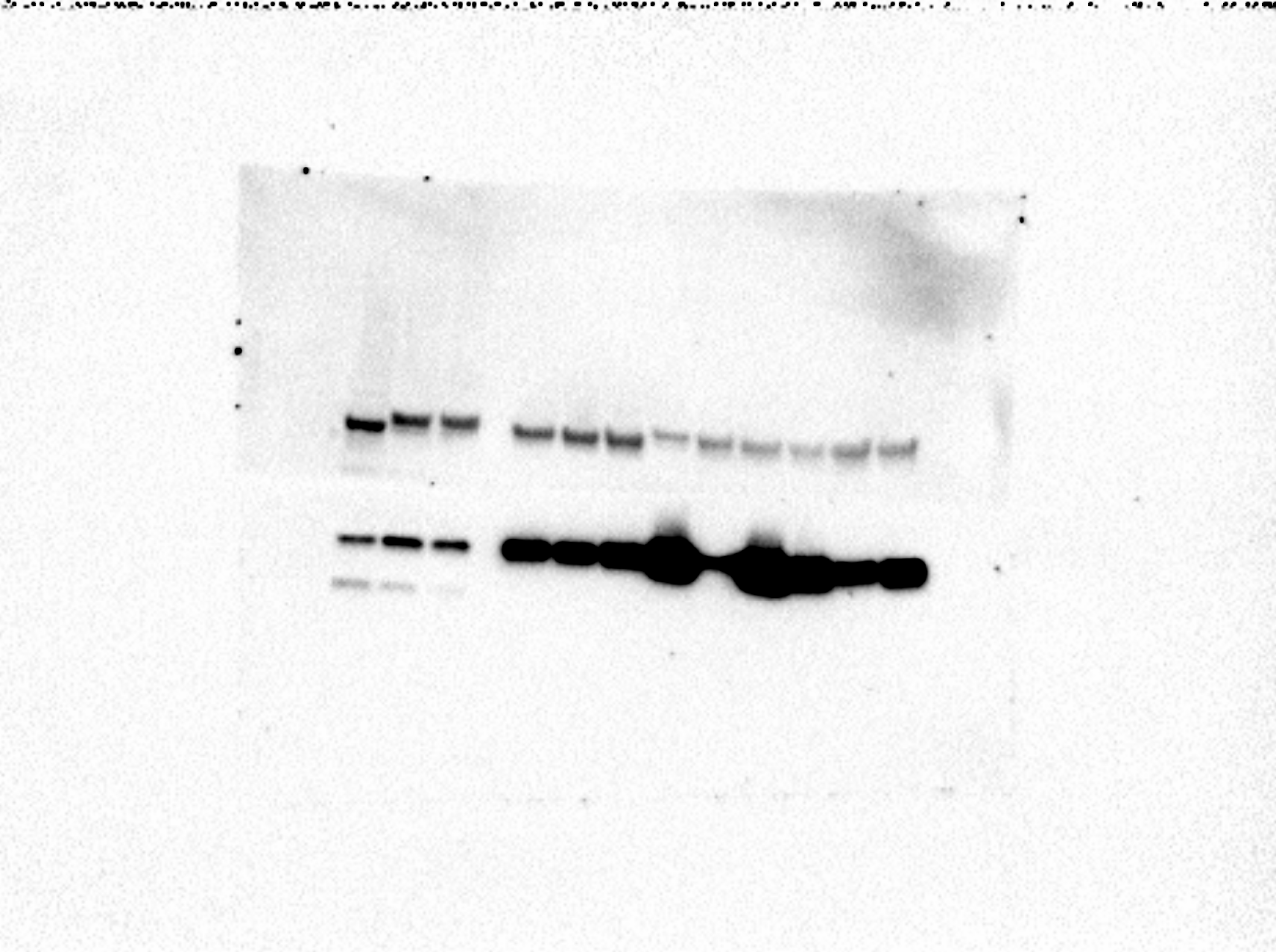

Supplement: Figure 4—figure supplement 2—source data 1. [file elife-79183-fig4-figsupp2-data1.zip › Figure 4-figure supplement 2-souce data1/Figure 4-figure supplement 2E-souce data1/Figure 4-figure supplement 2E-souce data1- BRC panel raw_Exposure_10.0sec.tif]

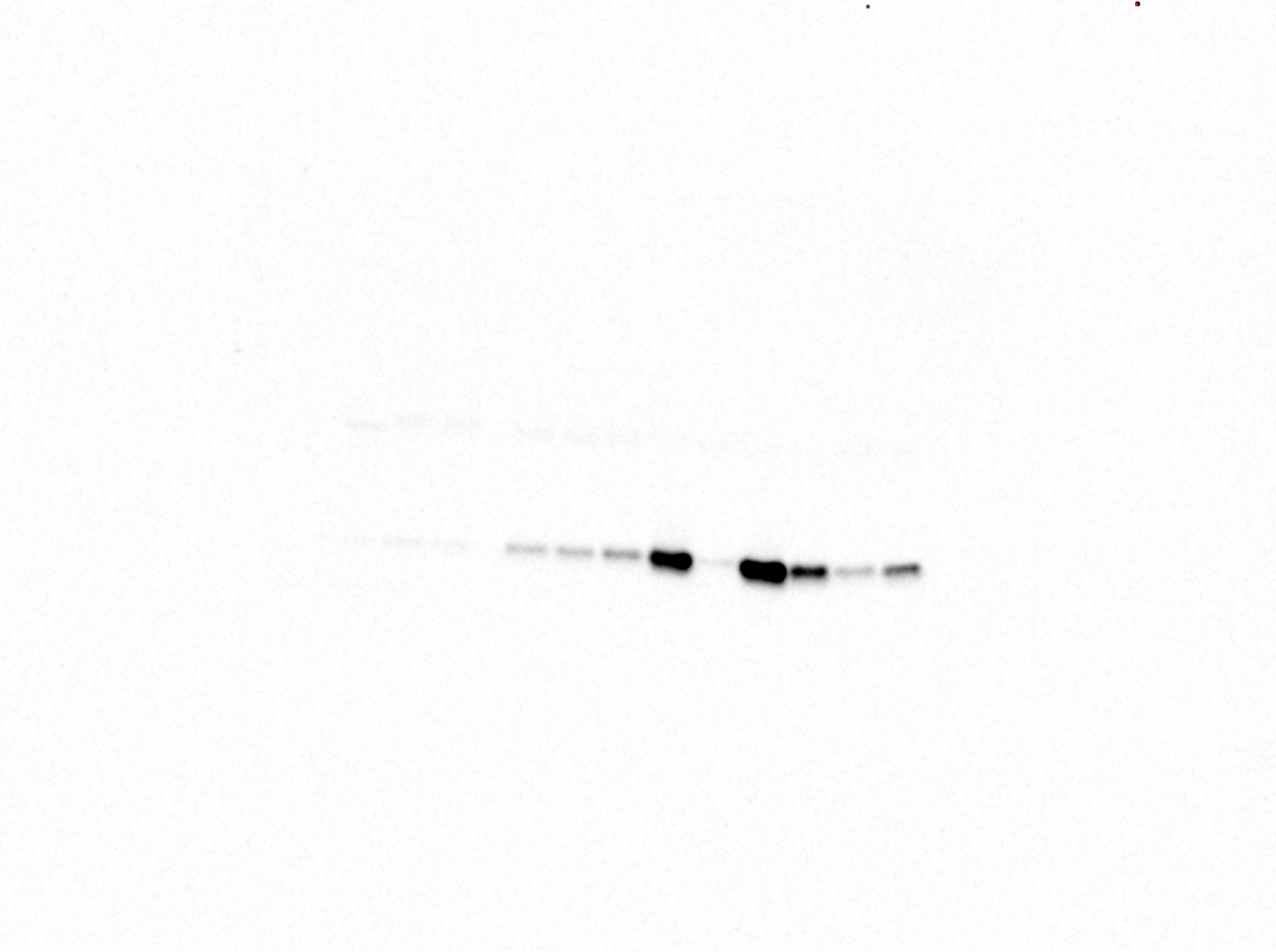

Supplement: Figure 4—figure supplement 2—source data 1. [file elife-79183-fig4-figsupp2-data1.zip › Figure 4-figure supplement 2-souce data1/Figure 4-figure supplement 2E-souce data1/Figure 4-figure supplement 2E-souce data2- RAD51 panel raw.tif]

**E**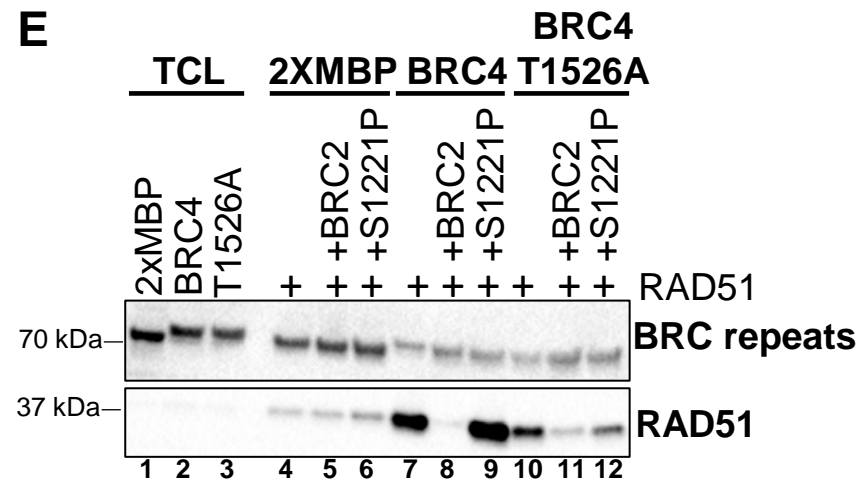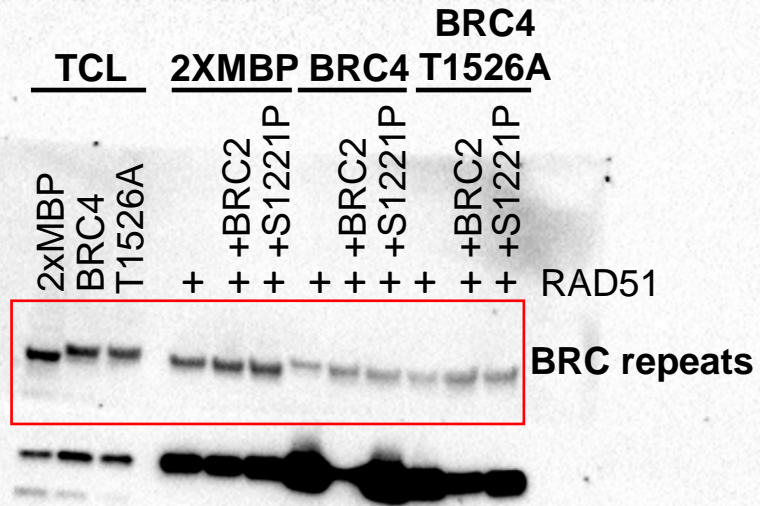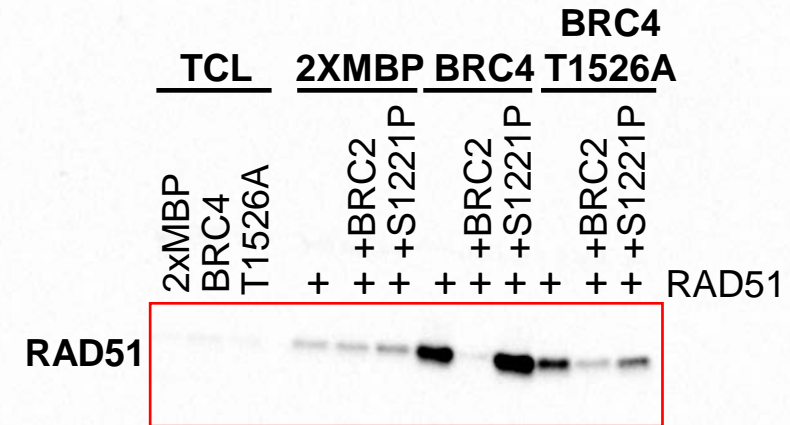

Supplement: Figure 4—figure supplement 2—source data 1. [file elife-79183-fig4-figsupp2-data1.zip › Figure 4-figure supplement 2-souce data1/Figure 4-figure supplement 2E-souce data1/Figure 4-figure supplement 2E-souce data3-highlightedbandsandlabeled.pdf]

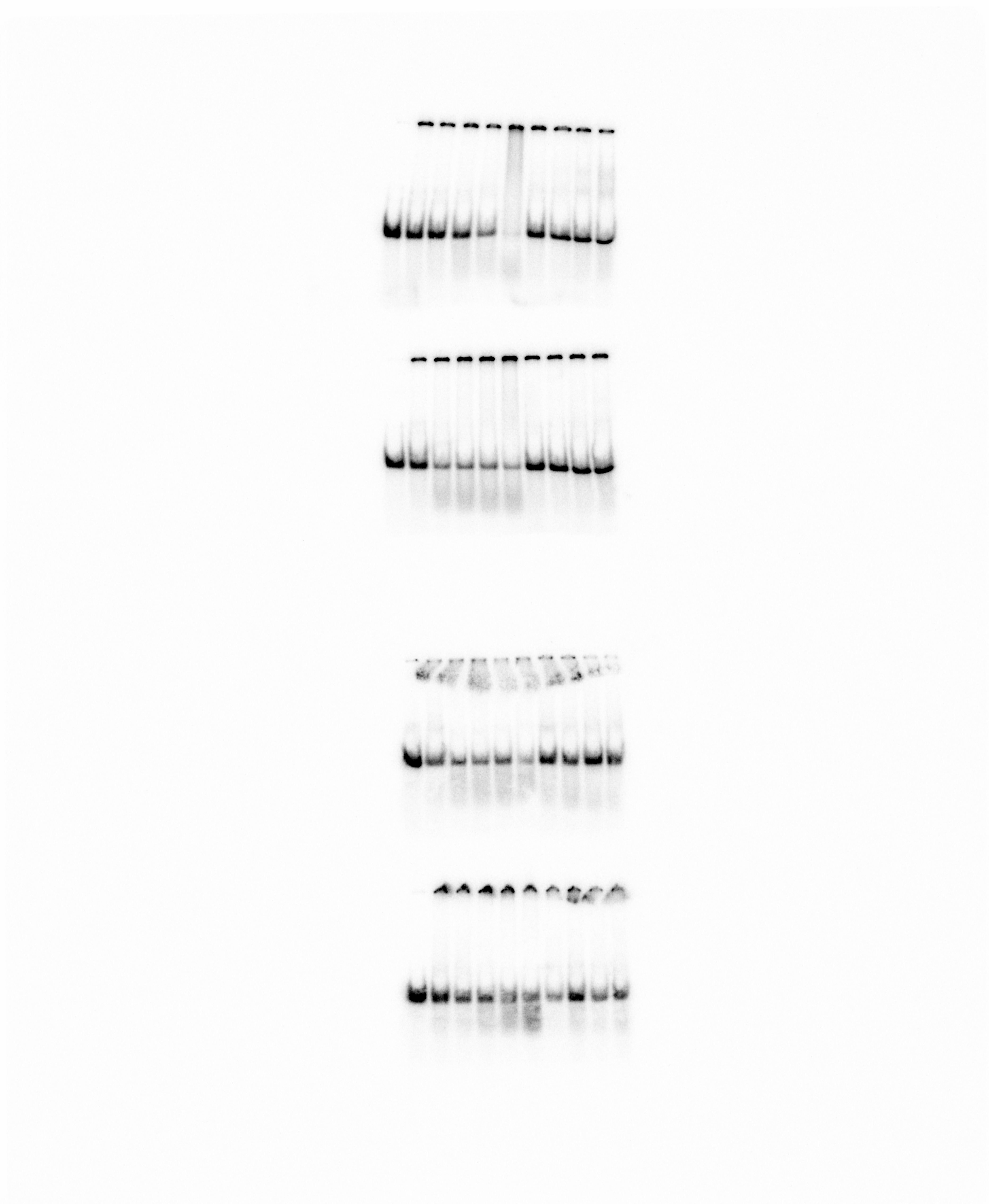

Supplement: Figure 5—source data 1. [file elife-79183-fig5-data1.zip › Figure 5-source data 1/Figure 5B-source data/Figure 5B-source data1-raw EMSA RAD51 BRC peptides.tif]

**B**

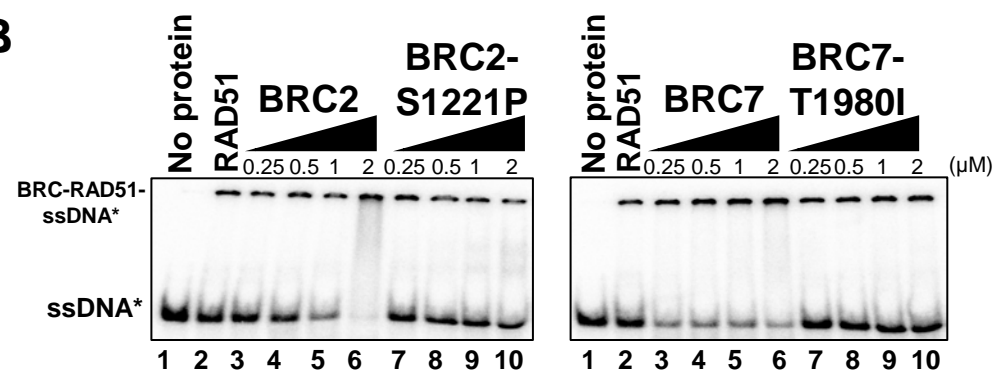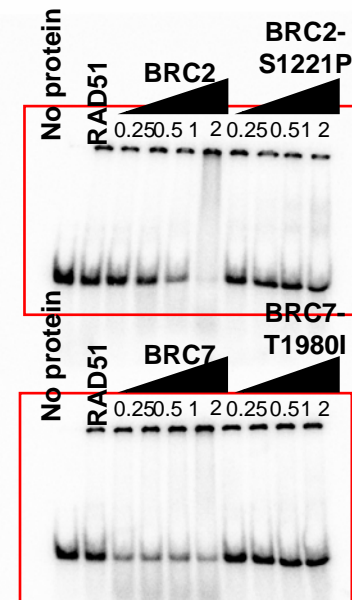

Supplement: Figure 5—source data 1. [file elife-79183-fig5-data1.zip › Figure 5-source data 1/Figure 5B-source data/Figure 5B-source data2-highlightedbandsandlabeled.pdf]

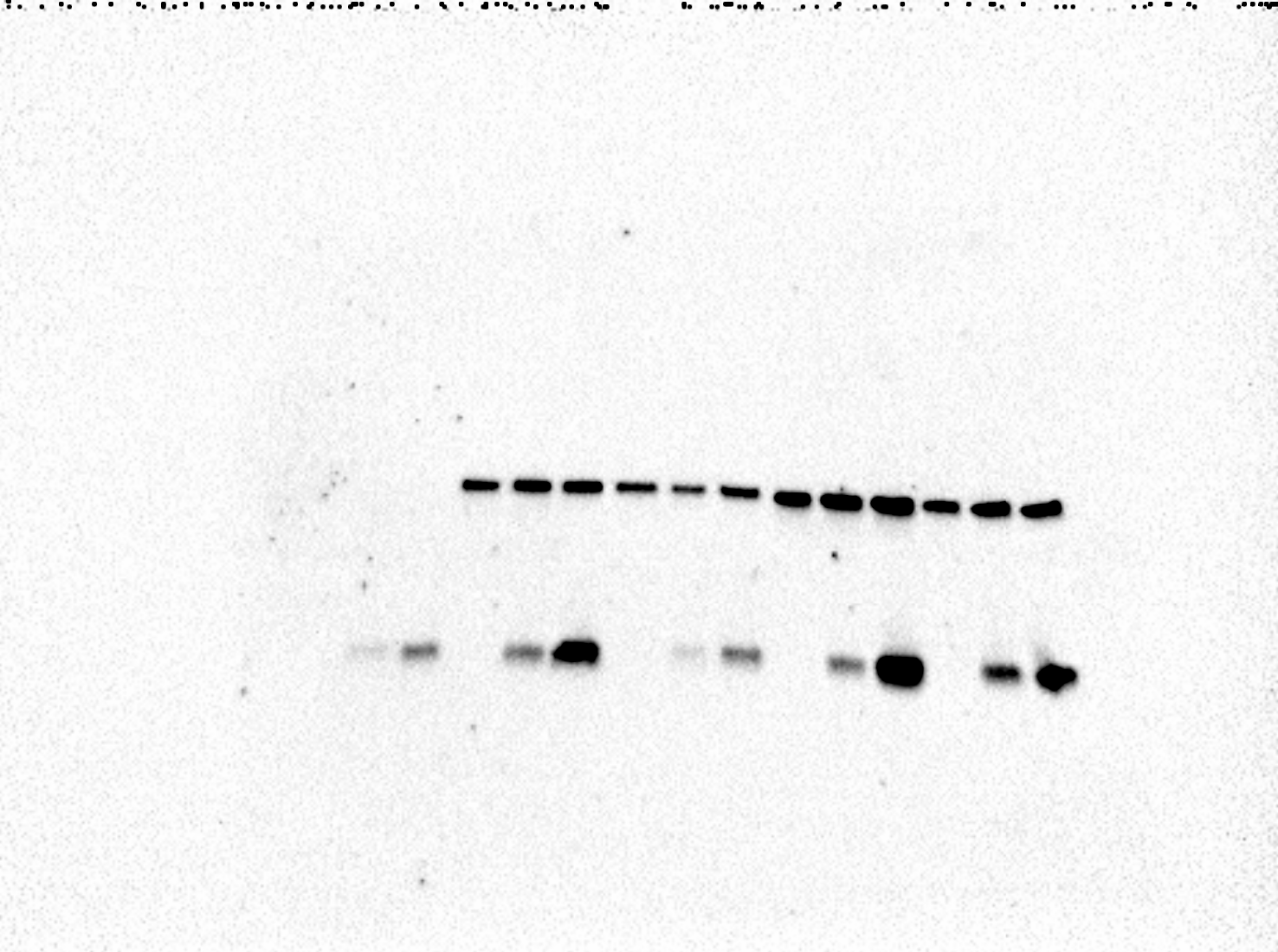

Supplement: Figure 5—source data 1. [file elife-79183-fig5-data1.zip › Figure 5-source data 1/Figure 5E-souce data/Figure 5E-source data1-rawRAD51panel_Exposure_37.8sec.tif]

**E**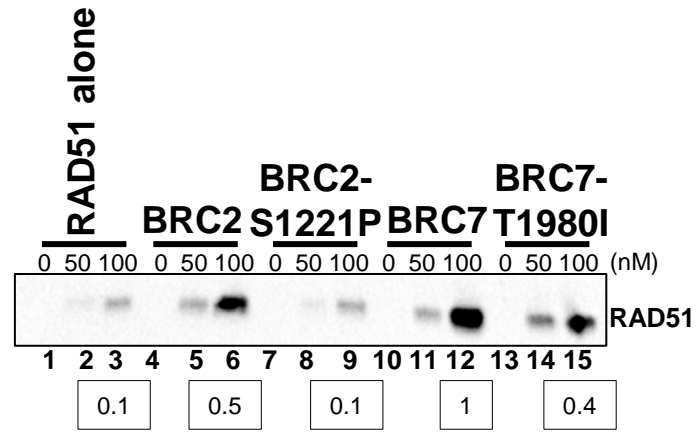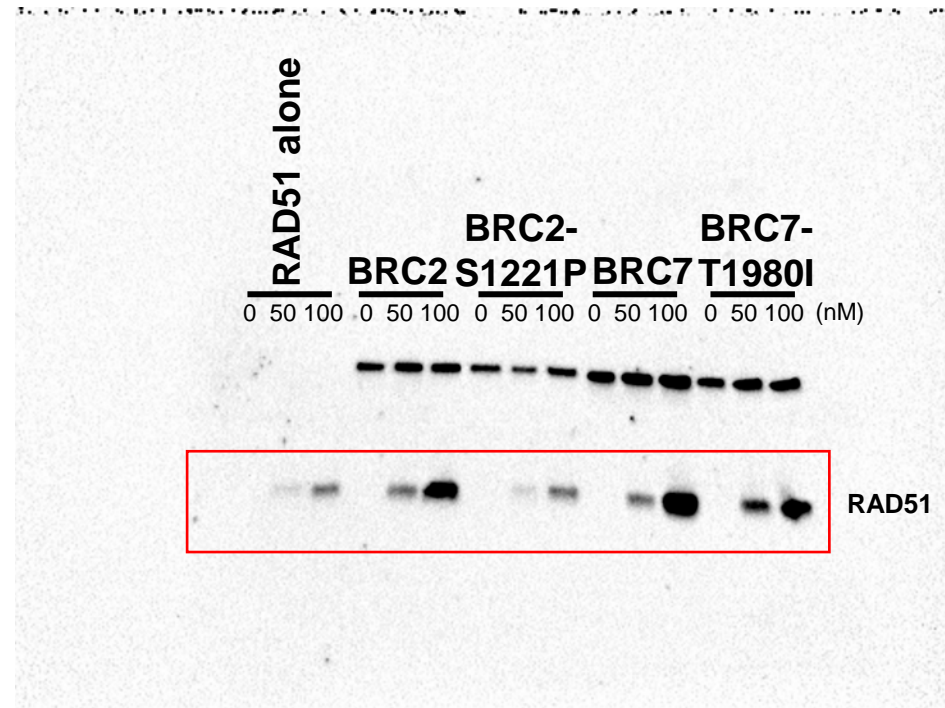

Supplement: Figure 5—source data 1. [file elife-79183-fig5-data1.zip › Figure 5-source data 1/Figure 5E-souce data/Figure 5E-source data3-highlightedbandsandlabeled.pdf]

## Slide 1
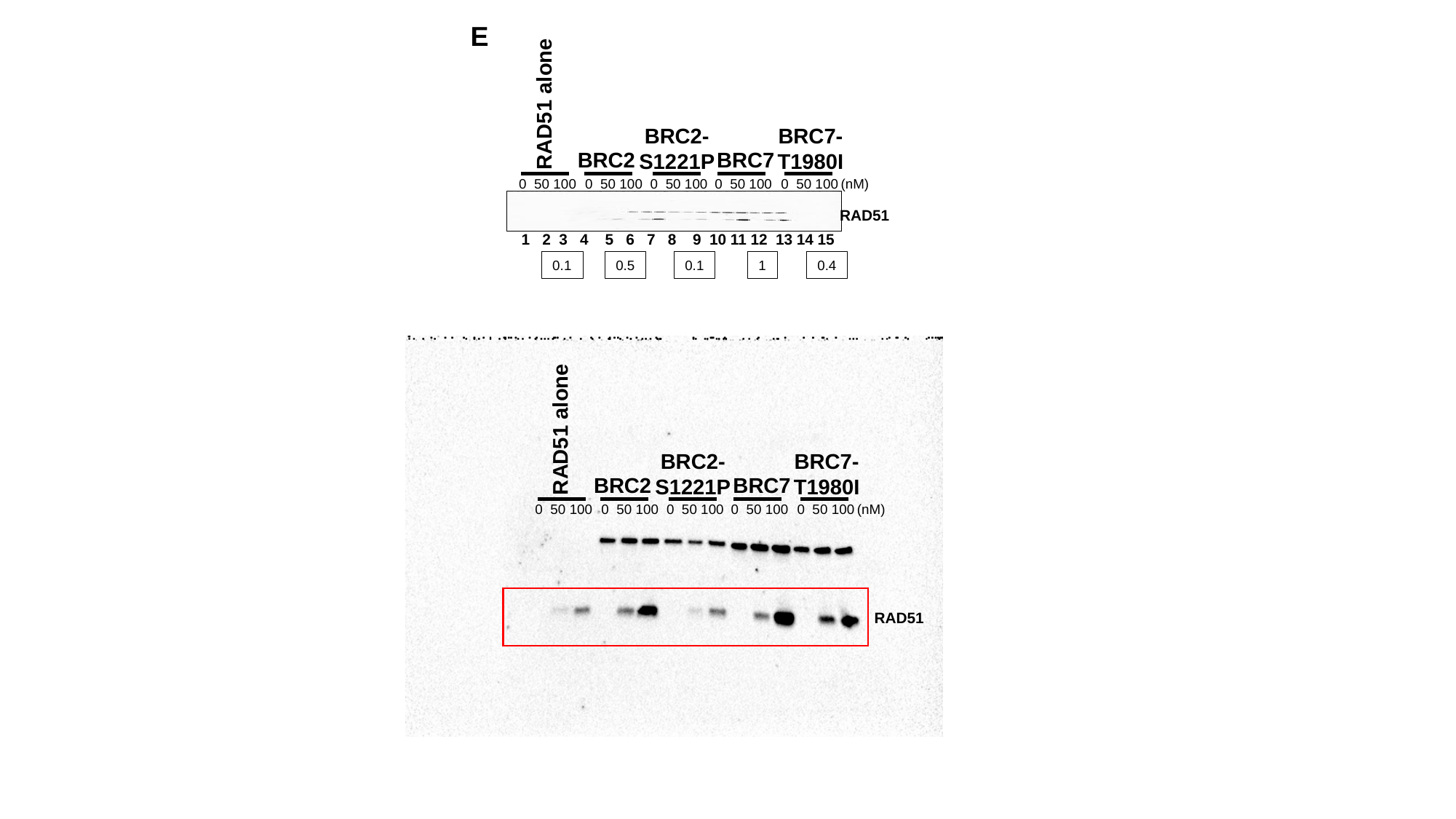

E
RAD51 alone
BRC2-
S1221P
BRC7-
T1980I
BRC2
BRC7
 (nM)
 0 50 100
 0 50 100
 0 50 100
 0 50 100
 0 50 100
RAD51
 1 2 3 4 5 6 7 8 9 10 11 12 13 14 15
0.1
0.5
0.1
1
0.4
RAD51 alone
BRC2-
S1221P
BRC7-
T1980I
BRC2
BRC7
 (nM)
 0 50 100
 0 50 100
 0 50 100
 0 50 100
 0 50 100
RAD51

Supplement: Figure 5—source data 1. [file elife-79183-fig5-data1.zip › Figure 5-source data 1/Figure 5E-souce data/Figure 5E-source data4-highlightedbandsandlabeled.pptx]

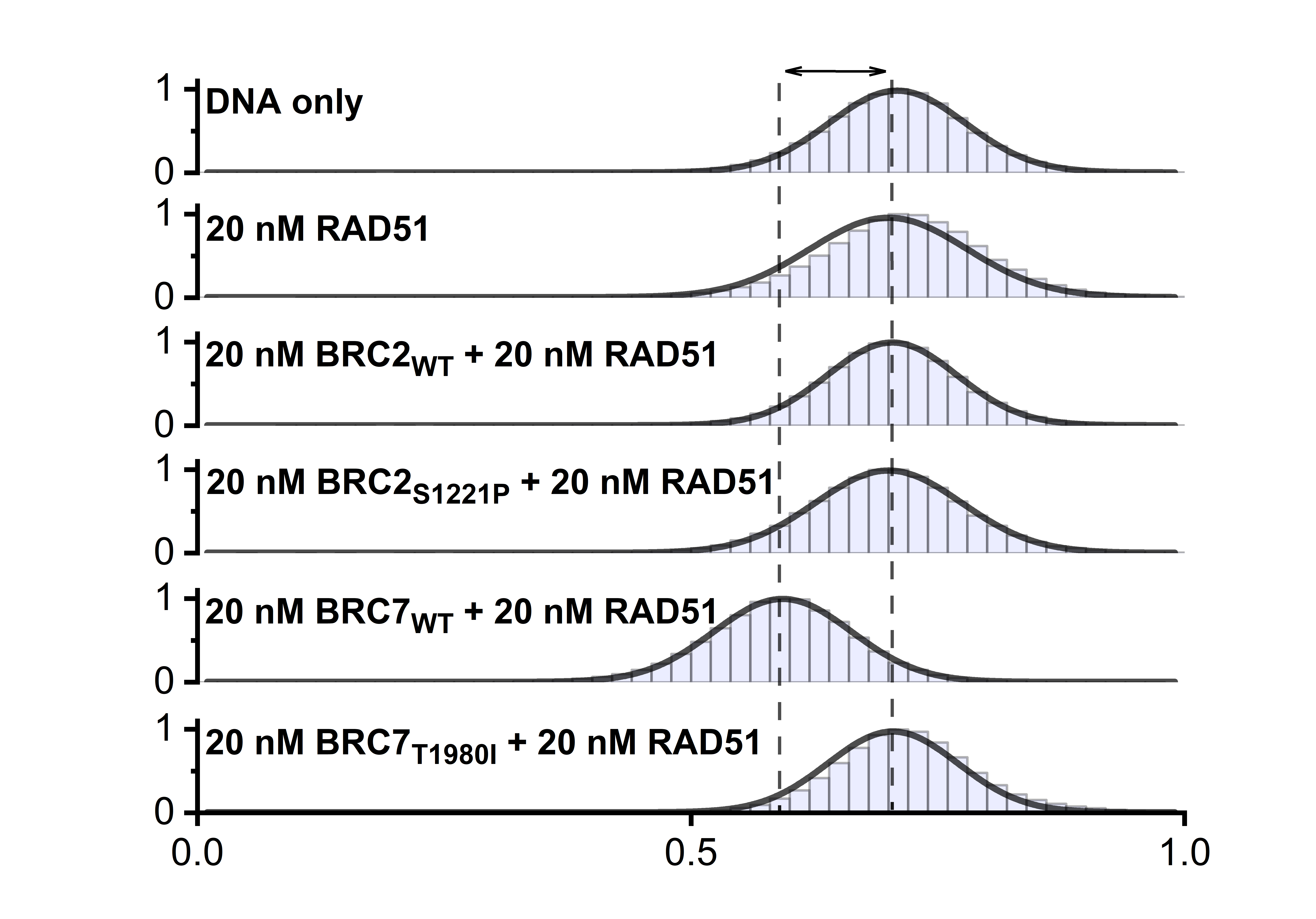

Supplement: Figure 5—source data 1. [file elife-79183-fig5-data1.zip › Figure 5-source data 1/Figure 5F-source data/Figure 5F-source data1.tif]

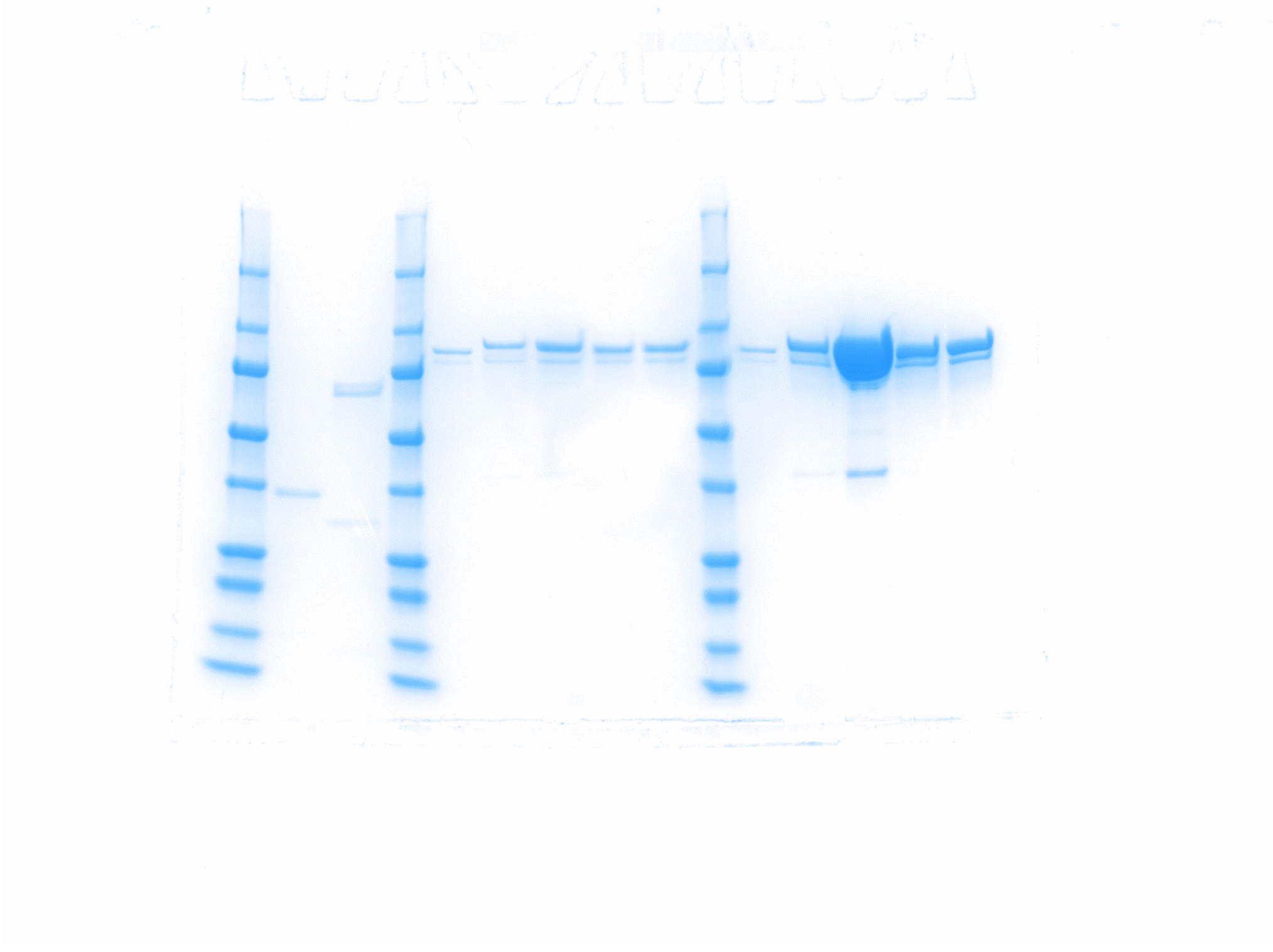

Supplement: Figure 5—figure supplement 1—source data 1. [file elife-79183-fig5-figsupp1-data1.zip › Figure 5-figure supplement 1-source data1/Figure 5-figure supplement 1A-source data1/Figure 5-figure supplement 1A-source data1-raw protein purifications.tif]

**A**

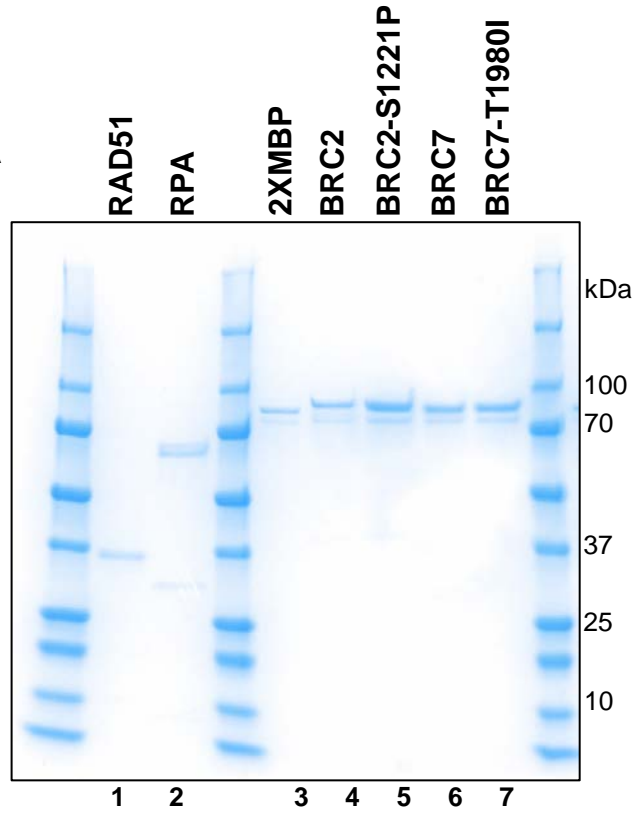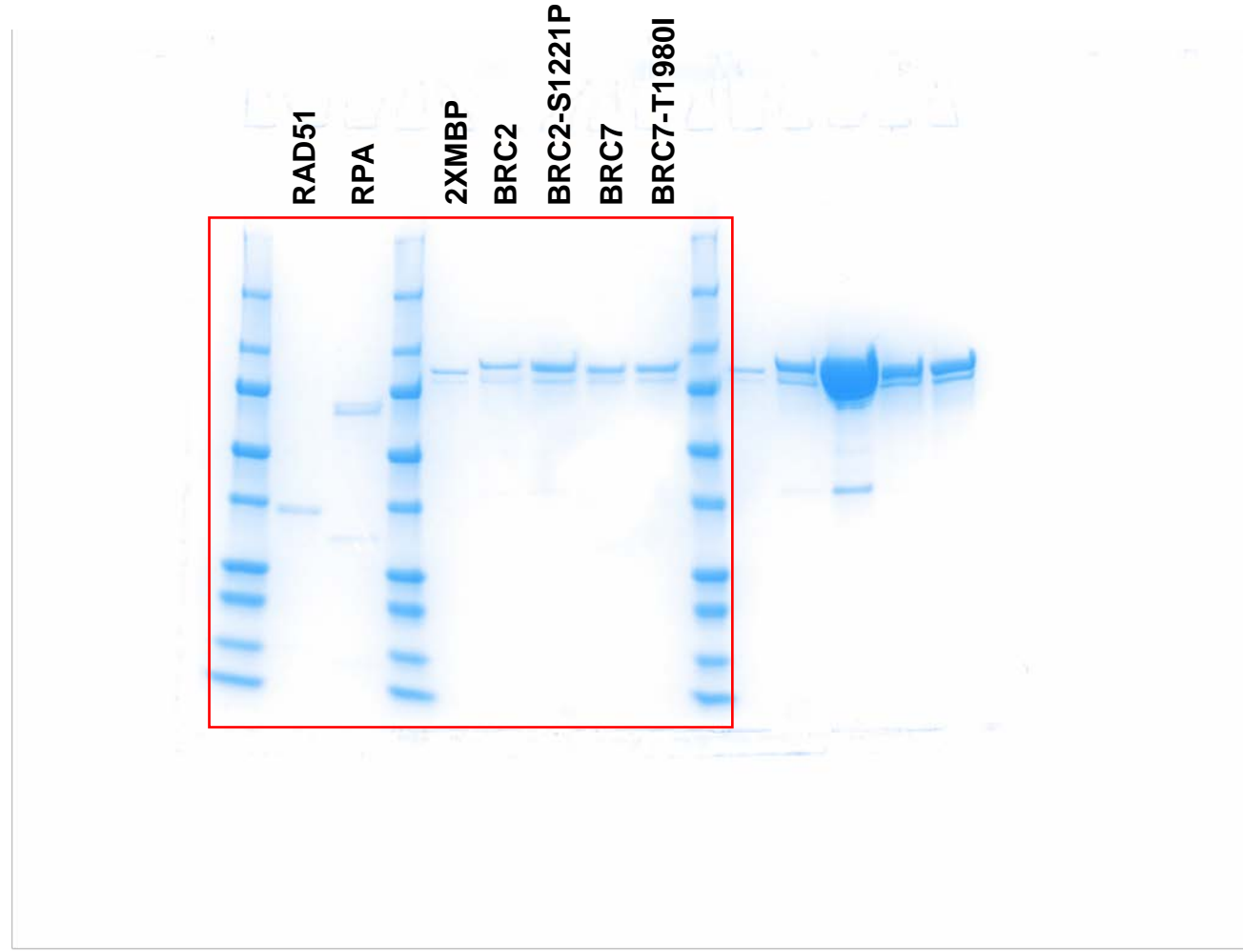

Supplement: Figure 5—figure supplement 1—source data 1. [file elife-79183-fig5-figsupp1-data1.zip › Figure 5-figure supplement 1-source data1/Figure 5-figure supplement 1A-source data1/Figure 5-figure supplement 1A-source data2-highlightedbandsandlabeled.pdf]

## Slide 1
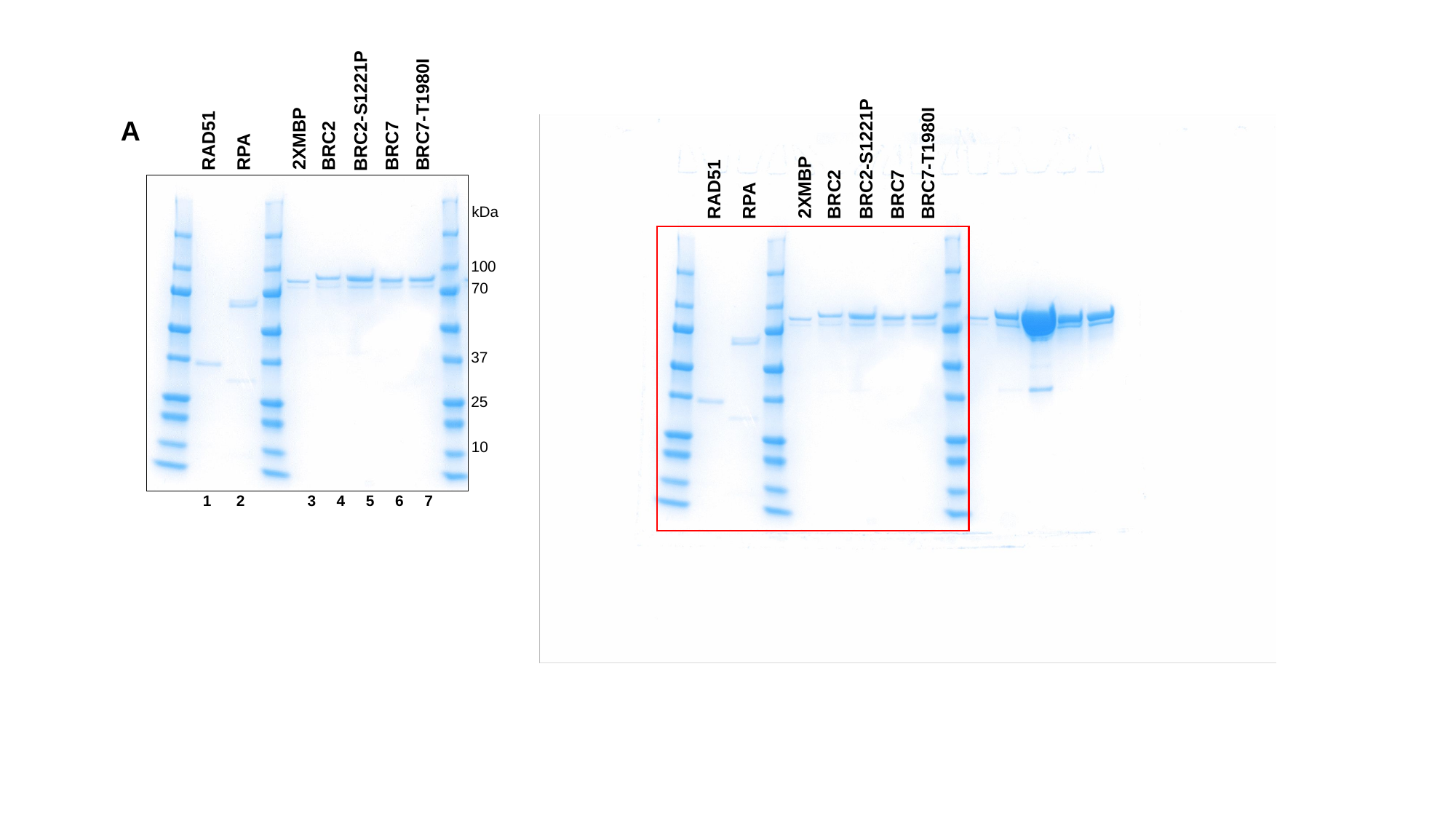

BRC2-S1221P
BRC7-T1980I
A
2XMBP
RAD51
BRC2
BRC7
RPA
BRC2-S1221P
BRC7-T1980I
2XMBP
RAD51
BRC2
BRC7
RPA
kDa
100
70
37
25
10
1 2 3 4 5 6 7

Supplement: Figure 5—figure supplement 1—source data 1. [file elife-79183-fig5-figsupp1-data1.zip › Figure 5-figure supplement 1-source data1/Figure 5-figure supplement 1A-source data1/Figure 5-figure supplement 1A-source data3-highlightedbandsandlabeled.pptx]

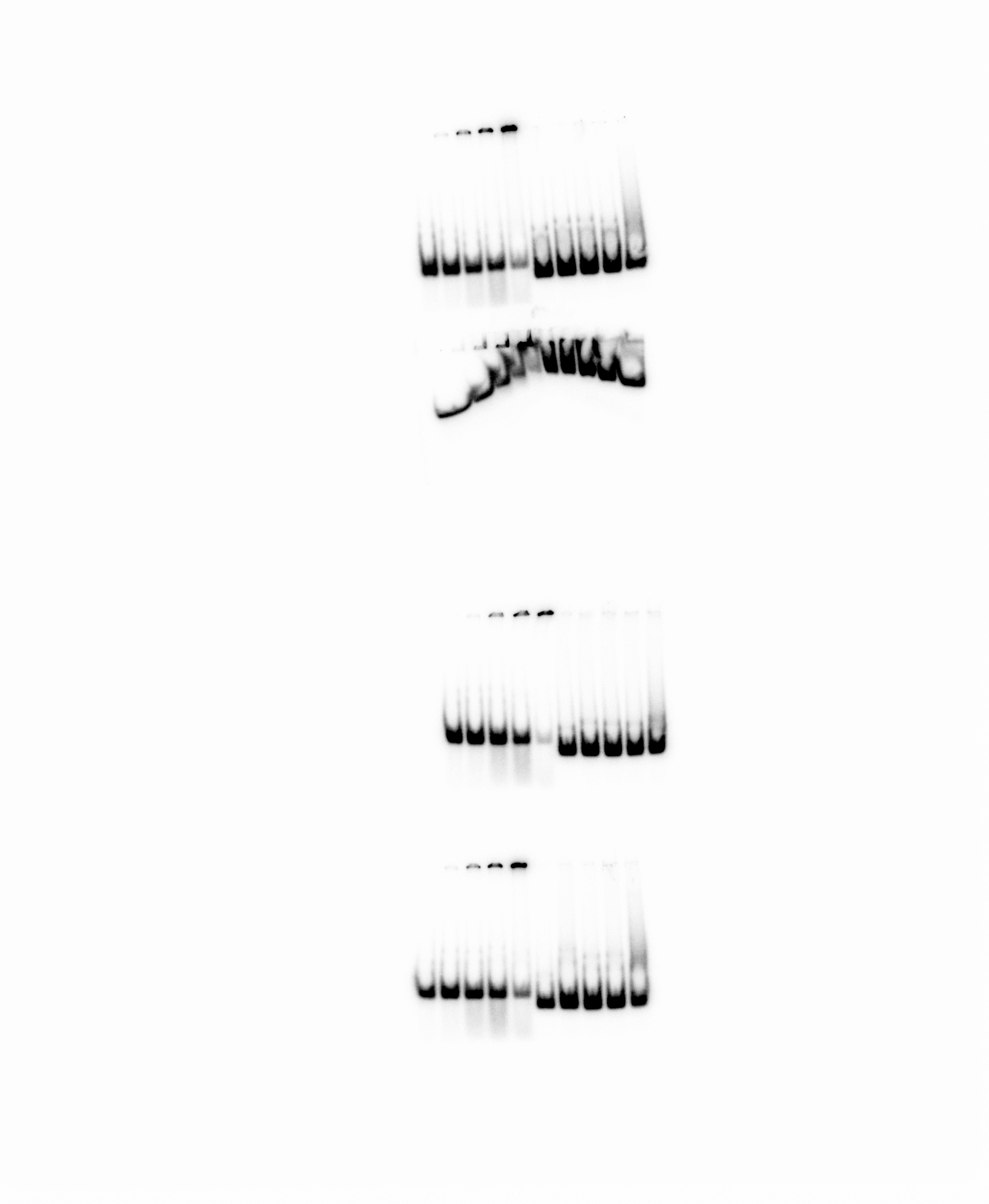

Supplement: Figure 5—figure supplement 1—source data 1. [file elife-79183-fig5-figsupp1-data1.zip › Figure 5-figure supplement 1-source data1/Figure 5-figure supplement 1B-source data1/Figure 5-figure supplement 1B-source data1-raw EMSA different RAD51concentrations.tif]

**B**

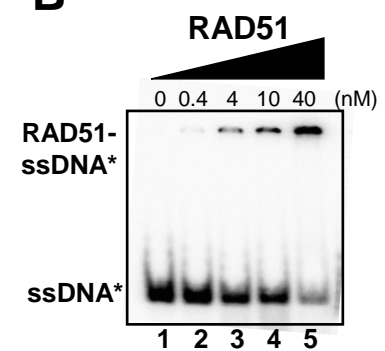

**RAD51**

0 0.4 4 10 40 (nM)

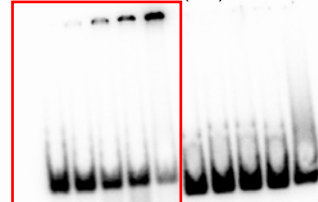

Supplement: Figure 5—figure supplement 1—source data 1. [file elife-79183-fig5-figsupp1-data1.zip › Figure 5-figure supplement 1-source data1/Figure 5-figure supplement 1B-source data1/Figure 5-figure supplement 1B-source data2-highlightedbandsandlabeled.pdf]

## Slide 1
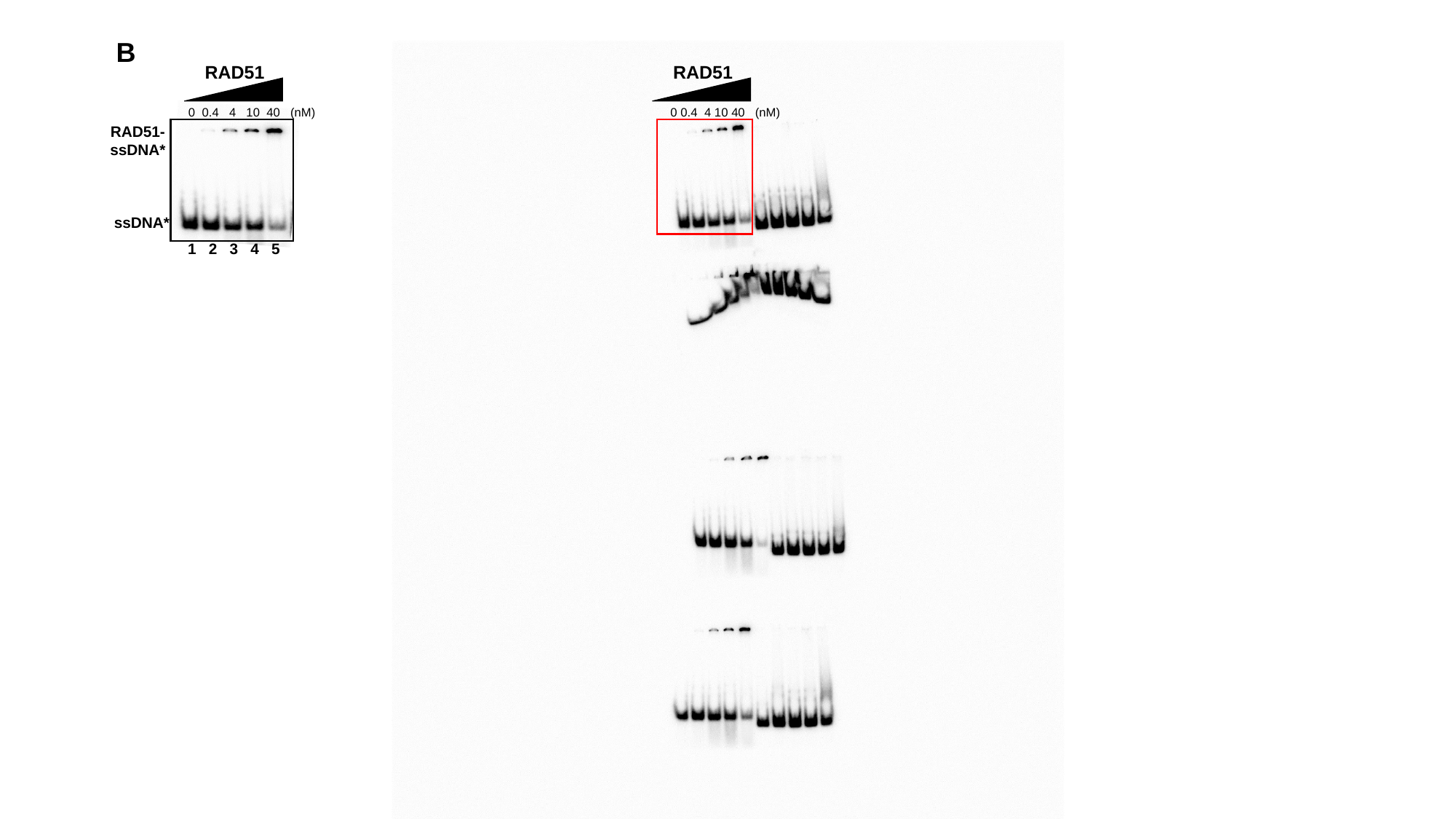

B
RAD51
RAD51
 0 0.4 4 10 40 (nM)
0 0.4 4 10 40 (nM)
RAD51-ssDNA*
ssDNA*
1 2 3 4 5

Supplement: Figure 5—figure supplement 1—source data 1. [file elife-79183-fig5-figsupp1-data1.zip › Figure 5-figure supplement 1-source data1/Figure 5-figure supplement 1B-source data1/Figure 5-figure supplement 1B-source data3-highlightedbandsandlabeled.pptx]

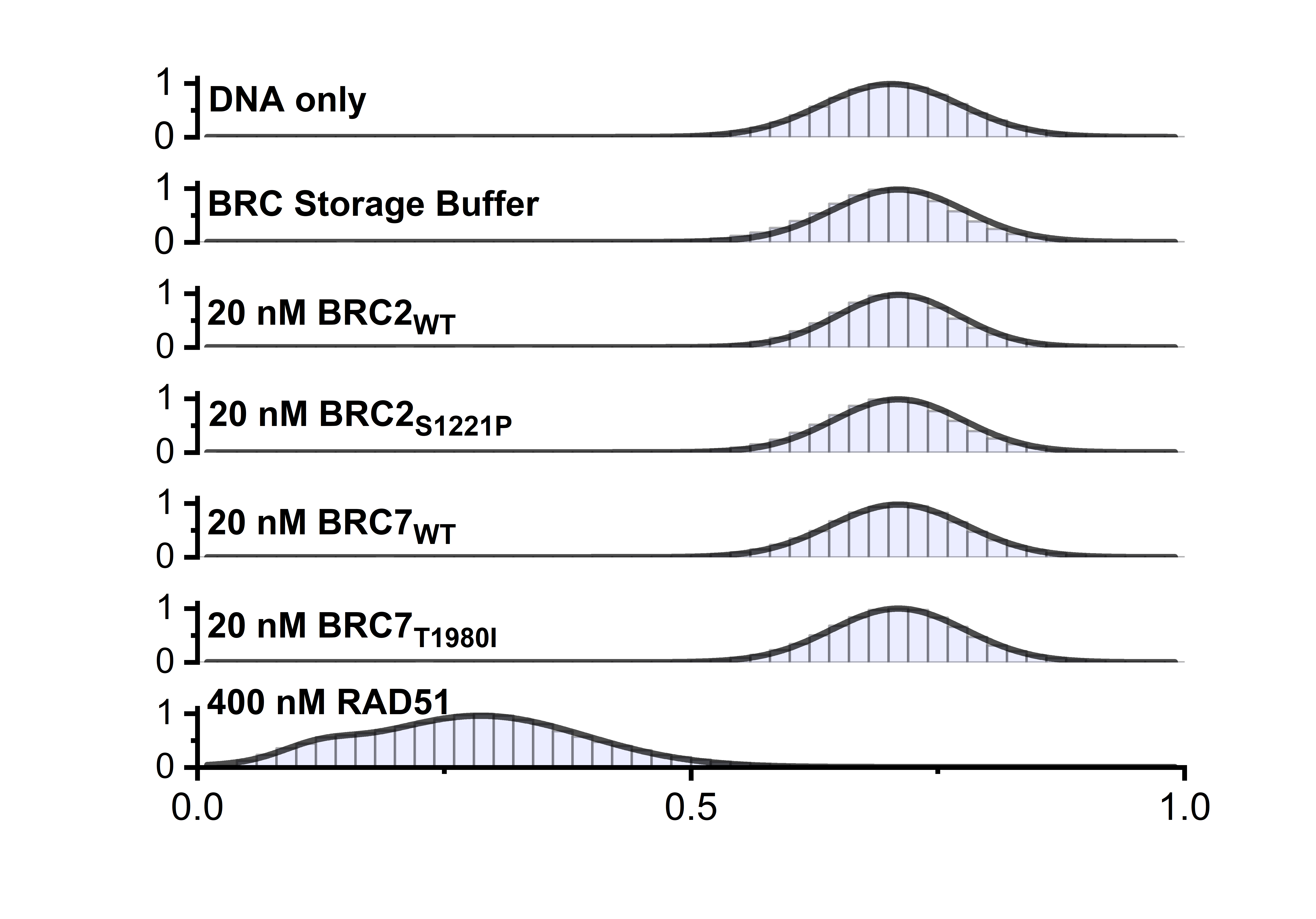

Supplement: Figure 5—figure supplement 2—source data 1. [file elife-79183-fig5-figsupp2-data1.zip › Figure 5-figure supplement 2-source data1/Figure 5-figure supplement 2A-source data1/Figure 5-figure supplement 2A-source data1-raw.tif]

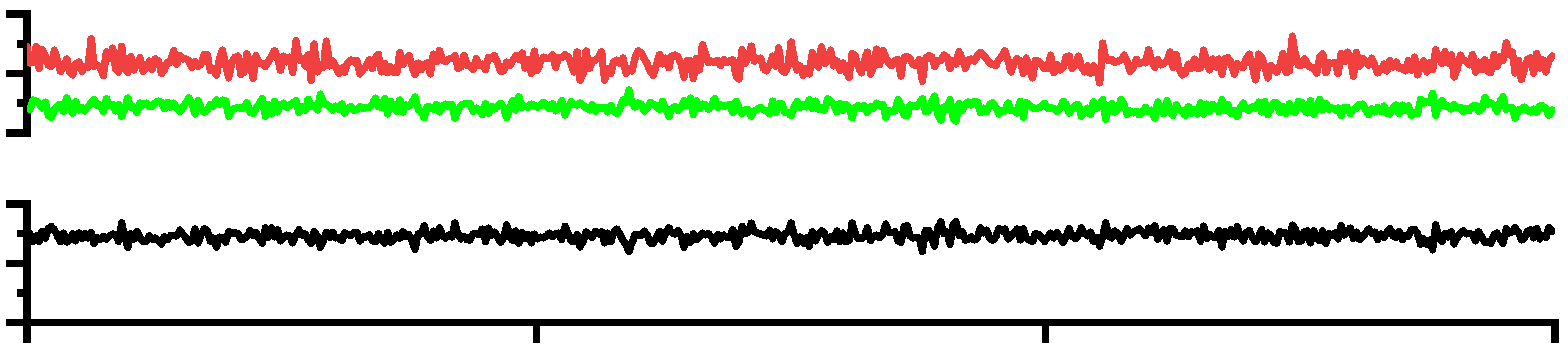

Supplement: Figure 5—figure supplement 2—source data 1. [file elife-79183-fig5-figsupp2-data1.zip › Figure 5-figure supplement 2-source data1/Figure 5-figure supplement 2C-source data1/Figure 5-figure supplement 2C-source data1-DNA only.tif]

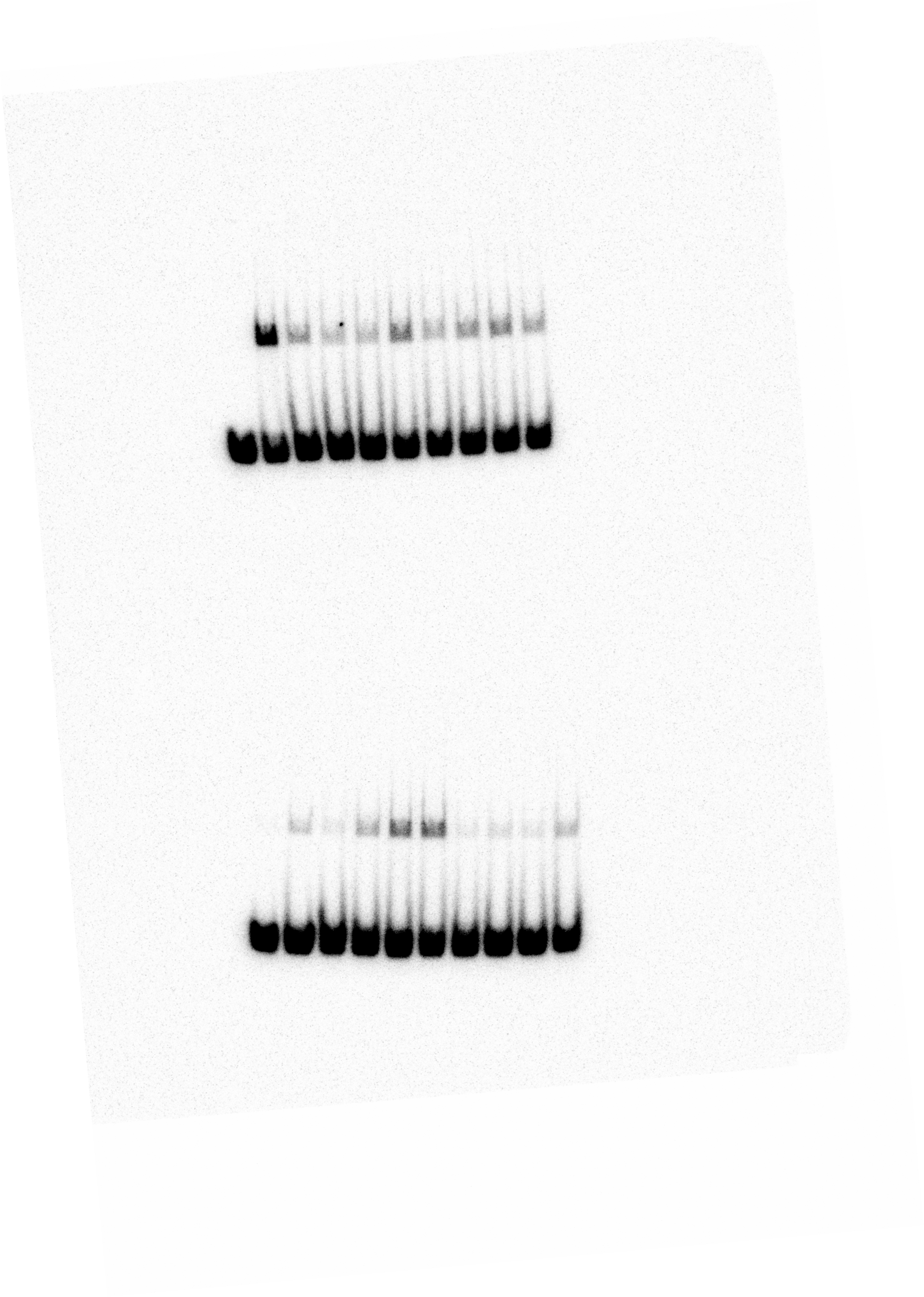

Supplement: Figure 6—source data 1. [file elife-79183-fig6-data1.zip › Figure 6-source data 1/Figure 6B-source data1/Figure 6B-source data1-rawDNA strand exchange.tif]

**B**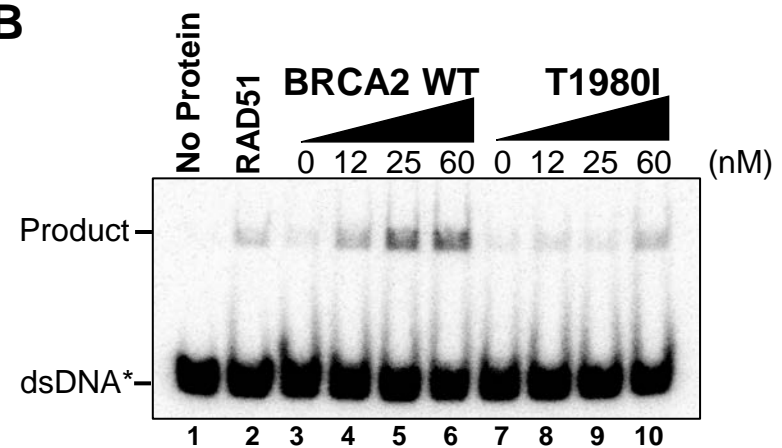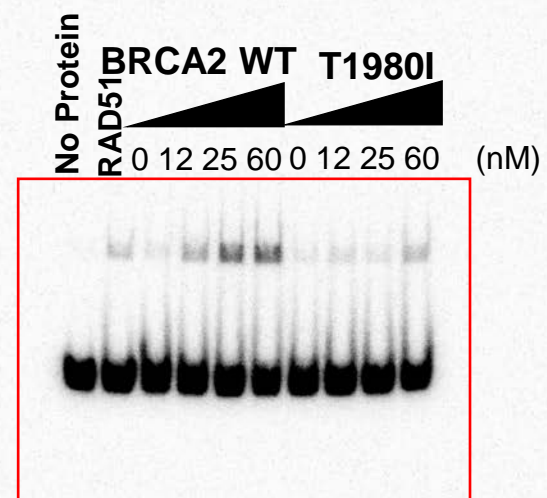

Supplement: Figure 6—source data 1. [file elife-79183-fig6-data1.zip › Figure 6-source data 1/Figure 6B-source data1/Figure 6B-source data2-highlightedbandsandlabeled.pdf]

## Slide 1
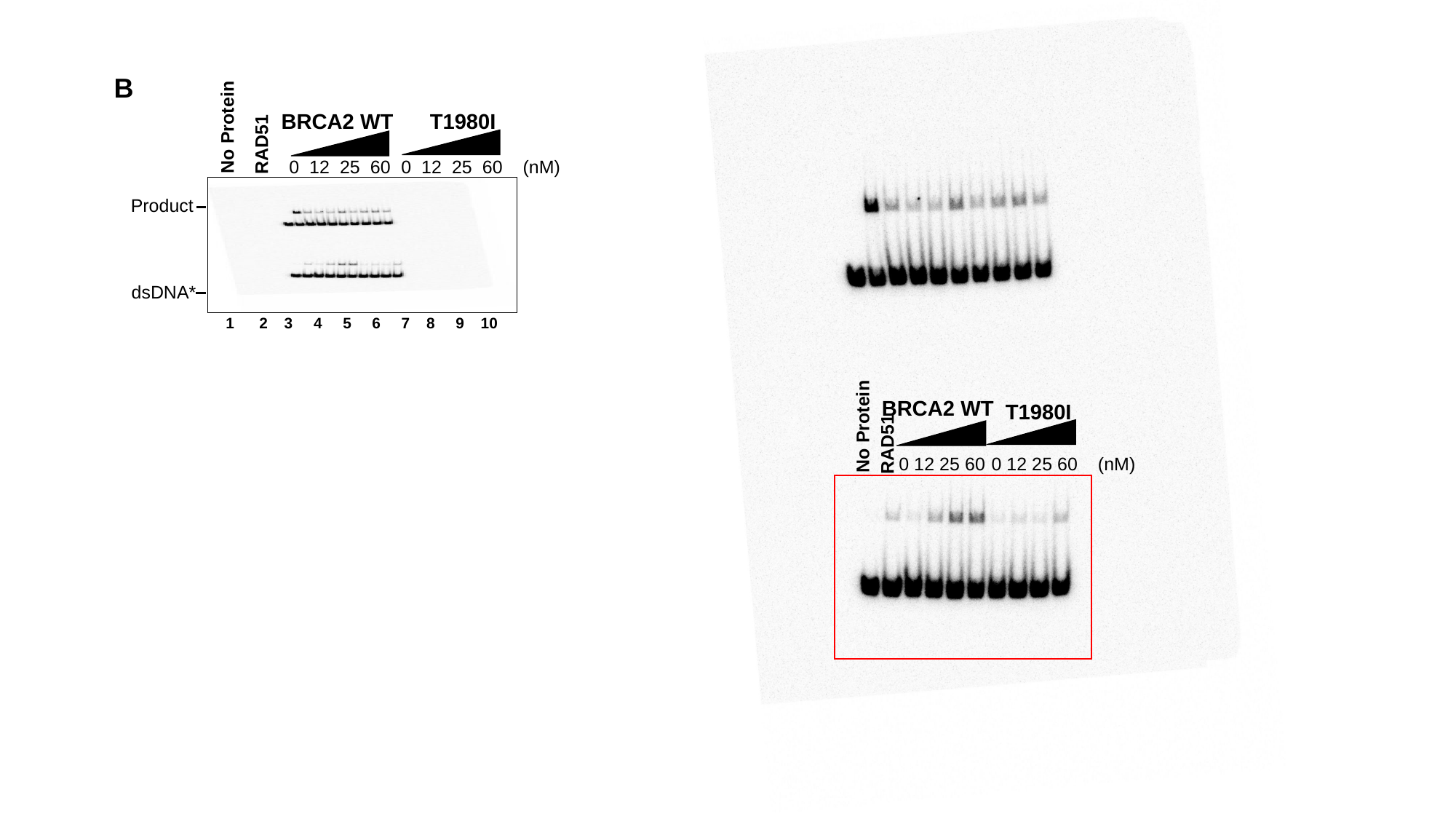

B
BRCA2 WT
T1980I
No Protein
RAD51
0 12 25 60
0 12 25 60 (nM)
Product
dsDNA*
1 2 3 4 5 6 7 8 9 10
BRCA2 WT
T1980I
No Protein
RAD51
0 12 25 60 (nM)
0 12 25 60

Supplement: Figure 6—source data 1. [file elife-79183-fig6-data1.zip › Figure 6-source data 1/Figure 6B-source data1/Figure 6B-source data3-highlightedbandsandlabeled.pptx]

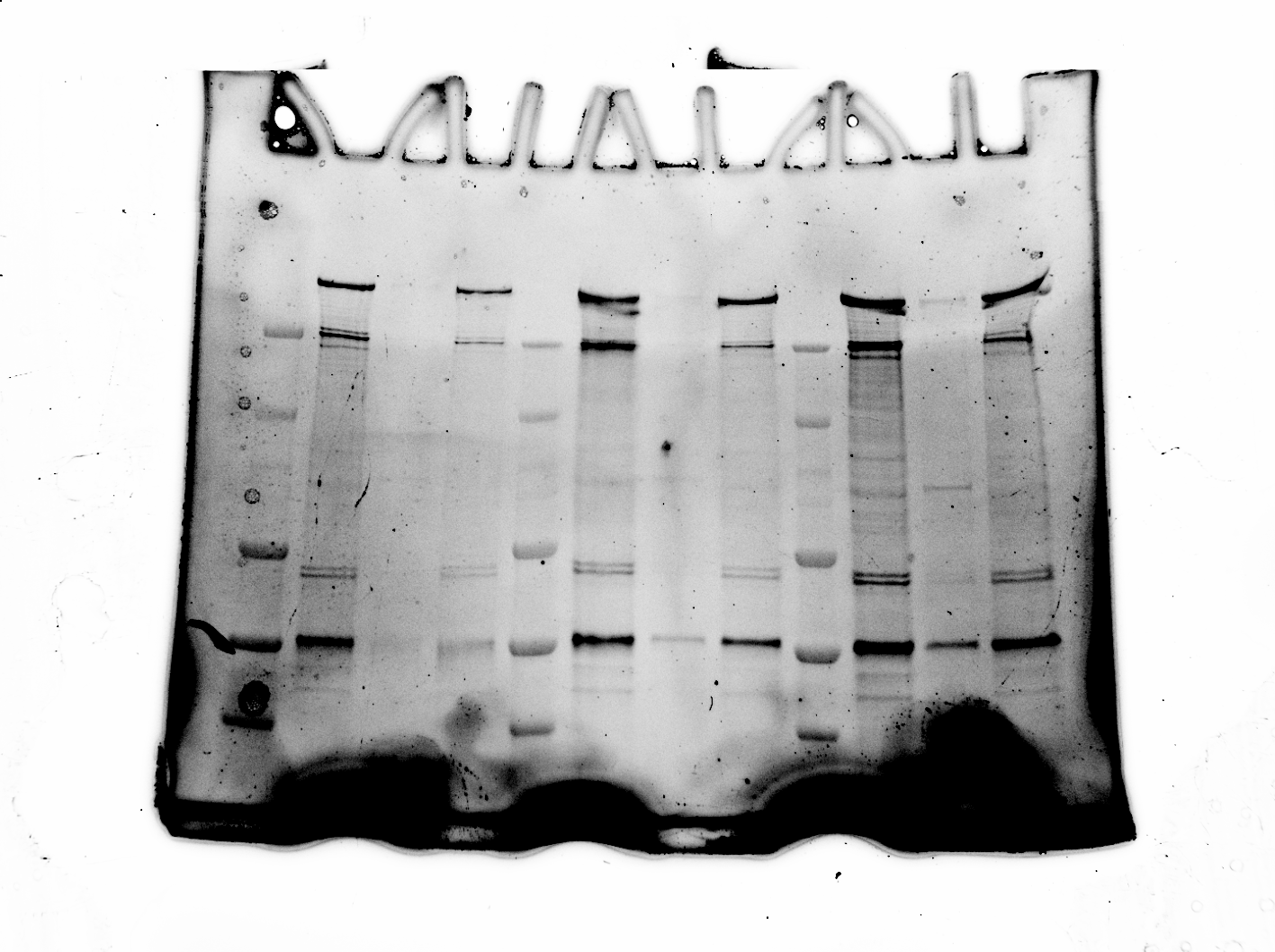

Supplement: Figure 6—figure supplement 1—source data 1. [file elife-79183-fig6-figsupp1-data1.zip › Figure 6-figure supplement 1-source data1/Figure 6-figure supplement 1A-source data1/Figure 6-figure supplement 1A-source data1-BRCA2 purification.tif]

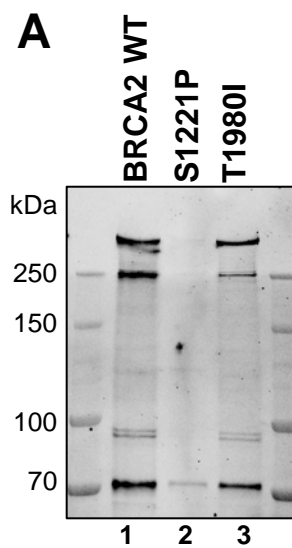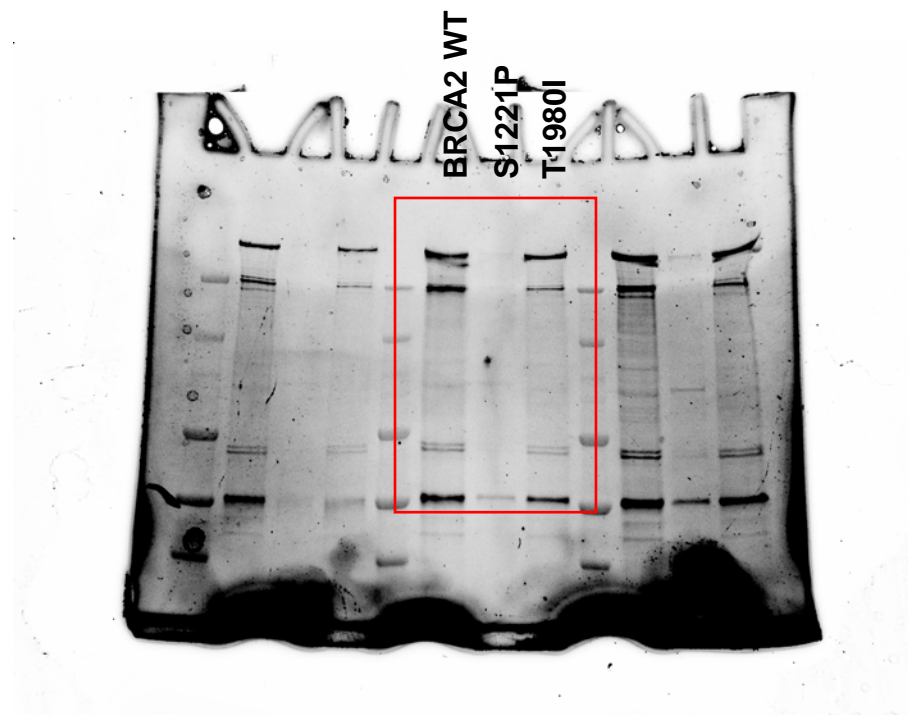

Supplement: Figure 6—figure supplement 1—source data 1. [file elife-79183-fig6-figsupp1-data1.zip › Figure 6-figure supplement 1-source data1/Figure 6-figure supplement 1A-source data1/Figure 6-figure supplement 1A-sourcedat2-BRCA2 purificationhighlightedbandsandlabeled.pdf]

## Slide 1
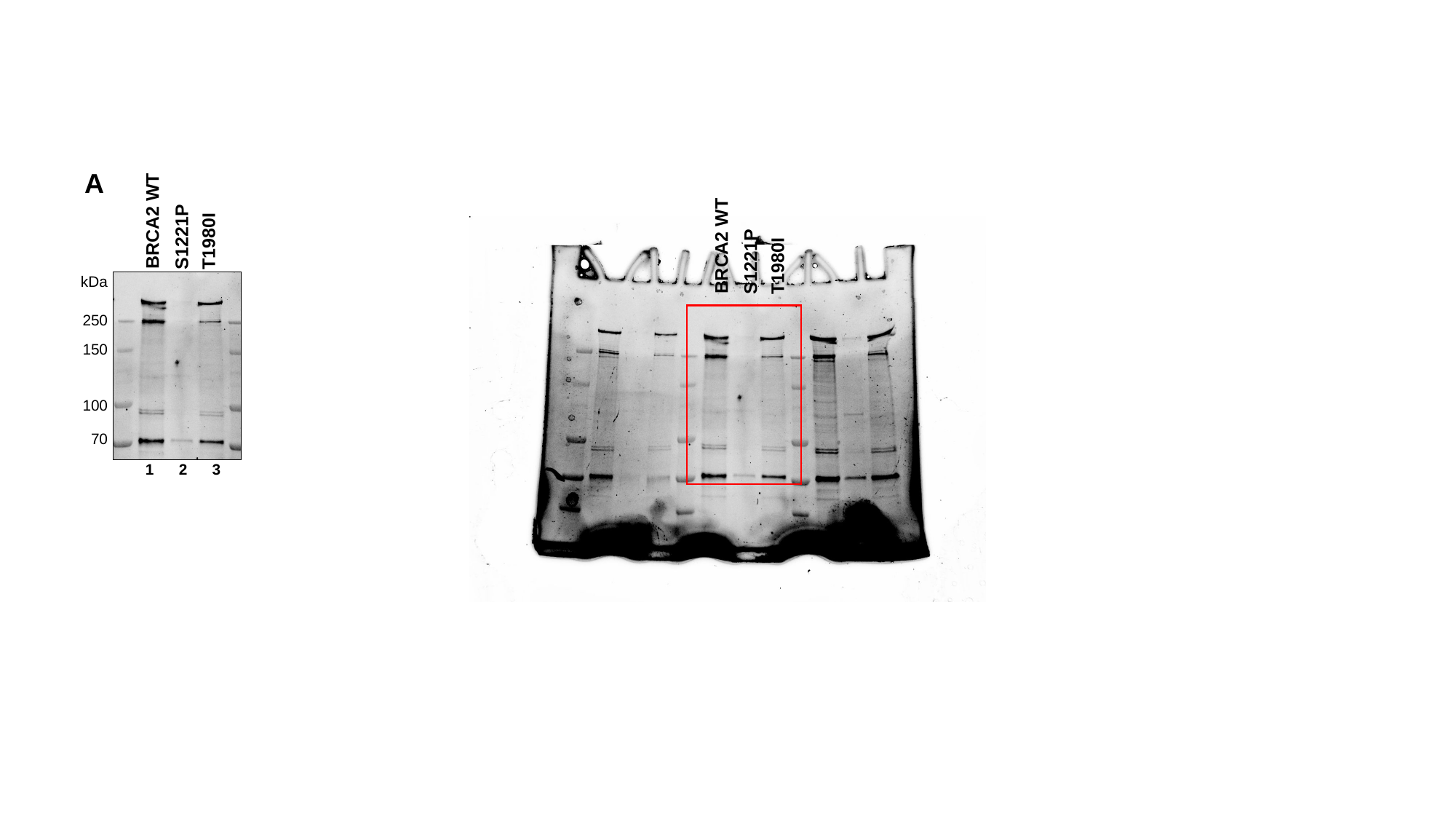

A
BRCA2 WT
S1221P
T1980I
BRCA2 WT
S1221P
T1980I
kDa
250
150
100
70
1 2 3

Supplement: Figure 6—figure supplement 1—source data 1. [file elife-79183-fig6-figsupp1-data1.zip › Figure 6-figure supplement 1-source data1/Figure 6-figure supplement 1A-source data1/Figure 6-figure supplement 1A-sourcedat3-BRCA2 purificationhighlightedbandsandlabeled.pptx]

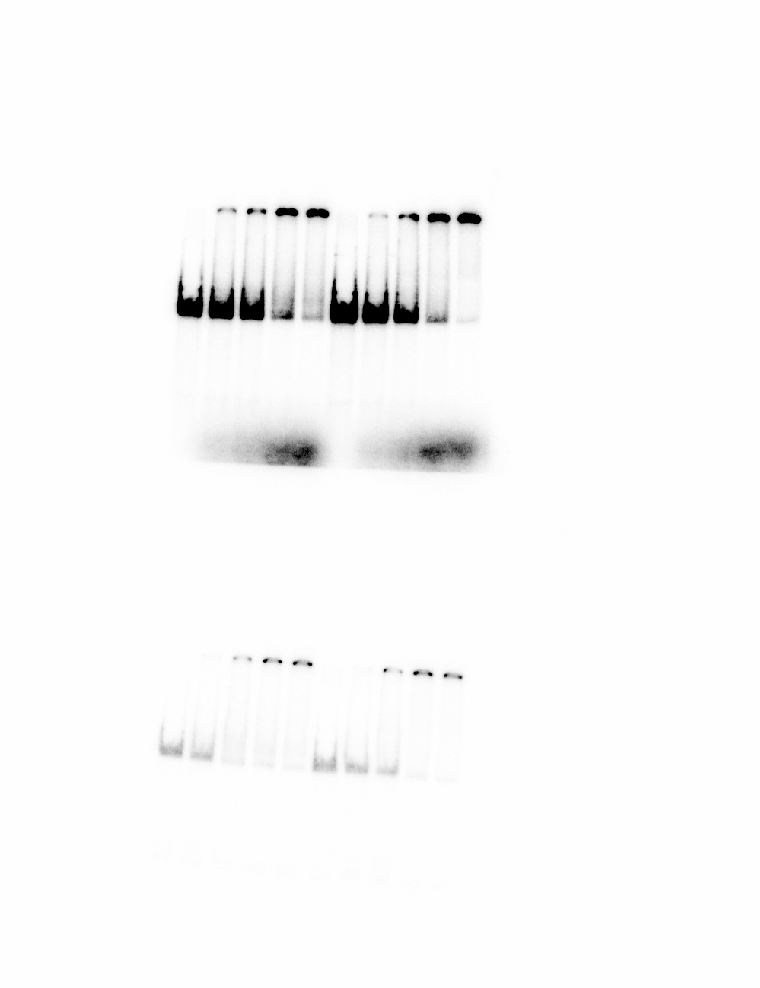

Supplement: Figure 6—figure supplement 1—source data 1. [file elife-79183-fig6-figsupp1-data1.zip › Figure 6-figure supplement 1-source data1/Figure 6-figure supplement 1B-source data1/Figure 6-figure supplement 1B-source data1-3'T1980I raw.tif]

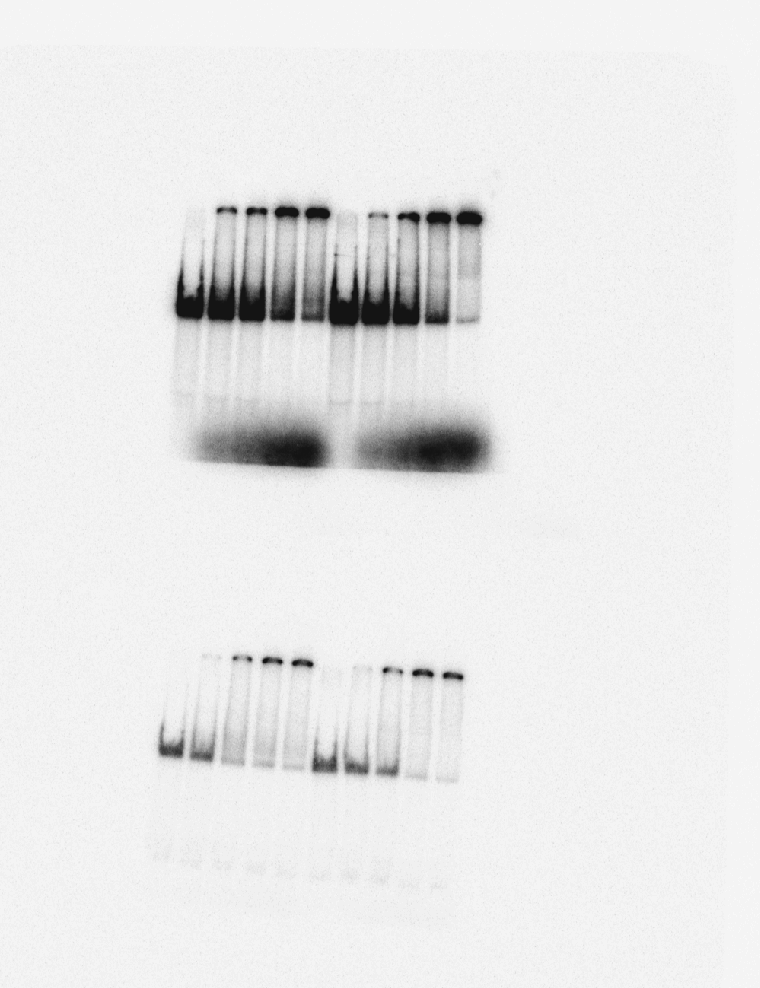

Supplement: Figure 6—figure supplement 1—source data 1. [file elife-79183-fig6-figsupp1-data1.zip › Figure 6-figure supplement 1-source data1/Figure 6-figure supplement 1B-source data1/Figure 6-figure supplement 1B-source data2-5'T1980I raw.tif]

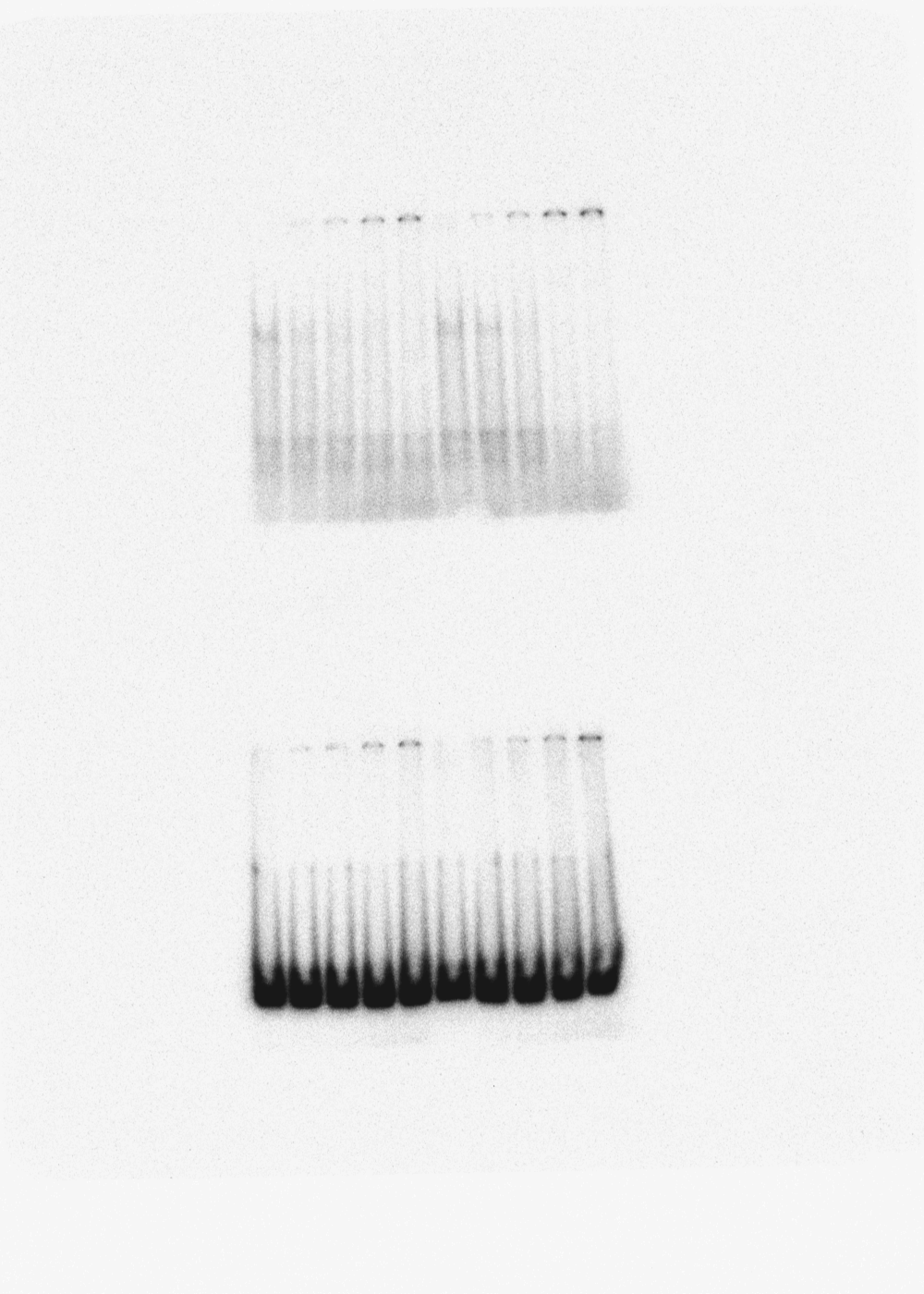

Supplement: Figure 6—figure supplement 1—source data 1. [file elife-79183-fig6-figsupp1-data1.zip › Figure 6-figure supplement 1-source data1/Figure 6-figure supplement 1B-source data1/Figure 6-figure supplement 1B-source data3-ssDNA dsDNA raw.tif]
