# Supplementary material for: BRCA2 BRC missense variants disrupt RAD51-dependent DNA repair
Source: eLife. 2022 Sep 13;11:e79183. doi: 10.7554/eLife.79183 (PMC9545528; doi:10.7554/eLife.79183)
Supplement: Figure 2—source data 1. [file elife-79183-fig2-data1.zip › Figure 2-source data 1/Figure2A-source data1/Figure2A-sourcedata4-highlightedbandsandlabels.pptx]

## Slide 1
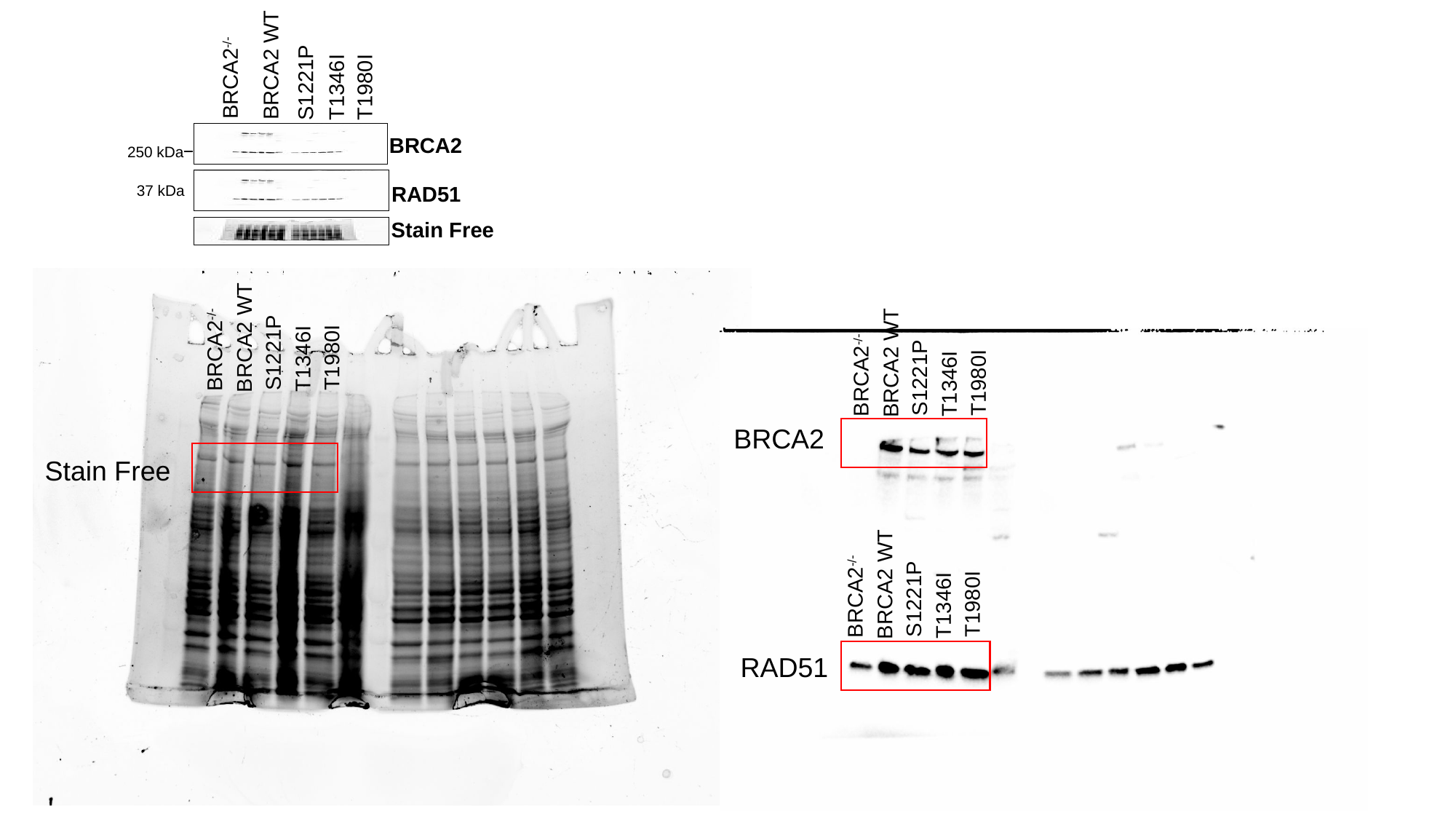

BRCA2 WT
BRCA2-/-
S1221P
T1346I
T1980I
BRCA2
250 kDa
 37 kDa
RAD51
Stain Free
BRCA2 WT
BRCA2-/-
S1221P
T1980I
T1346I
BRCA2 WT
BRCA2-/-
S1221P
T1980I
T1346I
BRCA2
Stain Free
BRCA2 WT
BRCA2-/-
S1221P
T1980I
T1346I
RAD51
