# Supplementary material for: BRCA2 BRC missense variants disrupt RAD51-dependent DNA repair
Source: eLife. 2022 Sep 13;11:e79183. doi: 10.7554/eLife.79183 (PMC9545528; doi:10.7554/eLife.79183)
Supplement: Figure 2—figure supplement 1—source data 1. — Raw Stain Free. Nt Ct BRCA2 RAD51 highlighedbandsandlabels. [file elife-79183-fig2-figsupp1-data1.zip › Figure 2-figure supplement 1-source data1 A/Figure1-suppfig2-highligthedbandsandlabelsA.pptx]

## Slide 1
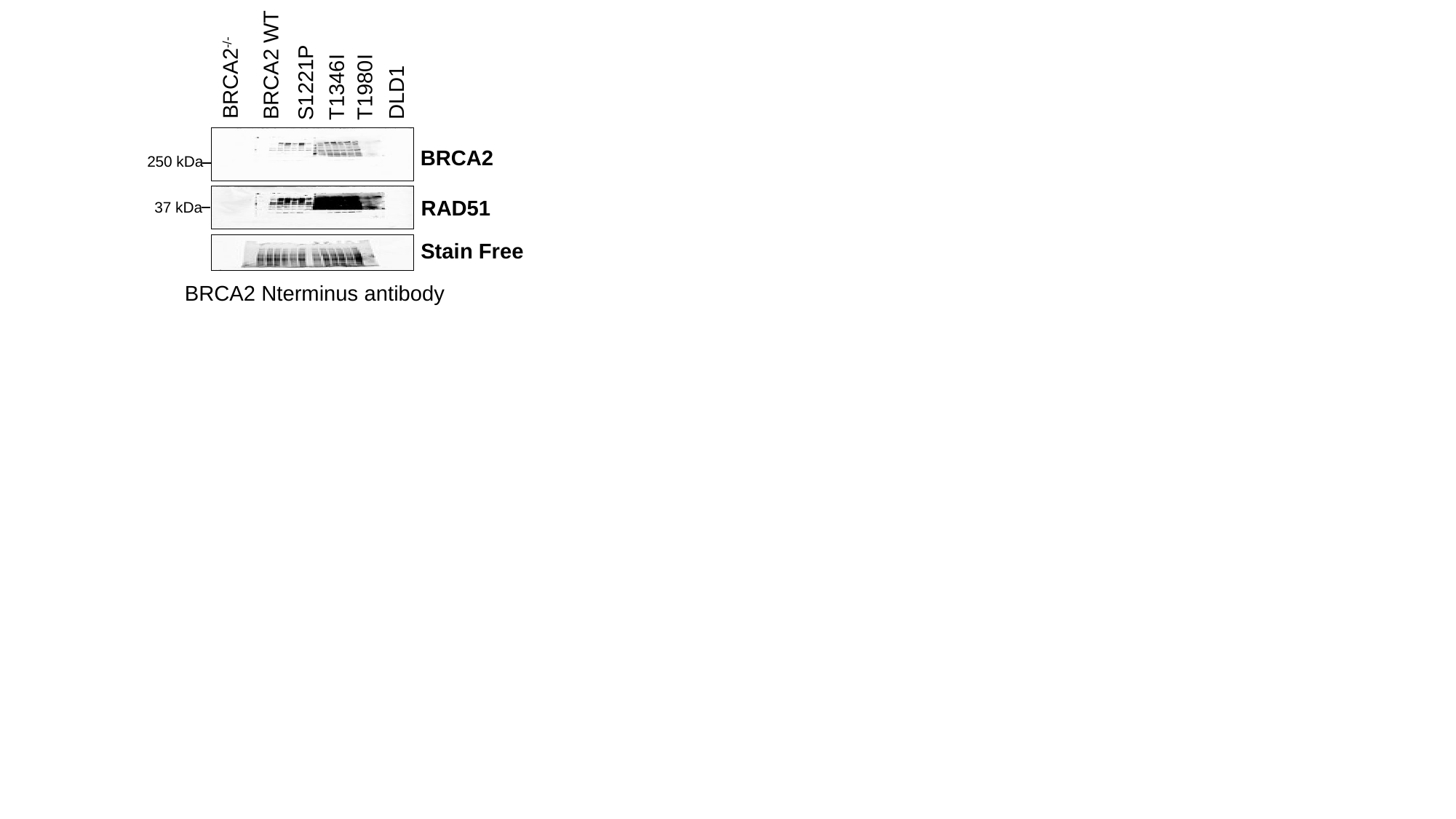

BRCA2 WT
BRCA2-/-
S1221P
T1346I
T1980I
DLD1
BRCA2
250 kDa
RAD51
 37 kDa
Stain Free
BRCA2 Nterminus antibody

## Slide 2
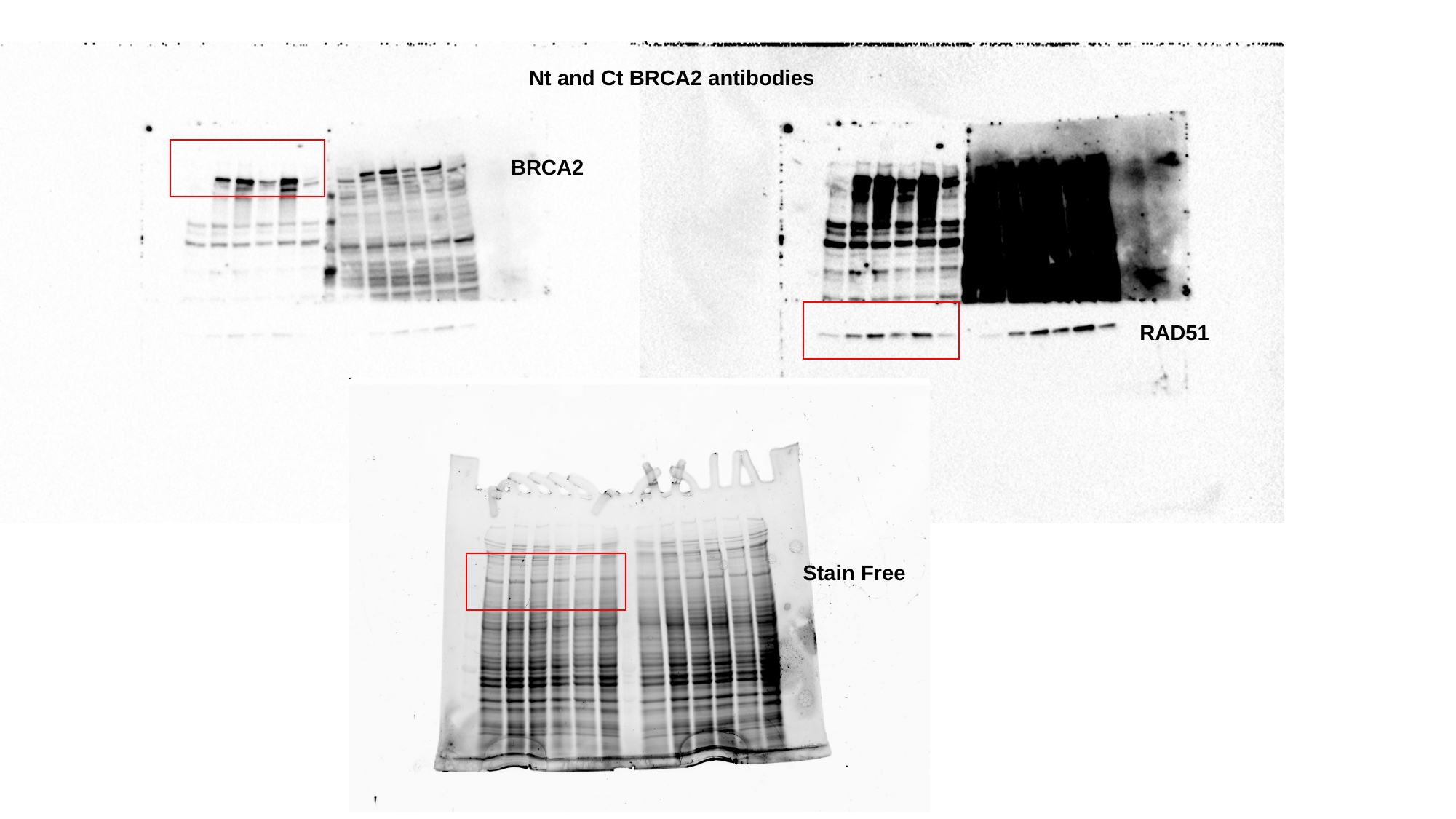

Nt and Ct BRCA2 antibodies
BRCA2
RAD51
Stain Free
