# Supplementary material for: BRCA2 BRC missense variants disrupt RAD51-dependent DNA repair
Source: eLife. 2022 Sep 13;11:e79183. doi: 10.7554/eLife.79183 (PMC9545528; doi:10.7554/eLife.79183)
Supplement: Figure 4—source data 1. [file elife-79183-fig4-data1.zip › Figure 4-source data 1/Figure4D-source data1/Figure4D-source data3-highlightedbandsandlabeled.pptx]

## Slide 1
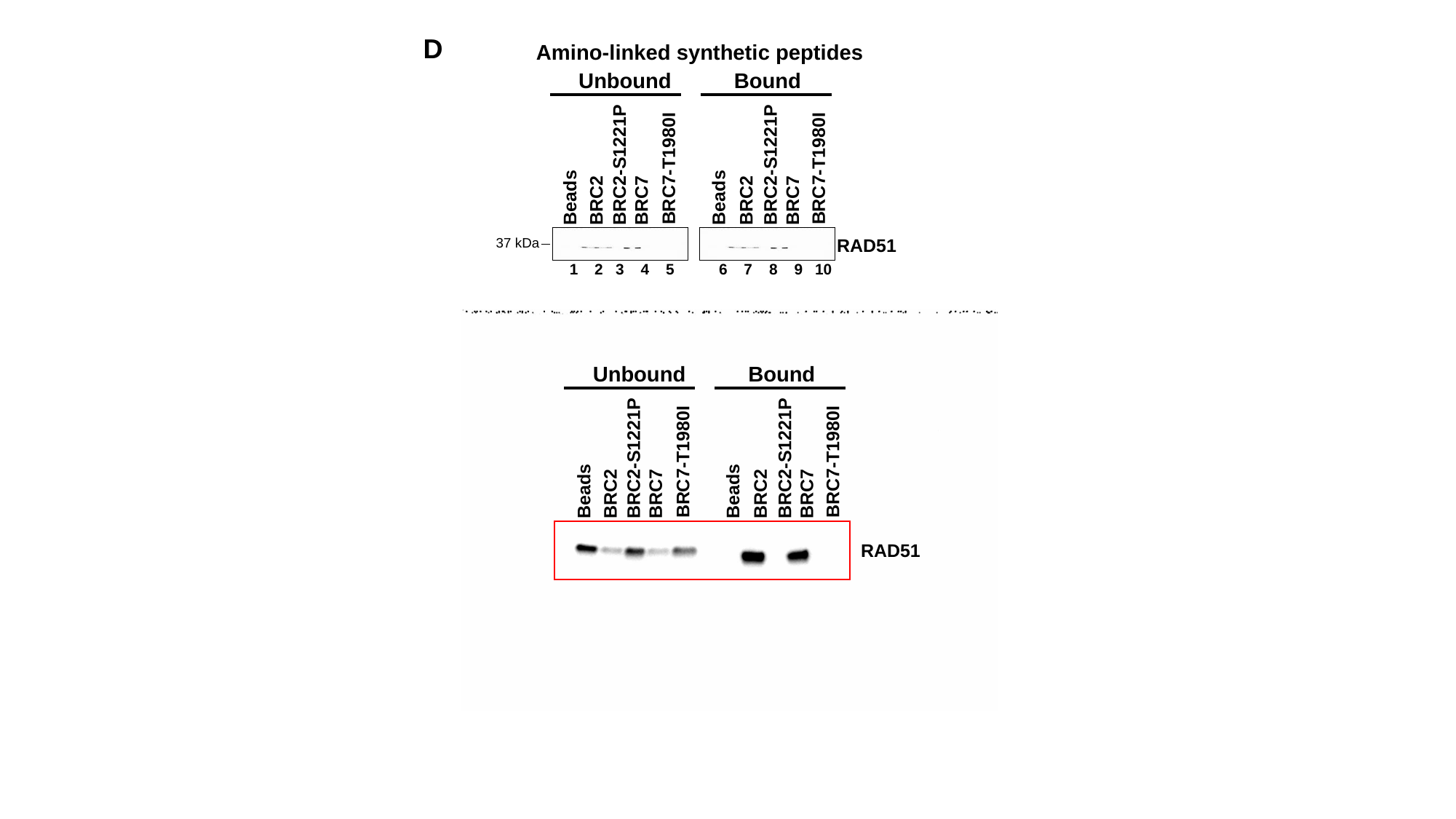

D
Amino-linked synthetic peptides
Unbound
Bound
BRC2-S1221P
BRC2-S1221P
BRC7-T1980I
BRC7-T1980I
Beads
Beads
BRC2
BRC7
BRC2
BRC7
RAD51
 37 kDa
 1 2 3 4 5
 6 7 8 9 10
Unbound
Bound
BRC2-S1221P
BRC2-S1221P
BRC7-T1980I
BRC7-T1980I
Beads
Beads
BRC2
BRC7
BRC2
BRC7
RAD51
