# Supplementary material for: BRCA2 BRC missense variants disrupt RAD51-dependent DNA repair
Source: eLife. 2022 Sep 13;11:e79183. doi: 10.7554/eLife.79183 (PMC9545528; doi:10.7554/eLife.79183)
Supplement: Figure 4—figure supplement 1—source data 1. [file elife-79183-fig4-figsupp1-data1.zip › Figure 4-figure supplement 1-source data1/Figure 4-figure supplement 1A-source data 1/Figure 4-figure supplement 1A-source data8-highlightedbandsandlabeled.pptx]

## Slide 1
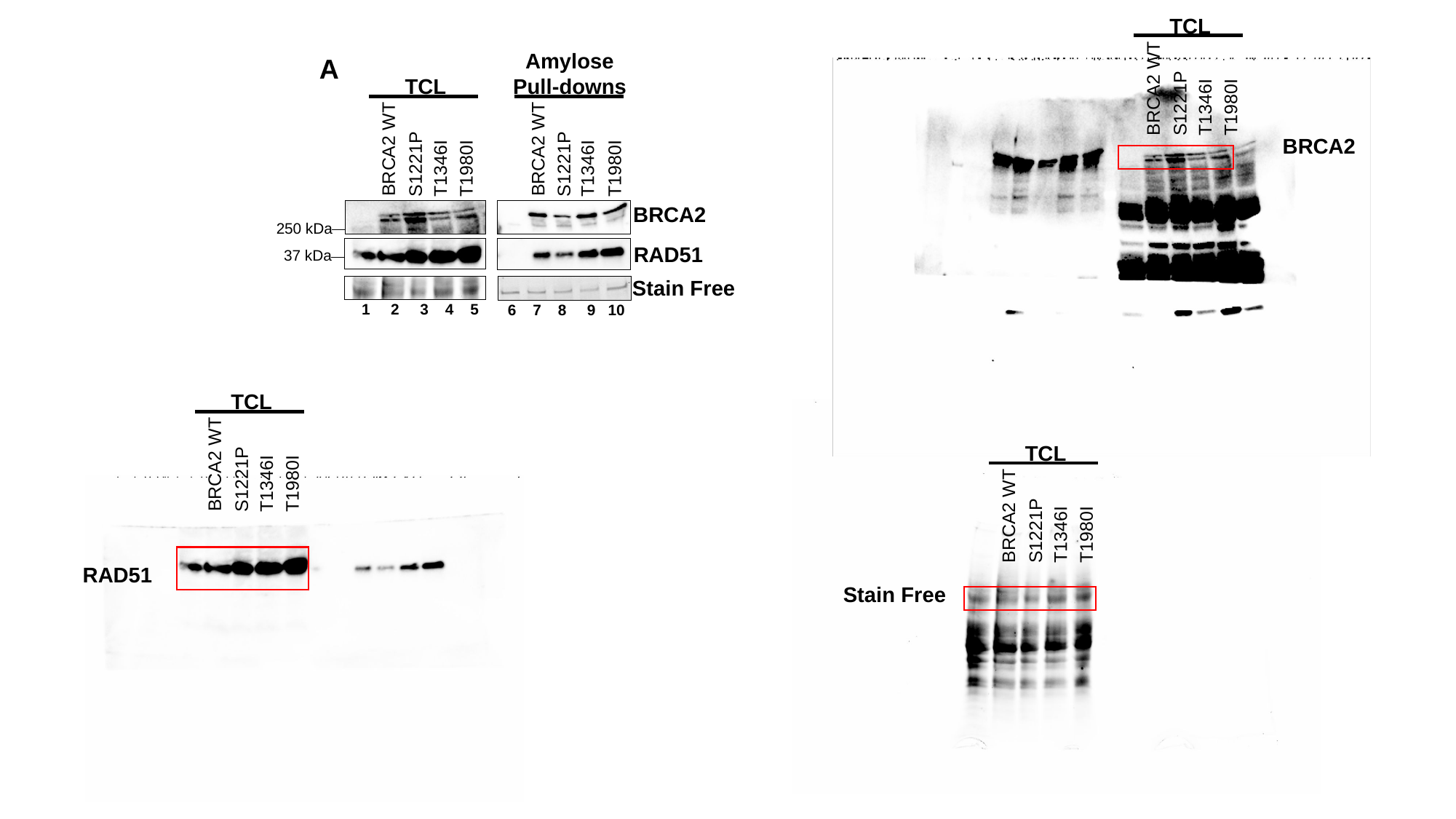

TCL
Amylose
Pull-downs
A
TCL
BRCA2 WT
S1221P
T1346I
T1980I
BRCA2
BRCA2 WT
BRCA2 WT
S1221P
S1221P
T1346I
T1980I
T1346I
T1980I
BRCA2
250 kDa
RAD51
 37 kDa
Stain Free
1 2 3 4 5
6 7 8 9 10
TCL
TCL
BRCA2 WT
S1221P
T1346I
T1980I
BRCA2 WT
S1221P
T1346I
T1980I
RAD51
Stain Free

## Slide 2
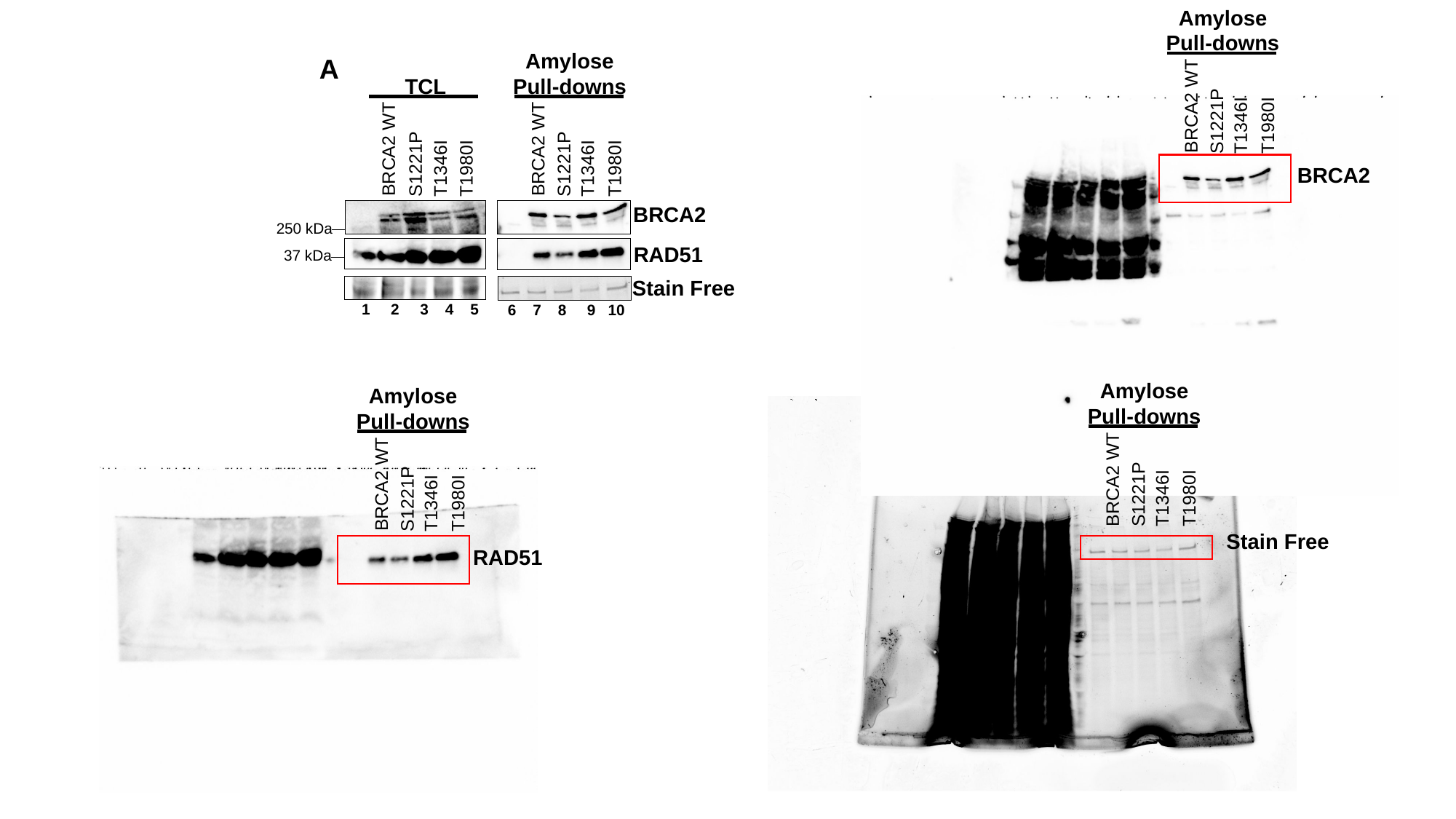

Amylose
Pull-downs
Amylose
Pull-downs
A
TCL
BRCA2 WT
S1221P
T1346I
T1980I
BRCA2 WT
BRCA2 WT
S1221P
S1221P
T1346I
T1980I
T1346I
T1980I
BRCA2
BRCA2
250 kDa
RAD51
 37 kDa
Stain Free
1 2 3 4 5
6 7 8 9 10
Amylose
Pull-downs
Amylose
Pull-downs
BRCA2 WT
BRCA2 WT
S1221P
T1346I
T1980I
S1221P
T1346I
T1980I
Stain Free
RAD51
