# Supplementary material for: BRCA2 BRC missense variants disrupt RAD51-dependent DNA repair
Source: eLife. 2022 Sep 13;11:e79183. doi: 10.7554/eLife.79183 (PMC9545528; doi:10.7554/eLife.79183)
Supplement: Figure 4—figure supplement 1—source data 1. [file elife-79183-fig4-figsupp1-data1.zip › Figure 4-figure supplement 1-source data1/Figure 4-figure supplement 1B-source data 1/Figure 4-figure supplement 1B-source data5-highlightedbandsandlabeled.pptx]

## Slide 1
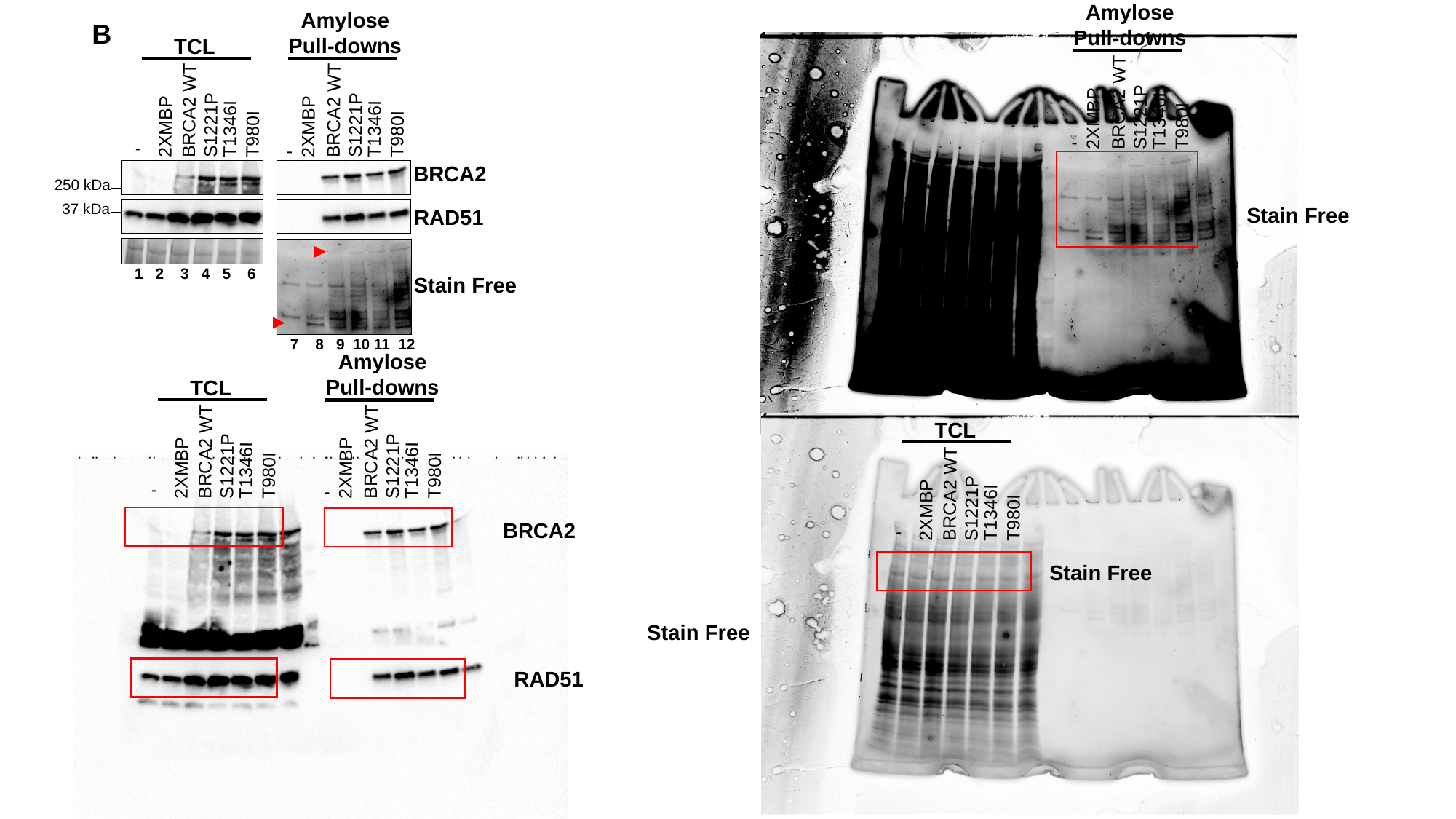

Amylose
Pull-downs
Amylose
Pull-downs
B
TCL
BRCA2 WT
S1221P
T1346I
T980I
BRCA2 WT
S1221P
T1346I
T980I
BRCA2 WT
S1221P
T1346I
T980I
2XMBP
2XMBP
2XMBP
-
-
-
BRCA2
250 kDa
 37 kDa
Stain Free
RAD51
1 2 3 4 5 6
Stain Free
Amylose
Pull-downs
7 8 9 10 11 12
TCL
BRCA2 WT
S1221P
T1346I
T980I
BRCA2 WT
S1221P
T1346I
T980I
TCL
2XMBP
2XMBP
BRCA2 WT
S1221P
T1346I
T980I
2XMBP
-
-
BRCA2
-
Stain Free
Stain Free
RAD51
