# Supplementary material for: BRCA2 BRC missense variants disrupt RAD51-dependent DNA repair
Source: eLife. 2022 Sep 13;11:e79183. doi: 10.7554/eLife.79183 (PMC9545528; doi:10.7554/eLife.79183)
Supplement: Figure 4—figure supplement 2—source data 1. [file elife-79183-fig4-figsupp2-data1.zip › Figure 4-figure supplement 2-souce data1/Figure 4-figure supplement 2B-souce data1/Figure 4-figure supplement 2B-souce data6-highlightedbandsandlabeled.pptx]

## Slide 1
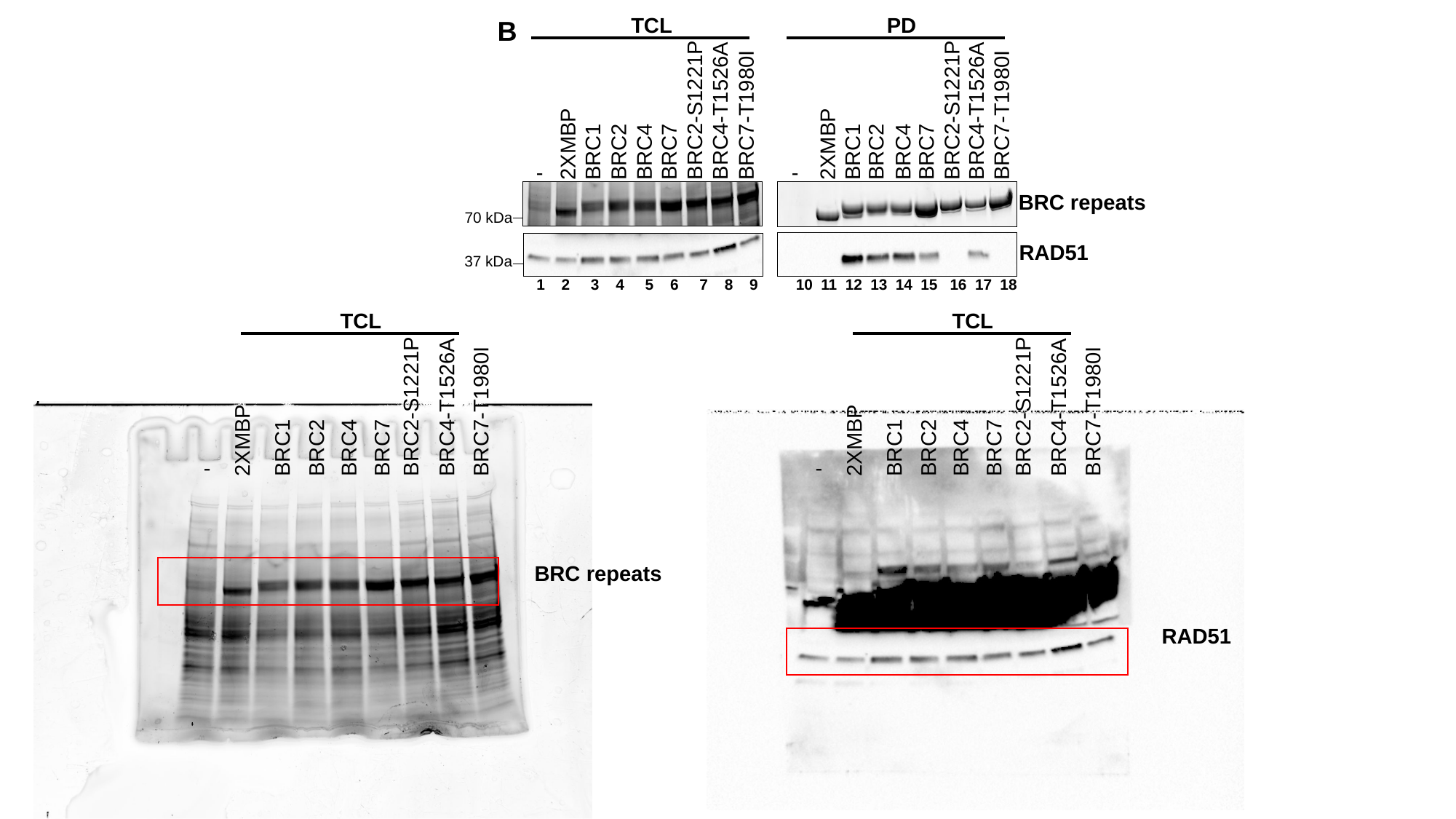

TCL
PD
B
BRC2-S1221P
BRC2-S1221P
BRC4-T1526A
BRC4-T1526A
BRC7-T1980I
BRC7-T1980I
2XMBP
2XMBP
BRC1
BRC2
BRC4
BRC7
BRC1
BRC2
BRC4
BRC7
-
-
BRC repeats
70 kDa
RAD51
37 kDa
 10 11 12 13 14 15 16 17 18
 1 2 3 4 5 6 7 8 9
TCL
TCL
BRC2-S1221P
BRC2-S1221P
BRC4-T1526A
BRC4-T1526A
BRC7-T1980I
BRC7-T1980I
2XMBP
2XMBP
BRC1
BRC2
BRC4
BRC7
BRC1
BRC2
BRC4
BRC7
-
-
BRC repeats
RAD51

## Slide 2
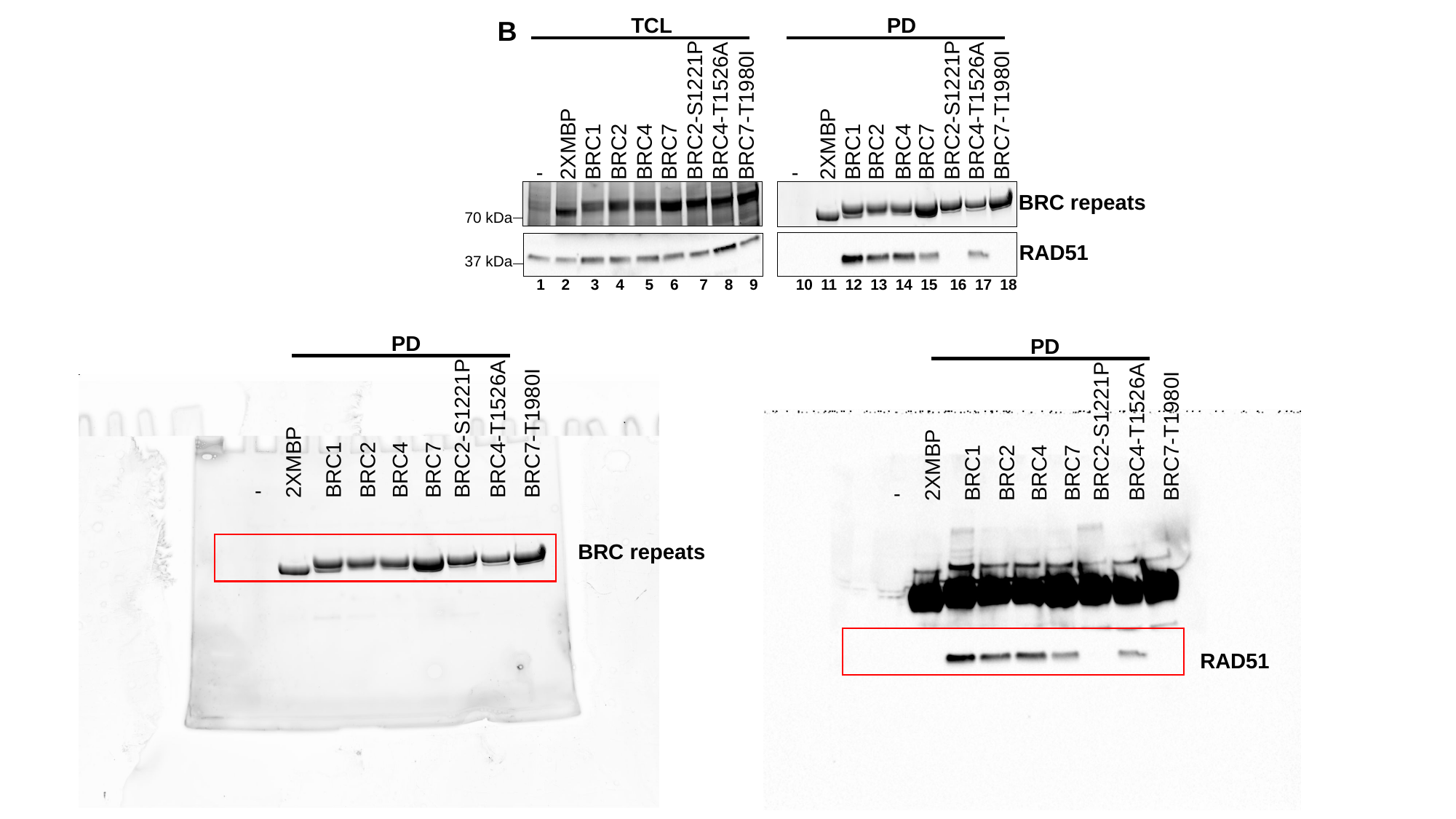

TCL
PD
B
BRC2-S1221P
BRC2-S1221P
BRC4-T1526A
BRC4-T1526A
BRC7-T1980I
BRC7-T1980I
2XMBP
2XMBP
BRC1
BRC2
BRC4
BRC7
BRC1
BRC2
BRC4
BRC7
-
-
BRC repeats
70 kDa
RAD51
37 kDa
 10 11 12 13 14 15 16 17 18
 1 2 3 4 5 6 7 8 9
PD
PD
BRC2-S1221P
BRC4-T1526A
BRC2-S1221P
BRC4-T1526A
BRC7-T1980I
BRC7-T1980I
2XMBP
2XMBP
BRC1
BRC2
BRC4
BRC7
BRC1
BRC2
BRC4
BRC7
-
-
BRC repeats
RAD51
