# Supplementary material for: BRCA2 BRC missense variants disrupt RAD51-dependent DNA repair
Source: eLife. 2022 Sep 13;11:e79183. doi: 10.7554/eLife.79183 (PMC9545528; doi:10.7554/eLife.79183)
Supplement: Figure 4—figure supplement 2—source data 1. [file elife-79183-fig4-figsupp2-data1.zip › Figure 4-figure supplement 2-souce data1/Figure 4-figure supplement 2C-souce data1/Figure 4-figure supplement 2C-souce data4-highlightedbandsandlabeled.pptx]

## Slide 1
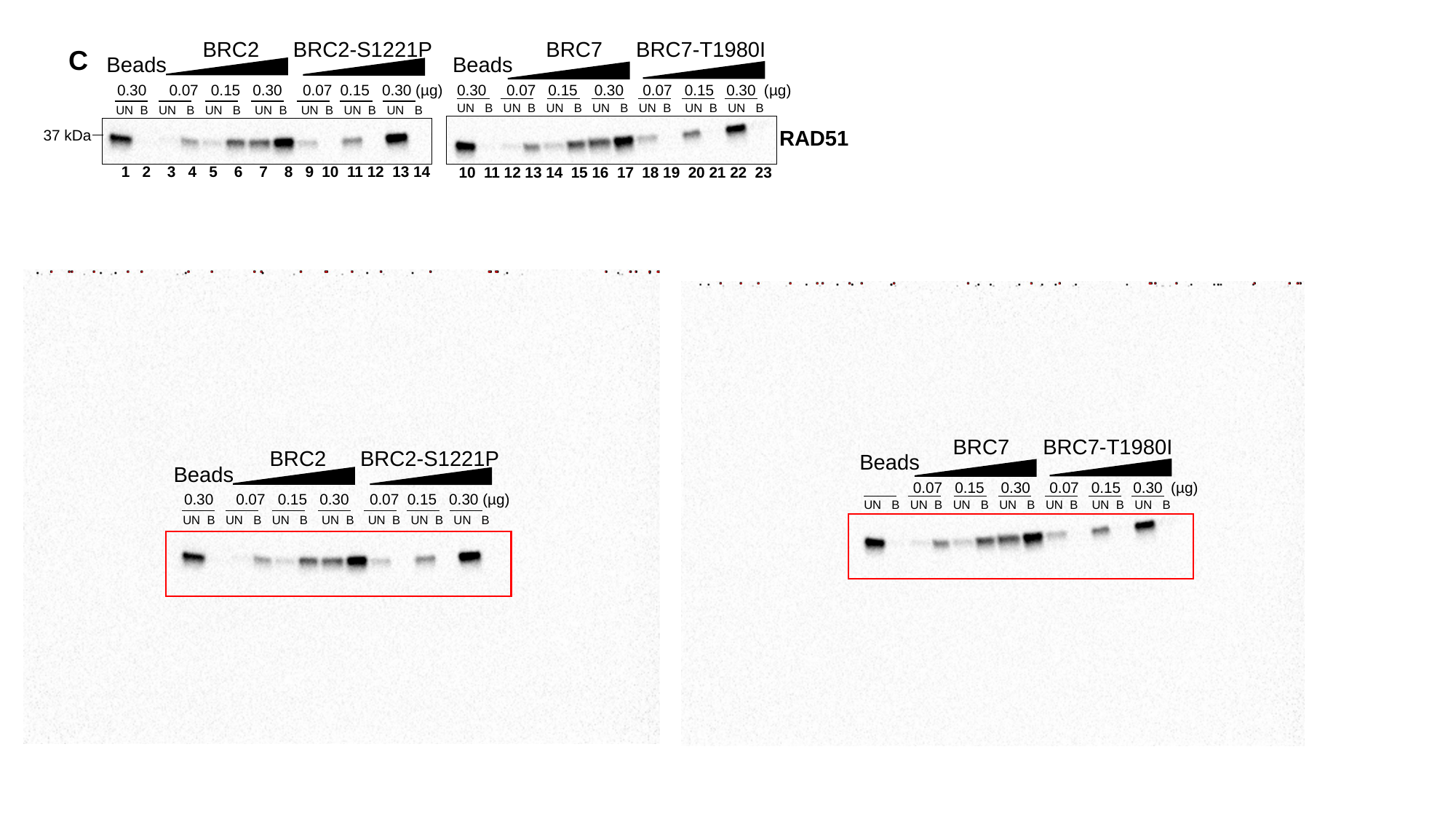

BRC2
BRC2-S1221P
BRC7
BRC7-T1980I
C
Beads
Beads
 0.30
0.07 0.15 0.30
0.07 0.15 0.30 (µg)
 0.30
0.07 0.15 0.30
0.07 0.15 0.30 (µg)
 UN B UN B UN B UN B UN B UN B UN B
 UN B UN B UN B UN B UN B UN B UN B
D
RAD51
 37 kDa
 1 2 3 4 5 6 7 8 9 10 11 12 13 14
 10 11 12 13 14 15 16 17 18 19 20 21 22 23
BRC7
BRC7-T1980I
BRC2
BRC2-S1221P
Beads
Beads
0.07 0.15 0.30
0.07 0.15 0.30 (µg)
 0.30
0.07 0.15 0.30
0.07 0.15 0.30 (µg)
 UN B UN B UN B UN B UN B UN B UN B
 UN B UN B UN B UN B UN B UN B UN B
