# Supplementary material for: BRCA2 BRC missense variants disrupt RAD51-dependent DNA repair
Source: eLife. 2022 Sep 13;11:e79183. doi: 10.7554/eLife.79183 (PMC9545528; doi:10.7554/eLife.79183)
Supplement: Figure 4—figure supplement 2—source data 1. [file elife-79183-fig4-figsupp2-data1.zip › Figure 4-figure supplement 2-souce data1/Figure 4-figure supplement 2D-souce data1/Figure 4-figure supplement 2D-souce data6-highlightedbandsandlabeled.pptx]

## Slide 1
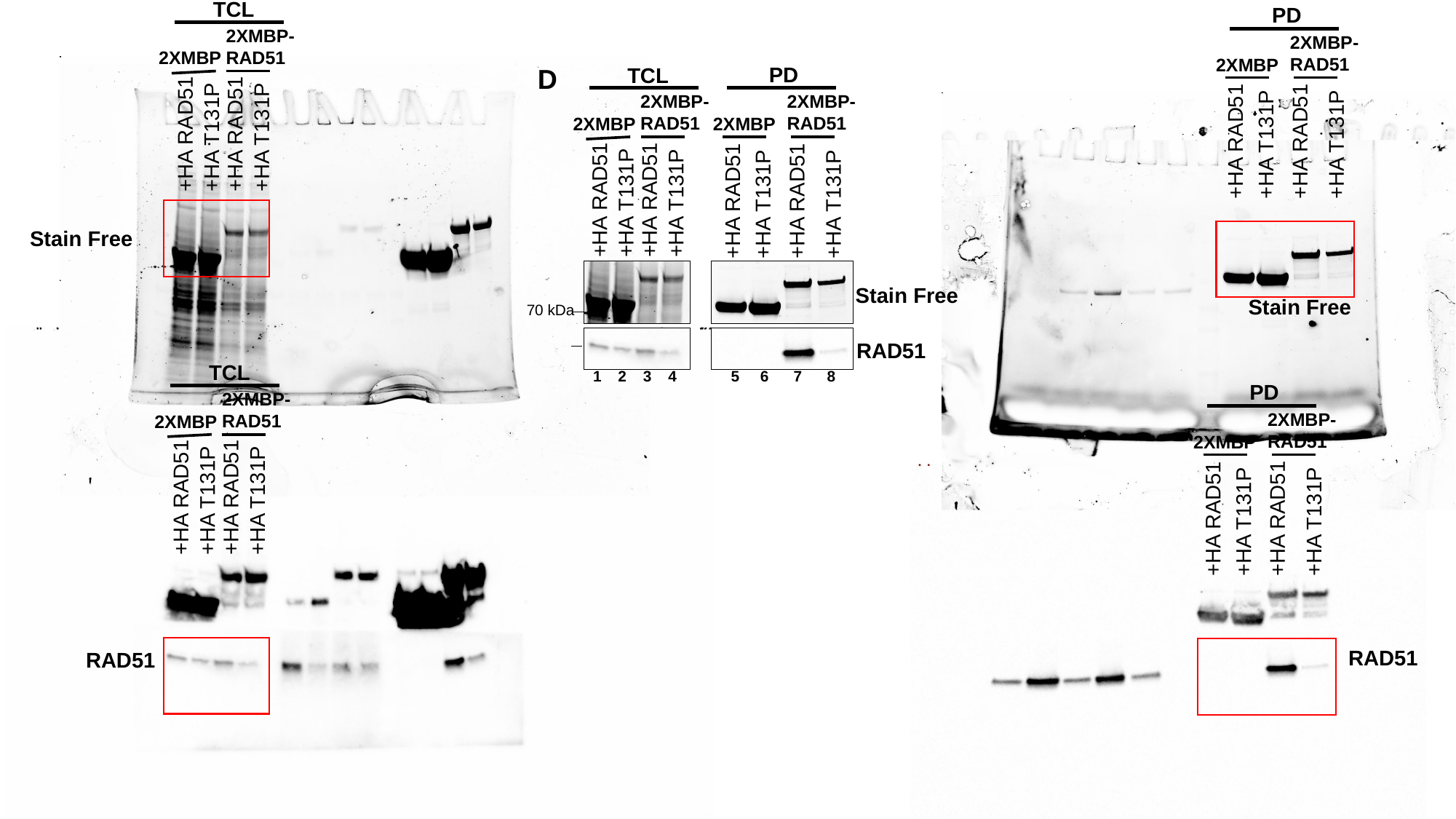

TCL
PD
2XMBP-
RAD51
2XMBP-
RAD51
2XMBP
2XMBP
PD
D
TCL
2XMBP-
RAD51
2XMBP-
RAD51
2XMBP
2XMBP
+HA RAD51
+HA RAD51
+HA T131P
+HA T131P
+HA RAD51
+HA RAD51
+HA T131P
+HA T131P
+HA RAD51
+HA RAD51
+HA RAD51
+HA RAD51
+HA T131P
+HA T131P
+HA T131P
+HA T131P
Stain Free
Stain Free
Stain Free
 70 kDa
RAD51
TCL
 1 2 3 4 5 6 7 8
PD
2XMBP-
RAD51
2XMBP-
RAD51
2XMBP
2XMBP
+HA RAD51
+HA RAD51
+HA T131P
+HA T131P
+HA RAD51
+HA RAD51
+HA T131P
+HA T131P
RAD51
RAD51
