# Supplementary material for: BRCA2 BRC missense variants disrupt RAD51-dependent DNA repair
Source: eLife. 2022 Sep 13;11:e79183. doi: 10.7554/eLife.79183 (PMC9545528; doi:10.7554/eLife.79183)
Supplement: Figure 4—figure supplement 2—source data 1. [file elife-79183-fig4-figsupp2-data1.zip › Figure 4-figure supplement 2-souce data1/Figure 4-figure supplement 2E-souce data1/Figure 4-figure supplement 2E-souce data4-highlightedbandsandlabeled.pptx]

## Slide 1
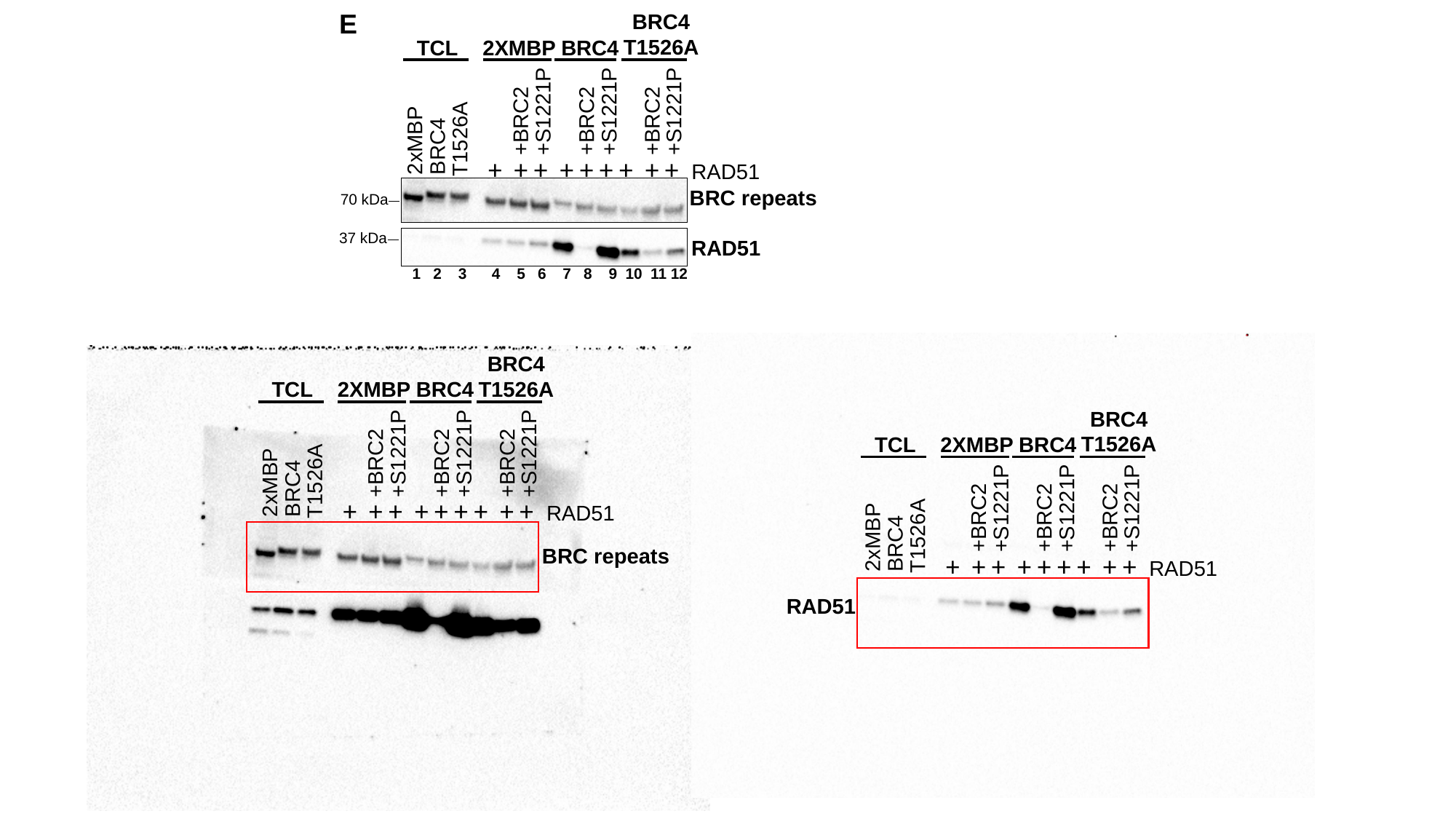

E
BRC4 T1526A
TCL
2XMBP
BRC4
T1526A
+S1221P
+S1221P
+S1221P
+BRC2
+BRC2
+BRC2
2xMBP
BRC4
+ + + + + + + + + RAD51
BRC repeats
 70 kDa
 37 kDa
RAD51
 1 2 3 4 5 6 7 8 9 10 11 12
BRC4 T1526A
TCL
2XMBP
BRC4
BRC4 T1526A
T1526A
TCL
2XMBP
BRC4
+S1221P
+S1221P
+S1221P
+BRC2
+BRC2
+BRC2
2xMBP
BRC4
T1526A
+ + + + + + + + + RAD51
+S1221P
+S1221P
+S1221P
+BRC2
+BRC2
+BRC2
2xMBP
BRC4
BRC repeats
+ + + + + + + + + RAD51
RAD51
