# Supplementary material for: BRCA2 BRC missense variants disrupt RAD51-dependent DNA repair
Source: eLife. 2022 Sep 13;11:e79183. doi: 10.7554/eLife.79183 (PMC9545528; doi:10.7554/eLife.79183)
Supplement: Figure 5—source data 1. [file elife-79183-fig5-data1.zip › Figure 5-source data 1/Figure 5B-source data/Figure 5B-source data3-highlightedbandsandlabeled.pptx]

## Slide 1
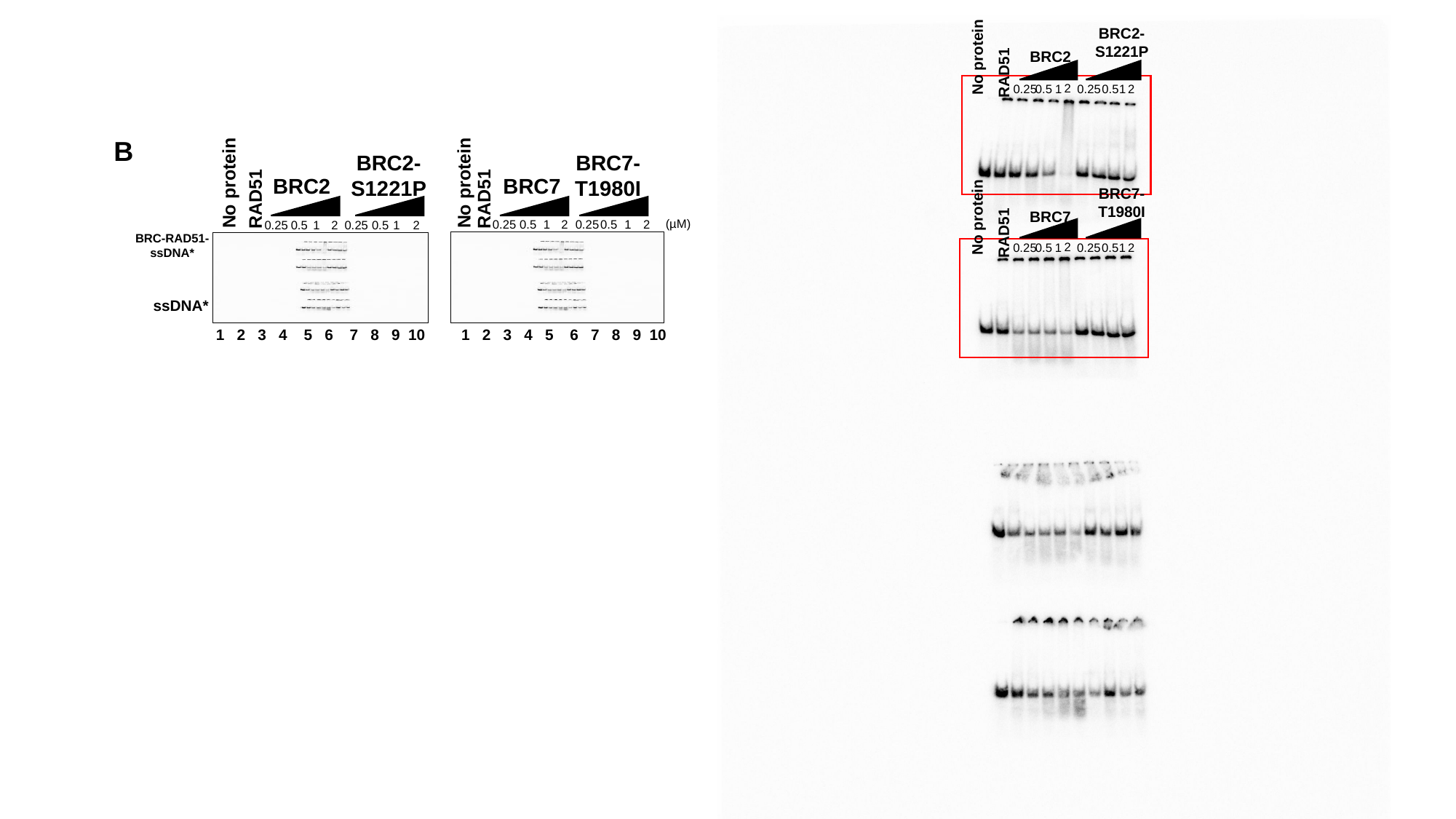

BRC2-
S1221P
BRC2
No protein
RAD51
2
0.25
0.5
1
0.25
0.5
1
2
B
BRC2-
S1221P
BRC7-
T1980I
No protein
No protein
BRC2
BRC7
BRC7-
T1980I
RAD51
RAD51
BRC7
No protein
(µM)
0.25 0.5 1
2
0.25
0.5
1
2
0.25
0.5
1
2
0.25
0.5
1
2
RAD51
BRC-RAD51-ssDNA*
2
0.25
0.5
1
0.25
0.5
1
2
ssDNA*
1 2 3 4 5 6 7 8 9 10
1 2 3 4 5 6 7 8 9 10
