# Supplementary material for: BRCA2 BRC missense variants disrupt RAD51-dependent DNA repair
Source: eLife. 2022 Sep 13;11:e79183. doi: 10.7554/eLife.79183 (PMC9545528; doi:10.7554/eLife.79183)
Supplement: Figure 6—figure supplement 1—source data 1. [file elife-79183-fig6-figsupp1-data1.zip › Figure 6-figure supplement 1-source data1/Figure 6-figure supplement 1B-source data1/Figure 6-figure supplement 1B-source data5-highlightedbandandlabeled.pptx]

## Slide 1
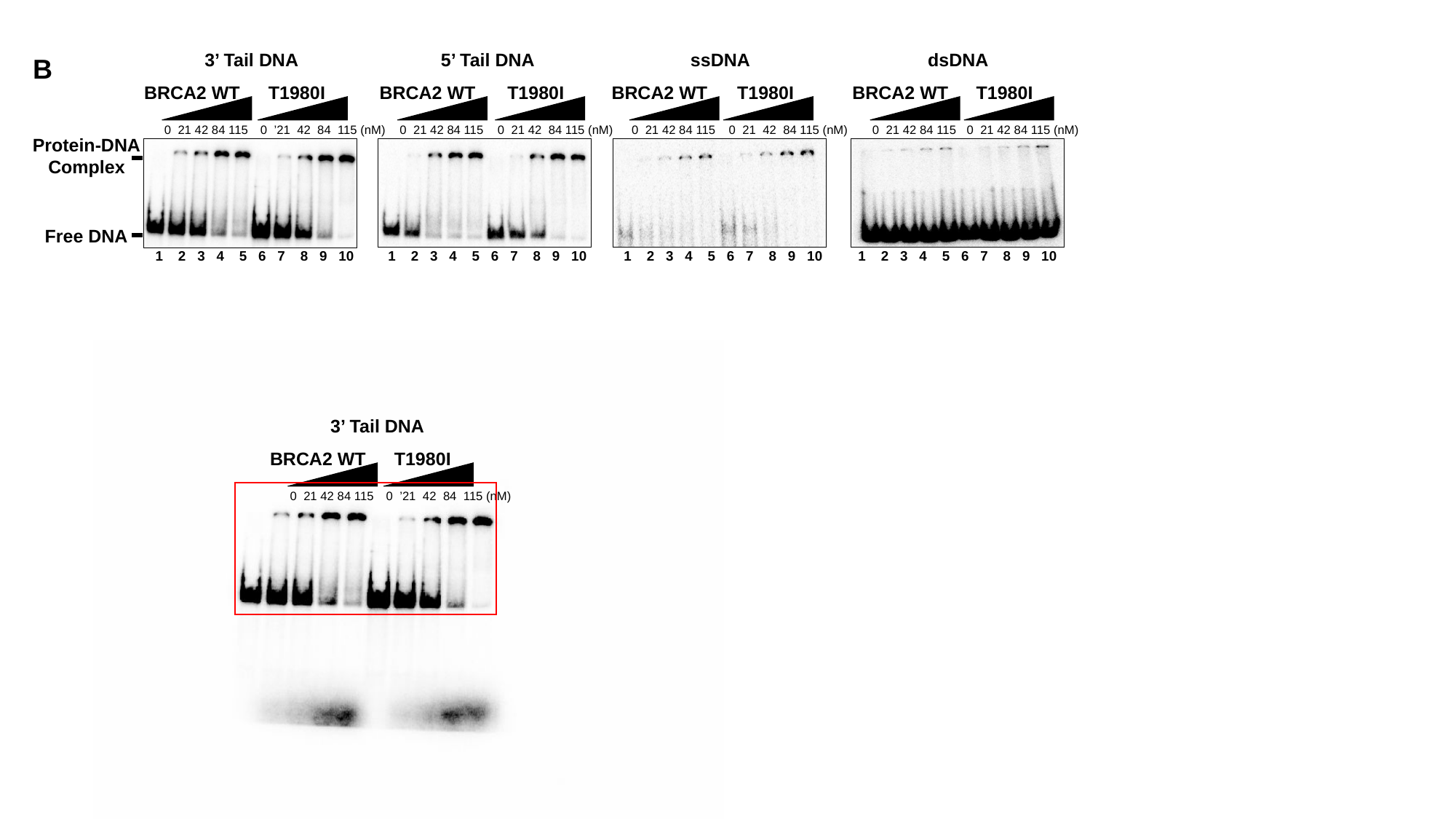

3’ Tail DNA
5’ Tail DNA
ssDNA
dsDNA
B
BRCA2 WT
T1980I
BRCA2 WT
T1980I
BRCA2 WT
T1980I
BRCA2 WT
T1980I
0 21 42 84 115
0 ’21 42 84 115 (nM)
0 21 42 84 115
0 21 42 84 115 (nM)
0 21 42 84 115
0 21 42 84 115 (nM)
0 21 42 84 115
0 21 42 84 115 (nM)
Protein-DNA
Complex
Free DNA
 1 2 3 4 5 6 7 8 9 10
 1 2 3 4 5 6 7 8 9 10
 1 2 3 4 5 6 7 8 9 10
 1 2 3 4 5 6 7 8 9 10
3’ Tail DNA
BRCA2 WT
T1980I
0 21 42 84 115
0 ’21 42 84 115 (nM)

## Slide 2
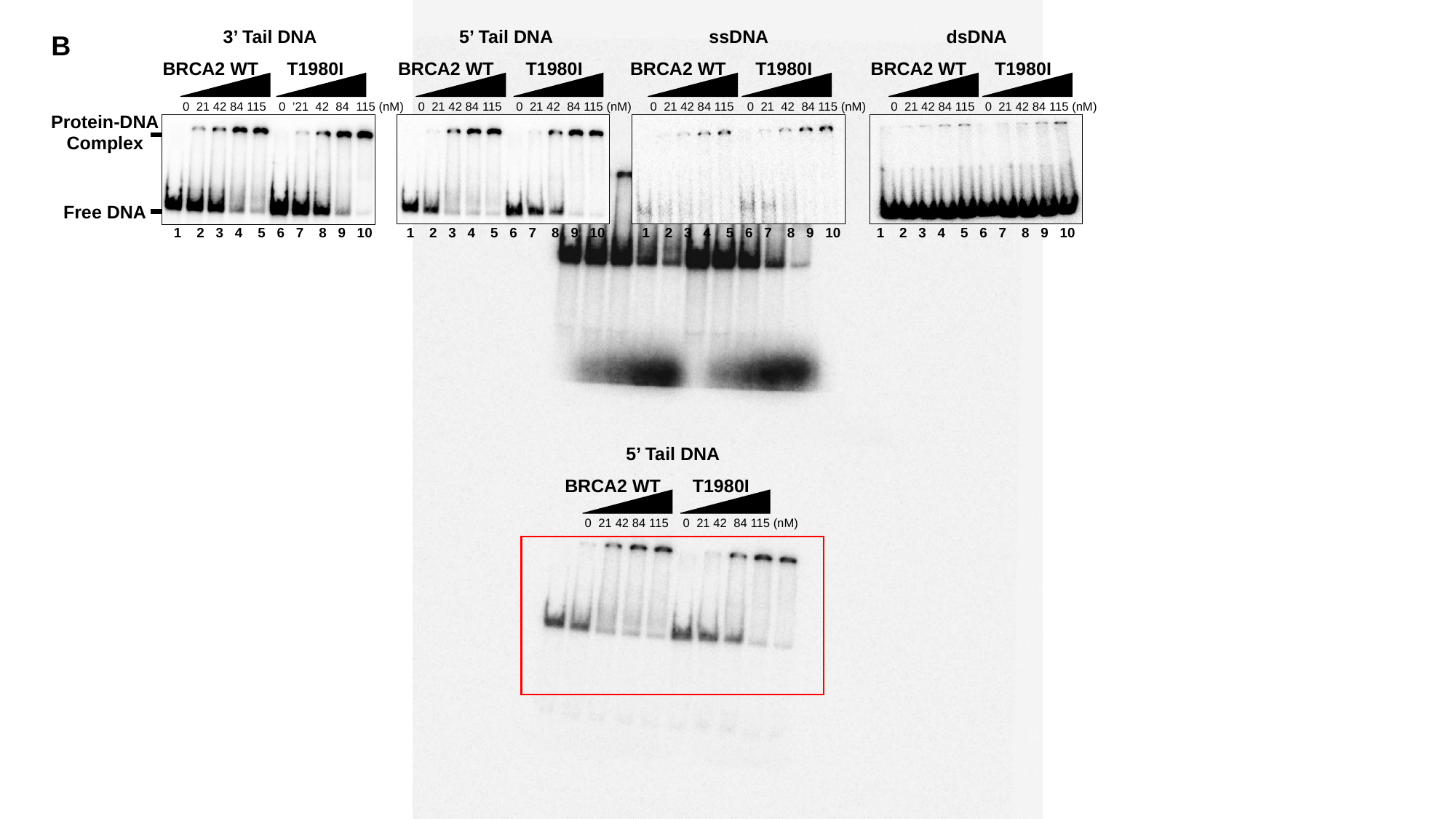

3’ Tail DNA
5’ Tail DNA
ssDNA
dsDNA
B
BRCA2 WT
T1980I
BRCA2 WT
T1980I
BRCA2 WT
T1980I
BRCA2 WT
T1980I
0 21 42 84 115
0 ’21 42 84 115 (nM)
0 21 42 84 115
0 21 42 84 115 (nM)
0 21 42 84 115
0 21 42 84 115 (nM)
0 21 42 84 115
0 21 42 84 115 (nM)
Protein-DNA
Complex
Free DNA
 1 2 3 4 5 6 7 8 9 10
 1 2 3 4 5 6 7 8 9 10
 1 2 3 4 5 6 7 8 9 10
 1 2 3 4 5 6 7 8 9 10
5’ Tail DNA
BRCA2 WT
T1980I
0 21 42 84 115
0 21 42 84 115 (nM)

## Slide 3
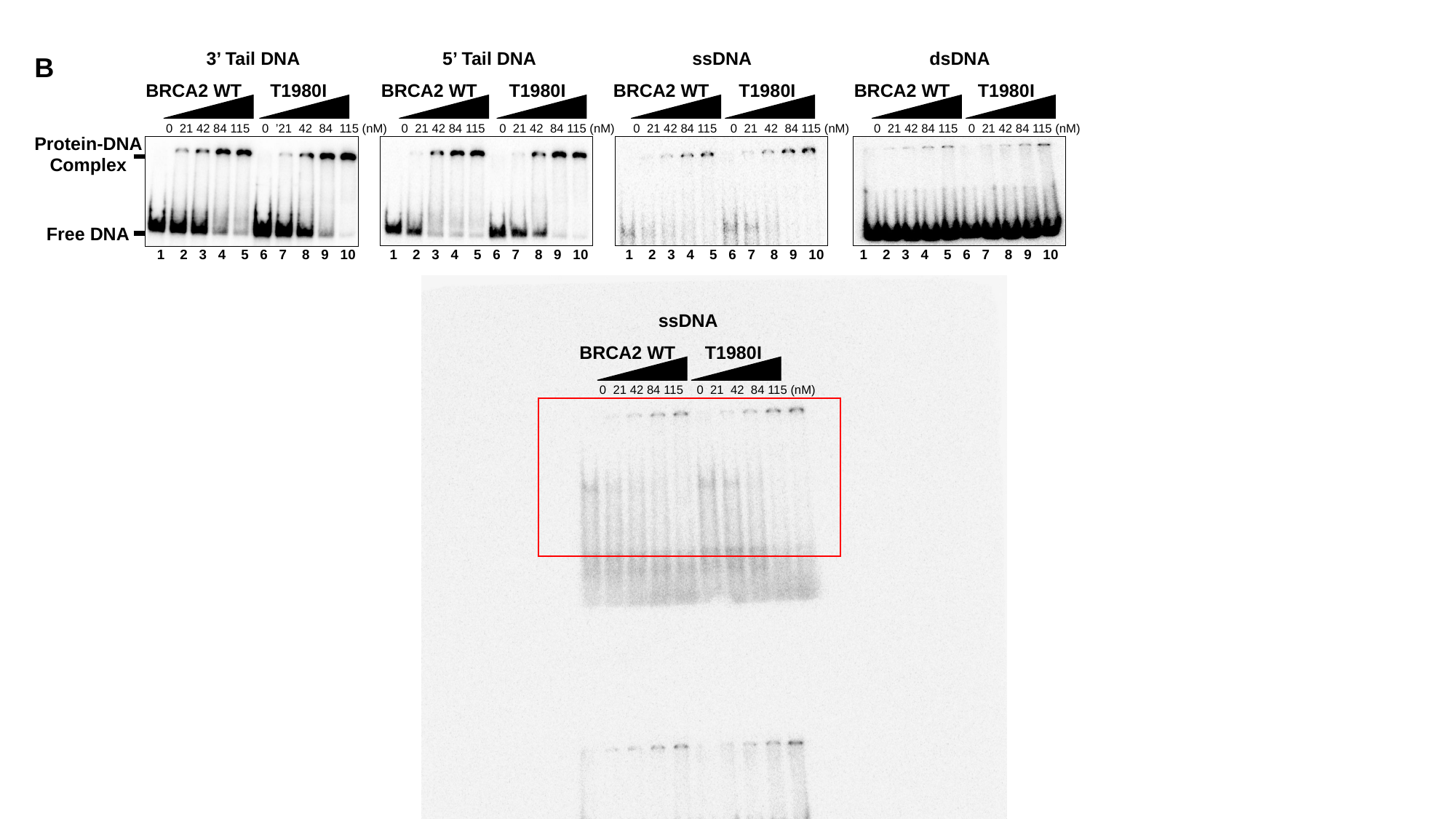

3’ Tail DNA
5’ Tail DNA
ssDNA
dsDNA
B
BRCA2 WT
T1980I
BRCA2 WT
T1980I
BRCA2 WT
T1980I
BRCA2 WT
T1980I
0 21 42 84 115
0 ’21 42 84 115 (nM)
0 21 42 84 115
0 21 42 84 115 (nM)
0 21 42 84 115
0 21 42 84 115 (nM)
0 21 42 84 115
0 21 42 84 115 (nM)
Protein-DNA
Complex
Free DNA
 1 2 3 4 5 6 7 8 9 10
 1 2 3 4 5 6 7 8 9 10
 1 2 3 4 5 6 7 8 9 10
 1 2 3 4 5 6 7 8 9 10
ssDNA
BRCA2 WT
T1980I
0 21 42 84 115
0 21 42 84 115 (nM)

## Slide 4
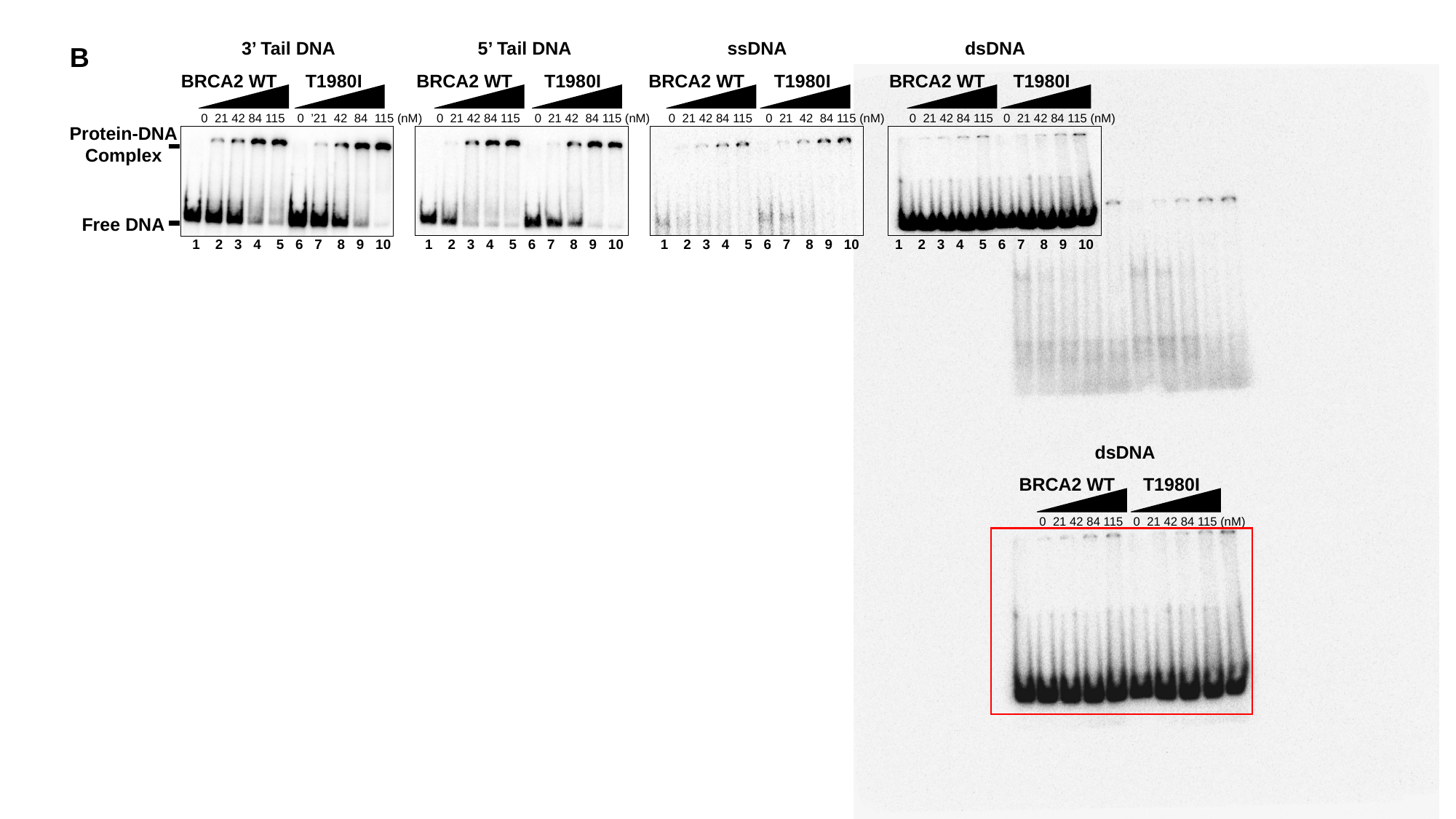

3’ Tail DNA
5’ Tail DNA
ssDNA
dsDNA
B
BRCA2 WT
T1980I
BRCA2 WT
T1980I
BRCA2 WT
T1980I
BRCA2 WT
T1980I
0 21 42 84 115
0 ’21 42 84 115 (nM)
0 21 42 84 115
0 21 42 84 115 (nM)
0 21 42 84 115
0 21 42 84 115 (nM)
0 21 42 84 115
0 21 42 84 115 (nM)
Protein-DNA
Complex
Free DNA
 1 2 3 4 5 6 7 8 9 10
 1 2 3 4 5 6 7 8 9 10
 1 2 3 4 5 6 7 8 9 10
 1 2 3 4 5 6 7 8 9 10
dsDNA
BRCA2 WT
T1980I
0 21 42 84 115
0 21 42 84 115 (nM)
